# Supplementary material for: Catalytic asymmetric Tsuji–Trost α−benzylation reaction of N-unprotected amino acids and benzyl alcohol derivatives
Source: Nat Commun. 2022 May 6;13:2509. doi: 10.1038/s41467-022-30277-9 (PMC9076619; doi:10.1038/s41467-022-30277-9)
Supplement: Supplementary file 1 — Supplementary Information [file 41467_2022_30277_MOESM1_ESM.pdf]

Supplementary Information

for

Catalytic asymmetric Tsuji–Trost α–benzylation reaction of *N*-unprotected amino acids and benzyl alcohol derivatives

Jian-Hua Liu, Wei Wen*, Jian Liao, Qi-Wen Shen, Zhu-Lian Wu, Tian Cai, Qi-Xiang Guo*

Key Laboratory of Applied Chemistry of Chongqing Municipality, and Chongqing Key Laboratory of Soft-Matter Material Chemistry and Function Manufacturing, School of Chemistry and Chemical Engineering, Southwest University, Chongqing, 400715, China.

Table of Contents

1. General data **1**

2. Supplementary tables for the reaction condition optimization **1**

3. Supplementary methods and analytic data for the catalytic asymmetric reaction **7**

4 . Determination of the absolute configuration of 5a **39**

5. Supplementary methods for the derivatization of products **39**

6. Supplementary figures for the reaction mechanism investigation **47**

7. Supplementary figures of ^{1}H and ^{13}C NMR spectrums **50**

8. Supplementary References **111**

1. General data

All non-aqueous reactions were carried out in a flame-dried glassware under nitrogen atmosphere or in a nitrogen-filled glove box unless otherwise noted. Solvents for reactions were dried appropriately before use: toluene, THF and Et_2O were dried by refluxing with sodium and benzophenone as indicator, CH_2Cl_2 and $CHCl_3$ were dried by refluxing with CaH_2. All other reagents were directly used as purchased from Aladdin, Adamas-beta® and Energy Chemical. ^1H NMR and ^{13}C NMR spectra were recorded on Bruker Avance 600 MHz or 400 MHz spectrometer. Chemical shifts (δ) are reported in ppm from tetramethylsilane (TMS) with the solvent resonance as the internal standard. Proton signal multiplicities are given as s(singlet), d (doublet), t (triplet), q (quartet), m (multiplet), br (broad) or a combination of them. J-values are in Hz. HRMS (ESI-Q-TOF) spectra were recorded on Bruker Impact-II mass spectrometer. Enantiomer ratios were determined by HPLC (Chiralpak AD-H, IC-H, OD-H columns were purchased from Daicel Chemical Industries, LTD). Optical rotations were determined at λ = 589 nm (sodium D line) by using a Rudolph-API automatic polarimeter. Arylmethyl tert-butyl carbonates,[1] amino acid ethyl esters[2] and chiral aldehydes catalysts[3] were prepared according to the literature.

2. Supplementary tables for the reaction condition optimization

Supplementary Table 1: Chiral aldehyde catalyst screening

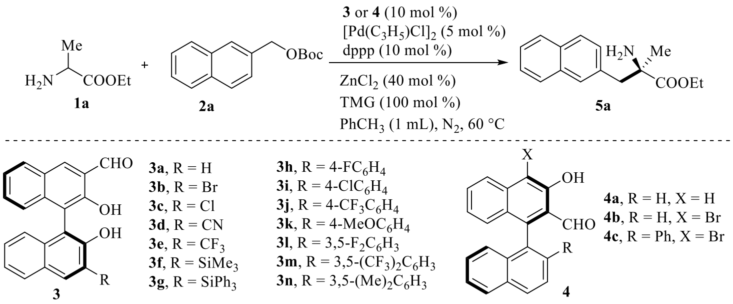

entry	3 or 4	time (h)	yield (%)[b]	ee (%)[c]
1	**3a**	8	85	68
2	**3b**	13	30	52
3	**3c**	18	44	60
4	**3d**	6	70	14
5	**3e**	15	49	56
6	**3f**	10	36	76
7	**3g**	9	28	39
8	**3h**	17	51	76
9	**3i**	8.5	85	70
10	**3j**	6	55	70

11	**3k**	10	35	72
12	**3l**	4	85	62
13	**3m**	10	75	58
14	**3n**	11	47	68
15	**4a**	22	25	66
16	**4b**	9	13	64
17	**4c**	9	6	78

[a] Unless noted otherwise, reactions were performed with **1a** (0.30 mmol), **2a** (0.20 mmol), **3** or **4** (0.02 mmol), dppp (0.02 mmol), $[Pd(C_3H_5)Cl]_2$ (0.01mmol), TMG (0.20 mmol), and $ZnCl_2$ (0.08 mmol) in toluene (1.0 mL) at 60 ºC. [b] Isolated yield. [c] Determined by chiral HPLC analysis.

Supplementary Table 2: Transition-metal screening

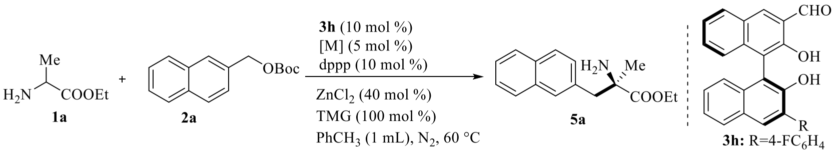

entry	M	time (h)	yield (%)[b]	ee (%)[c]
1	$[Pd(C_3H_5)Cl]_2$	17	51	76
2	$Pd(PPh_3)_4$	27	N.R.[d]	N.D.[e]
3	$Pd(OAc)_2$	22	N.R.	N.D.
4	$Pd(PPh_3)_2Cl_2$	17	N.R.	N.D.
5	$Pd_2(dba)_3$	27	N.R.	N.D.
6	$PdCl_2$	19	N.R.	N.D.
7	$Ni(cod)_2$	18	N.R.	N.D.

[a] Unless noted otherwise, reactions were performed with **1a** (0.30 mmol), **2a** (0.20 mmol), **3h** (0.02 mmol), dppp (0.02 mmol), [M] (0.01mmol), TMG (0.20 mmol), and $ZnCl_2$ (0.08 mmol) in toluene (1.0 mL) at 60º C. [b] Isolated yield. [c] Determined by chiral HPLC analysis. [d] N.R. = No reaction. [e] N.D. = Not determined.

Supplementary Table 3: Ligand screening

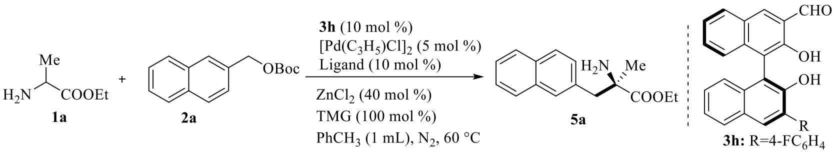

entry	ligand	time (h)	yield (%)[b]	ee (%)[c]
1	PPh_3[d]	22	N.R.[e]	N.D.[f]
2	dppf	19	Trace	N.D.
3	dppe	17	17	73
4	dppb	18	10	75
5	dppp	17	51	76
6	(±)BINAP	15	35	48
7	Tri(*o*-tolyl)phosphine[d]	17	Trace	N.D.

[a] Unless noted otherwise, reactions were performed with **1a** (0.30 mmol), **2** (0.20 mmol), **3h** (0.02 mmol), ligand (0.02 mmol), $[Pd(C_3H_5)Cl]_2$ (0.01mmol), TMG (0.20 mmol), and $ZnCl_2$ (0.08 mmol) in toluene (1.0 mL) at 60º C. [b] Isolated yield. [c] Determined by chiral HPLC analysis. [d] PPh_3 (0.04 mmol) and tri(*o*-tolyl)phosphine (0.04). [e] N.R. = No reaction. [f] N.D. = Not determined.

Supplementary Table 4: Lewis acid screening

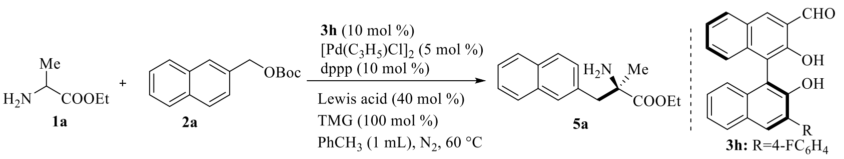

entry	lewis acid	time (h)	yield (%)[b]	ee (%)[c]
1	LiCl	23	Trace	N.D.[d]
2	$Cu(OTf)_2$	23	Trace	N.D.
3	$Bi(OTf)_2$	22	Trace	N.D.
4	$Sc(OTf)_3$	18	Trace	N.D.
5	$Zn(CN)_2$	18	Trace	N.D.
6	$ZnCl_2$	17	51	76
7	ZnF_2	19	20	66
8	$ZnBr_2$	17	17	58
9	$Zn(OAc)_2$	21	8	74
10	$Zn(OTf)_2$	11	46	6
11	$Zn(ClO_4)_2.6H_2O$	16	25	30
12	$AlCl_3$	16	10	36
13	$MgBr_2$	17	10	42
14	$Mg(OTf)_2$	17	33	35
15	$MgCl_2$	19	12	42
16	$NiBr_2$	18	26	12
17	$Ni(acac)_2$	16	41	62

[a] Unless noted otherwise, reactions were performed with **1a** (0.30 mmol), **2** (0.20 mmol), **3h** (0.02 mmol), dppp (0.02 mmol), $[Pd(C_3H_5)Cl]_2$ (0.01mmol), TMG (0.20 mmol), and lewis acid (0.08 mmol) in toluene (1.0 mL) at 60ºC. [b] Isolated yield. [c] Determined by chiral HPLC analysis. [d] N.D. = Not determined.

Supplementary Table 5: Base screening

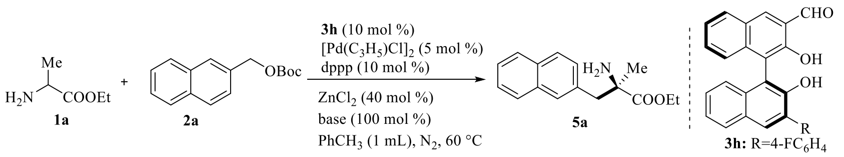

entry	base	time (h)	yield (%)[b]	ee (%)[c]
1	Et_3N	19	Trace	N.D.[d]
2	^{t}BuOK	13	Trace	N.D.
3	DABCO	14	Trace	N.D.
4	TBD	14	8	52
5	2-^{t}BuTMG[e]	10	12	15
6	DBN[f]	16	23	60
7	DBU	17	35	55
8	Cs_2CO_3	18	31	17
9	TMG[g]	17	51	76
10	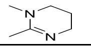	16	46	50

[a] Unless noted otherwise, reactions were performed with **1a** (0.30 mmol), **2a** (0.20 mmol), **3h** (0.02 mmol), dppp

(0.02 mmol), $[Pd(C_3H_5)Cl]_2$ (0.01mmol), base (0.20 mmol), and $ZnCl_2$ (0.08 mmol) in toluene (1.0 mL) at 60 °C. [b] Isolated yield. [c] Determined by chiral HPLC analysis. [d] N.D. = Not determined. [e]2-tertbutyl-1,1,3,3-tetramethylguanidine.[f]1,5-diazabicyclo [4.3.0] non-5-ene. [g] 1,1,3,3-tetramethylguanidine.

Supplementary Table 6: Screening of the alkoxyl groups of amino acid esters

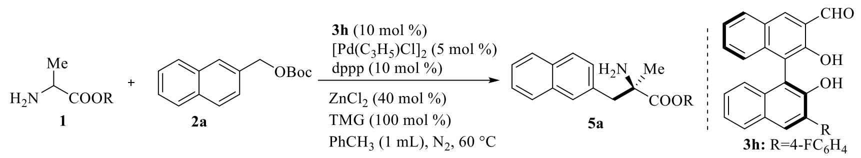

entry	R	time (h)	yield (%)[b]	ee (%)[c]
1	Me	15	60	69
2	Et	17	51	76
3	Bn	9	40	73
4	n-Pr	8	44	76

[a] Unless noted otherwise, reactions were performed with **1** (0.30 mmol), **2a** (0.20 mmol), **3h** (0.02 mmol), dppp (0.02 mmol), $[Pd(C_3H_5)Cl]_2$ (0.01mmol), TMG (0.20 mmol), and $ZnCl_2$ (0.08 mmol) in toluene (1.0 mL) at 60 ºC. [b] Isolated yield. [c] Determined by chiral HPLC analysis.

Supplementary Table 7: Leaving group screening

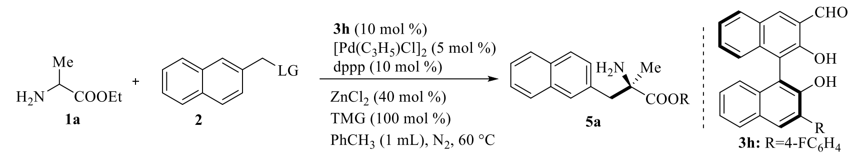

entry	LG	time (h)	yield (%)[b]	ee (%)[c]
1	OAc	15	3	18
2	OBoc	17	51	76
3	$O(CO)OCH_3$	15	55	64
4	O(CO)OEt	11	47	72
5	$O(OP)(OPh)_2$	10	45	32
6	$O(OP)(OEt)_2$	9	22	62

[a] Unless noted otherwise, reactions were performed with **1** (0.30 mmol), **2a** (0.20 mmol), **3h** (0.02 mmol), dppp (0.02 mmol), $[Pd(C_3H_5)Cl]_2$ (0.01mmol), TMG (0.20 mmol), and $ZnCl_2$ (0.08 mmol) in toluene (1.0 mL) at 60 ºC. [b] Isolated yield. [c] Determined by chiral HPLC analysis.

Supplementary Table 8: Solvent screening

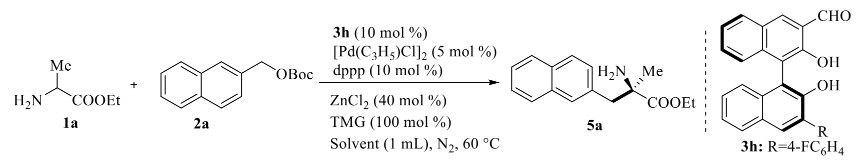

entry	solvent	time (h)	yield (%)[b]	ee (%)[c]
1	Toluene	17	51	76
2	*o*-xylene	20	Trace	N.D.[d]
3	*p*-xylene	20	3	70
4	Mesitylene	15	95	74
5	CH_3CN	16	12	3

6	DME	15	55	58
7	$PhCF_3$	15	50	56

[a] Unless noted otherwise, reactions were performed with **1a** (0.30 mmol), **2** (0.20 mmol), **3h** (0.02 mmol), dppp (0.02 mmol), $[Pd(C_3H_5)Cl]_2$ (0.01mmol), TMG (0.20 mmol), and $ZnCl_2$ (0.08 mmol) in svolent (1.0 mL) at 60° C. [b] Isolated yield. [c] Determined by chiral HPLC analysis. [d] N.D. = Not determined.

Supplementary Table 9: Screening of the equivalents of TMG

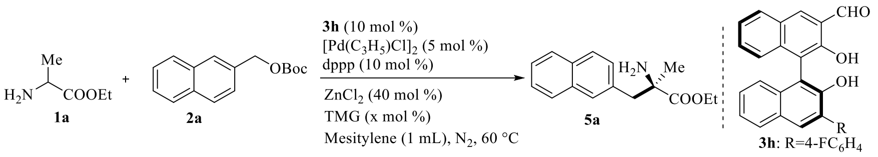

entry	TMG (x mol%)	time (h)	yield (%)[b]	ee (%)[c]
1	40	5	90	72
2	60	5	95	72
3	80	5	92	72
4	100	5	95	74

[a] Unless noted otherwise, reactions were performed with **1a** (0.30 mmol), **2a** (0.20 mmol), **3h** (0.02 mmol), dppp (0.02 mmol), $[Pd(C_3H_5)Cl]_2$ (0.01mmol), TMG (x mmol), and $ZnCl_2$ (0.08 mmol) in mesitylene (1.0 mL) at 60° C. [b] Isolated yield. [c] Determined by chiral HPLC analysis.

Supplementary Table 10: Re-screening of chiral aldehyde catalysts

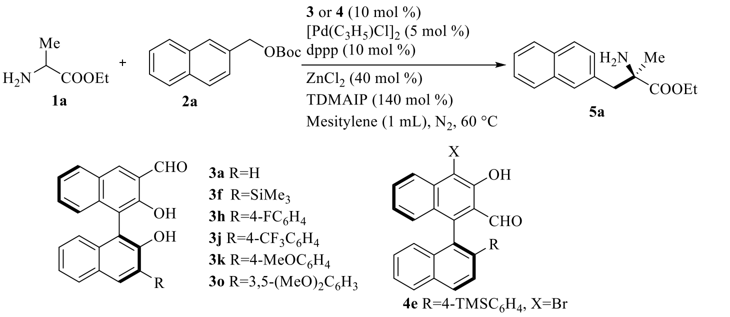

entry	3 or 4	time (h)	yield (%)[b]	ee (%)[c]
1	**3a**	12	15	80
2	**3f**	3.5	94	84
3	**3h**	7	70	84
4	**3j**	13	73	74
5	**3k**	12	77	78
6	**3o**	15	5	71
7	**4e**	10	Trace	N.D.[d]

[a] Unless noted otherwise, reactions were performed with **1a** (0.30 mmol), **2a** (0.20 mmol), **3** or **4** (0.02 mmol), dppp (0.02 mmol), $[Pd(C_3H_5)Cl]_2$ (0.01mmol), TDMAIP (0.28 mmol), and $ZnCl_2$ (0.08 mmol) in mesitylene (1.0 mL) at 60° C. [b] Isolated yield. [c] Determined by chiral HPLC analysis. [d] N.D. = Not determined.

Supplementary Table 11: Screening of the equivalents of Lewis acid

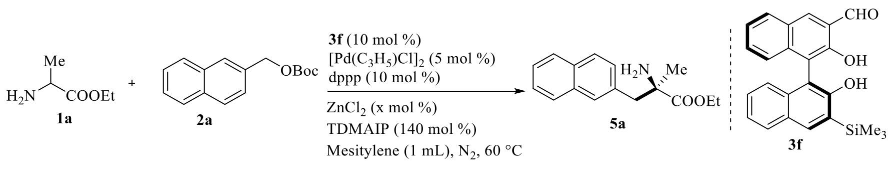

entry	x	time (h)	yield (%)[b]	ee (%)[c]
1	0	7	34	20
2	20	5	90	84
3	40	3.5	94	84
4	60	5	66	90

[a] Unless noted otherwise, reactions were performed with **1a** (0.30 mmol), **2a** (0.20 mmol), **3f** (0.02 mmol), dppp (0.02 mmol), $[Pd(C_3H_5)Cl]_2$ (0.01mmol), TDMAIP (0.28 mmol), and $ZnCl_2$ (x mmol) in mesitylene (1.0 mL) at 60 °C. [b] Isolated yield. [c] Determined by chiral HPLC analysis.

Supplementary Table 12: Reactant concentration screening

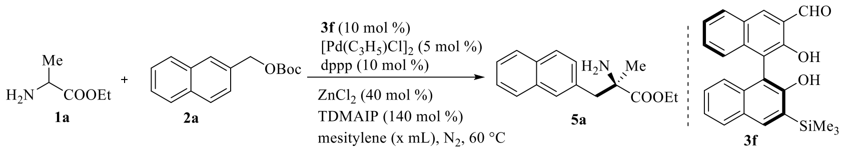

entry	x	time (h)	yield (%)[b]	ee (%)[c]
1	1	3.5	94	84
2	0.5	3.5	93	90
3	0.3	5	61	79

[a] Unless noted otherwise, reactions were performed with **1a** (0.30 mmol), **2a** (0.20 mmol), **3f** (0.02 mmol), dppp (0.02 mmol), $[Pd(C_3H_5)Cl]_2$ (0.01mmol), TDMAIP (0.28 mmol), and $ZnCl_2$ (0.08 mmol) in mesitylene at 60 °C. [b] Isolated yield. [c] Determined by chiral HPLC analysis.

Supplementary Table 13: Screening of the equivalents of base TDMAIP

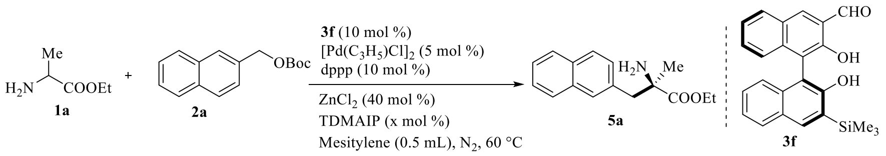

entry	x	time (h)	yield (%)[b]	ee (%)[c]
1	100	3.5	90	90
2	120	3.5	93	88
3	140	3.5	93	90

[a] Unless noted otherwise, reactions were performed with **1a** (0.30 mmol), **2a** (0.20 mmol), **3f** (0.02 mmol), dppp (0.02 mmol), $[Pd(C_3H_5)Cl]_2$ (0.01mmol), TDMAIP (x mmol), and $ZnCl_2$ (0.08 mmol) in mesitylene (0.5 mL) at 60 °C. [b] Isolated yield. [c] Determined by chiral HPLC analysis.

Supplementary Table 14: Reaction condition optimization-II

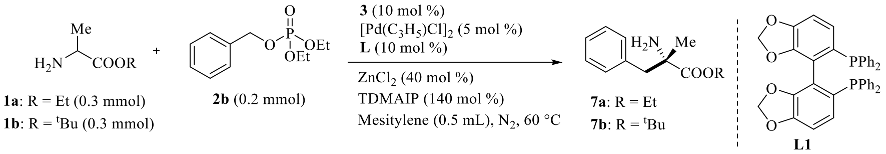

entry	3	R	L	Time (h)	Yield (%)[b]	ee (%)[c]
1[e]	**3a**	Et	(*S*)-L1	12	51	19
2[e]	**3a**	Et	(*R*)-L1	12	60	72
3	**3f**	Et	dppp	12	26	75
4[d]	**3f**	Et	dppp	12	25	78

5[e]	**3f**	Et	dppp	9	56	76
6[e]	**3f**	Et	(*R*)-L1	9	60	78
7	***rac*-3a**	^{t}Bu	(*R*)-L1	12	62	74
8[e]	**3f**	^{t}Bu	dppp	9.5	50	83
9[e]	**3f**	^{t}Bu	(*R*)-L1	9.5	91	93
10	**3f**	^{t}Bu	(*R*)-L1	9.5	83	95

[a] Unless noted otherwise, reactions were performed with **1a** or **1b** (0.30 mmol), **2b** (0.20 mmol), **3** (0.02 mmol), **L** (0.02 mmol), $[Pd(C_3H_5)Cl]_2$ (0.01mmol), TDMAIP (0.28 mmol), and $ZnCl_2$ (0.08 mmol) in mesitylene (0.5 mL) at 60 ºC. [b] Isolated yield. [c] Determined by chiral HPLC analysis. [d] **3f** (0.04 mmol). [e] **L** (0.04 mmol), $[Pd(C_3H_5)Cl]_2$ (0.02 mmol).

3. Supplementary methods and analytic data for the catalytic asymmetric reaction

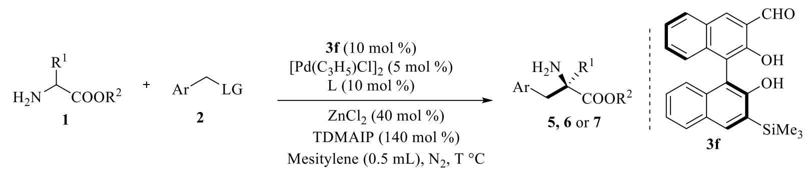

To a 10 mL vial charged with $[Pd(C_3H_5)Cl]_2$ (3.6 mg, 0.01 mmol) and ligand (dppp or R-L1) (0.02 mmol) was added 0.5 mL mesitylene, and the mixture was stirred under nitrogen atmosphere at room temperature for 30 min. Then, ethyl amino acid ester **1** (0.3 mmol), benzyl aocohol derivative **2** (0.2 mmol), chiral aldehyde **3f** (7.7 mg, 0.02 mmol), $ZnCl_2$ (10.9 mg, 0.08 mmol) and TDMAIP (50.9 uL, 0.28 mmol) were added. The mixture was continuously stirred at indicated reaction temperature under nitrogen atmosphere. After the reaction completed, the solvent was removed by rotary evaporation, and the residue was purified by flash chromatography column on silica gel (eluent: petroleum ether/ ethyl acetate/ triethylamine =200/100/3).

Ethyl (S)-2-amino-2-methyl-3-(naphthalen-2-yl)propanoate (5a):

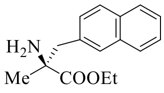

Colorless oil (47.9 mg, 93%); R_f = 0.25 (petroleum ether/ ethyl acetate = 2:1); the enantiomeric excess was determined to be 90% by HPLC analysis on Daicel Chirapak AD-H column (hexane/isopropanol = 90/10, flow rate 1.0 mL/min, T = 30 ºC), UV 254 nm, t_R(major) 10.976 min, t_R(minor) 7.591 min; $[\alpha]_D^{25}$ = -35.81 (c=0.85, $CHCl_3$); **^{1}H NMR (400 MHz, $CDCl_3$)** δ 7.88 – 7.72 (m, 3H), 7.64 (s, 1H), 7.48 – 7.38 (m, 2H), 7.30 (m, 1H), 4.16 (q, *J* = 7.2 Hz, 2H), 3.30 (d, *J* = 13.2 Hz, 1H), 2.97 (d, *J* = 13.2 Hz, 1H), 1.69 (s, 2H), 1.43 (s, 3H), 1.25 (t, *J* = 7.2 Hz, 3H); **^{13}C NMR (101 MHz, $CDCl_3$)** δ 177.06, 134.24, 133.37, 132.45, 128.78, 128.29, 127.79, 127.63, 127.59, 126.01, 125.61, 61.12, 58.81, 46.94, 26.83, 14.21; **HRMS(ESI)** m/z: $[M+H]^+$ Calculated for $C_{16}H_{20}NO_2^+$ 258.1489; found 258.1479.

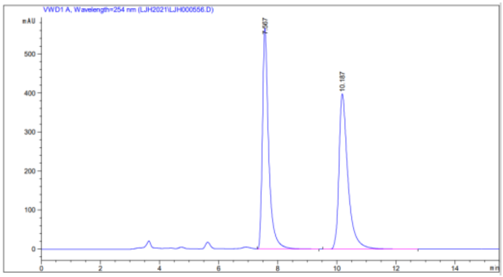

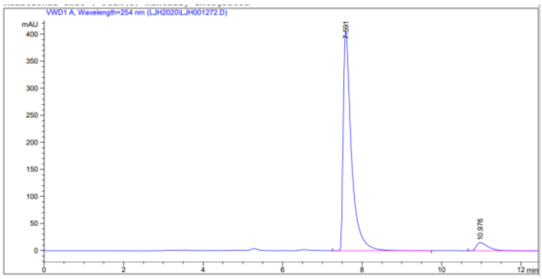

Peak #	RetTime [min]	Type	Width [min]	Area [mAU*s]	Height [mAU]	Area %
1	7.567	BB	0.2140	8251.56152	565.38098	49.7671
2	10.187	BB	0.3100	8328.79785	397.54800	50.2329

Peak #	RetTime [min]	Type	Width [min]	Area [mAU*s]	Height [mAU]	Area %
1	7.591	BB	0.2113	5823.79443	405.57529	94.8602
2	10.976	BBA	0.3090	315.55240	15.31036	5.1398

Ethyl (S)-2-amino-2-(naphthalen-2-ylmethyl)butanoate (5b):

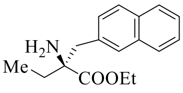

Colorless oil (50.8 mg, 93%); R_f= 0.26 (petroleum ether/ ethyl acetate = 5:1); the enantiomeric excess was determined to be 88% by HPLC analysis on Daicel Chirapak AD-H column (hexane/isopropanol = 90/10, flow rate 1.0 mL/min, T = 30 ºC), UV 254 nm, t_R(major) 10.677 min, t_R(minor) 7.340 min; $[\alpha]_D^{25}$= -45.07 (c=0.86, $CHCl_3$); **^{1}H NMR (600 MHz, $CDCl_3$)** δ 7.87 – 7.70 (m, 3H), 7.64 (s, 1H), 7.50 – 7.38 (m, 2H), 7.33 – 7.27 (m, 1H), 4.18 (q, *J* = 7.2 Hz, 2H), 3.35 (d, *J* = 13.2 Hz, 1H), 2.92 (d, *J* = 13.2 Hz, 1H), 2.01 (m, 1H), 1.76 – 1.64 (m, 1H), 1.61 (s, 2H), 1.27 (t, *J* = 7.2 Hz, 3H), 0.92 (t, *J* = 7.8 Hz, 3H); **^{13}C NMR (151 MHz, $CDCl_3$)** δ 176.52, 134.20, 133.40, 132.46, 128.79, 128.25, 127.86, 127.63, 127.60, 126.02, 125.60, 62.53, 61.01, 45.90, 33.40, 14.33, 8.37; **HRMS(ESI)** m/z: $[M+H]^+$ Calculated for $C_{17}H_{22}NO_2^+$ 272.1645; found 272.1640.

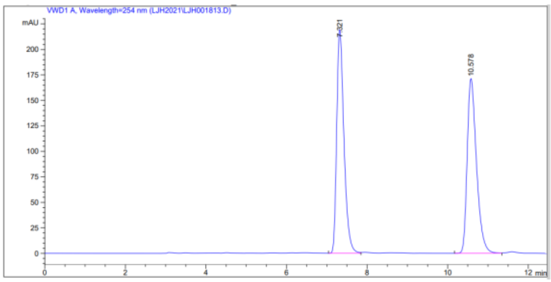

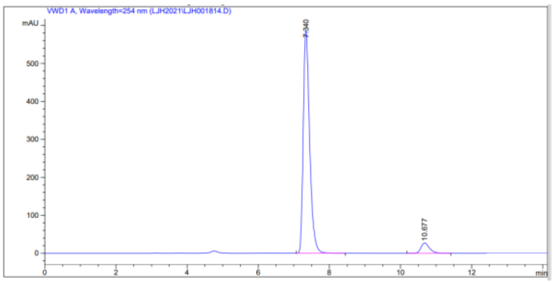

Peak #	RetTime [min]	Type	Width [min]	Area [mAU*s]	Height [mAU]	Area %
1	7.321	BV	0.1895	2738.86182	219.27248	49.9713
2	10.578	BV	0.2450	2742.01294	171.37408	50.0287

Peak #	RetTime [min]	Type	Width [min]	Area [mAU*s]	Height [mAU]	Area %
1	7.340	VB	0.1882	7274.92920	587.39520	94.2273
2	10.677	BB	0.2524	445.68530	26.98899	5.7727

Ethyl (S)-2-amino-2-(naphthalen-2-ylmethyl)pentanoate (5c):

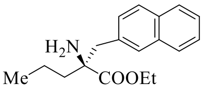

A pale yellow oil (50.5 mg, 89%); R_f= 0.24 (petroleum ether/ ethyl acetate = 5:1); the enantiomeric excess was determined to be 90% by HPLC analysis on Daicel Chirapak AD-H column (hexane/isopropanol = 90/10, flow rate 1.0 mL/min, T = 30 ºC), UV 254 nm, t_R(major) 9.878min, t_R(minor) 7.081 min; $[\alpha]_D^{25}$= -45.71 (c=0.62, $CHCl_3$); **^{1}H NMR (600 MHz, $CDCl_3$)** δ 7.81 – 7.73 (m, 3H), 7.63 (s, 1H), 7.43 (t, *J* = 6.6 Hz, 2H), 7.28 (d, *J* = 8.3

Hz, 1H), 4.17 (q, J = 7.1 Hz, 2H), 3.35 (d, J = 12.0 Hz, 1H), 2.92 (d, J = 12.0 Hz, 1H), 1.93 (m, 1H), 1.64 (s, 1H), 1.62 (d, J = 4.6 Hz, 1H), 1.44 (m, 2H), 1.26 (t, J = 7.1 Hz, 3H), 1.22 – 1.16 (m, 1H), 0.94 (t, J = 7.3 Hz, 3H); **^{13}C NMR (151 MHz, $CDCl_3$)** δ 176.63, 134.14, 133.40, 132.46, 128.80, 128.25, 127.86, 127.64, 127.60, 126.02, 125.60, 62.18, 61.00, 46.20, 42.90, 17.41, 14.39, 14.31; **HRMS(ESI)** m/z: $[M+H]^+$ Calculated for $C_{18}H_{24}NO_2^+$ 286.1802; found 286.1795.

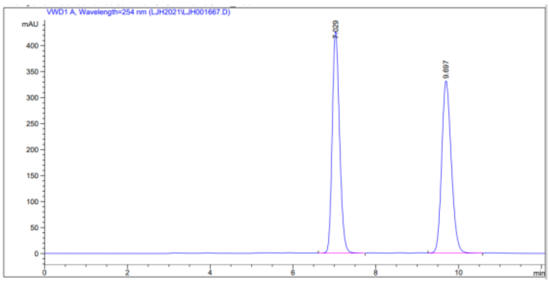

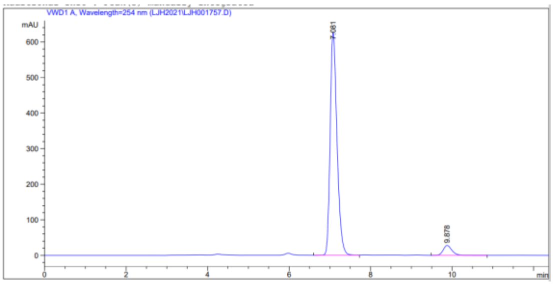

Peak #	RetTime [min]	Type	Width [min]	Area [mAU*s]	Height [mAU]	Area %
1	7.029	BV	0.1899	5248.99268	427.44888	49.6336
2	9.697	VB	0.2485	5326.49023	331.87207	50.3664

Peak #	RetTime [min]	Type	Width [min]	Area [mAU*s]	Height [mAU]	Area %
1	7.081	BV	0.1781	7310.96338	627.79810	94.7019
2	9.878	BB	0.2277	409.01404	27.69617	5.2981

Ethyl (S)-2-amino-2-(naphthalen-2-ylmethyl)hexanoate (5d):

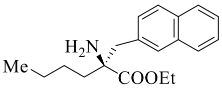

Colorless oil (52.5 mg, 88%); R_f = 0.26 (petroleum ether/ ethyl acetate = 5:1); the enantiomeric excess was determined to be 90% by HPLC analysis on Daicel Chirapak AD-H column (hexane/isopropanol = 90/10, flow rate 1.0 mL/min, T = 30 °C), UV 254 nm, t_R(major) 10.567 min, t_R(minor) 7.012 min; $[\alpha]_D^{25}$ = -37.79 (c=1.01, $CHCl_3$); **^{1}H NMR (600 MHz, $CDCl_3$)** δ 7.86 – 7.70 (m, 3H), 7.63 (s, 1H), 7.49 – 7.38 (m, 2H), 7.28 (d, J = 8.4 Hz, 1H), 4.17 (m, 2H), 3.35 (d, J = 13.2 Hz, 1H), 2.92 (d, J = 13.2 Hz, 1H), 1.95 (m, 1H), 1.71 – 1.56 (m, 3H), 1.45 – 1.29 (m, 3H), 1.32 – 1.22 (t, J = 7.2 Hz, 3H), 1.21 – 1.11 (m, 1H), 0.91 (t, J = 7.2 Hz, 3H); **^{13}C NMR (151 MHz, CDCl3)** δ 176.67, 134.14, 133.39, 132.46, 128.79, 128.22, 127.89, 127.64, 127.60, 126.03,125.61, 62.16, 61.02, 46.21, 40.37, 26.24, 22.98, 14.33, 13.98; HRMS(ESI) m/z: $[M+H]^+$ Calculated for $C_{19}H_{26}NO_2^+$ 300.1958; found 300.1956.

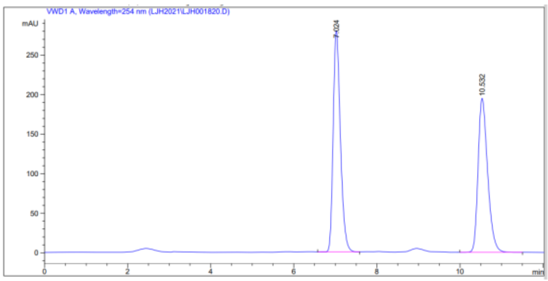

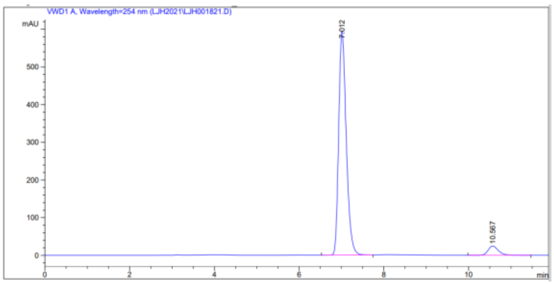

Peak #	RetTime [min]	Type	Width [min]	Area [mAU*s]	Height [mAU]	Area %
1	7.024	VB	0.1849	3388.36890	280.01654	52.1394
2	10.532	BB	0.2461	3110.30005	194.77750	47.8606

Peak #	RetTime [min]	Type	Width [min]	Area [mAU*s]	Height [mAU]	Area %
1	7.012	BB	0.1895	7355.65039	594.82190	94.7619
2	10.567	BB	0.2533	406.59805	24.50887	5.2381

Ethyl (S)-2-amino-2-(naphthalen-2-ylmethyl)heptanoate (5e):

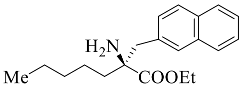

Colorless oil (57.5 mg, 92%); R_f = 0.25 (petroleum ether/ ethyl acetate = 5:1); the enantiomeric excess was determined to be 90% by HPLC analysis on Daicel Chirapak AD-H column (hexane/isopropanol = 90/10, flow rate 1.0 mL/min, T = 30 °C), UV 254 nm, t_R(major) 9.292 min, t_R(minor) 6.534 min; $[\alpha]_D^{25}$ = -35.50 (c=0.52, $CHCl_3$); **^{1}H NMR (600 MHz, $CDCl_3$)** δ 7.85 – 7.70 (m, 3H), 7.63 (s, 1H), 7.49 – 7.38 (m, 2H), 7.35 – 7.26 (m, 1H), 4.17 (m, 2H), 3.35 (d, *J* = 13.2 Hz, 1H), 2.92 (d, *J* = 13.2 Hz, 1H), 1.95 (m, 1H), 1.70 – 1.57 (m, 3H), 1.41 (m, 1H), 1.31 (m, 4H), 1.27 (t, *J* = 7.2 Hz, 3H), 1.22 – 1.11 (m, 1H), 0.89 (t, *J* = 6.6 Hz, 3H); **^{13}C NMR (151 MHz, $CDCl_3$)** δ 176.68, 134.14, 133.39, 132.45, 128.79, 128.22, 127.88, 127.64, 127.60, 126.02, 125.60, 62.19, 61.01, 46.22, 40.61, 32.05, 23.70, 22.47, 14.33, 13.98; **HRMS(ESI)** m/z: $[M+H]^+$ Calculated for $C_{20}H_{28}NO_2^+$ 314.2115; found 314.2110.

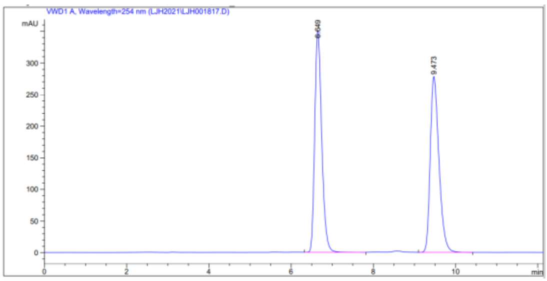

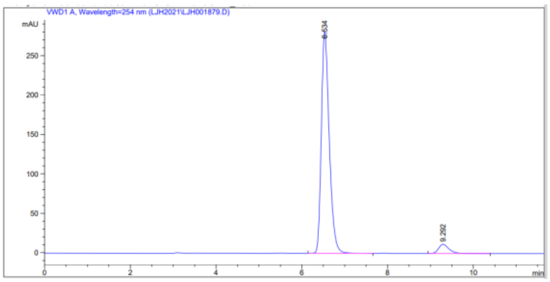

Peak #	RetTime [min]	Type	Width [min]	Area [mAU*s]	Height [mAU]	Area %
1	6.649	VB	0.1813	4135.35107	350.69736	50.2122
2	9.473	BB	0.2259	4100.39063	278.14197	49.7878

Peak #	RetTime [min]	Type	Width [min]	Area [mAU*s]	Height [mAU]	Area %
1	6.534	BB	0.1940	3591.88965	281.55273	94.9755
2	9.292	BB	0.2473	190.02206	11.63900	5.0245

Ethyl (S)-2-amino-2-(naphthalen-2-ylmethyl)octanoate (5f):

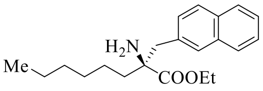

Colorless oil (55.5 mg, 85%); R_f = 0.28 (petroleum ether/ ethyl acetate = 5:1); the enantiomeric excess was determined to be 89% by HPLC analysis on Daicel Chirapak AD-H column (hexane/isopropanol = 90/10, flow rate 1.0 mL/min, T = 30 °C), UV 254 nm, t_R(major) 7.834 min, t_R(minor) 6.149 min; $[\alpha]_D^{25}$ = -35.57 (c=0.94, $CHCl_3$); **^{1}H NMR (600 MHz, $CDCl_3$)** δ 7.83 – 7.71 (m, 3H), 7.63 (s, 1H), 7.49 – 7.39 (m, 2H), 7.28 (m, 1H), 4.17 (m, 2H), 3.35 (d, *J* = 13.2 Hz, 1H), 2.92 (d, *J* =13.2 Hz, 1H), 1.95 (m, 1H), 1.71 – 1.55 (m, 3H), 1.41 (m, 1H), 1.27 (m, 9H), 1.22 – 1.11 (m, 1H), 0.88 (t, *J* = 6.0 Hz, 3H); **^{13}C NMR (151 MHz, $CDCl_3$)** δ 176.66, 134.15, 133.39, 132.46, 128.79, 128.23, 127.86, 127.63, 127.59, 126.01, 125.59, 62.19, 60.99, 46.21, 40.66, 31.64, 29.52, 23.99, 22.54, 14.32, 14.02; **HRMS(ESI)** m/z: $[M+H]^+$ Calculated for $C_{21}H_{30}NO_2^+$ 328.2271; found 328.2270.

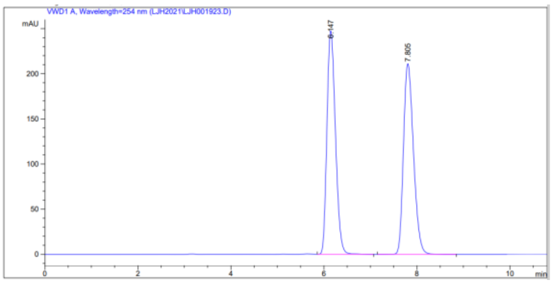

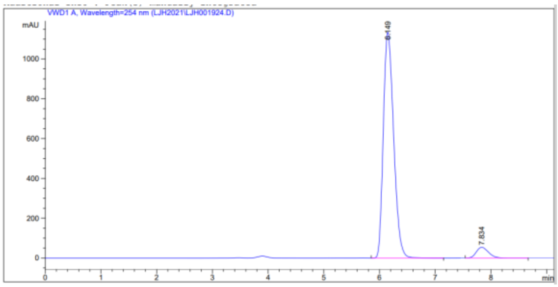

Peak #	RetTime [min]	Type	Width [min]	Area [mAU*s]	Height [mAU]	Area %
1	6.147	VB	0.1990	3137.88696	247.44989	49.9338
2	7.805	BB	0.2321	3146.20239	211.14696	50.0662

Peak #	RetTime [min]	Type	Width [min]	Area [mAU*s]	Height [mAU]	Area %
1	6.149	VB	0.2007	1.45528e4	1134.30627	94.7142
2	7.834	VB	0.2355	812.16486	53.92172	5.2858

Ethyl (R)-2-amino-2-cyclopropyl-3-(naphthalen-2-yl)propanoate (5h):

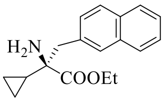

White solid (36.8 mg, 68%); m.p. = 79-81 ºC; R_f = 0.25 (petroleum ether/ ethyl acetate = 5:1); the enantiomeric excess was determined to be 85% by HPLC analysis on Daicel Chirapak AD-H column (hexane/isopropanol = 90/10, flow rate 1.0 mL/min, T = 30 °C), UV 254 nm, t_R(major) 9.216 min, t_R(minor) 7.560 min; $[\alpha]_D^{25}$ = -13.66 (c=0.45, $CHCl_3$); **^{1}H NMR (600 MHz, $CDCl_3$)** δ 7.83 – 7.73 (m, 3H), 7.63 (s, 1H), 7.48 – 7.40 (m, 2H), 7.29 (d, *J* = 8.4 Hz, 1H), 3.74 (s, 3H), 3.46 (d, *J* = 13.2 Hz, 1H), 3.01 (d, *J* = 13.2 Hz, 1H), 1.33 (m, 4H), 0.55 – 0.31 (m, 5H); **^{13}C NMR (151 MHz, CDCl3)** δ 176.46, 133.66, 132.92, 131.95, 128.32, 127.72, 127.41, 127.15, 127.10, 125.55, 125.13, 60.28, 51.58, 45.36, 19.04, 0.18; **HRMS(ESI)** m/z: $[M+H]^+$ Calculated for $C_{18}H_{22}NO_2^+$ 284.1645; found 284.1639.

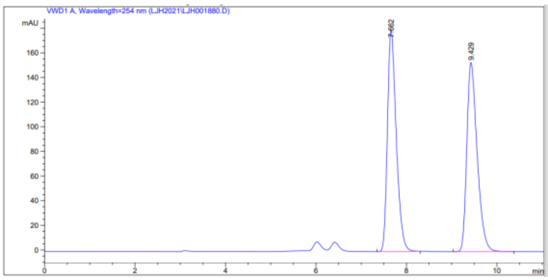

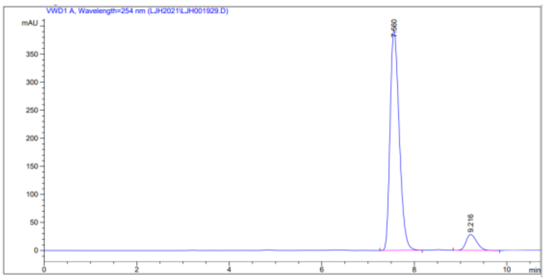

Peak #	RetTime [min]	Type	Width [min]	Area [mAU*s]	Height [mAU]	Area %
1	7.662	BB	0.2060	2432.79468	179.70012	49.9037
2	9.429	BB	0.2410	2442.18140	153.49788	50.0963

Peak #	RetTime [min]	Type	Width [min]	Area [mAU*s]	Height [mAU]	Area %
1	7.560	BB	0.2158	5400.72900	392.81384	92.2614
2	9.216	BB	0.2505	452.99405	28.35808	7.7386

Ethyl (S)-2-amino-4-methyl-2-(naphthalen-2-ylmethyl)pentanoate (5i):

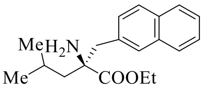

Colorless oil (57.7 mg, 94%); R_f = 0.26 (petroleum ether/ ethyl acetate = 5:1); the enantiomeric excess was determined to be 91% by HPLC analysis on Daicel Chirapak AD-H column (hexane/isopropanol = 90/10, flow rate 1.0 mL/min, T = 30 ºC), UV 254 nm, t_R(major) 9.727 min, t_R(minor) 6.864 min; $[\alpha]_D^{25}$ = -46.30 (c=1.00, $CHCl_3$); **^{1}H NMR (600 MHz, $CDCl_3$)** δ 7.86 – 7.70 (m, 3H), 7.62 (s, 1H), 7.50 – 7.37 (m, 2H), 7.26 (m, 1H), 4.21 – 4.09 (m, 2H), 3.33 (d, *J* = 13.2 Hz, 1H), 2.88 (d, *J* = 13.2 Hz, 1H), 1.97 (dd, *J* = 13.8, 7.8 Hz, 1H),

1.78 (m, 1H), 1.65 (m, 3H), 1.26 (t, J = 7.2 Hz, 3H), 0.98 (d, J = 6.6 Hz, 3H), 0.87 (d, J = 6.6 Hz, 3H); **^{13}C NMR (151 MHz, $CDCl_3$)** δ 177.07, 133.89, 133.39, 132.49, 128.87, 128.22, 127.87, 127.64, 127.60, 126.02, 125.62, 61.79, 60.98, 49.14, 47.61, 24.76, 24.46, 22.96, 14.21; **HRMS(ESI)** m/z: $[M+H]^+$ Calculated for $C_{19}H_{26}NO_2^+$ 300.1958; found 300.1952.

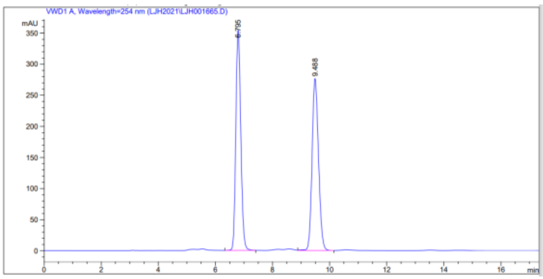

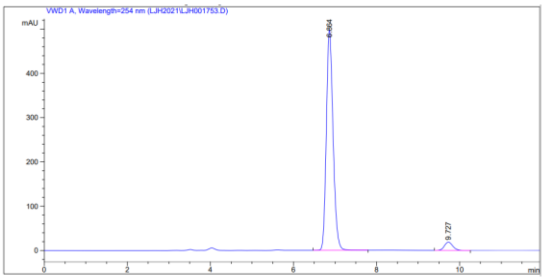

Peak #	RetTime [min]	Type	Width [min]	Area [mAU*s]	Height [mAU]	Area %
1	6.795	BB	0.1881	4288.47412	353.62714	49.7542
2	9.488	VB	0.2426	4330.83887	276.36078	50.2458

Peak #	RetTime [min]	Type	Width [min]	Area [mAU*s]	Height [mAU]	Area %
1	6.864	BB	0.1715	5569.50684	497.08478	95.5170
2	9.727	BB	0.2138	261.40204	18.90482	4.4830

Ethyl (S)-2-amino-2-benzyl-3-(naphthalen-2-yl)propanoate (5j):

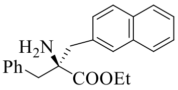

A pale yellow soild (60.0 mg, 90%); m.p. = 70-72 ºC; R_f = 0.24 (petroleum ether/ ethyl acetate = 5:1); the enantiomeric excess was determined to be 87% by HPLC analysis on Daicel Chirapak AD-H column (hexane/isopropanol = 90/10, flow rate 1.0 mL/min, T = 30 ºC), UV 254 nm, t_R(major) 10.319 min, t_R(minor) 6.945 min; $[\alpha]_D^{25}$ = -26.08 (c=1.28, $CHCl_3$); **^{1}H NMR (400 MHz, $CDCl_3$)** δ 7.90 – 7.71 (m, 3H), 7.68 (s, 1H), 7.52 – 7.40 (m, 2H), 7.35 – 7.16 (m, 6H), 4.28 – 4.01 (m, 2H), 3.53 (d, J = 12.8.1 Hz, 1H), 3.41 (d, J = 12.8 Hz, 1H), 3.01 (d, J = 12.8 Hz, 1H), 2.89 (d, J = 12.8 Hz, 1H), 1.57 (s, 2H), 1.20 (t, J = 6.8 Hz, 3H); **^{13}C NMR (101 MHz, $CDCl_3$)** δ 175.87, 136.28, 133.89, 133.43, 132.53, 130.06, 128.90, 128.44, 128.18, 127.99, 127.67, 127.62, 127.07, 126.08, 125.69, 63.23, 61.10, 46.57, 46.55, 14.23; **HRMS(ESI)** m/z: $[M+H]^+$ Calculated for $C_{22}H_{24}NO_2^+$ 334.1802; found 334.1805.

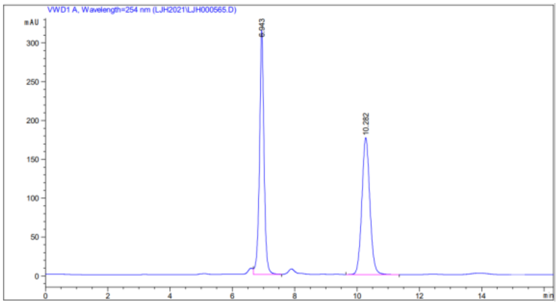

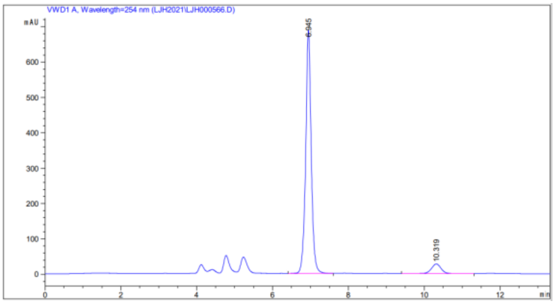

Peak #	RetTime [min]	Type	Width [min]	Area [mAU*s]	Height [mAU]	Area %
1	6.943	VV	0.1482	3166.29639	314.27573	50.1140
2	10.282	BB	0.2742	3151.89014	176.31761	49.8860

Peak #	RetTime [min]	Type	Width [min]	Area [mAU*s]	Height [mAU]	Area %
1	6.945	VB	0.1489	6975.94629	688.04016	93.3029
2	10.319	BB	0.2782	500.72244	27.48233	6.6971

Ethyl (S)-2-amino-2-(naphthalen-2-ylmethyl)-4-phenylbutanoate (5k):

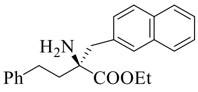

Colorless oil (61.7mg, 89%); R_f = 0.23 (petroleum ether/ ethyl acetate = 5:1); the enantiomeric excess was determined to be 90% by HPLC analysis on Daicel Chirapak AD-H column (hexane/isopropanol = 90/10, flow rate 1.0 mL/min, T = 30 °C), UV 254 nm, t_R(major) 17.924 min, t_R(minor) 10.401 min; $[\alpha]_D^{25}$ = -36.02 (c=1.09, $CHCl_3$); **^{1}H NMR (600 MHz, $CDCl_3$)** δ 7.87 – 7.71 (m, 3H), 7.64 (s, 1H), 7.51 – 7.39 (m, 2H), 7.35 – 7.25 (m, 3H), 7.18 (m, 3H), 4.23 – 4.13 (m, 2H), 3.37 (d, *J* = 13.2 Hz, 1H), 2.98 (d, *J* = 13.2 Hz, 1H), 2.76 (m, 1H), 2.52 (m, 1H), 2.27 (m, 1H), 1.98 (m, 1H), 1.89 (s, 2H), 1.28 (t, *J* = 7.2 Hz, 3H); **^{13}C NMR (151 MHz, $CDCl_3$)** δ 176.20, 141.58, 133.79, 133.42, 132.53, 128.90, 128.52, 128.45, 128.21, 128.01, 127.68, 127.65, 126.12, 126.06, 125.72, 62.24, 61.23, 46.26, 42.39, 30.71, 14.39; **HRMS(ESI)** m/z: $[M+H]^+$ Calculated for $C_{23}H_{26}NO_2^+$ 348.1958; found 348.1954.

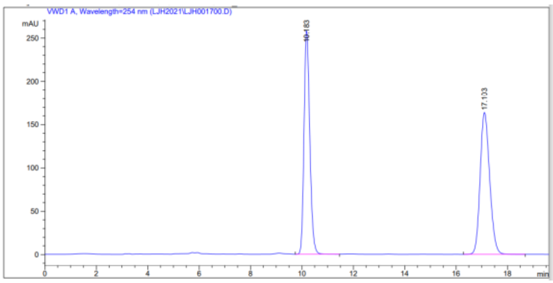

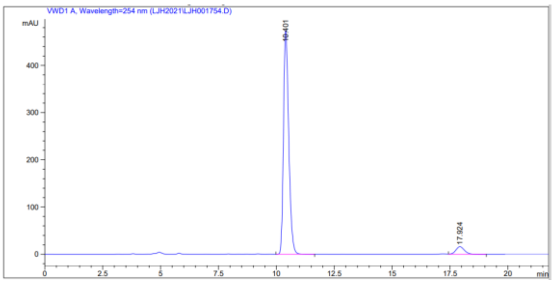

Peak #	RetTime [min]	Type	Width [min]	Area [mAU*s]	Height [mAU]	Area %
1	10.183	BB	0.2411	4008.17725	257.89270	48.7559
2	17.103	BB	0.3992	4212.73438	163.77713	51.2441

Peak #	RetTime [min]	Type	Width [min]	Area [mAU*s]	Height [mAU]	Area %
1	10.401	BB	0.2422	7472.77051	474.09418	94.7580
2	17.924	VB	0.3952	413.39429	16.05157	5.2420

Ethyl (S)-2-amino-2-(naphthalen-2-ylmethyl)pent-4-enoate (5l):

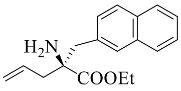

Colorless oil (51.1mg, 90%); R_f = 0.26 (petroleum ether/ ethyl acetate = 5:1); the enantiomeric excess was determined to be 90% by HPLC analysis on Daicel Chirapak AD-H column (hexane/isopropanol = 90/10, flow rate 1.0 mL/min, T = 30 °C), UV 254 nm, t_R(major) 9.731 min, t_R(minor) 6.916 min; $[\alpha]_D^{25}$ = -10.20 (c=0.92, $CHCl_3$); **^{1}H NMR (600 MHz, $CDCl_3$)** δ 7.85 – 7.71 (m, 3H), 7.64 (s, 1H), 7.50 – 7.39 (m, 2H), 7.28 (d, *J* = 7.8 Hz, 1H), 5.74 (m, 1H), 5.18 (m, 2H), 4.17 (m, 2H), 3.35 (d, *J* = 13.2 Hz, 1H), 2.95 (d, *J* = 13.2 Hz, 1H), 2.77 (dd, *J* = 13.8, 6.6 Hz, 1H), 2.36 (dd, *J* = 13.2, 8.4 Hz, 1H), 1.66 (s, 2H), 1.25 (t, *J* = 7.2 Hz, 3H); **^{13}C NMR (151 MHz, $CDCl_3$)** δ 176.09, 133.91, 133.39, 132.58, 132.50, 128.83, 128.19, 127.91, 127.65, 127.61, 126.05, 125.65, 119.70, 61.78, 61.14, 45.98, 44.69, 14.32; **HRMS(ESI)** m/z: $[M+H]^+$ Calculated for $C_{18}H_{22}NO_2^+$ 284.1645; found 284.1640.

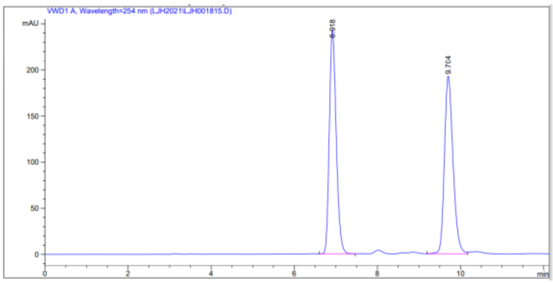

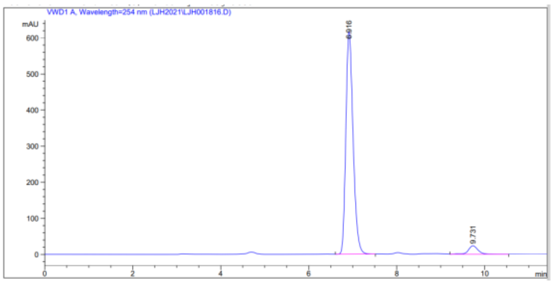

Peak #	RetTime [min]	Type	Width [min]	Area [mAU*s]	Height [mAU]	Area %
1	6.918	BB	0.1780	2792.59961	242.59850	50.2830
2	9.704	BV	0.2196	2761.16479	192.78438	49.7170

Peak #	RetTime [min]	Type	Width [min]	Area [mAU*s]	Height [mAU]	Area %
1	6.916	BB	0.1751	7030.06787	617.31146	95.0424
2	9.731	VB	0.2329	366.69995	23.70666	4.9576

Methyl (S)-2-amino-3-((tert-butoxycarbonyl)amino)-2-(naphthalen-2-ylmethyl)propanoate (5m):

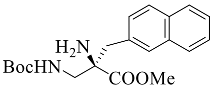

White solid (47.2 mg, 66%); m.p. = 87-89 °C; R_f = 0.24 (petroleum ether/ ethyl acetate = 1:1); the enantiomeric excess was determined to be 72% by HPLC analysis on Daicel Chirapak AD-H column (hexane/isopropanol = 95/5, flow rate 1.0 mL/min, T = 30 °C), UV 254 nm, t_R(major) 15.245 min, t_R(minor) 10.720 min; $[\alpha]_D^{25}$ = -25.03 (c=0.50, $CHCl_3$); **^{1}H NMR (600 MHz, $CDCl_3$)** δ 7.86 – 7.71 (m, 3H), 7.61 (s, 1H), 7.50 – 7.40 (m, 2H), 7.24 (d, *J* = 8.4 Hz, 1H), 5.06 (s, 1H), 3.73 (s, 3H), 3.58 (m, 1H), 3.38 (m, 1H), 3.31 (d, *J* = 13.8 Hz, 1H), 2.95 (d, *J* = 13.8 Hz, 1H), 1.72 (s, 2H), 1.44 (s, 9H); **^{13}C NMR (151 MHz, $CDCl_3$)** δ 175.45, 156.06, 133.40, 133.05, 132.53, 131.91, 128.83, 128.14, 127.94, 127.64, 127.62, 126.17, 125.79, 79.57, 62.63, 52.34, 48.48, 43.40, 28.35; **HRMS(ESI)** m/z: $[M+H]^+$ Calculated for $C_{20}H_{27}N_2O_4^+$ 359.1965; found 359.1964.

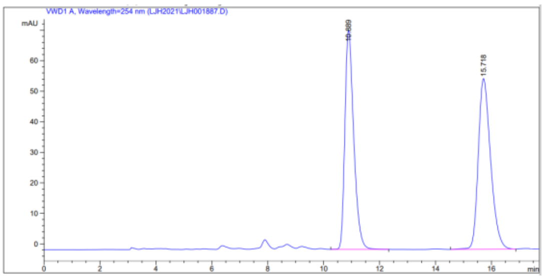

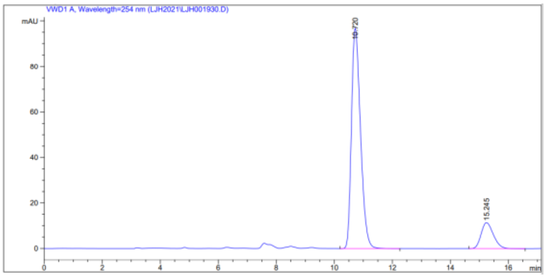

Peak #	RetTime [min]	Type	Width [min]	Area [mAU*s]	Height [mAU]	Area %
1	10.889	BB	0.3345	1571.73779	71.69930	47.9309
2	15.718	BB	0.4672	1707.43652	55.88614	52.0691

Peak #	RetTime [min]	Type	Width [min]	Area [mAU*s]	Height [mAU]	Area %
1	10.720	BB	0.3345	2097.67627	96.78536	86.1840
2	15.245	BB	0.4521	336.27594	11.44672	13.8160

Ethyl (S)-2-amino-4-(methylthio)-2-(naphthalen-2-ylmethyl)butanoate (5n):

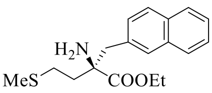

Colorless oil (54.1 mg, 85%); R_f = 0.28 (petroleum ether/ ethyl acetate = 5:1); the enantiomeric excess was determined to be 89% by HPLC analysis on Daicel Chirapak AD-H column (hexane/isopropanol = 90/10, flow rate 1.0 mL/min, T = 30 °C), UV 254 nm, t_R(major) 18.901 min, t_R(minor) 11.123 min; $[\alpha]_D^{25}$ = -34.76 (c=1.00, $CHCl_3$); **^{1}H**

NMR (600 MHz, $CDCl_3$) δ 7.81 – 7.72 (m, 3H), 7.62 (s, 1H), 7.48 – 7.40 (m, 2H), 7.29 – 7.23 (m, 1H), 4.18 (q, *J* = 7.2 Hz, 2H), 3.33 (d, *J* = 13.2 Hz, 1H), 2.94 (d, *J* = 13.2 Hz, 1H), 2.64 – 2.57 (m, 1H), 2.48 – 2.41 (m, 1H), 2.26 (m, 1H), 2.11 (s, 3H), 1.94 (m, 1H), 1.65 (s, 2H), 1.26 (t, *J* = 7.2 Hz, 3H); **^{13}C NMR (151 MHz, $CDCl_3$)** δ 175.89, 133.51, 133.37, 132.51, 128.88, 128.13, 128.00, 127.64, 127.63, 126.14, 125.74, 61.98, 61.30, 46.31, 39.84, 28.97, 15.62, 14.28; **HRMS(ESI)** m/z: $[M+H]^+$ Calculated for $C_{18}H_{24}NO_2S^+$ 318.1522; found 318.1517.

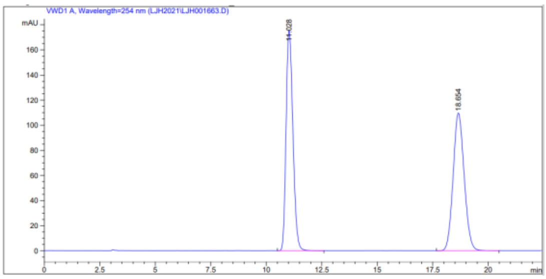

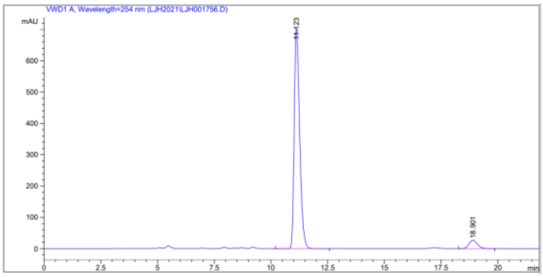

Peak #	RetTime [min]	Type	Width [min]	Area [mAU*s]	Height [mAU]	Area %
1	11.028	BB	0.3354	3758.54443	175.80751	49.8005
2	18.654	BB	0.5300	3788.65430	109.61792	50.1995

Peak #	RetTime [min]	Type	Width [min]	Area [mAU*s]	Height [mAU]	Area %
1	11.123	BB	0.2564	1.17601e4	703.03497	94.3751
2	18.901	BB	0.4020	700.92242	26.86775	5.6249

Methyl (R)-2-amino-6-(((benzyloxy)carbonyl)amino)-2-(naphthalen-2-ylmethyl)hexanoate (5o):

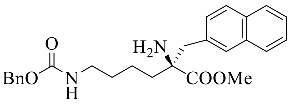

Colorless oil (68.8 mg, 79%); R_f = 0.26 (petroleum ether/ethyl acetate = 1:1); the enantiomeric excess was determined to be 81% by HPLC analysis on Daicel Chirapak AD-H column (hexane/isopropanol = 70/30, flow rate 1.0 mL/min, T = 30 °C), UV 254 nm, t_R(major) 15.741 min, t_R(minor) 11.532 min; $[\alpha]_D^{25}$ = -18.35 (c=0.87, $CHCl_3$); **^1H NMR (600 MHz, $CDCl_3$)** δ 7.81 – 7.72 (m, 3H), 7.60 (s, 1H), 7.48 – 7.40 (m, 2H), 7.34 (m, 4H), 7.30 (m, 1H), 7.23 (d, *J* = 8.4 Hz, 1H), 5.09 (s, 2H), 4.87 (s, 1H), 3.69 (s, 3H), 3.32 (d, *J* = 13.2 Hz, 1H), 3.20 – 3.12 (m, 2H), 2.91 (d, *J* = 13.2 Hz, 1H), 1.94 (m, 1H), 1.71 – 1.59 (m, 3H), 1.47 (m, 3H), 1.27 – 1.13 (m, 1H); **^{13}C NMR (151 MHz, $CDCl_3$)** δ 176.87, 156.42, 136.68, 133.81, 133.40, 132.49, 128.74, 128.53, 128.09, 128.03, 128.01, 127.66, 127.62, 126.12, 125.71, 66.62, 62.33, 52.07, 46.35, 40.78, 40.00, 30.10, 21.31; **HRMS(ESI)** m/z: $[M+H]^+$ Calculated for $C_{26}H_{31}N_2O_4^+$ 435.2278; found 435.2280.

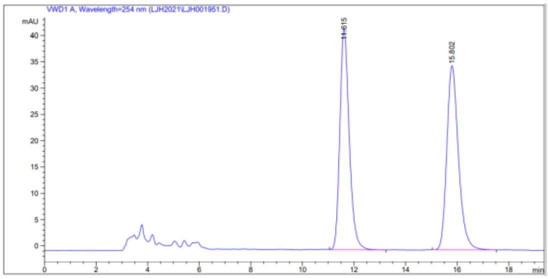

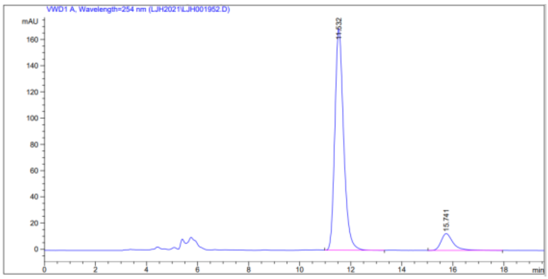

Peak #	RetTime [min]	Type	Width [min]	Area [mAU*s]	Height [mAU]	Area %
1	11.615	BB	0.3864	1060.56592	42.01219	49.1775
2	15.802	BB	0.4806	1096.04138	34.98800	50.8225

Peak #	RetTime [min]	Type	Width [min]	Area [mAU*s]	Height [mAU]	Area %
1	11.532	BB	0.3651	4023.29761	169.05864	90.4842
2	15.741	BB	0.4953	423.10904	12.83663	9.5158

Ethyl (R)-2-amino-3-(naphthalen-2-yl)-2-phenylpropanoate (5p):

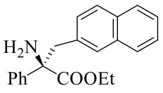

A pale yellow solid (54.1 mg, 85%); m.p. = 73-75 °C; R_f = 0.27 (petroleum ether/ ethyl acetate = 5:1); the enantiomeric excess was determined to be 78% by HPLC analysis on Daicel Chirapak AD-H column (hexane/isopropanol = 90/10, flow rate 1.0 mL/min, T = 30 °C), UV 254 nm, t_R(major) 13.259 min, t_R(minor) 10.726 min; $[\alpha]_D^{25}$ = +34.18 (c=1.06, $CHCl_3$); **^{1}H NMR (400 MHz, $CDCl_3$)** δ 7.81 – 7.67 (m, 3H), 7.65 – 7.57 (m, 3H), 7.46 – 7.39 (m, 2H), 7.39 – 7.27 (m, 2H), 7.22 (m, 2H), 4.19 (q, *J* = 7.2 Hz, 2H), 3.79 (d, *J* = 13.2 Hz, 1H), 3.30 (d, *J* = 13.2 Hz, 1H), 1.91 (s, 2H), 1.24 (t, *J* = 7.2 Hz, 3H); **^{13}C NMR (101 MHz, $CDCl_3$)** δ 175.04, 143.17, 133.91, 133.35, 132.50, 129.47, 128.67, 128.42, 127.73, 127.60, 127.59, 126.00, 125.70, 125.63, 64.54, 61.57, 46.26, 14.14; **HRMS(ESI)** m/z: $[M+H]^+$ Calculated. for $C_{21}H_{22}NO_2^+$ 320.1645; found 320.1643.

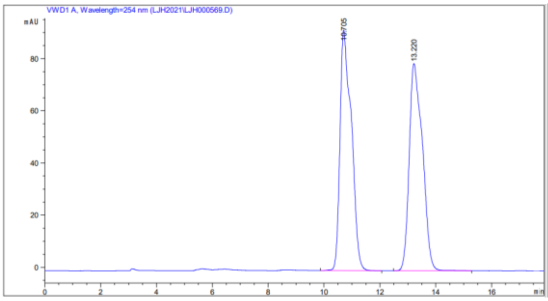

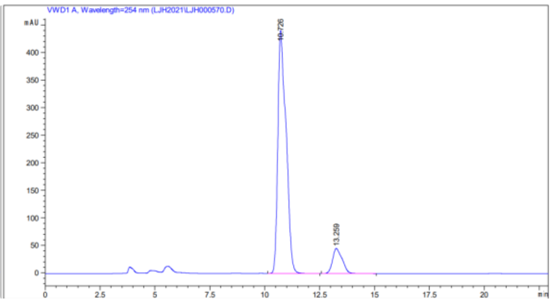

Peak #	RetTime [min]	Type	Width [min]	Area [mAU*s]	Height [mAU]	Area %
1	10.705	BB	0.3881	2605.56958	92.16690	49.9180
2	13.220	BB	0.4597	2614.13062	79.43458	50.0820

Peak #	RetTime [min]	Type	Width [min]	Area [mAU*s]	Height [mAU]	Area %
1	10.726	BB	0.3615	1.14588e4	440.92923	89.2063
2	13.259	BB	0.4306	1386.48560	45.59238	10.7937

Ethyl (S)-2-(naphthalen-2-ylmethyl)-5-oxopyrrolidine-2-carboxylate (5q):

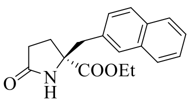

Colorless oil (47.2 mg, 67%); R_f = 0.23 (petroleum ether/ethyl acetate = 1:1); the enantiomeric excess was determined to be 89% by HPLC analysis on Daicel Chirapak AD-H column (hexane/isopropanol = 85/15, flow rate 1.0 mL/min, T = 30 °C), UV 254 nm, t_R(major) 17.493 min, t_R(minor) 7.860 min; $[\alpha]_D^{25}$ = +12.40 (c=0.55, $CHCl_3$); **^{1}H NMR (600 MHz, $CDCl_3$)** δ 7.84 – 7.73 (m, 3H), 7.61 (s, 1H), 7.50 – 7.43 (m, 2H), 7.29 – 7.23 (m, 1H), 6.34 (s, 1H), 4.14 (q, *J* = 6.6 Hz, 2H), 3.41 (d, *J* = 13.8 Hz, 1H), 3.07 (d, *J* = 13.8 Hz, 1H), 2.54 – 2.45 (m, 1H), 2.36 – 2.24 (m, 2H), 2.19 (m, 1H), 1.18 (t, *J* = 7.2 Hz, 3H); **^{13}C NMR (151 MHz, $CDCl_3$)** δ 176.62, 173.17, 133.37, 132.59, 132.44, 128.76, 128.37, 127.67, 127.65, 126.36,

126.03, 66.47, 61.86, 45.23, 30.96, 29.70, 14.08; **HRMS(ESI)** m/z: $[M+H]^+$ Calculated for $C_{18}H_{20}NO_3^+$ 298.1438; found 298.1435.

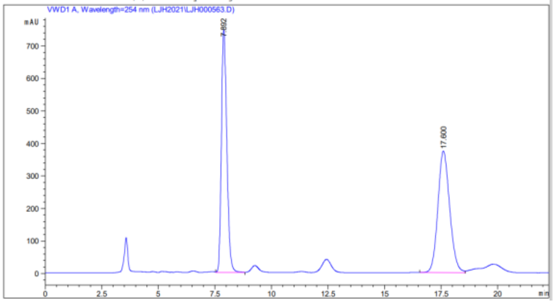

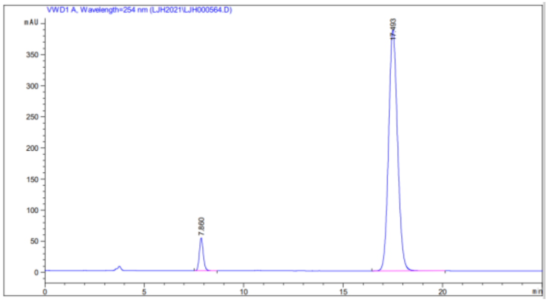

Peak #	RetTime [min]	Type	Width [min]	Area [mAU*s]	Height [mAU]	Area %
1	7.892	VV	0.2592	1.24623e4	745.46539	48.0079
2	17.600	BV	0.5560	1.34965e4	373.18155	51.9921

Peak #	RetTime [min]	Type	Width [min]	Area [mAU*s]	Height [mAU]	Area %
1	7.860	BB	0.2117	726.97491	52.76842	5.6681
2	17.493	BB	0.4841	1.20988e4	387.14594	94.3319

Ethyl (S)-2-amino-3-(6-methoxynaphthalen-2-yl)-2-methylpropanoate (6a):

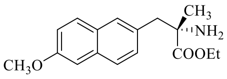

White solid (54.5 mg, 95%); m.p. = 67-69 °C; R_f = 0.23 (petroleum ether/ethyl acetate = 2:1); the enantiomeric excess was determined to be 91% by HPLC analysis on Daicel Chirapak AD-H column (hexane/isopropanol = 95/5, flow rate 1.0 mL/min, T = 30 °C), UV 254 nm, t_R(major) 26.252 min, t_R(minor) 16.971 min; $[\alpha]_D^{25}$ = -35.81 (c=0.97, $CHCl_3$); **^{1}H NMR (600 MHz, $CDCl_3$)** δ 7.65 (t, *J* = 9.6 Hz, 2H), 7.56 (s, 1H), 7.25 (d, *J* = 7.8 Hz, 1H), 7.12 (m, 1H), 7.09 (s, 1H), 4.16 (q, *J* = 6.6 Hz, 2H), 3.89 (s, 3H), 3.26 (d, *J* = 13.2 Hz, 1H), 2.92 (d, *J* = 13.2 Hz, 1H), 1.75 (s, 2H), 1.42 (s, 3H), 1.25 (t, *J* = 7.2 Hz, 3H); **^{13}C NMR (151 MHz, $CDCl_3$)** δ 177.08, 157.52, 133.54, 131.82, 129.12, 128.87, 128.78, 128.60, 126.67, 118.87, 105.59, 61.09, 58.84, 55.29, 46.77, 26.76, 14.23; **HRMS(ESI)** m/z: $[M+Na]^+$ Calculated for $C_{17}H_{21}NO_3Na^+$ 310.1414; found 310.1402.

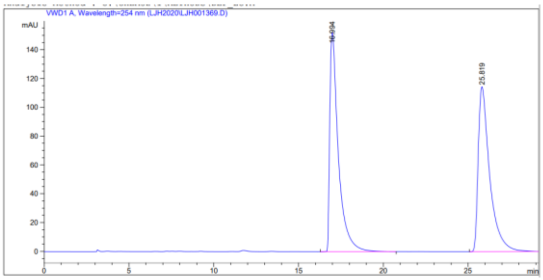

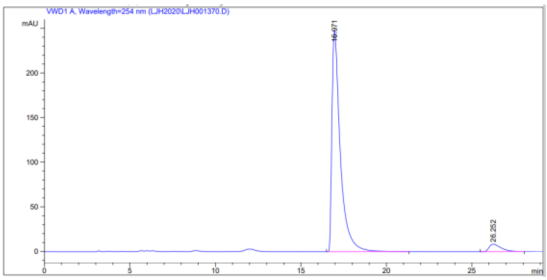

Peak #	RetTime [min]	Type	Width [min]	Area [mAU*s]	Height [mAU]	Area %
1	16.994	BB	0.5179	5377.58643	152.45140	49.9971
2	25.819	BBA	0.6956	5378.21289	114.42953	50.0029

Peak #	RetTime [min]	Type	Width [min]	Area [mAU*s]	Height [mAU]	Area %
1	16.971	BB	0.4997	8412.58105	247.69865	95.4795
2	26.252	BB	0.7227	398.29123	8.28495	4.5205

Ethyl (S)-2-amino-3-(6-(dimethylamino)naphthalen-2-yl)-2-methylpropanoate (6b):

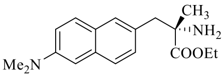

A pale yellow solid (55.1 mg, 90%); m.p. = 60-62 °C; R_f = 0.22 (petroleum ether/ethyl acetate = 2:1); the enantiomeric excess was

determined to be 89% by HPLC analysis on Daicel Chirapak AD-H column (hexane/isopropanol = 90/10, flow rate 1.0 mL/min, T = 30 °C), UV 254 nm, t_R(major) 22.530 min, t_R(minor) 13.106 min; $[\alpha]_D^{25}$ = -35.80 (c= 1.02, $CHCl_3$); **^{1}H NMR (600 MHz, $CDCl_3$)** δ 7.63 (d, *J* = 9.0 Hz, 1H), 7.56 (d, *J* = 8.4 Hz, 1H), 7.48 (s, 1H), 7.20 – 7.11 (m, 2H), 6.88 (s, 1H), 4.15 (q, *J* = 7.2 Hz, 2H), 3.24 (d, *J* = 13.2 Hz, 1H), 3.01 (s, 6H), 2.89 (d, *J* = 13.2 Hz, 1H), 1.90 (s, 2H), 1.42 (s, 3H), 1.25 (t, *J* = 7.2 Hz, 3H); **^{13}C NMR (151 MHz, $CDCl_3$)** δ 177.11, 148.59, 133.96, 130.07, 128.60, 128.44, 128.41, 126.77, 126.18, 116.71, 106.36, 61.08, 58.89, 46.77, 40.93, 26.73, 14.24; **HRMS(ESI)** m/z: $[M+H]^+$ Calculated for $C_{18}H_{25}N_2O_2^+$ 301.1911; found 301.1904.

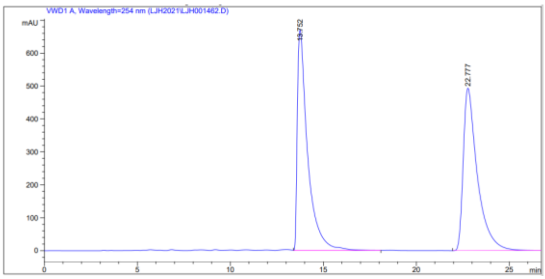

Peak #	RetTime [min]	Type	Width [min]	Area [mAU*s]	Height [mAU]	Area %
1	13.752	VB	0.5570	2.57690e4	669.40320	49.8416
2	22.777	BBA	0.7667	2.59328e4	493.01889	50.1584

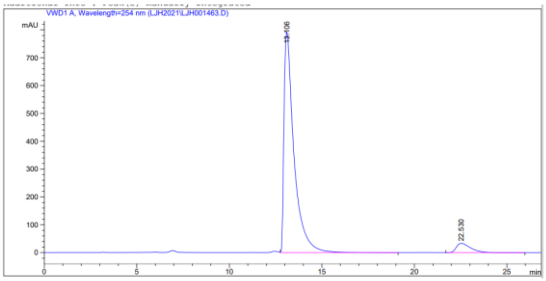

Peak #	RetTime [min]	Type	Width [min]	Area [mAU*s]	Height [mAU]	Area %
1	13.106	VB	0.5475	2.97357e4	791.75543	94.2528
2	22.530	BB	0.8205	1813.17908	33.37062	5.7472

Ethyl (S)-2-amino-3-(6-fluoronaphthalen-2-yl)-2-methylpropanoate (6c):

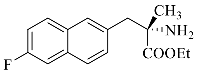

Colorless oil (49.2 mg, 89%); R_f = 0.23 (petroleum ether/ethyl acetate = 2:1); the enantiomeric excess was determined to be 91% by HPLC analysis on Daicel Chirapak AD-H column (hexane/isopropanol = 90/10, flow rate 1.0 mL/min, T = 30 °C), UV 254 nm, t_R(major) 9.879 min, t_R(minor) 7.922 min; $[\alpha]_D^{25}$ = -26.14 (c=1.00, $CHCl_3$); **^{1}H NMR (600 MHz, $CDCl_3$)** δ 7.75 (m, 1H), 7.69 (d, *J* = 8.4 Hz, 1H), 7.64 (s, 1H), 7.41 (m, 1H), 7.32 (d, *J* = 8.4 Hz, 1H), 7.23 (m, 1H), 4.16 (q, *J* = 7.8 Hz, 2H), 3.29 (d, *J* = 13.2.0 Hz, 1H), 2.97 (d, *J* = 13.2 Hz, 1H), 2.01 (s, 2H), 1.45 (s, 3H), 1.25 (t, *J* = 7.2 Hz, 3H); **^{13}C NMR (151 MHz, $CDCl_3$)** δ 176.79, 161.32, 159.69, 133.41, 133.12, 133.05, 130.35, 130.02, 129.96, 129.32, 128.78, 127.19, 127.15, 116.50, 116.33, 110.68, 110.55, 61.20, 58.88, 46.63, 26.61, 14.21; **HRMS(ESI)** m/z: $[M+H]^+$ Calculated for $C_{16}H_{19}FNO_2^+$ 276.1394; found 276.1387.

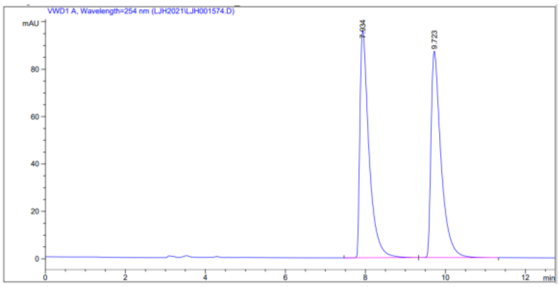

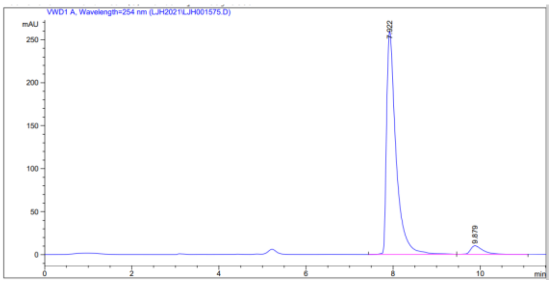

Peak #	RetTime [min]	Type	Width [min]	Area [mAU*s]	Height [mAU]	Area %
1	7.934	BB	0.2342	1515.76721	95.78631	50.0528
2	9.723	BB	0.2566	1512.56763	87.08632	49.9472

Peak #	RetTime [min]	Type	Width [min]	Area [mAU*s]	Height [mAU]	Area %
1	7.922	BB	0.2229	3882.76978	259.19791	95.3881
2	9.879	BB	0.2921	187.72539	9.73391	4.6119

Ethyl (S)-2-amino-2-methyl-3-(naphthalen-1-yl)propanoate (6d):

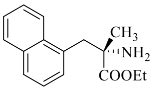

A pale yellow oil (43.5 mg, 85%); R_f = 0.25 (petroleum ether/ethyl acetate = 2:1); the enantiomeric excess was determined to be 81% by HPLC analysis on Daicel Chirapak AD-H column (hexane/isopropanol = 95/5, flow rate 1.0 mL/min, T = 30 °C), UV 254 nm, t_R(major) 11.406min, t_R(minor) 9.014 min; $[\alpha]_D^{25}$ = -5.83 (c=0.72, $CHCl_3$); **^{1}H NMR (600 MHz, $CDCl_3$)** δ 8.15 (d, *J* = 8.4 Hz, 1H), 7.83 (d, *J* = 7.8 Hz, 1H), 7.75 (d, *J* = 8.4 Hz, 1H), 7.47 (m, 2H), 7.40 (t, *J* = 7.2 Hz, 1H), 7.35 (d, *J* = 6.6 Hz, 1H), 4.09 – 3.97 (q, *J* = 7.2 Hz, 2H), 3.54 (d, *J* = 13.8 Hz, 1H), 3.41 (d, *J* = 13.8 Hz, 1H), 1.71 (s, 2H), 1.48 (s, 3H), 1.13 (t, *J* = 6.6 Hz, 3H); **^{13}C NMR (151 MHz, $CDCl_3$)** δ 177.04, 133.95, 133.12, 132.99, 128.69, 128.35, 127.67, 125.77, 125.50, 125.16, 124.51, 61.14, 59.37, 42.54, 26.97, 13.97; **HRMS(ESI)** m/z: $[M+H]^+$ Calculated for $C_{16}H_{20}NO_2^+$ 258.1489; found 258.1486.

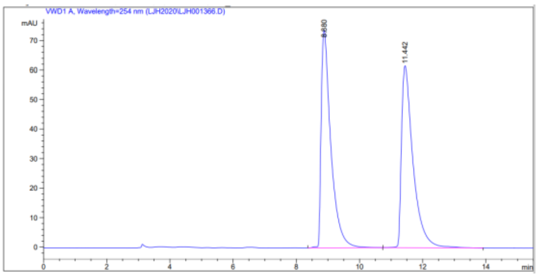

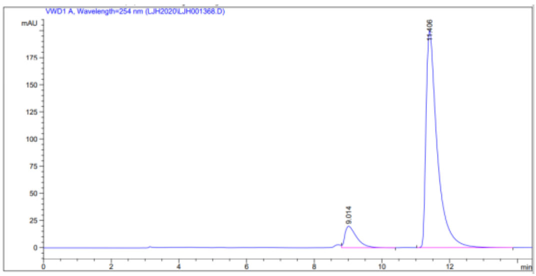

Peak #	RetTime [min]	Type	Width [min]	Area [mAU*s]	Height [mAU]	Area %
1	8.880	BB	0.3141	1561.53320	73.73036	50.0309
2	11.442	BB	0.3755	1559.60205	61.60803	49.9691

Peak #	RetTime [min]	Type	Width [min]	Area [mAU*s]	Height [mAU]	Area %
1	9.014	VB	0.3643	478.05698	19.83072	9.3998
2	11.406	BB	0.3397	4607.77881	200.40436	90.6002

Ethyl (S)-2-amino-2-methyl-3-(4-methylnaphthalen-1-yl)propanoate (6e):

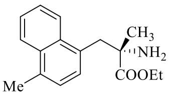

Colorless oil (50.9 mg, 93%); R_f = 0.25 (petroleum ether/ethyl acetate = 2:1); the enantiomeric excess was determined to be 83% by HPLC analysis on Daicel Chirapak IC-H column (hexane/isopropanol = 95/5, flow rate 1.0 mL/min, T = 30 °C), UV 254 nm, t_R(major) 9.491 min, t_R(minor) 8.489 min; $[\alpha]_D^{25}$ = -9.75 (c=0.59, $CHCl_3$); **^{1}H NMR (600 MHz,**

CDCl₃) δ 8.20 – 8.13 (m, 1H), 8.00 (m, 1H), 7.54 – 7.42 (m, 2H), 7.24 (s, 2H), 4.09 – 3.94 (q, *J* = 7.2 Hz, 2H), 3.51 (d, *J* = 13.8 Hz, 1H), 3.37 (d, *J* = 13.8 Hz, 1H), 2.66 (s, 3H), 1.64 (s, 2H), 1.46 (s, 3H), 1.15 (t, *J* = 7.2 Hz, 3H); **^{13}C NMR (151 MHz, $CDCl_3$)** δ 177.17, 133.63, 133.01, 131.24, 128.06, 126.05, 125.35, 125.32, 125.07, 124.71, 61.10, 59.35, 42.49, 27.01, 19.51, 14.00; **HRMS(ESI)** m/z: $[M+H]^+$ Calculated for $C_{17}H_{22}NO_2^+$ 272.1645; found 272.1641.

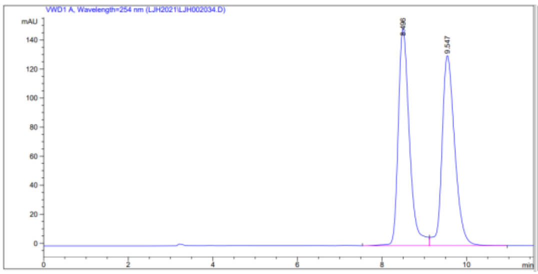

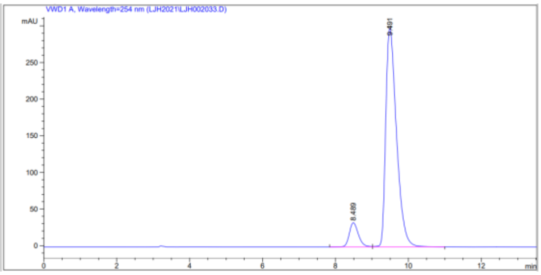

Peak #	RetTime [min]	Type	Width [min]	Area [mAU*s]	Height [mAU]	Area %
1	8.496	BV	0.2806	2738.31226	149.60304	50.0208
2	9.547	VB	0.3210	2736.03027	130.93661	49.9792

Peak #	RetTime [min]	Type	Width [min]	Area [mAU*s]	Height [mAU]	Area %
1	8.489	BV	0.2658	569.29956	32.93406	8.5271
2	9.491	VB	0.3145	6107.01758	298.53424	91.4729

Ethyl (S)-2-amino-3-(4-ethylnaphthalen-1-yl)-2-methylpropanoate (6f):

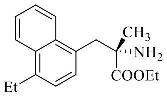

Colorless oil (52.4 mg, 94%); R_f= 0.26 (petroleum ether/ethyl acetate = 2:1); the enantiomeric excess was determined to be 83% by HPLC analysis on Daicel Chirapak AD-H column (hexane/isopropanol = 85/15, flow rate 1.0 mL/min, T = 30 ºC), UV 254 nm, t_R(major) 12.160 min, t_R(minor) 9.510 min; $[\alpha]_D^{25}$ = -14.77 (c=0.86, $CHCl_3$); **^{1}H NMR (600 MHz, $CDCl_3$)** δ 8.18 (m, 1H), 8.06 (m, 1H), 7.54 – 7.41 (m, 2H), 7.27 (m, 2H), 4.09 – 3.94 (q, *J* = 7.2 Hz, 2H), 3.51 (d, *J* = 13.8 Hz, 1H), 3.38 (d, *J* = 13.8 Hz, 1H), 3.08 (q, *J* = 7.2 Hz, 2H), 1.65 (s, 2H), 1.46 (s, 3H), 1.36 (t, *J* = 7.8 Hz, 3H), 1.14 (t, *J* = 7.2 Hz, 3H); **^{13}C NMR (151 MHz, $CDCl_3$)** δ 177.18, 139.59, 133.24, 132.18, 131.15, 128.20, 125.28, 125.23, 124.30, 124.27, 61.09, 59.36, 42.53, 27.03, 25.93, 14.92, 13.98; **HRMS(ESI)** m/z: $[M+H]^+$ Calculated for $C_{18}H_{24}NO_2^+$ 286.1802; found 286.1798.

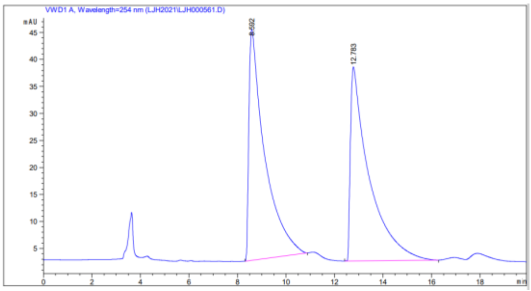

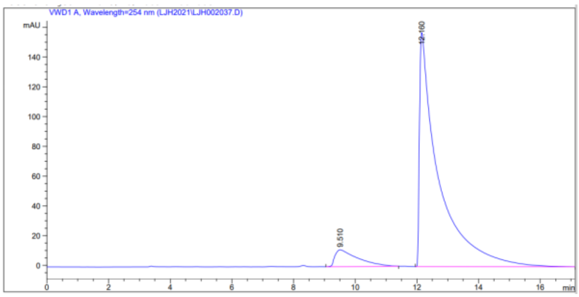

Peak #	RetTime [min]	Type	Width [min]	Area [mAU*s]	Height [mAU]	Area %
1	8.592	BB	0.6125	1933.60681	42.72653	50.2263
2	12.783	BB	0.6946	1916.17920	35.87431	49.7737

Peak #	RetTime [min]	Type	Width [min]	Area [mAU*s]	Height [mAU]	Area %
1	9.510	BB	0.6992	580.42920	11.39477	7.9251
2	12.160	BBA	0.5532	6743.51367	157.13216	92.0749

Ethyl (S)-2-amino-3-(4-methoxynaphthalen-1-yl)-2-methylpropanoate (6g):

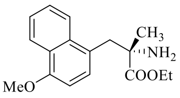

Colorless oil (53.5 mg, 92%); R_f = 0.21 (petroleum ether/ethyl acetate = 2:1); the enantiomeric excess was determined to be 87% by HPLC analysis on Daicel Chirapak OD-H column (hexane/isopropanol = 90/10, flow rate 1.0 mL/min, T = 30 °C), UV 254 nm, t_R(major) 13,682 min, t_R(minor) 12.744 min; $[\alpha]_D^{25}$ = -7.96 (c=0.45, $CHCl_3$); **^{1}H NMR (600 MHz, $CDCl_3$)** δ 8.29 (d, *J* = 7.8 Hz, 1H), 8.08 (d, *J* = 8.4 Hz, 1H), 7.50 (m, 1H), 7.45 (t, *J* = 7.8 Hz, 1H), 7.26 (m, 1H), 6.75 (d, *J* = 7.8 Hz, 1H), 4.03 (q, *J* = 7.2 Hz,, 2H), 3.98 (s, 3H), 3.47 (d, *J* = 14.4 Hz, 1H), 3.31 (d, *J* = 14.4 Hz, 1H), 1.66 (s, 2H), 1.46 (s, 3H), 1.15 (t, *J* = 7.2 Hz, 3H); **^{13}C NMR (151 MHz, $CDCl_3$)** δ 177.25, 154.76, 133.75, 128.26, 126.23, 125.88, 124.92, 124.81, 124.32, 122.44, 103.26, 61.08, 59.36, 55.46, 42.13, 26.92, 14.01; **HRMS(ESI)** m/z: $[M+H]^+$ Calculated for $C_{17}H_{22}NO_3^+$ 288.1594; found 288.1588.

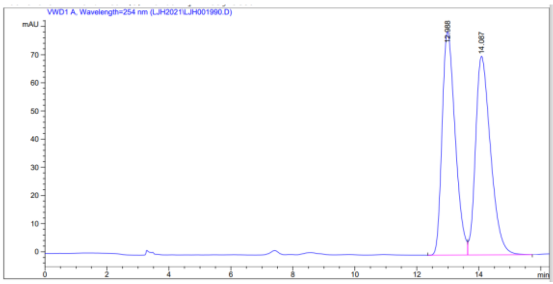

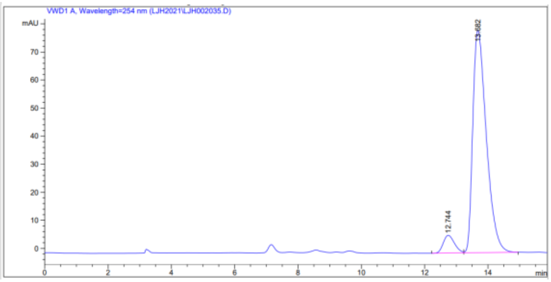

Peak #	RetTime [min]	Type	Width [min]	Area [mAU*s]	Height [mAU]	Area %
1	12.988	BV	0.4513	2326.70459	79.38637	49.4476
2	14.087	VB	0.5172	2378.69116	70.56109	50.5524

Peak #	RetTime [min]	Type	Width [min]	Area [mAU*s]	Height [mAU]	Area %
1	12.744	BV	0.3849	155.27435	6.33958	6.1950
2	13.682	VB	0.4597	2351.18896	79.26820	93.8050

Ethyl (S)-2-amino-3-(4-fluoronaphthalen-1-yl)-2-methylpropanoate (6h):

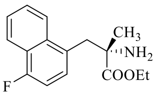

Colorless oil (46.9 mg, 85%); R_f = 0.22 (petroleum ether/ethyl acetate = 2:1); the enantiomeric excess was determined to be 82% by HPLC analysis on Daicel Chirapak AD-H column (hexane/isopropanol = 95/5, flow rate 1.0 mL/min, T = 30 °C), UV 254 nm, t_R(major) 8.974 min, t_R(minor) 8.099 min; $[\alpha]_D^{25}$ = -15.77 (c=0.74, $CHCl_3$); **^{1}H NMR (600 MHz, $CDCl_3$)** δ 8.13 (m, 2H), 7.54 (m, 2H), 7.34 – 7.23 (m, 1H), 7.07 (m, 1H), 4.02 (q, *J* = 7.2 Hz,, 2H), 3.49 (d, *J* = 14.4 Hz, 1H), 3.34 (d, *J* = 14.4 Hz, 1H), 1.63 (s, 2H), 1.46 (s, 3H), 1.14 (t, *J* = 7.2 Hz, 3H); **^{13}C NMR (151 MHz, $CDCl_3$)** δ 177.02, 158.94, 157.27, 134.15, 134.12, 129.03, 129.00, 127.95, 127.89, 126.65, 125.81, 124.67, 124.65, 124.03, 123.93, 121.04, 121.00, 108.78, 108.65, 61.17, 59.30, 42.16, 26.89, 13.98; **HRMS(ESI)** m/z: $[M+H]^+$ Calculated for $C_{16}H_{19}FNO_2^+$ 276.1394; found 276.1387.

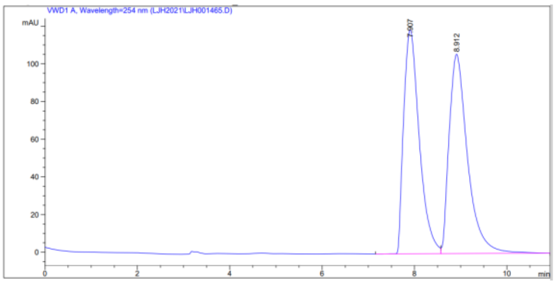

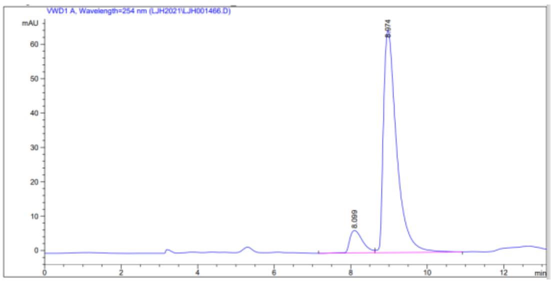

Peak #	RetTime [min]	Type	Width [min]	Area [mAU*s]	Height [mAU]	Area %
1	7.907	BV	0.3761	2791.62183	118.85358	48.7797
2	8.912	VBA	0.4356	2931.29468	105.77251	51.2203

Peak #	RetTime [min]	Type	Width [min]	Area [mAU*s]	Height [mAU]	Area %
1	8.099	BV	0.3680	154.75266	6.53722	9.1076
2	8.974	VB	0.3646	1544.40601	64.67361	90.8924

Ethyl (S)-2-((tert-butoxycarbonyl)amino)-2-methyl-3-(quinolin-6-yl)propanoate (6i):

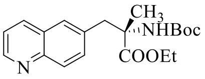

Colorless oil (44.5mg, 62%); R_f = 0.29 (petroleum ether/ethyl acetate = 5:1); the enantiomeric excess was determined to be 91% by HPLC analysis on Daicel Chirapak AD-H column (hexane/isopropanol = 95/5, flow rate 0.8 mL/min, T = 30 °C), UV 254 nm, t_R(major) 18.932 min, t_R(minor) 17.579 min; $[\alpha]_D^{25}$ = +5.78 (c=0.64, $CHCl_3$); **^{1}H NMR (600 MHz, $CDCl_3$)** δ 8.88 (m, 1H), 8.06 (d, *J* = 8.4 Hz, 1H), 8.00 (d, *J* = 8.4 Hz, 1H), 7.55 (s, 1H), 7.46 (m, 1H), 7.38 (m, 1H), 5.20 (s, 1H), 4.33 – 4.13 (q, *J* = 8.4 Hz, 2H), 3.61 (d, *J* = 13.2 Hz, 1H), 3.44 (d, *J* = 13.2 Hz, 1H), 1.62 (s, 3H), 1.49 (s, 9H), 1.31 (t, *J* = 7.2 Hz, 3H); **^{13}C NMR (151 MHz, $CDCl_3$)** δ 173.79, 154.35, 150.11, 147.44, 135.67, 135.26, 131.95, 129.07, 128.71, 128.04, 121.17, 79.43, 61.77, 60.30, 41.20, 28.44, 23.98, 14.16; **HRMS(ESI)** m/z: $[M+H]^+$ Calculated for $C_{20}H_{27}N_2O_4^+$ 359.1965 ; found 359.1966.

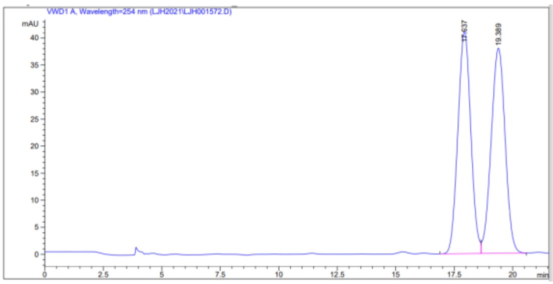

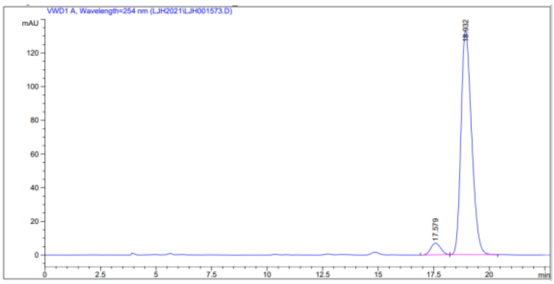

Peak #	RetTime [min]	Type	Width [min]	Area [mAU*s]	Height [mAU]	Area %
1	17.937	BV	0.5950	1569.15149	41.15047	49.9332
2	19.389	VB	0.6548	1573.34717	37.90837	50.0668

Peak #	RetTime [min]	Type	Width [min]	Area [mAU*s]	Height [mAU]	Area %
1	17.579	BB	0.4880	212.66557	6.92600	4.6147
2	18.932	BB	0.5106	4395.81592	133.11978	95.3853

Ethyl (S)-2-((tert-butoxycarbonyl)amino)-2-methyl-3-(2-methylquinolin-6-yl)propanoate (6j):

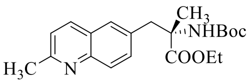

White solid (59.1 mg, 79%); m.p. = 119-121 °C; R_f = 0.32 (petroleum ether/ethyl acetate = 5:1); the enantiomeric excess was determined to be 92% by HPLC analysis on Daicel Chirapak AD-H column (hexane/isopropanol = 95/5, flow rate

0.8 mL/min, T = 30 ºC), UV 254 nm, t_R(major) 17.678 min, t_R(minor) 14.799 min; $[\alpha]_D^{25}$ = +2.95 (c=0.85, $CHCl_3$); **^{1}H NMR (600 MHz, $CDCl_3$)** δ 7.92 (m, 2H), 7.49 (s, 1H), 7.41 (m, 1H), 7.30 – 7.21 (m, 1H), 5.19 (s, 1H), 4.21 (q, *J* = 7.2 Hz, 2H), 3.56 (d, *J* = 13.8 Hz, 1H), 3.41 (d, *J* = 13.8 Hz, 1H), 2.75 (s, 3H), 1.61 (s, 3H), 1.49 (s, 9H), 1.30 (t, *J* = 7.2 Hz, 3H); **^{13}C NMR (151 MHz, $CDCl_3$)** δ 173.82, 158.68, 154.34, 147.01, 135.79, 134.20, 131.87, 128.42, 128.24, 126.22, 122.06, 79.45, 61.70, 60.29, 41.26, 28.42, 25.27, 23.93, 14.15; **HRMS(ESI)** m/z: $[M+H]^+$ Calculated for $C_{21}H_{29}N_2O_4^+$ 373.2122; found 373.2124.

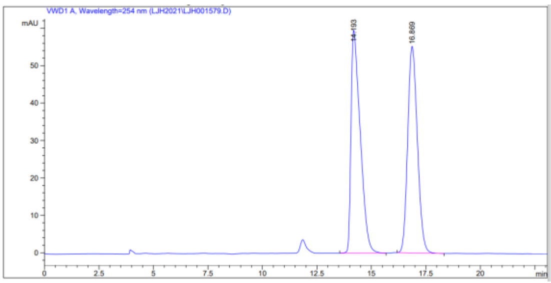

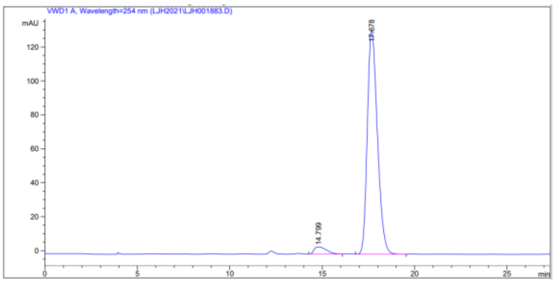

Peak #	RetTime [min]	Type	Width [min]	Area [mAU*s]	Height [mAU]	Area %
1	14.193	BB	0.4215	1742.08740	59.53989	50.4974
2	16.869	BB	0.4819	1707.76868	55.21155	49.5026

Peak #	RetTime [min]	Type	Width [min]	Area [mAU*s]	Height [mAU]	Area %
1	14.799	BB	0.7144	196.39095	4.20137	3.7964
2	17.678	BB	0.5861	4976.64209	131.86073	96.2036

Ethyl (S)-2-((tert-butoxycarbonyl)amino)-2-methyl-3-(2-phenylquinolin-7-yl)propanoate (6k):

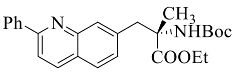

Colorless oil (81.5 mg, 94%); R_f = 0.38 (petroleum ether/ethyl acetate = 5:1); the enantiomeric excess was determined to be 83% by HPLC analysis on Daicel Chirapak AD-H column (hexane/isopropanol = 90/10, flow rate 1.0 mL/min, T = 30 ºC), UV 254 nm, t_R(major) 10.618 min, t_R(minor) 8.379 min; $[\alpha]_D^{25}$ = -7.64 (c=1.43, $CHCl_3$); **^{1}H NMR (400 MHz, $CDCl_3$)** δ 8.36 – 8.09 (m, 3H), 7.93 (s, 1H), 7.83 (d, *J* = 12.6 Hz, 1H), 7.71 (d, *J* = 12.6 Hz, 1H), 7.49 (m, 3H), 7.32 – 7.17 (m, 1H), 5.17 (s, 1H), 4.34 – 4.20 (m, 2H), 3.59 (d, *J* = 19.6 Hz, 1H), 3.48 (d, *J* = 19.8 Hz, 1H), 1.62 (s, 3H), 1.52 (s, 9H), 1.32 (t, *J* = 10.8 Hz, 3H); **^{13}C NMR (101 MHz, $CDCl_3$)** δ 173.88, 157.28, 154.45, 148.18, 139.68, 138.45, 136.35, 130.83, 129.29, 128.92, 128.79, 127.49, 126.99, 126.05, 118.61, 79.64, 61.76, 60.22, 28.47, 23.92, 18.69, 14.18; **HRMS(ESI)** m/z: $[M+H]^+$ Calculated for $C_{26}H_{31}N_2O_4^+$ 435.2278; found 435.2278.

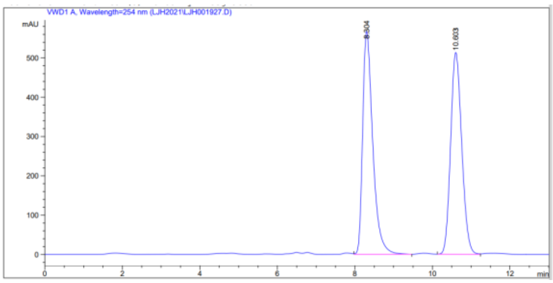

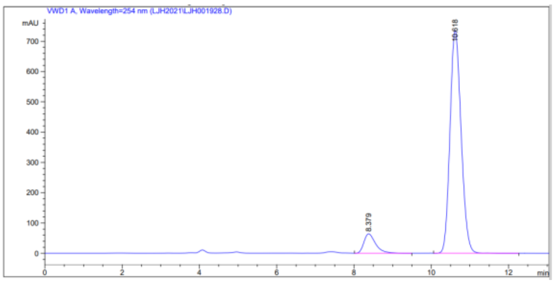

Peak #	RetTime [min]	Type	Width [min]	Area [mAU*s]	Height [mAU]	Area %
1	8.304	VB	0.2754	1.02684e4	566.99695	50.1790
2	10.603	BV	0.3061	1.01952e4	513.17499	49.8210

Peak #	RetTime [min]	Type	Width [min]	Area [mAU*s]	Height [mAU]	Area %
1	8.379	VB	0.3219	1353.39978	64.15916	8.4437
2	10.618	BB	0.3114	1.46751e4	735.68768	91.5563

Ethyl (S)-2-((tert-butoxycarbonyl)amino)-2-methyl-3-(quinolin-7-yl)propanoate (6l):

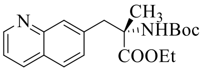

Colorless oil (69.8 mg, 93%); R_f = 0.37 (petroleum ether/ethyl acetate = 5:1); the enantiomeric excess was determined to be 87% by HPLC analysis on Daicel Chirapak AD-H column (hexane/isopropanol = 95/5, flow rate 1.0 mL/min, T = 30 °C), UV 254 nm, t_R(major) 19.228min, t_R(minor) 15.523 min; $[\alpha]_D^{25}$ = +15.20 (c=1.32, $CHCl_3$); **^{1}H NMR (600 MHz, $CDCl_3$)** δ 8.88 (m, 1H), 8.11 (d, *J* = 7.8 Hz, 1H), 7.84 (s, 1H), 7.72 (d, *J* = 8.4 Hz, 1H), 7.34 (m, 2H), 5.25 (s, 1H), 4.33 – 4.17 (q, *J* = 7.8 Hz, 2H), 3.61 (d, *J* = 14.4 Hz, 1H), 3.47 (d, *J* = 14.4 Hz, 1H), 1.62 (s, 3H), 1.51 (s, 9H), 1.31 (t, *J* = 7.2 Hz, 3H); **^{13}C NMR (151 MHz, $CDCl_3$)** δ 173.80, 154.38, 150.47, 148.18, 138.42, 135.65, 130.29, 129.25, 127.32, 127.15, 120.79, 79.57, 61.78, 60.28, 41.59, 28.43, 23.88, 14.16; **HRMS(ESI)** m/z: $[M+H]^+$ Calculated for $C_{20}H_{27}N_2O_4^+$ 359.1965; found 359.1964.

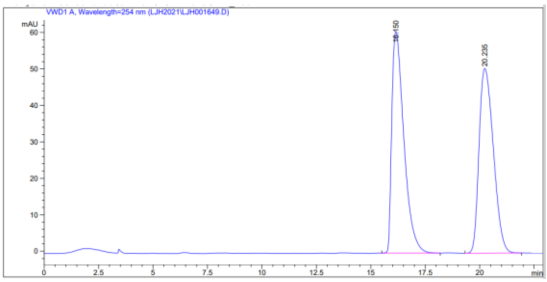

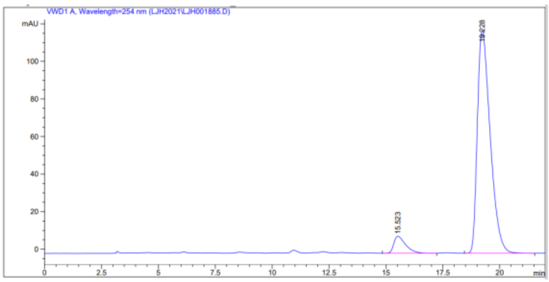

Peak #	RetTime [min]	Type	Width [min]	Area [mAU*s]	Height [mAU]	Area %
1	16.150	BB	0.5956	2338.42798	60.84239	50.2321
2	20.235	BB	0.7245	2316.81909	50.65685	49.7679

Peak #	RetTime [min]	Type	Width [min]	Area [mAU*s]	Height [mAU]	Area %
1	15.523	BB	0.5403	328.36600	9.04301	6.6049
2	19.228	BB	0.6046	4643.18945	118.42770	93.3951

Ethyl (S)-2-((tert-butoxycarbonyl)amino)-2-methyl-3-(quinoxalin-6-yl)propanoate (6m):

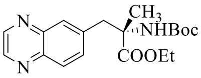

Colorless oil (58.7 mg, 81%); R_f = 0.38 (petroleum ether/ethyl acetate = 5:1); the enantiomeric excess was determined to be 90% by HPLC analysis on Daicel Chirapak AD-H column (hexane/isopropanol = 95/5, flow rate 0.8 mL/min, T = 30 °C), UV 254 nm, t_R(major) 15.849 min, t_R(minor) 14.399 min; $[\alpha]_D^{25}$ = +13.40 (c=1.05, $CHCl_3$); **^{1}H NMR**

(600 MHz, CDCl₃) δ 8.81 (d, *J* = 9.6 Hz, 2H), 8.00 (d, *J* = 9.0 Hz, 1H), 7.84 (s, 1H), 7.55 (d, *J* = 8.4 Hz, 1H), 5.27 (s, 1H), 4.36 – 4.07 (q, *J* = 9.0 Hz 2H), 3.68 (d, *J* = 13.2 Hz,, 1H), 3.51 (d, *J* = 13.2 Hz, 1H), 1.63 (s, 3H), 1.50 (s, 9H), 1.33 (t, *J* = 7.2 Hz, 3H); **^{13}C NMR (151 MHz, $CDCl_3$)** δ 173.64, 154.35, 145.01, 144.59, 142.81, 142.08, 139.43, 132.73, 130.21, 128.89, 79.72, 61.88, 60.29, 41.21, 28.41, 23.97, 14.15; **HRMS(ESI)** m/z: $[M+H]^+$ Calculated for $C_{19}H_{26}N_3O_4^+$ 360.1918; found 360.1916.

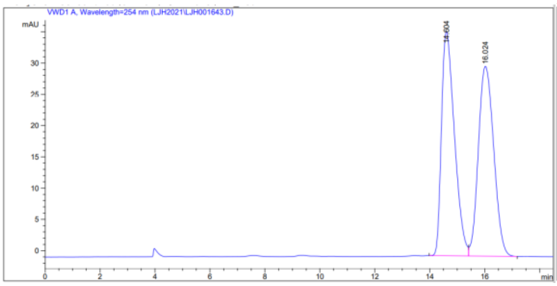

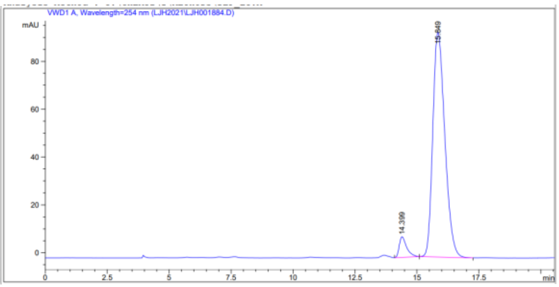

Peak #	RetTime [min]	Type	Width [min]	Area [mAU*s]	Height [mAU]	Area %
1	14.604	BV	0.4995	1171.28723	35.82538	49.8060
2	16.024	VB	0.6140	1180.41357	30.35711	50.1940

Peak #	RetTime [min]	Type	Width [min]	Area [mAU*s]	Height [mAU]	Area %
1	14.399	BB	0.3094	177.66331	8.60625	5.3061
2	15.849	BB	0.5238	3170.59473	93.86991	94.6939

Ethyl 3-(1-acetyl-1H-indol-3-yl)-2-((tert-butoxycarbonyl)amino)-2-methylpropanoate (6n):

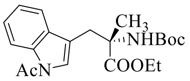

Colorless oil (49.9 mg, 65%); R_f= 0.29 (petroleum ether/ethyl acetate = 5:1); the enantiomeric excess was determined to be 84% by HPLC analysis on Daicel Chirapak IC-H column (hexane/isopropanol = 90/10, flow rate 1.0 mL/min, T = 30 °C), UV 254 nm, t_R(major) 18.691 min, t_R(minor) 16.175 min; $[\alpha]_D^{25}$ = +23.04 (c=0.84, $CHCl_3$); **^{1}H NMR (400 MHz, $CDCl_3$)** δ 8.39 (d, *J* = 8.0 Hz, 1H), 7.52 (d, *J* = 8.0 Hz, 1H), 7.33 (t, *J* = 8.0 Hz, 1H), 7.28 – 7.22 (m, 1H), 7.19 (s, 1H), 5.27 (s, 1H), 4.26 – 4.00 (m, 2H), 3.51 (d, *J* = 16.0 Hz, 1H), 3.39 (d, *J* = 16.0 Hz, 1H), 2.59 (s, 3H), 1.61 (s, 3H), 1.48 (s, 9H), 1.25 (t, *J* = 4.0 Hz, 3H); **^{13}C NMR (101 MHz, $CDCl_3$)** δ 173.96, 168.25, 154.38, 135.47, 131.26, 125.17, 124.24, 123.42, 119.20, 117.46, 116.46, 79.63, 61.78, 59.82, 30.96, 28.44, 24.11, 23.97, 14.05; **HRMS(ESI)** m/z: $[M+H]^+$ Calculated for $C_{21}H_{29}N_2O_5^+$ 389.2071; found 389.2068.

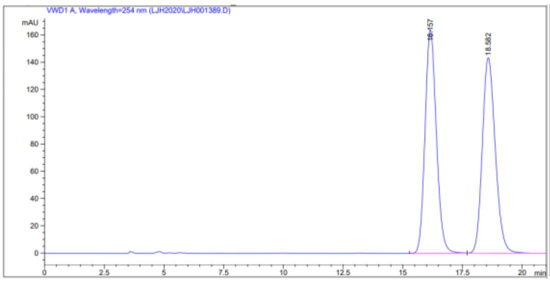

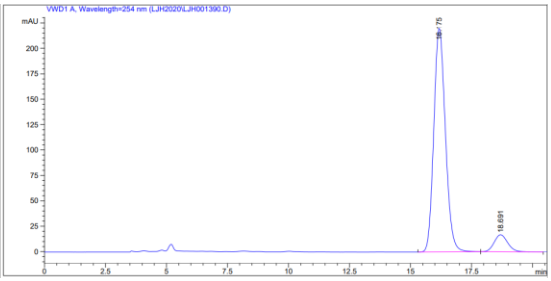

Peak #	RetTime [min]	Type	Width [min]	Area [mAU*s]	Height [mAU]	Area %
1	16.157	BB	0.5080	5398.26660	163.95268	49.9889
2	18.582	BBA	0.5850	5400.65967	143.45322	50.0111

Peak #	RetTime [min]	Type	Width [min]	Area [mAU*s]	Height [mAU]	Area %
1	16.175	BB	0.5137	7260.26611	219.79112	91.8345
2	18.691	BBA	0.5950	645.54669	16.76650	8.1655

Ethyl (S)-2-amino-3-(anthracen-2-yl)-2-methylpropanoate (6o):

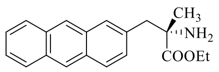

A pale yellow solid (50.7 mg, 82%); m.p. = 75-77 ºC; R_f = 0.22 (petroleum ether/ethyl acetate = 2:1); the enantiomeric excess was determined to be 88% by HPLC analysis on Daicel Chirapak IA-H column (hexane/isopropanol = 80/20, flow rate 1.0 mL/min, T = 30 ºC), UV 254 nm, t_R(major) 17.992 min, t_R(minor) 9.030 min; $[\alpha]_D^{25}$ = -40.66 (c=0.91, $CHCl_3$); **^{1}H NMR (600 MHz, CDCl$_3$)** δ 8.35 (d, *J* = 20.4 Hz, 2H), 7.97 (m, 2H), 7.91 (d, *J* = 8.4 Hz, 1H), 7.79 (s, 1H), 7.52 – 7.36 (m, 2H), 7.28 (d, *J* = 8.4 Hz, 1H), 4.17 (q, *J* = 7.2 Hz, 2H), 3.32 (d, *J* = 13.2 Hz, 1H), 3.00 (d, *J* = 13.2 Hz, 1H), 1.80 (s, 2H), 1.46 (s, 3H), 1.25 (t, *J* = 7.2 Hz, 3H); **^{13}C NMR (151 MHz, CDCl$_3$)** δ 177.05, 133.74, 131.88, 131.61, 131.58, 130.78, 128.70, 128.18, 128.16, 128.11, 128.10, 125.98, 125.84, 125.41, 125.27, 61.19, 58.90, 47.12, 26.84, 14.25; **HRMS(ESI)** m/z: $[M+H]^+$ Calculated for $C_{20}H_{22}NO_2^+$ 308.1645; found 308.1643.

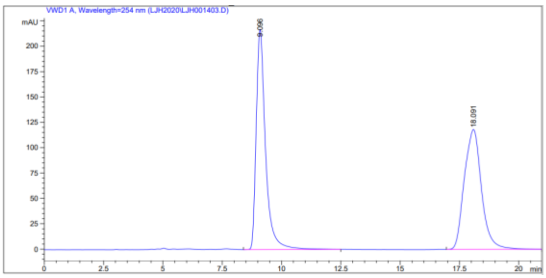

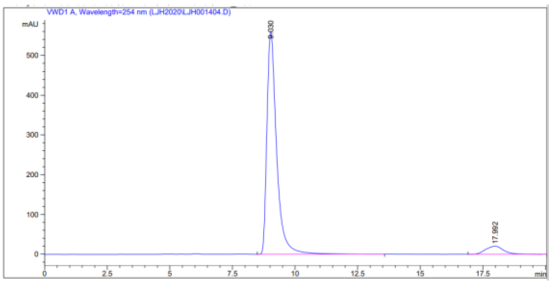

Peak #	RetTime [min]	Type	Width [min]	Area [mAU*s]	Height [mAU]	Area %
1	9.096	BB	0.4133	5921.81787	216.87189	49.9106
2	18.091	BBA	0.8089	5943.04004	118.05328	50.0894

Peak #	RetTime [min]	Type	Width [min]	Area [mAU*s]	Height [mAU]	Area %
1	9.030	BB	0.4105	1.51061e4	560.81256	93.6431
2	17.992	BBA	0.8042	1025.47131	20.43066	6.3569

Ethyl (S)-2-amino-2-methyl-3-(phenanthren-9-yl)propanoate (6p):

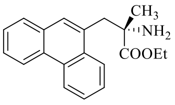

White solid (55.9 mg, 90%); m.p. = 85-87 ºC; R_f = 0.24 (petroleum ether/ethyl acetate = 2:1); the enantiomeric excess was determined to be 88% by HPLC analysis on Daicel Chirapak IC-H column (hexane/isopropanol = 90/10, flow rate 1.0 mL/min, T = 30 ºC), UV 254 nm, t_R(major) 32.559 min, t_R(minor) 22.353 min; $[\alpha]_D^{25}$ = -2.75 (c=0.91, $CHCl_3$); **^{1}H NMR (600 MHz, CDCl$_3$)** δ 8.75 – 8.67 (m, 1H), 8.63 (d, *J* = 7.8 Hz, 1H), 8.21 (d, *J* = 7.2 Hz, 1H), 7.81 (d, *J* = 7.2 Hz, 1H), 7.66 – 7.58 (m, 4H), 7.56 (m, 1H), 4.04 – 3.94 (q, *J* = 7.2 Hz,, 2H), 3.60 (d, *J* = 14.4 Hz, 1H), 3.43 (d, *J* = 14.4 Hz, 1H), 1.78 (s, 2H), 1.51 (s, 3H), 1.08 (t, *J* = 7.2 Hz, 3H); **^{13}C NMR (151 MHz, CDCl$_3$)** δ 177.06, 131.91, 131.51, 131.40, 130.70, 129.99, 129.08,

128.27, 126.70, 126.49, 126.38, 126.28, 125.17, 123.15, 122.48, 61.19, 59.30, 42.87, 27.17, 13.99; **HRMS(ESI)** m/z: $[M+H]^+$ Calculated for $C_{20}H_{22}NO_2^+$ 308.1645; found 308.1641.

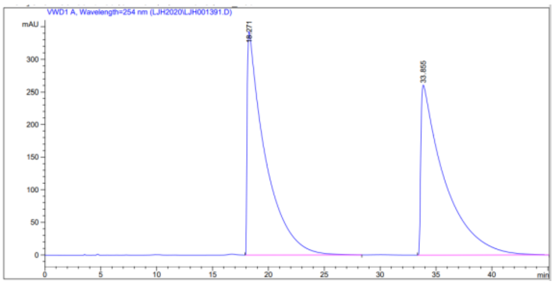

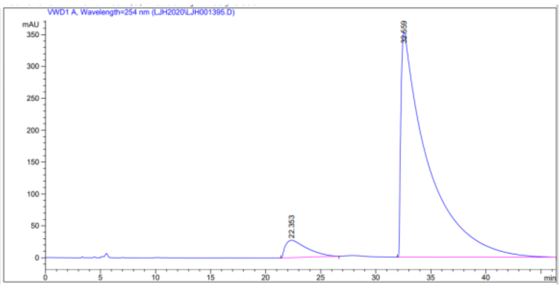

Peak #	RetTime [min]	Type	Width [min]	Area [mAU*s]	Height [mAU]	Area %
1	18.271	BB	1.4332	3.80999e4	342.92929	50.0395
2	33.855	BBA	1.8798	3.80397e4	260.76126	49.9605

Peak #	RetTime [min]	Type	Width [min]	Area [mAU*s]	Height [mAU]	Area %
1	22.353	BB	1.9261	3694.30078	27.34391	5.8447
2	32.559	BBA	2.1695	5.95139e4	353.52863	94.1553

Ethyl (S)-2-amino-2-methyl-3-(2,3,6-trimethoxyphenanthren-9-yl)propanoate (6q):

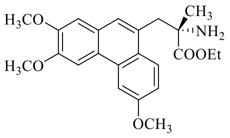

White solid (70.5 mg, 88%); m.p. = 81-83 °C; R_f = 0.25 (petroleum ether/ethyl acetate = 1:2); the enantiomeric excess was determined to be 95% by HPLC analysis on Daicel Chirapak OD-H column (hexane/isopropanol = 70/30, flow rate 1.0 mL/min, T = 30 °C), UV 254 nm, t_R(major) 21.510 min, t_R(minor) 14.249 min; $[\alpha]_D^{25}$ = -4.54 (c=1.11, $CHCl_3$); **^{1}H NMR (600 MHz, $CDCl_3$)** δ 8.11 (d, J = 9.0 Hz, 1H), 7.87 (s, 1H), 7.83 (s, 1H), 7.41 (s, 1H), 7.20 (d, J = 9.0 Hz, 1H), 7.14 (s, 1H), 4.09 (s, 3H), 4.01 (m, 8H), 3.55 (d, J = 14.4 Hz, 1H), 3.36 (d, J = 14.4 Hz, 1H), 1.94 (s, 2H), 1.50 (s, 3H), 1.10 (t, J = 7.2 Hz, 3H); **^{13}C NMR (151 MHz, $CDCl_3$)** δ 177.08, 157.78, 149.61, 149.04, 131.59, 129.49, 127.11, 126.86, 125.95, 125.85, 123.84, 114.73, 108.00, 104.51, 103.33, 61.16, 59.25, 56.08, 55.90, 55.50, 42.86, 27.09, 14.00; **HRMS(ESI)** m/z: $[M+H]^+$ Calculated for $C_{23}H_{28}NO_5^+$ 398.1962; found 398.1964.

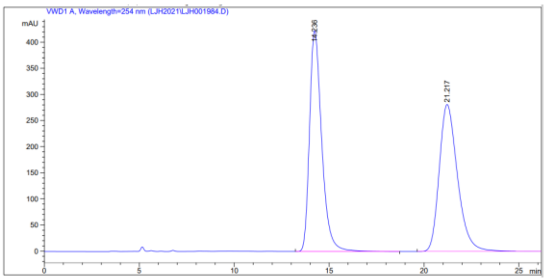

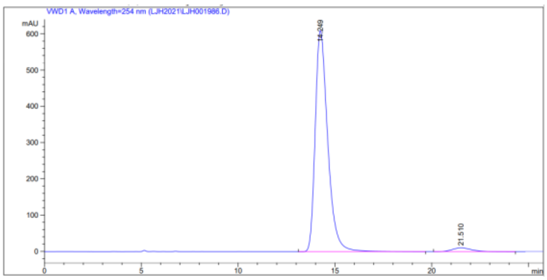

Peak #	RetTime [min]	Type	Width [min]	Area [mAU*s]	Height [mAU]	Area %
1	14.236	BB	0.6835	1.88629e4	422.99799	49.9803
2	21.217	BBA	1.0270	1.88778e4	281.61667	50.0197

Peak #	RetTime [min]	Type	Width [min]	Area [mAU*s]	Height [mAU]	Area %
1	14.249	BB	0.6861	2.74055e4	609.82220	97.4276
2	21.510	BB	1.0392	723.59882	10.38109	2.5724

Ethyl (S)-2-amino-2-methyl-3-6-(3-(adamantan-1-yl)-4-methoxyphenyl)naphthalen-2-yl)-2,2-dimethylpropanoate (6r):

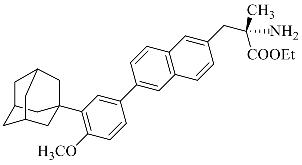

White solid (95.5 mg, 95%); m.p. = 89-91 ºC; R_f = 0.23 (petroleum ether/ethyl acetate = 2:1); the enantiomeric excess was determined to be 88% by HPLC analysis on Daicel Chirapak IA-H column (hexane/isopropanol = 90/10, flow rate 1.0 mL/min, T = 30 ºC), UV 254 nm, t_R(major) 13.212min, t_R(minor) 11.688 min; $[\alpha]_D^{25}$ = -20.63 (c=1.94, $CHCl_3$); **^{1}H NMR (600 MHz, $CDCl_3$)** δ 7.94 (s, 1H), 7.79 (t, *J* = 8.4 Hz, 2H), 7.70 (d, *J* = 7.8 Hz, 1H), 7.64 (s, 1H), 7.57 (s, 1H), 7.51 – 7.46 (m, 1H), 7.29 (d, *J* = 8.4 Hz, 1H), 6.95 (d, *J* = 8.4 Hz, 1H), 4.16 (q, *J* = 7.2 Hz, 2H), 3.85 (s, 3H), 3.29 (d, *J* = 13.2 Hz, 1H), 2.97 (d, *J* = 13.2 Hz, 1H), 2.18 (s, 6H), 2.09 (s, 3H), 1.89 (s, 2H), 1.79 (s, 6H), 1.44 (s, 3H), 1.25 (t, *J* = 6.6 Hz, 3H); **^{13}C NMR (151 MHz, $CDCl_3$)** δ 176.99, 158.61, 138.90, 138.86, 133.90, 133.22, 132.85, 132.20, 128.65, 128.59, 128.03, 128.00, 125.95, 125.90, 125.61, 124.77, 112.17, 61.18, 58.92, 55.19, 46.98, 40.71, 37.22, 29.21, 26.79, 14.29; **HRMS(ESI)** m/z: $[M+H]^+$ Calculated for $C_{33}H_{40}NO_3^+$ 498.3003; found 498.3012.

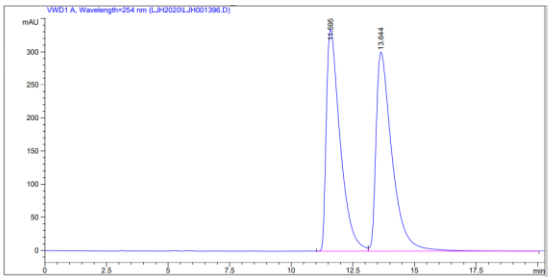

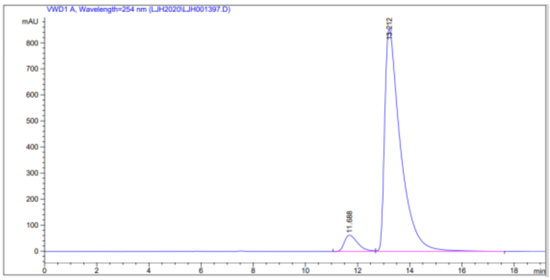

Peak #	RetTime [min]	Type	Width [min]	Area [mAU*s]	Height [mAU]	Area %
1	11.595	BV	0.5802	1.30669e4	335.20374	49.0935
2	13.644	VB	0.6680	1.35495e4	301.00998	50.9065

Peak #	RetTime [min]	Type	Width [min]	Area [mAU*s]	Height [mAU]	Area %
1	11.688	BV	0.5427	2202.15649	62.44096	5.8029
2	13.212	VB	0.6070	3.57471e4	855.54034	94.1971

tert-Butyl (S)-2-amino-2-methyl-3-phenylpropanoate (7b):

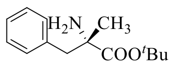

A pale yellow oil (39.3 mg, 83%); R_f = 0.26 (petroleum ether/ethyl acetate = 2:1); the enantiomeric excess was determined to be 95% by HPLC analysis on Daicel Chirapak OJ-H column (hexane/isopropanol = 98/2, flow rate 0.8 mL/min, T = 30 ºC), UV 220 nm, t_R(major) 12.614 min, t_R(minor) 10.387 min; $[\alpha]_D^{25}$ = -27.54 (c=0.47, $CHCl_3$); **^{1}H NMR (600 MHz, $CDCl_3$)** δ 7.32 – 7.26 (m, 2H), 7.23 (m, 3H), 3.11 (d, J = 12.0 Hz, 1H), 2.78 (d, J = 12.0 Hz, 1H), 1.62 (s, 2H), 1.46 (s, 9H), 1.34 (s, 3H); **^{13}C NMR (151 MHz, $CDCl_3$)** δ 176.31, 136.87, 130.22, 128.17, 126.79, 81.10, 58.76, 46.44, 28.00, 27.02; **HRMS(ESI)** m/z: $[M+H]^+$ Calculated for $C_{14}H_{22}NO_2^+$ 236.1645; found 236.1640.

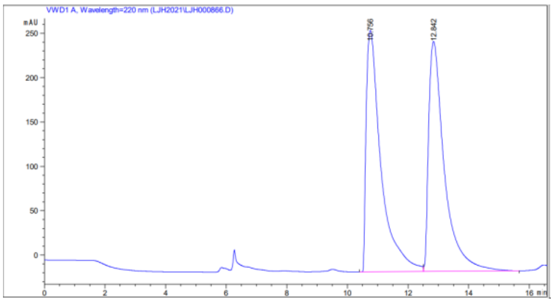

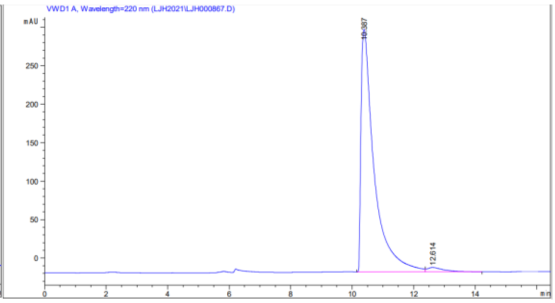

Peak #	RetTime [min]	Type	Width [min]	Area [mAU*s]	Height [mAU]	Area %
1	10.756	BV	0.5053	9270.35254	272.15164	49.7733
2	12.842	VB	0.5422	9354.78516	259.08063	50.2267

Peak #	RetTime [min]	Type	Width [min]	Area [mAU*s]	Height [mAU]	Area %
1	10.387	BV	0.4244	9341.16016	315.27921	97.7010
2	12.614	VB	0.5362	219.80809	5.71541	2.2990

tert-Butyl (S)-2-amino-2-methyl-3-(o-tolyl)propanoate (7c):

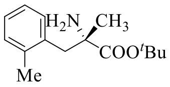

Colorless oil (26.7 mg, 53%); R_f= 0.27 (petroleum ether/ethyl acetate = 2:1); the enantiomeric excess was determined to be 96% by HPLC analysis on Daicel Chirapak IC-H column (hexane/isopropanol = 90/10, flow rate 1 mL/min, T = 30 ºC), UV 220 nm, t_R(major) 13.072 min, t_R(minor) 11.965 min; $[\alpha]_D^{25}$ = -9.49 (c=0.53, $CHCl_3$); **^{1}H NMR (600 MHz, $CDCl_3$)** δ 7.21 – 7.17 (m, 1H), 7.17 – 7.08 (m, 3H), 3.06 (d, *J* = 12.0 Hz, 1H), 2.94 (d, *J* = 12.0 Hz, 1H), 2.38 (s, 3H), 1.58 (s, 2H), 1.46 (s, 9H), 1.34 (s, 3H); **^{13}C NMR (151 MHz, $CDCl_3$)** δ 176.76, 137.50, 135.44, 130.53, 130.37, 126.69, 125.60, 81.09, 59.46, 42.14, 27.95, 27.06, 20.42; **HRMS(ESI)** m/z: $[M+H]^+$ Calculated for $C_{15}H_{24}NO_2^+$ 250.1802; found 250.1797.

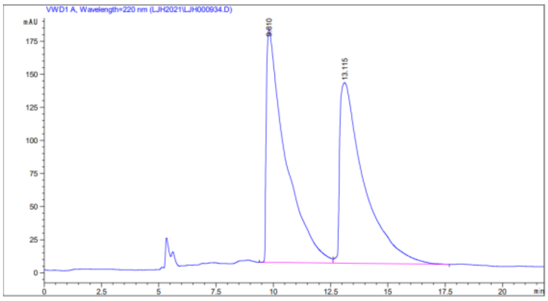

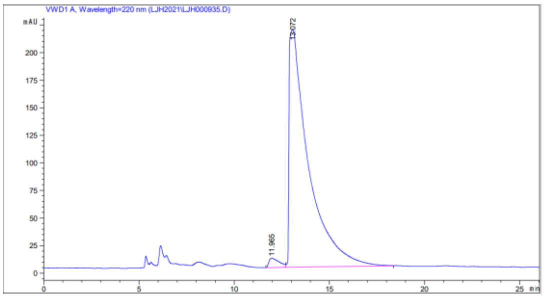

Peak #	RetTime [min]	Type	Width [min]	Area [mAU*s]	Height [mAU]	Area %
1	9.810	BV	0.7862	1.02827e4	175.72530	50.5689
2	13.115	VB	1.0241	1.00513e4	136.45082	49.4311

Peak #	RetTime [min]	Type	Width [min]	Area [mAU*s]	Height [mAU]	Area %
1	11.965	BV	0.5634	316.00082	8.38861	2.0487
2	13.072	VB	0.9351	1.51085e4	215.37021	97.9513

tert-Butyl (S)-2-amino-2-methyl-3-(m-tolyl)propanoate (7d):

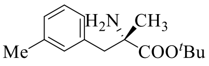

Colorless oil (44.1 mg, 88%); R_f= 0.25 (petroleum ether/ethyl acetate = 2:1); the enantiomeric excess was determined to be 97% by HPLC analysis on Daicel Chirapak IC-H column (hexane/isopropanol = 70/30, flow rate 1 mL/min, T = 30 ºC), UV 220 nm, t_R(major) 12.708 min, t_R(minor) 8.316 min; $[\alpha]_D^{25}$ = -31.57 (c=0.64, $CHCl_3$); **^{1}H NMR (600 MHz, $CDCl_3$)** δ 7.16 (t, *J* = 6.0 Hz, 1H), 7.02 (m, 3H), 3.09 (d, *J* = 12.0 Hz, 1H), 2.73 (d, *J* =

12.0 Hz, 1H), 2.31 (s, 3H), 1.62 (s, 2H), 1.46 (s, 9H), 1.34 (s, 3H); **^{13}C NMR (151 MHz, CDCl$_3$)** δ 176.37, 137.65, 136.76, 130.94, 128.07, 127.52, 127.20, 81.04, 58.76, 46.36, 28.01, 27.14, 21.34; **HRMS(ESI)** m/z: $[M+H]^+$ Calculated for $C_{15}H_{24}NO_2^+$ 250.1802; found 250.1796.

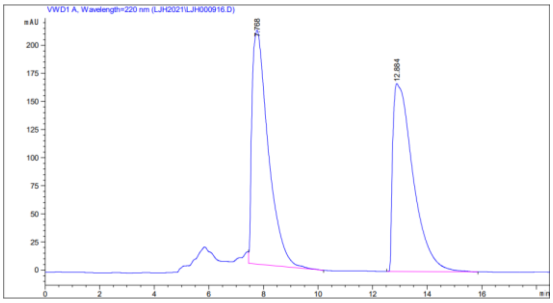

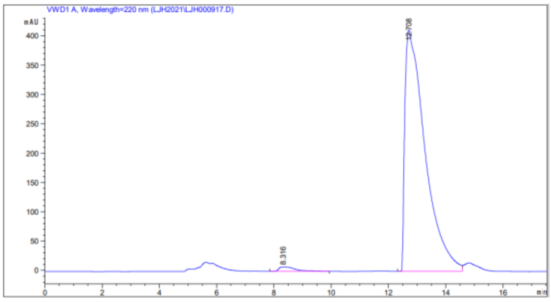

Peak #	RetTime [min]	Type	Width [min]	Area [mAU*s]	Height [mAU]	Area %
1	7.768	VB	0.6265	8607.03125	208.22993	49.8685
2	12.884	BB	0.6754	8652.42480	167.12894	50.1315

Peak #	RetTime [min]	Type	Width [min]	Area [mAU*s]	Height [mAU]	Area %
1	8.316	BB	0.5301	301.25381	7.54283	1.4769
2	12.708	BV	0.6399	2.00961e4	411.40137	98.5231

tert-Butyl (S)-2-amino-3-(3-fluorophenyl)-2-methylpropanoate (7e):

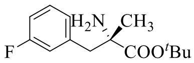

Colorless oil (42.0 mg, 83%); R_f= 0.27 (petroleum ether/ethyl acetate = 2:1); the enantiomeric excess was determined to be 98% by HPLC analysis on Daicel Chirapak IC-H column (hexane/isopropanol = 90/10, flow rate 1 mL/min, T = 30 ºC), UV 220 nm, t_R(major) 9.505 min, t_R(minor) 6.034 min; $[\alpha]_D^{25}$ = -25.36 (c=0.46, CHCl$_3$); **^{1}H NMR (600 MHz, CDCl$_3$)** δ 7.24 (m, 1H), 7.00 (d, J = 6.0 Hz, 1H), 6.98 – 6.91 (m, 2H), 3.09 (d, J = 12.0 Hz, 1H), 2.77 (d, J = 12.0 Hz, 1H), 1.60 (s, 2H), 1.46 (s, 9H), 1.35 (s, 3H).; **^{13}C NMR (151 MHz, CDCl$_3$)** δ 176.07, 163.43, 161.80, 139.46, 139.41, 129.56, 129.50, 125.91, 125.89, 117.12, 116.98, 113.75, 113.62, 81.35, 58.74, 46.11, 27.98, 27.01; **HRMS(ESI)** m/z: $[M+H]^+$ Calculated for $C_{14}H_{21}FNO_2^+$ 254.1551; found 254.1546.

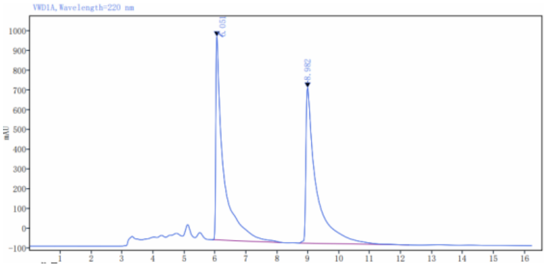

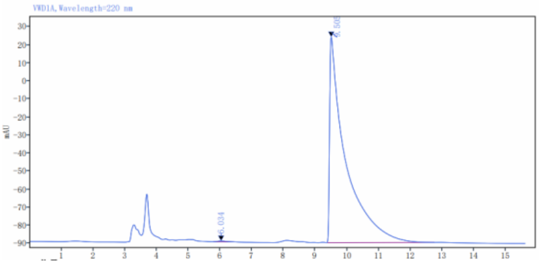

Peak	RetTime[min]	Type	Width[min]	Area[mAU^s]	Height[mAU]	Area%
	6.051	BB	2.3297	18063.5865	1031.1829	49.4770
	8.982	BB	3.5656	18445.4450	788.1207	50.5230

Peak	RetTime[min]	Type	Width[min]	Area[mAU^s]	Height[mAU]	Area%
	6.034	MM m	0.2860	8.5426	0.4449	0.2137
	9.505	BB	3.4007	3988.1838	113.9117	99.7863

tert-Butyl (S)-2-(((benzyloxy)carbonyl)amino)-2-methyl-3-(4-(trifluoromethyl)phenyl)propanoate (N-Cbz-7f):

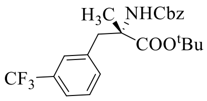

Colorless oil (53,3 mg, 61%); R_f= 0.38 (petroleum ether/ethyl acetate = 5:1); the enantiomeric excess was determined to be 98% by HPLC analysis on Daicel Chirapak OD-H column (hexane/isopropanol = 80/20, flow rate 1 mL/min, T = 30 ºC), UV 254 nm, t_R(major) 8.274 min, t_R(minor) 5.590 min; $[\alpha]_D^{25}$ = +97.40 (c=0.51, $CHCl_3$); **^{1}H NMR (600 MHz, $CDCl_3$)** δ 7.45 (d, J = 6.0 Hz, 1H), 7.41 – 7.30 (m, 6H), 7.29 – 7.23 (m, 1H), 7.17 (d, J = 6.0 Hz, 1H), 5.62 (s, 1H), 5.17 (d, J = 12.0 Hz, 1H), 5.07 (d, J = 12.0 Hz, 1H), 3.61 (d, J = 18.0 Hz, 1H), 3.18 (d, J = 18.0 Hz, 1H), 1.64 (s, 3H), 1.45 (s, 9H); **^{13}C NMR (151 MHz, $CDCl_3$)** δ 172.26, 154.57, 137.68, 136.61, 133.45, 130.44, 130.23, 128.53, 128.50, 128.15, 128.13, 126.63, 126.61, 125.07, 123.64, 123.61, 123.27, 83.01, 66.40, 60.87, 40.66, 29.71, 27.83, 24.27; **HRMS(ESI)** m/z: $[M+H]^+$ Calculated for $C_{23}H_{27}F_3NO_4^+$ 438.1887; found 438.1885.

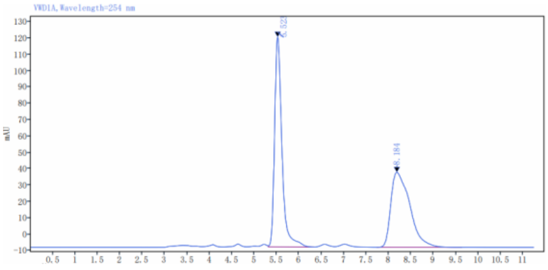

Peak	RetTime[min]	Type	Width[min]	Area[mAU*s]	Height[mAU]	Area%
	5.523	VB	1.0254	1466.5596	128.2821	52.1884
	8.184	BB	1.9033	1343.5659	45.7532	47.8116

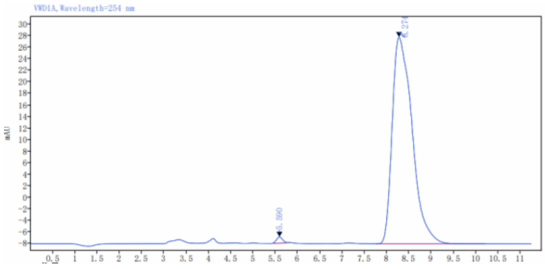

Peak	RetTime[min]	Type	Width[min]	Area[mAU*s]	Height[mAU]	Area%
	5.590	MM m	0.1540	10.3802	1.0532	0.9238
	8.274	BB	2.4633	1113.2488	35.8103	99.0762

tert-Butyl (S)-2-amino-3-(4-methoxyphenyl)-2-methylpropanoate (7g):

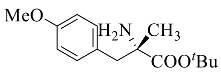

Colorless oil (41.5 mg, 78%); R_f= 0.25 (petroleum ether/ethyl acetate = 2:1); the enantiomeric excess was determined to be 98% by HPLC analysis on Daicel Chirapak IC-H column (hexane/isopropanol = 70/30, flow rate 1 mL/min, T = 30 ºC), UV 220 nm, t_R(major) 12.923 min, t_R(minor) 10.874 min; $[\alpha]_D^{25}$ = -39.77 (c=0.77, $CHCl_3$); **^{1}H NMR (600 MHz, $CDCl_3$)** δ 7.13 (d, J = 6.0 Hz, 2H), 6.81 (d, J = 6.0 Hz, 2H), 3.78 (s, 3H), 3.05 (d, J = 12.0 Hz, 1H), 2.71 (d, J = 12.0 Hz, 1H), 1.61 (s, 2H), 1.46 (s, 9H), 1.32 (s, 3 H); **^{13}C NMR (151 MHz, $CDCl_3$)** δ 176.43, 158.53, 131.16, 128.89, 113.60, 81.02, 58.81, 55.20, 45.52, 28.02, 26.91; **HRMS(ESI)** m/z: $[M+H]^+$ Calculated for $C_{15}H_{24}NO_3^+$ 266.1751; found 266.1746.

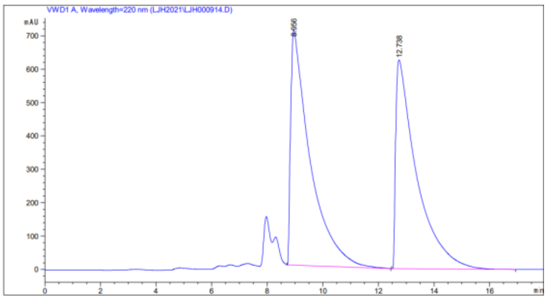

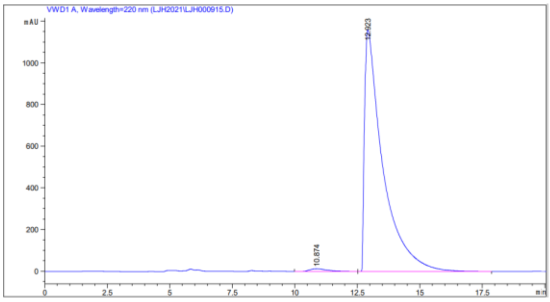

Peak #	RetTime [min]	Type	Width [min]	Area [mAU*s]	Height [mAU]	Area %
1	8.956	BB	0.6430	3.40685e4	704.35052	51.2186
2	12.738	BB	0.7176	3.24474e4	625.32318	48.7814

Peak #	RetTime [min]	Type	Width [min]	Area [mAU*s]	Height [mAU]	Area %
1	10.874	BB	0.7555	611.34070	12.53654	1.0272
2	12.923	BB	0.7182	5.89063e4	1159.16711	98.9728

tert-Butyl (S)-2-amino-2-methyl-3-(p-tolyl)propanoate (7h):

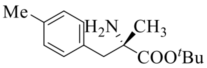

Colorless oil (39.1 mg, 78%); R_f= 0.26 (petroleum ether/ethyl acetate = 2:1); the enantiomeric excess was determined to be 97% by HPLC analysis on Daicel Chirapak IC-H column (hexane/isopropanol = 90/10, flow rate 1 mL/min, T = 30 ºC), UV 220 nm, t_R(major) 17.710 min, t_R(minor) 10.008 min; $[\alpha]_D^{25}$ = -33.70 (c=0.55, $CHCl_3$); **^{1}H NMR (600 MHz, $CDCl_3$)** δ 7.09 (q, *J* = 12.0 Hz, 4H), 3.07 (d, *J* = 12.0 Hz, 1H), 2.73 (d, *J* = 12.0 Hz, 1H), 2.31 (s, 3H), 1.68 (s, 2H), 1.46 (s, 9H), 1.33 (s, 3H); **^{13}C NMR (151 MHz, $CDCl_3$)** δ 176.40, 136.30, 133.69, 130.07, 128.88, 81.05, 58.76, 45.95, 28.02, 26.97, 21.02; **HRMS(ESI)** m/z: $[M+H]^+$ Calculated for $C_{15}H_{24}NO_2^+$ 250.1802; found 250.1796.

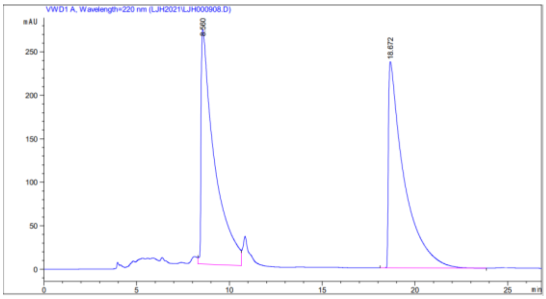

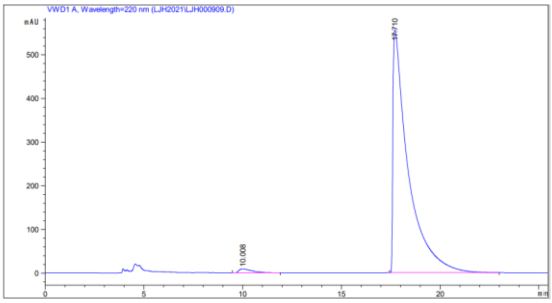

Peak #	RetTime [min]	Type	Width [min]	Area [mAU*s]	Height [mAU]	Area %
1	8.560	VV	0.6685	1.33944e4	269.61099	49.2445
2	18.672	BB	0.7766	1.38053e4	237.22800	50.7555

Peak #	RetTime [min]	Type	Width [min]	Area [mAU*s]	Height [mAU]	Area %
1	10.008	BB	0.6686	391.20352	8.51616	1.2982
2	17.710	BB	0.7264	2.97432e4	555.73517	98.7018

tert-Butyl (S)-2-amino-3-(4-(tert-butyl)phenyl)-2-methylpropanoate (7i):

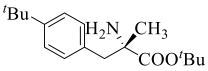

Colorless oil (48.7 mg, 83%); R_f= 0.28 (petroleum ether/ethyl acetate = 2:1); the enantiomeric excess was determined to be 91% by HPLC analysis on Daicel Chirapak IF-H column (hexane/isopropanol = 98/2, flow rate 1 mL/min, T = 30 ºC), UV 220 nm, t_R(major) 8.972 min, t_R(minor) 8.221 min; $[\alpha]_D^{25}$ = -39.13 (c=0.95, $CHCl_3$); **^{1}H NMR (600 MHz, $CDCl_3$)** δ 7.29 (d, *J* = 6.0 Hz, 2H), 7.14 (d, *J* = 6.0 Hz, 2H), 3.08 (d, *J* = 12.0 Hz, 1H),

2.74 (d, J = 12.0 Hz, 1H), 1.60 (s, 2H), 1.46 (s, 9H), 1.34 (s, 3H), 1.30 (s, 9H); **^{13}C NMR (151 MHz, CDCl$_3$)** δ 176.47, 149.55, 133.74, 129.88, 125.06, 81.00, 58.76, 45.89, 34.39, 31.37, 28.02, 27.05; **HRMS(ESI)** m/z: $[M+H]^+$ Calculated for $C_{18}H_{30}NO_2^+$ 292.2271; found 292.2267.

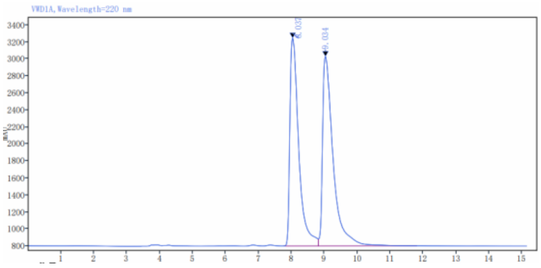

Peak	RetTime [min]	Type	Width[min]	Area[mAU*s]	Height[mAU]	Area%
	8.037	BV	1.0740	45634.8144	2440.9126	47.9217
	9.034	VB	2.9932	49593.0904	2223.7515	52.0783

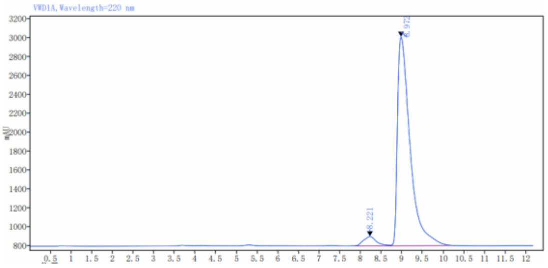

Peak	RetTime [min]	Type	Width[min]	Area[mAU*s]	Height[mAU]	Area%
	8.221	MM m	0.3117	2172.8525	99.1488	4.4692
	8.972	MM m	0.3176	46445.1865	2206.6559	95.5308

tert-Butyl (S)-3-([1,1'-biphenyl]-4-yl)-2-amino-2-methylpropanoate (7j):

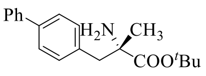

Colorless oil (54.3 mg, 85%); R_f = 0.25 (petroleum ether/ethyl acetate = 2:1); the enantiomeric excess was determined to be 98% by HPLC analysis on Daicel Chirapak OJ-H column (hexane/isopropanol = 98/2, flow rate 0.8 mL/min, T = 30 ºC), UV 220 nm, t_R(major) 13.665 min, t_R(minor) 13.079 min; $[\alpha]_D^{25}$ = -45.78 (c=1.13, CHCl$_3$); **^{1}H NMR (600 MHz, CDCl$_3$)** δ 7.57 (d, J = 6.0 Hz, 2H), 7.51 (d, J = 6.0 Hz, 2H), 7.42 (t, J = 6.0 Hz, 2H), 7.31 (m, 3H), 3.15 (d, J = 18.0 Hz, 1H), 2.81 (d, J = 18.0 Hz, 1H), 1.63 (s, 2H), 1.47 (s, 9H), 1.37 (s, 3H); **^{13}C NMR (151 MHz, CDCl$_3$)** δ 176.35, 140.92, 139.70, 136.00, 130.66, 128.75, 127.18, 127.02, 126.90, 81.18, 58.84, 46.08, 28.05, 27.06; **HRMS(ESI)** m/z: $[M+H]^+$ Calculated for $C_{20}H_{26}NO_2^+$ 312.1958; found 312.1955.

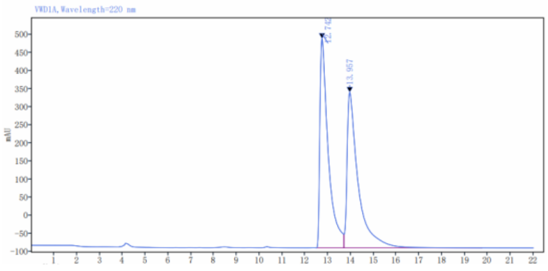

Peak	RetTime [min]	Type	Width[min]	Area[mAU*s]	Height[mAU]	Area%
	12.742	VV	1.2167	14754.7306	579.5885	50.2461
	13.957	VB	6.0710	14610.2012	430.6353	49.7539

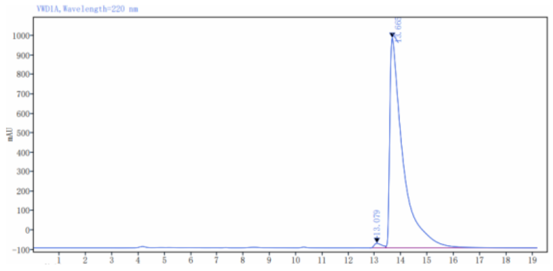

Peak	RetTime [min]	Type	Width[min]	Area[mAU*s]	Height[mAU]	Area%
	13.079	BV	0.6135	471.4516	23.1780	1.2357
	13.665	VBA	5.7599	37679.8230	1079.0467	98.7643

tert-butyl (S)-2-(((benzyloxy)carbonyl)amino)-3-(4-fluorophenyl)-2-methylpropanoate (N-Cbz-7k):

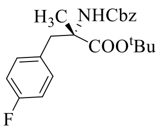

Colorless oil (58.1 mg, 75%); R_f = 0.38 (petroleum ether/ethyl acetate = 5:1); the enantiomeric excess was determined to be 98% by HPLC analysis on Daicel Chirapak OD-H column (hexane/isopropanol = 80/20, flow rate 1 mL/min, T = 30 ºC), UV 254 nm, t_R(major) 9.726 min, t_R(minor) 6.757 min; $[\alpha]_D^{25}$ = +81.18 (c=0.51, $CHCl_3$); **^{1}H NMR (600 MHz, CDCl$_3$)** δ 7.40 – 7.32 (m, 5H), 7.00 – 6.93 (m, 2H), 6.84 (t, *J* = 6.0 Hz, 2H), 5.56 (s, 1H), 5.19 (d, *J* = 12.0 Hz, 1H), 5.05 (d, *J* = 12.0 Hz, 1H), 3.44 (d, *J* = 12.0 Hz, 1H), 3.09 (d, *J* = 12.0 Hz, 1H), 1.60 (s, 3H), 1.44 (s, 9H); **^{13}C NMR (151 MHz, CDCl$_3$)** δ 172.49, 162.66, 161.04, 154.54, 136.77, 132.29, 131.47, 131.42, 128.51, 128.20, 128.13, 114.91, 114.77, 82.61, 66.25, 60.87, 40.23, 27.90, 24.08; **HRMS(ESI)** m/z: $[M+H]^+$ Calculated for $C_{22}H_{27}FNO_4^+$ 388.1919; found 388.1915.

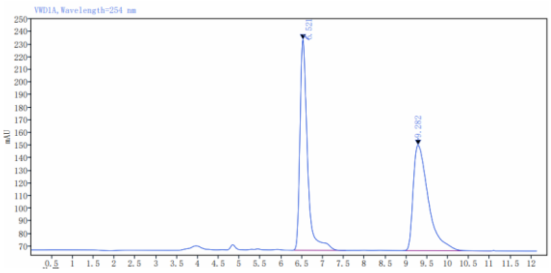

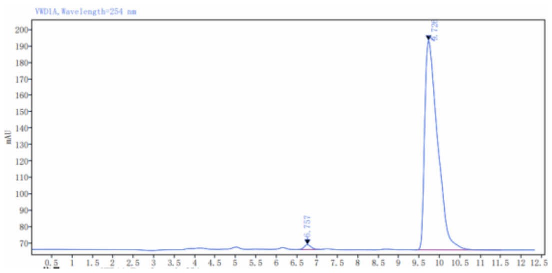

Peak	RetTime [min]	Type	Width[min]	Area[mAU*s]	Height[mAU]	Area%
	6.521	BB	1.2993	2083.4335	166.7401	49.7783
	9.282	BB	2.1233	2101.9902	83.5587	50.2217

Peak	RetTime [min]	Type	Width[min]	Area[mAU*s]	Height[mAU]	Area%
	6.757	BB	0.5550	33.9240	2.8523	1.1946
	9.726	BB	2.1133	2805.7710	127.4937	98.8054

tert-Butyl (S)-2-amino-3-(benzo[d][1,3]dioxol-5-yl)-2-methylpropanoate (7l):

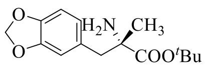

Colorless oil (44.7 mg, 80%); R_f = 0.26 (petroleum ether/ethyl acetate = 2:1); the enantiomeric excess was determined to be 94% by HPLC analysis on Daicel Chirapak IC-H column (hexane/isopropanol = 70/30, flow rate 1 mL/min, T = 30 ºC), UV 220 nm, t_R(major) 22.132 min, t_R(minor) 13.084 min; $[\alpha]_D^{25}$ = -26.53 (c=0.77, $CHCl_3$); **^{1}H NMR (600 MHz, CDCl$_3$)** δ 6.75 – 6.67 (m, 2H), 6.67 (d, *J* = 6.0 Hz, 1H), 5.92 (s, 2H), 3.03 (d, *J* = 12.0 Hz, 1H), 2.68 (d, *J* = 12.0 Hz, 1H), 1.68 (s, 2H), 1.47 (s, 9H), 1.32 (s, 3H); **^{13}C NMR (151 MHz, CDCl$_3$)** δ 176.34, 147.41, 146.43, 130.51, 123.24, 110.47, 107.99, 100.85, 81.16, 58.82, 46.06, 28.03, 26.94; **HRMS(ESI)** m/z: $[M+H]^+$ Calculated for $C_{15}H_{22}NO_4^+$ 280.1543; found 280.1539.

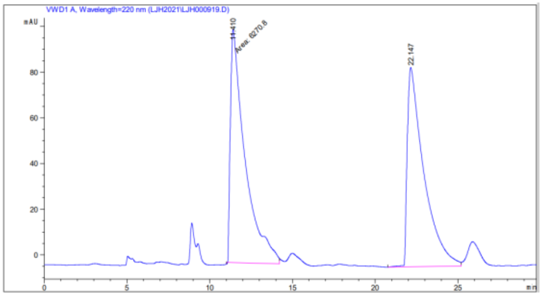

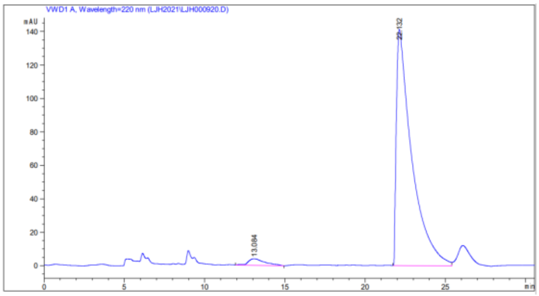

Peak #	RetTime [min]	Type	Width [min]	Area [mAU*s]	Height [mAU]	Area %
1	11.410	MM	1.0243	6270.79688	102.03079	50.7736
2	22.147	BV	0.9821	6079.71240	87.24239	49.2264

Peak #	RetTime [min]	Type	Width [min]	Area [mAU*s]	Height [mAU]	Area %
1	13.084	BB	0.9768	277.93622	3.88140	2.8670
2	22.132	BV	0.9404	9416.54102	141.10281	97.1330

tert-Butyl (S)-2-amino-3-(3-fluoro-4-methoxyphenyl)-2-methylpropanoate (7m):

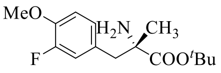

A pale yellow oil (45.3 mg, 77%); R_f= 0.25 (petroleum ether/ethyl acetate = 2:1); the enantiomeric excess was determined to be 95% by HPLC analysis on Daicel Chirapak IC-H column (hexane/isopropanol = 90/10, flow rate 1 mL/min, T = 30 ºC), UV 220 nm, t_R(major) 25.000 min, t_R(minor) 21.698 min; $[\alpha]_D^{25}$ = -38.58 (c=0.90, $CHCl_3$); **^{1}H NMR (600 MHz, $CDCl_3$)** δ 6.97 (d, J = 12.0 Hz, 1H), 6.93 (d, J = 6.0 Hz, 1H), 6.87 (t, J = 6.0 Hz, 1H), 3.86 (s, 3H), 3.03 (d, J = 12.0 Hz, 1H), 2.69 (d, J = 12.0 Hz, 1H), 1.61 (s, 2H), 1.46 (s, 9H), 1.33 (s, 3H); **^{13}C NMR (151 MHz, $CDCl_3$)** δ 176.20, 152.78, 151.15, 146.52, 146.45, 129.95, 129.91, 125.85, 125.82, 117.86, 117.74, 113.13, 81.26, 58.76, 56.26, 45.41, 28.00, 26.90; **HRMS(ESI)** m/z: $[M+H]^+$ Calculated for $C_{15}H_{23}FNO_3^+$ 284.1656; found 284.1652.

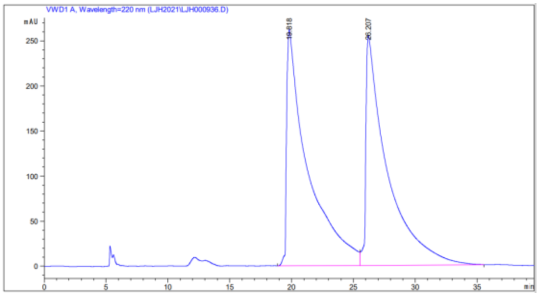

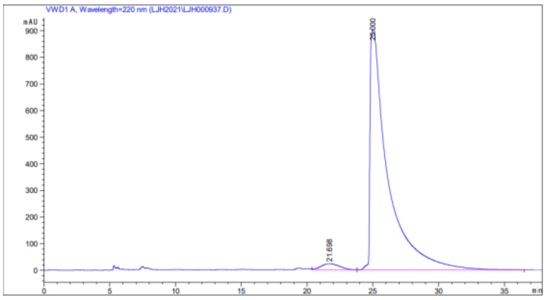

Peak #	RetTime [min]	Type	Width [min]	Area [mAU*s]	Height [mAU]	Area %
1	19.818	BV	1.5381	3.08886e4	262.10989	50.3687
2	26.207	VB	1.5474	3.04364e4	254.94171	49.6313

Peak #	RetTime [min]	Type	Width [min]	Area [mAU*s]	Height [mAU]	Area %
1	21.698	VB	1.4042	2258.51758	23.07750	2.6125
2	25.000	BB	1.2753	8.41911e4	900.99609	97.3875

tert-Butyl (S)-2-amino-2-methyl-3-(thiophen-3-yl)propanoate (7n):

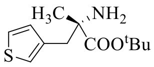

Colorless oil (41.0 mg, 80%); R_f= 0.27 (petroleum ether/ethyl acetate = 2:1); the enantiomeric excess was determined to be 96% by HPLC analysis on Daicel Chirapak IC-H column (hexane/isopropanol = 90/10, flow rate 1 mL/min, T = 30 ºC), UV 220 nm, t_R(major) 16.970 min, t_R(minor) 15.235 min; $[\alpha]_D^{25}$ = -30.25 (c=0.73, $CHCl_3$); **^{1}H NMR (600**

MHz, $CDCl_3$) δ 7.24 (m, 1H), 7.05 (m, 1H), 6.97 (d, J = 6.0 Hz, 1H), 3.14 (d, J = 12.0 Hz, 1H), 2.81 (d, J = 12.0 Hz, 1H), 1.66 (s, 2H), 1.46 (s, 9H), 1.34 (s, 3H); **^{13}C NMR (151 MHz, $CDCl_3$)** δ 176.40, 137.20, 129.35, 125.19, 123.10, 81.09, 58.54, 40.80, 27.99, 27.04; **HRMS(ESI)** m/z: $[M+H]^+$ Calculated for $C_{12}H_{20}NO_2S^+$ 242.1209; found 242.1204.

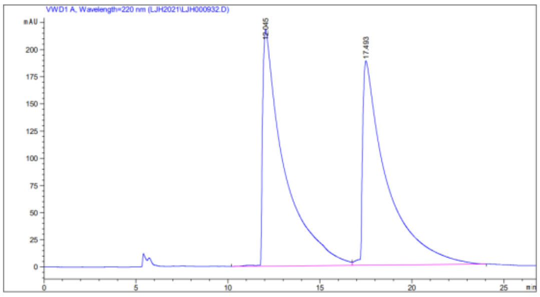

Peak #	RetTime [min]	Type	Width [min]	Area [mAU*s]	Height [mAU]	Area %
1	12.045	BV	1.0872	1.77323e4	217.71571	50.9996
2	17.493	VB	1.2058	1.70372e4	188.02979	49.0004

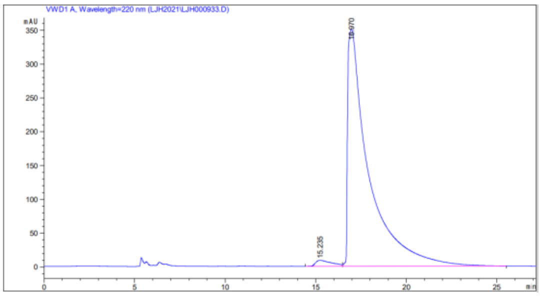

Peak #	RetTime [min]	Type	Width [min]	Area [mAU*s]	Height [mAU]	Area %
1	15.235	BV	0.8718	542.94012	8.88558	1.8811
2	16.970	VB	1.0999	2.83206e4	350.05414	98.1189

tert-Butyl (S)-2-amino-2-benzylbutanoate (7o):

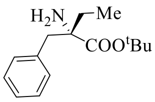

Colorless oil (33.9 mg, 68%); R_f= 0.28 (petroleum ether/ethyl acetate = 5:1); the enantiomeric excess was determined to be 97% by HPLC analysis on Daicel Chirapak OJ-H column (hexane/isopropanol = 95/5, flow rate 0.8 mL/min, T = 30 ºC), UV 220 nm, t_R(major) 8.928 min, t_R(minor) 7.106 min; $[\alpha]_D^{25}$ = -42.16 (c=0.67, $CHCl_3$); **^{1}H NMR (600 MHz, $CDCl_3$)** δ 7.29 – 7.25 (m, 2H), 7.22 (m, 3H), 3.16 (d, J = 12.0 Hz, 1H), 2.73 (d, J = 12.0 Hz, 1H), 1.92 (m, 1H), 1.58 (m, 3H), 1.46 (s, 9H), 0.90 (t, J = 6.0 Hz, 3H); **^{13}C NMR (151 MHz, $CDCl_3$)** δ 175.68, 136.80, 130.23, 128.19, 126.77, 81.17, 62.26, 45.58, 33.63, 28.08, 8.20; **HRMS(ESI)** m/z: $[M+H]^+$ Calculated for $C_{15}H_{24}NO_2^+$ 250.1802; found 250.1797.

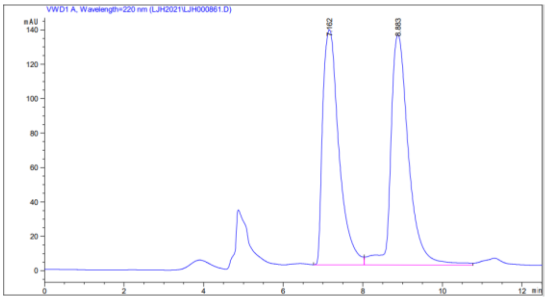

Peak #	RetTime [min]	Type	Width [min]	Area [mAU*s]	Height [mAU]	Area %
1	7.162	BV	0.4382	3867.59033	136.58496	48.1315
2	8.883	VV	0.4743	4167.87939	133.77834	51.8685

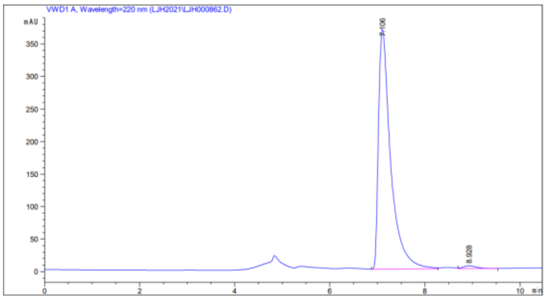

Peak #	RetTime [min]	Type	Width [min]	Area [mAU*s]	Height [mAU]	Area %
1	7.106	BV	0.2624	6526.06543	367.74921	98.7104
2	8.928	VB	0.2970	85.26232	4.32532	1.2896

tert-Butyl (R)-2-amino-2-benzyl-4-(methylthio)butanoate (7p):

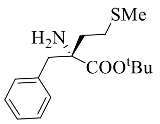

Colorless oil (45.7 mg, 77%); R_f = 0.25 (petroleum ether/ethyl acetate = 5:1); the enantiomeric excess was determined to be 98% by HPLC analysis on Daicel Chirapak IC-H column (hexane/isopropanol = 90/10, flow rate 1 mL/min, T = 30 ºC), UV 220 nm, t_R(major) 15.699 min, t_R(minor) 9.551 min; $[\alpha]_D^{25}$ = -21.00 (c=0.69, $CHCl_3$); **^{1}H NMR (600 MHz, $CDCl_3$)** δ 7.30 – 7.26 (m, 2H), 7.24 (m, 1H), 7.20 (d, *J* = 6.0 Hz, 2H), 3.14 (d, *J* = 12.0 Hz, 1H), 2.76 (d, *J* = 12.0 Hz, 1H), 2.59 (m, 1H), 2.41 (m, 1H), 2.16 (m, 1H), 2.11 (s, 3H), 1.84 (m, 1H), 1.57 (s, 2H), 1.46 (s, 9H); **^{13}C NMR (151 MHz, $CDCl_3$)** δ 175.01, 136.11, 130.25, 128.30, 127.00, 81.66, 61.87, 45.91, 40.21, 28.82, 28.07, 15.57; **HRMS(ESI)** m/z: $[M+H]^+$ Calculated for $C_{16}H_{26}NO_2S^+$ 296.1679; found 296.1675.

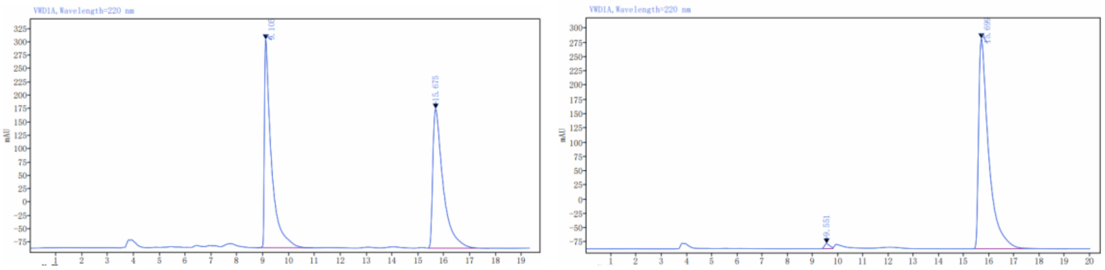

Peak	RetTime [min]	Type	Width[min]	Area[mAU^s]	Height[mAU]	Area%
	9.105	BB	2.1700	7284.4198	389.4054	50.2018
	15.675	VB	2.3831	7225.8661	260.7593	49.7982

Peak	RetTime [min]	Type	Width[min]	Area[mAU^s]	Height[mAU]	Area%
	9.551	BV	0.5270	125.4298	8.3960	1.2074
	15.699	BB	3.8033	10262.7294	368.0089	98.7926

tert-Butyl (S)-2-amino-2-benzylpent-4-enoate (7q):

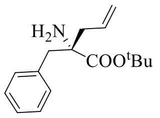

Colorless oil (41.0 mg, 79%); R_f = 0.30 (petroleum ether/ethyl acetate = 5:1); the enantiomeric excess was determined to be 97% by HPLC analysis on Daicel Chirapak IC-H column (hexane/isopropanol = 90/10, flow rate 1 mL/min, T = 30 ºC), UV 220 nm, t_R(major) 10.725 min, t_R(minor) 5.746 min; $[\alpha]_D^{25}$ = +24.39 (c=0.80, $CHCl_3$); **^{1}H NMR (600 MHz, $CDCl_3$)** δ 7.38 – 7.15 (m, 5H), 5.91 – 5.57 (m, 1H), 5.29 – 5.04 (m, 2H), 3.16 (d, *J* = 12.0 Hz, 1H), 2.76 (d, *J* = 12.0 Hz, 1H), 2.68 (dd, *J* = 12.0, 6.0 Hz, 1H), 2.27 (dd, *J* = 12.0, 6.0 Hz, 1H), 1.60 (s, 2H), 1.45 (s, 9H); **^{13}C NMR (151 MHz, $CDCl_3$)** δ 175.22, 136.50, 132.68, 130.24, 128.20, 126.86, 119.46, 81.42, 61.44, 45.70, 45.02, 28.09; **HRMS(ESI)** m/z: $[M+H]^+$ Calculated for $C_{16}H_{24}NO_2^+$ 262.1802; found 262.1796.

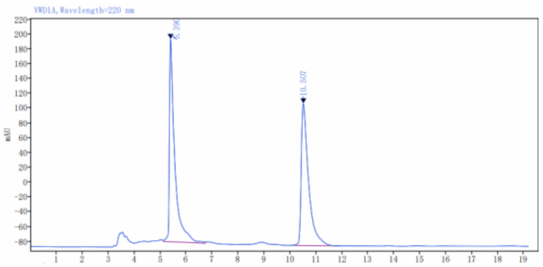

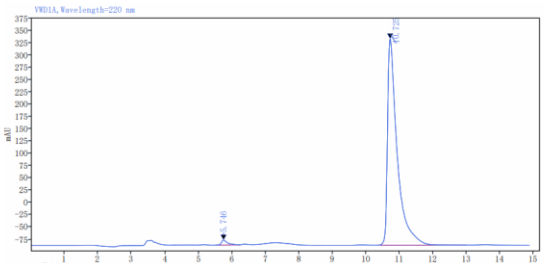

Peak	RetTime [min]	Type	Width[min]	Area[mAU^s]	Height[mAU]	Area%
	5.390	VV	1.6200	3914.0950	273.5172	51.1310
	10.507	BB	1.9100	3740.9365	191.5328	48.8690

Peak	RetTime [min]	Type	Width[min]	Area[mAU^s]	Height[mAU]	Area%
	5.746	BB	0.7646	127.8358	10.1312	1.4917
	10.725	BB	2.6783	8442.1905	420.5642	98.5083

tert-Butyl (R)-2-amino-2,3-diphenylpropanoate (7r):

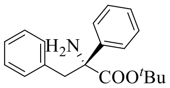

White solid (53.6 mg, 89%); m.p. = 66-68 ºC; R_f = 0.28 (petroleum ether/ethyl acetate = 5:1); the enantiomeric excess was determined to be 84% by HPLC analysis on Daicel Chirapak IC-H column (hexane/isopropanol = 90/10, flow rate 1 mL/min, T = 30 ºC), UV 220 nm, t_R(major) 14.345 min, t_R(minor) 6.554 min; $[\alpha]_D^{25}$ = +19.97 (c=0.82, $CHCl_3$); **^{1}H NMR (600 MHz, $CDCl_3$)** δ 7.61 – 7.53 (m, 2H), 7.35 (t, J = 6.0 Hz, 2H), 7.28 (t, J = 6.0 Hz, 1H), 7.26 – 7.20 (m, 3H), 7.18 – 7.14 (m, 2H), 3.61 (d, J = 12.0 Hz, 1H), 3.11 (d, J = 12.0 Hz, 1H), 1.85 (s, 2H), 1.42 (s, 9H); **^{13}C NMR (151 MHz, $CDCl_3$)** δ 174.11, 143.58, 136.58, 130.62, 128.24, 128.15, 127.32, 126.87, 125.52, 81.84, 64.49, 45.68, 27.91; **HRMS(ESI)** m/z: $[M+H]^+$ Calculated for $C_{19}H_{24}NO_2^+$ 298.1802; found 298.1797.

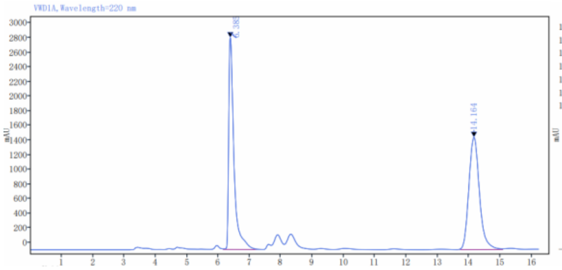

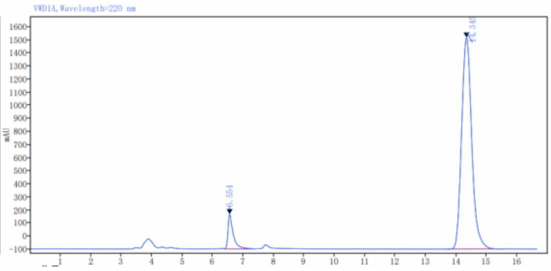

Peak	RetTime [min]	Type	Width[min]	Area[mAU^s]	Height[mAU]	Area%
	6.385	VB	1.2643	33819.3334	2892.4361	48.4718
	14.164	BV	1.4855	35951.8403	1532.2110	51.5282

Peak	RetTime [min]	Type	Width[min]	Area[mAU^s]	Height[mAU]	Area%
	6.554	VV	1.0445	3182.0533	263.4950	7.8375
	14.345	BB	1.8606	37418.2136	1612.6243	92.1625

tert-Butyl (R)-2-amino-3-phenyl-2-(p-tolyl)propanoate (7s):

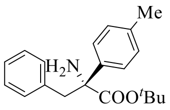

White solid (51.4 mg, 82%); m.p. = 61-62 ºC; R_f = 0.30 (petroleum ether/ethyl acetate = 5:1); the enantiomeric excess was determined to be 94% by HPLC analysis on Daicel Chirapak IC-H column (hexane/isopropanol = 90/10, flow rate 1 mL/min, T = 30 ºC), UV 220 nm, t_R(major) 19.857 min, t_R(minor) 10.308 min; $[\alpha]_D^{25}$ = +30.33 (c=0.64, $CHCl_3$); **^{1}H NMR (600 MHz, $CDCl_3$)** δ 7.46 (d, J = 12.0 Hz, 2H), 7.29 – 7.20 (m, 3H), 7.16 (m, 4H), 3.59 (d,

J = 12.0 Hz, 1H), 3.08 (d, J = 12.0 Hz, 1H), 2.34 (s, 3H), 1.81 (s, 2H), 1.42 (s, 9H); **^{13}C NMR (151 MHz, CDCl$_3$)** δ 174.24, 140.68, 136.90, 136.72, 130.63, 128.94, 128.13, 126.82, 125.41, 81.72, 64.28, 45.75, 27.92, 20.99; **HRMS(ESI)** m/z: [M+H]$^+$ Calculated for $C_{20}H_{26}NO_2^+$ 312.1958; found 312.1955.

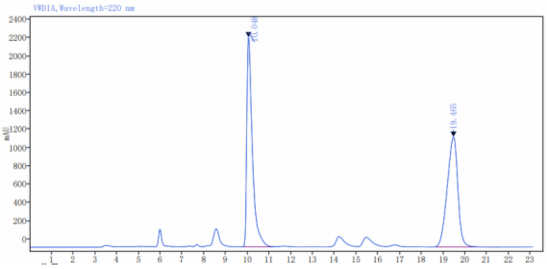

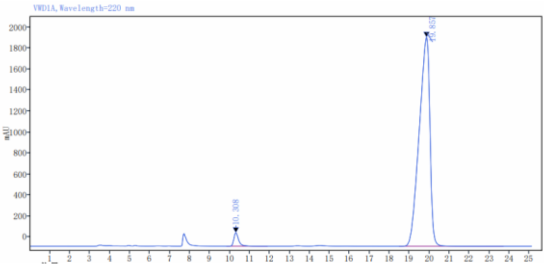

Peak	RetTime [min]	Type	Width[min]	Area[mAU*s]	Height[mAU]	Area%
	10.048	BV	1.5339	40815.8339	2286.8855	50.0824
	19.465	BB	3.1633	40681.5138	1202.3982	49.9176

Peak	RetTime [min]	Type	Width[min]	Area[mAU*s]	Height[mAU]	Area%
	10.308	BB	2.0667	2166.2130	127.5826	2.8390
	19.857	BB	5.2300	74135.9852	1989.1116	97.1610

4 . Determination of the absolute configuration of 5a

The absolute configuration of compound **5a** was established by comparing its optical rotation value with the literature data:

(*S*)-product (**5a**) in this work	(*S*)-product in literature[4]
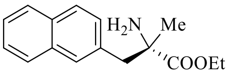 ethyl (*S*)-2-amino-2-methyl-3-(naphthalen-2-yl)propanoate	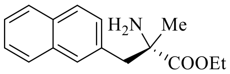 ethyl (*S*)-2-amino-2-methyl-3-(naphthalen-2-yl)propanoate
$[\alpha]_D^{25}$=-37.79(c 0.85, CHCl$_3$)	$[\alpha]_D^{25}$=-32.90(c 1.00, CHCl$_3$)

5. Supplementary methods for the derivatization of products

5.1 The synthesis of compounds 8a and 8b

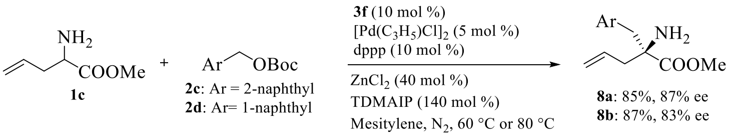

To a 10 mL vial charged with $[Pd(C_3H_5)Cl]_2$ (3.6 mg, 0.01 mmol) and dppp (8.2 mg, 0.02 mmol) was added 0.5 mL mesitylene, and the mixture was stirred under nitrogen atmosphere at room temperature for 30 min. Then, **1c** (0.3 mmol), **2c** or **2d** (0.2 mmol), chiral aldehyde **3f** (7.7 mg, 0.02 mmol), $ZnCl_2$ (10.9 mg, 0.08 mmol) and TDMAIP (50.9 uL, 0.28 mmol) were added. The mixture was continuously stirred at 60 ºC under nitrogen atmosphere. After the reaction completed, the mixture solvent was removed and the residue was purified with flash chromatography column on silica gel (eluent: petroleum ether/ ethyl acetate/ triethylamine =250/50/3).

Methyl (S)-2-amino-2-(naphthalen-2-ylmethyl)pent-4-enoat (8a):

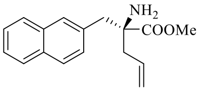

Colorless oil (45.7 mg, 85%); R_f = 0.24 (petroleum ether/ethyl acetate = 5:1); the enantiomeric excess was determined to be 87% by HPLC analysis on Daicel Chirapak AD-H column (hexane/isopropanol = 90/10, flow rate 1.0 mL/min, T = 30 °C), UV 254 nm, t_R(major) 9.912 min, t_R(minor) 7.804 min; $[\alpha]_D^{25}$ = -9.22 (c=0.64, $CHCl_3$); **^{1}H NMR (400 MHz, $CDCl_3$)** δ 7.86 – 7.70 (m, 3H), 7.63 (s, 1H), 7.51 – 7.40 (m, 2H), 7.33 – 7.20 (m, 1H), 5.73 (m, 1H), 5.25 – 5.07 (m, 2H), 3.70 (s, 3H), 3.35 (d, *J* = 13.2 Hz, 1H), 2.95 (d, *J* = 13.2 Hz, 1H), 2.76 (dd, *J* = 13.6, 6.4 Hz, 1H), 2.37 (dd, *J* = 13.2, 8.4 Hz, 1H), 1.66 (s, 2H); **^{13}C NMR (101 MHz, $CDCl_3$)** δ 176.56, 133.81, 133.41, 132.54, 132.50, 128.76, 128.05, 127.96, 127.66, 127.60, 126.07, 125.68, 119.74, 62.06, 52.00, 46.05, 44.60; **HRMS(ESI)** m/z: $[M+H]^+$ Calculated for $C_{17}H_{20}NO_2^+$ 270.1489; found 270.1482.

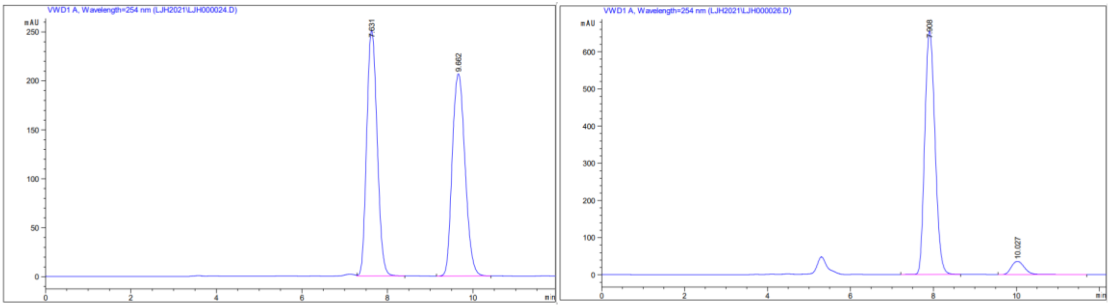

Peak #	RetTime [min]	Type	Width [min]	Area [mAU*s]	Height [mAU]	Area %
1	7.631	VB	0.2714	4267.43701	250.76236	49.7719
2	9.662	BB	0.3335	4306.54688	206.64680	50.2281

Peak #	RetTime [min]	Type	Width [min]	Area [mAU*s]	Height [mAU]	Area %
1	7.804	VV	0.2740	1.43110e4	836.10303	93.2562
2	9.912	BB	0.3456	1034.90405	47.58517	6.7438

Methyl (S)-2-amino-2-(naphthalen-1-ylmethyl)pent-4-enoa (8b):

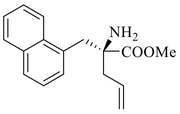

Colorless oil (46.9 mg, 87%); R_f = 0.23 (petroleum ether/ethyl acetate = 5:1); the enantiomeric excess was determined to be 83% by HPLC analysis on Daicel Chirapak OD-H column (hexane/isopropanol = 90/10, flow rate 1.0 mL/min, T = 30 °C), UV 254 nm, t_R(major) 7.043 min, t_R(minor) 6.453 min; $[\alpha]_D^{25}$ = +10.25 (c=0.45, $CHCl_3$); **^{1}H NMR (400 MHz, $CDCl_3$)** δ 8.16 (d, *J* = 8.4 Hz, 1H), 7.79 (d, *J* = 8.0 Hz, 1H), 7.74 (d, *J* = 8.0 Hz, 1H), 7.62 – 7.27 (m, 4H), 5.91 – 5.56 (m, 1H), 5.19 (m, 2H), 3.56 (s, 3H), 3.52 (d, *J* = 14.0 Hz, 1H), 3.41 (d, *J* = 14.0 Hz, 1H), 2.86 (dd, *J* = 13.2, 6.4 Hz, 1H), 2.43 (dd, *J* = 13.2, 8.4 Hz, 1H), 1.62 (s, 2H); **^{13}C NMR (101 MHz, $CDCl_3$)** δ 176.66, 133.96, 132.93, 132.75, 132.71, 128.70, 128.28, 127.79, 125.84, 125.55, 125.21, 124.43, 119.82, 62.58, 51.96, 44.74, 41.69; **HRMS(ESI)** m/z: $[M+H]^+$ Calculated for $C_{17}H_{20}NO_2^+$ 270.1489; found 270.1483.

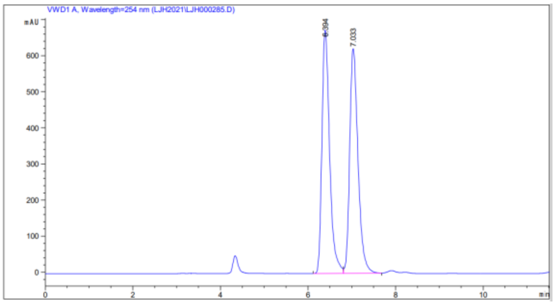

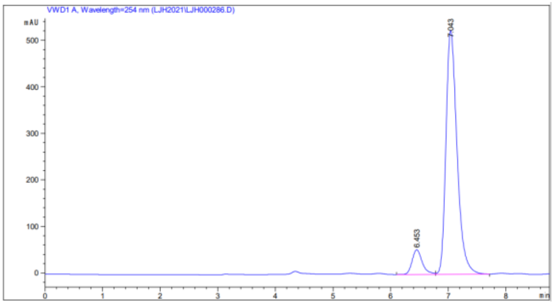

Peak #	RetTime [min]	Type	Width [min]	Area [mAU*s]	Height [mAU]	Area %
1	6.394	BV	0.1872	8206.85645	674.13824	49.9940
2	7.033	VB	0.2020	8208.81445	622.25403	50.0060

Peak #	RetTime [min]	Type	Width [min]	Area [mAU*s]	Height [mAU]	Area %
1	6.453	BV	0.1870	650.15405	52.94361	8.5299
2	7.043	VB	0.2049	6971.93799	523.77502	91.4701

5.2 The synthesis of compound 9a and 9b

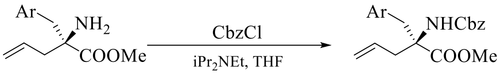

8a: Ar = 2-naphthyl
8b: Ar = 1-naphthyl

9a: 98%, 87% ee
9b: 96%, 84% ee

To a solution of **8a** or **8b** (44.2 mg, 0.16 mmol) in THF (1 mL) was added $^{i}Pr_2NEt$ (97.6 uL, 0.59 mmol) and CbzCl (67.7 uL, 0.49 mmol).The resulting mixture was stirred for 3h, and then the precipitation was removed by filtration. The filtrate was diluted with ether, washed with 1N hydrochloric acid, sat. aqueous $NaHCO_3$, and brine. The organic layer was separated and dried over Na_2SO_4, and concentrated in vacuo. The residue was purified by flash chromatography column on silica gel (eluent: petroleum ether / ethyl acetate =10/1).

Methyl (S)-2-(((benzyloxy)carbonyl)amino)-2-(naphthalen-2-ylmethyl)pent-4-enoate (9a):

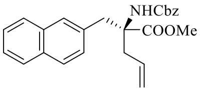

Colorless oil (56.1 mg, 98%); R_f= 0.37 (petroleum ether/ethyl acetate = 5:1); the enantiomeric excess was determined to be 87% by HPLC analysis on Daicel Chirapak AD-H column (hexane/isopropanol = 80/20, flow rate 1.0 mL/min, T = 30 ºC), UV 254 nm, t_R(major) 7.661 min, t_R(minor) 6.624 min; $[\alpha]_D^{25}$ = +56.11 (c=1.32, $CHCl_3$); **^{1}H NMR (400 MHz, $CDCl_3$)** δ 7.76 (m, 1H), 7.66 – 7.57 (m, 2H), 7.52 – 7.31 (m, 8H), 7.06 (m, 1H), 5.64 (m, 2H), 5.23 (d, *J* = 12.0 Hz, 1H), 5.14 – 5.06 (m, 3H), 3.79 (d, *J* = 13.6 Hz, 1H), 3.75 (s, 3H), 3.29 (d, *J* = 13.6 Hz, 1H), 3.25 (s, 1H), 2.67 (dd, *J* = 14.0, 7.2 Hz, 1H); **^{13}C NMR (101 MHz, $CDCl_3$)** δ 172.73, 154.45, 136.79, 133.61, 133.38, 132.45, 132.11, 128.63, 128.57, 128.15, 127.88, 127.79, 127.70, 127.58, 125.93, 125.64, 119.26, 66.35, 65.32, 52.61, 40.89, 40.06; **HRMS(ESI)** m/z: $[M+H]^+$ Calculated for $C_{25}H_{26}NO_4^+$ 404.1856; found 404.1857.

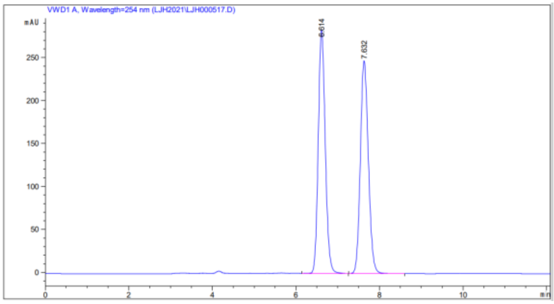

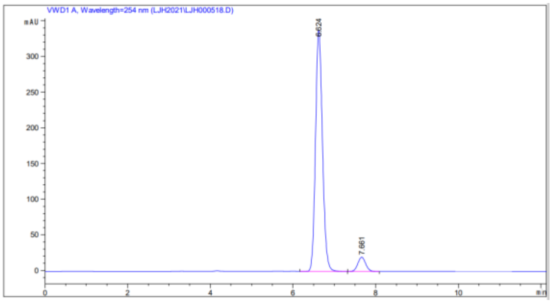

Peak #	RetTime [min]	Type	Width [min]	Area [mAU*s]	Height [mAU]	Area %
1	6.614	BB	0.1814	3302.71851	282.97000	50.0304
2	7.632	BB	0.2065	3298.70728	247.59250	49.9696

Peak #	RetTime [min]	Type	Width [min]	Area [mAU*s]	Height [mAU]	Area %
1	6.624	BB	0.1812	3940.73438	337.92651	93.6916
2	7.661	BB	0.2068	265.33380	20.06046	6.3084

Methyl (S)-2-(((benzyloxy)carbonyl)amino)-2-(naphthalen-1-ylmethyl)pent-4-enoate (9b):

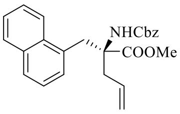

Colorless oil (62.1 mg, 96%); R_f = 0.36 (petroleum ether/ethyl acetate = 5:1); the enantiomeric excess was determined to be 84% by HPLC analysis on Daicel Chirapak AD-H column (hexane/isopropanol = 80/20, flow rate 1.0 mL/min, T = 30 °C), UV 254 nm, t_R(major) 7.024 min, t_R(minor) 6.297 min; $[\alpha]_D^{25}$ = +63.85 (c=1.21, $CHCl_3$); **^{1}H NMR (400 MHz, $CDCl_3$)** δ 8.06 (d, *J* = 8.0 Hz, 1H), 7.86 – 7.77 (m, 1H), 7.71 (d, *J* = 8.0 Hz, 1H), 7.45 – 7.25 (m, 8H), 7.15 (d, *J* = 6.8 Hz, 1H), 5.80 – 5.51 (m, 2H), 5.37 – 4.96 (m, 4H), 4.00 (d, *J* = 14.0 Hz, 1H), 3.64 (d, *J* = 14.0 Hz, 1H), 3.57 (s, 3H), 3.44 (dd, *J* = 14.0, 7.6 Hz, 1H), 2.80 (dd, *J* = 13.6, 7.2 Hz, 1H); **^{13}C NMR (101 MHz, $CDCl_3$)** δ 172.74, 154.54, 136.66, 133.85, 132.80, 132.40, 132.26, 128.74, 128.51, 128.27, 128.16, 128.11, 127.79, 125.72, 125.43, 125.14, 123.83, 119.26, 66.38, 65.26, 52.49, 39.74, 37.24;**HRMS(ESI)** m/z: $[M+H]^+$ Calculated for $C_{25}H_{26}NO_4^+$ 404.1856; found 404.1857.

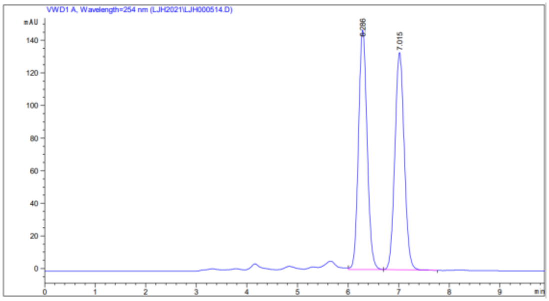

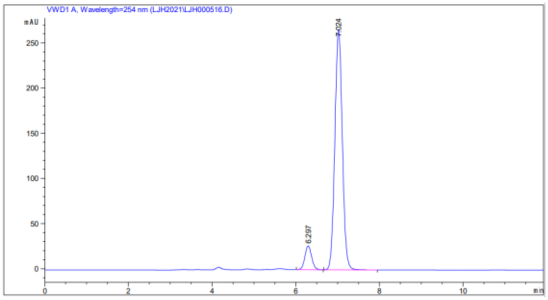

Peak #	RetTime [min]	Type	Width [min]	Area [mAU*s]	Height [mAU]	Area %
1	6.286	VB	0.1842	1731.79529	146.90143	50.0873
2	7.015	BB	0.2021	1725.75891	133.25005	49.9127

Peak #	RetTime [min]	Type	Width [min]	Area [mAU*s]	Height [mAU]	Area %
1	6.297	BB	0.1722	292.89764	26.30422	8.1285
2	7.024	BB	0.1935	3310.43896	265.70010	91.8715

5.3 The synthesis of compound 10a and 10b

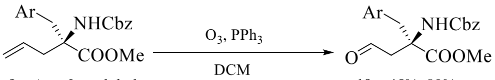

9a: Ar = 2-naphthyl
9b: Ar = 1-naphthyl

10a: 45%, 89% ee
10b: 47%, 86% ee

A solution of **9a** or **9b** (123 mg, 0.3 mmol) in CH_2Cl_2 (5.00 mL) was cooled to - 78 °C, ozone was then bubbled through the solution for about 5 minutes until the reaction mixture became blue. The O_3 generator was turned off and N_2 was bubbled through the reaction solution to remove the unreacted O_3. A solution of PPh_3 (80.1 mg, 0.3 mmol) in CH_2Cl_2 (1 mL) was then added to the reaction mixture at –78 °C. After warming to room temperature the solvent was evaporated in vacuo. The residue was purified by flash chromatography column on silica gel (eluent: petroleum ether/ ethyl acetate =10/1).

Methyl (R)-2-(((benzyloxy)carbonyl)amino)-2-(naphthalen-2-ylmethyl)-4-oxobutanoate (10 a):

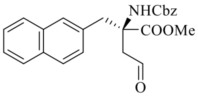

A pale yellow oil (86.6 mg, 45%); R_f= 0.31 (petroleum ether/ethyl acetate = 2:1); the enantiomeric excess was determined to be 89% by HPLC analysis on Daicel Chirapak AD-H column (hexane/isopropanol = 80/20, flow rate 1.0 mL/min, T = 30 ºC), UV 254 nm, t_R(major) 21.101 min, t_R(minor) 11.484 min; $[\alpha]_D^{25}$ = +95.51 (c=0.51, $CHCl_3$); **^{1}H NMR (400 MHz, $CDCl_3$)** δ 9.67 (s, 1H), 7.77 (m, 1H), 7.68 – 7.55 (m, 2H), 7.46 – 7.33 (m, 8H), 7.01 (m, 1H), 5.85 (s, 1H), 5.25 (d, *J* = 12.8 Hz, 1H), 5.01 (d, *J* = 12.4 Hz, 1H), 3.96 (d, *J* = 13.6 Hz, 1H), 3.77 (d, *J* = 13.6 Hz, 1H), 3.72 (s, 3H), 3.24 – 3.03 (m, 2H); **^{13}C NMR (101 MHz, $CDCl_3$)** δ 198.64, 171.93, 154.62, 136.41, 133.31, 132.58, 132.14, 128.77, 128.59, 128.22, 128.14, 127.95, 127.67, 127.61, 126.09, 125.86, 66.58, 61.21, 52.90, 48.88, 41.67; **HRMS(ESI)** m/z: $[M+H]^+$ Calculated for $C_{24}H_{24}NO_5^+$ 406.1649; found 406.1648.

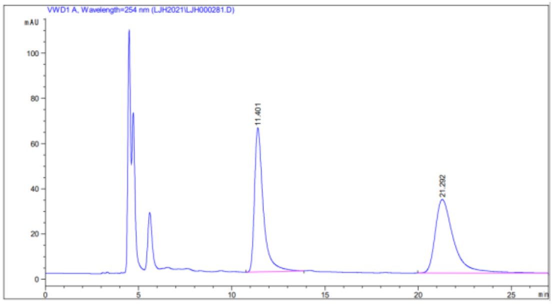

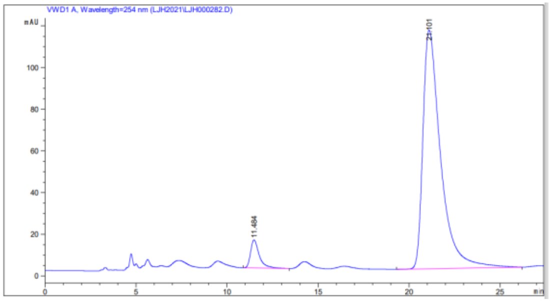

Peak #	RetTime [min]	Type	Width [min]	Area [mAU*s]	Height [mAU]	Area %
1	11.401	BB	0.4871	2086.89160	63.71700	48.3011
2	21.292	BBA	1.0227	2233.69263	32.52784	51.6989

Peak #	RetTime [min]	Type	Width [min]	Area [mAU*s]	Height [mAU]	Area %
1	11.484	BB	0.4943	445.89587	13.46161	5.4514
2	21.101	BB	1.0030	7733.56348	114.60736	94.5486

Methyl (R)-2-(((benzyloxy)carbonyl)amino)-2-(naphthalen-1-ylmethyl)-4-oxobutanoate (10 b):

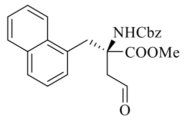

Colorless oil (57.2 mg, 47%); R_f = 0.32 (petroleum ether/ethyl acetate = 2:1); the enantiomeric excess was determined to be 86% by HPLC analysis on Daicel Chirapak OD-H column (hexane/isopropanol = 90/10, flow rate 1.0 mL/min, T = 30 °C), UV 254 nm, t_R(major) 15.066min, t_R(minor) 12.347 min; $[\alpha]_D^{25}$ = +99.55 (c=1.04, $CHCl_3$); **^{1}H NMR (400 MHz, $CDCl_3$)** δ 9.65 (s, 1H), 7.95 (d, *J* = 8.0 Hz, 1H), 7.86 – 7.77 (m, 1H), 7.73 (d, *J* = 8.0 Hz, 1H), 7.48 – 7.23 (m, 8H), 7.11 (d, *J* = 6.8 Hz, 1H), 5.87 (s, 1H), 5.17 (d, *J* = 12.0 Hz, 1H), 5.01 (d, *J* = 12.0 Hz, 1H), 4.11 (m, 1H), 3.89 (d, *J* = 14.0 Hz, 1H), 3.61 (d, *J* = 14.0 Hz, 1H), 3.48 (s, 3H), 3.22 (d, *J* = 18.0 Hz, 1H); **^{13}C NMR (101 MHz, $CDCl_3$)** δ 198.60, 171.92, 154.71, 136.32, 133.83, 132.71, 131.09, 128.88, 128.65, 128.55, 128.20, 128.12, 125.99, 125.57, 125.09, 123.46, 66.60, 61.13, 52.75, 48.55, 37.92; **HRMS(ESI)** m/z: $[M+H]^+$ Calculated for $C_{24}H_{24}NO_5^+$ 406.1649; found 406.1649.

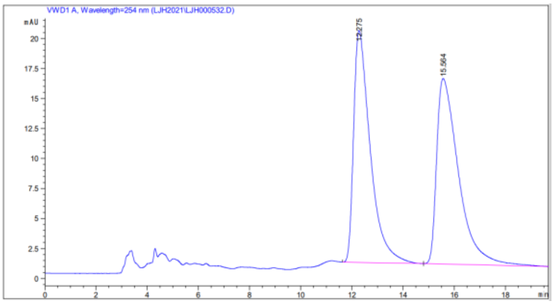

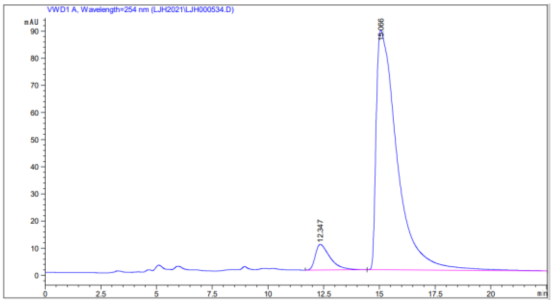

Peak #	RetTime [min]	Type	Width [min]	Area [mAU*s]	Height [mAU]	Area %
1	12.275	BB	0.6579	872.79602	19.33141	48.7546
2	15.564	BBA	0.8905	917.38715	15.46107	51.2454

Peak #	RetTime [min]	Type	Width [min]	Area [mAU*s]	Height [mAU]	Area %
1	12.347	BB	0.6749	433.03549	9.44385	6.9875
2	15.066	BBA	0.8737	5764.26904	88.32031	93.0125

5.4 The synthesis of compounds 12 and 13

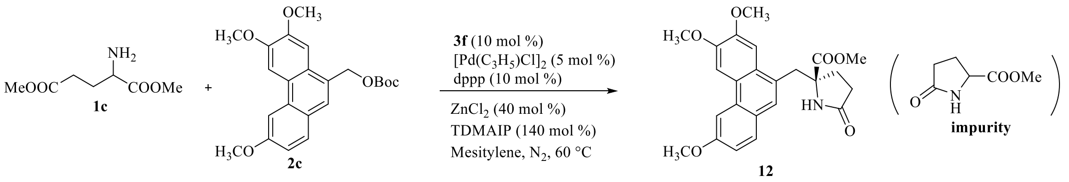

To a 10 mL vial charged with $[Pd(C_3H_5)Cl]_2$ (3.6 mg, 0.01 mmol) and dppp (8.2 mg, 0.02 mmol)was added 0.5 mL mesitylene, and the mixture was stirred under nitrogen atmosphere at room temperature for 30 min. Then, dimethyl glutamate **1c** (0.3 mmol), tert-butyl ((3,6,7-trimethoxyphenanthren-9-yl)methyl) carbonate **2c** (0.2 mmol), chiral aldehyde **3f** (7.7 mg, 0.02 mmol), $ZnCl_2$ (10.9 mg, 0.08 mmol) and TDMAIP (50.9 uL, 0.28 mmol) were added. The

mixture was continuously stirred at 60 ºC under nitrogen atmosphere. After the reaction completed, the solvent was removed by rotary evaporation, and the residue was purified by flash chromatography column on silica gel (eluent: petroleum ether/ ethyl acetate/ triethylamine =100/200/3) to afford crude product **12**, which contained the byproduct of methyl 5-oxopyrrolidine-2-carboxylate.

Methyl (S)-5-oxo-2-((3,6,7-trimethoxyphenanthren-9-yl)methyl)pyrrolidine-2-carboxylate (12)

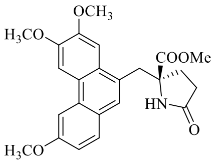

Colorless oil (56.1 mg, 65%); R_f= 0.28 (petroleum ether/ethyl acetate = 1:2); the enantiomeric excess was determined to be 86% by HPLC analysis on Daicel Chirapak AD-H column (hexane/isopropanol = 70/30, flow rate 1.0 mL/min, T = 30 ºC), UV 254 nm, t_R(major) 24.892min, t_R(minor) 10.677 min; $[\alpha]_D^{25}$ = +2.02 (c=1.82, $CHCl_3$); **^{1}H NMR (400 MHz, $CDCl_3$)** δ 7.89 (s, 1H), 7.80 (m, 1H), 7.68 (d, *J* = 8.8 Hz, 1H), 7.39 (s, 1H), 7.35 (s, 1H), 7.16 (dd, *J* = 8.8, 2.4 Hz, 1H), 6.41 (s, 1H), 4.09 (s, 3H), 4.06 (s, 3H), 3.99 (s, 3H), 3.68 (d, *J* = 14.3 Hz, 1H), 3.45 (s, 3H), 3.40 (d, *J* = 14.3 Hz, 1H), 2.63 – 2.54 (m, 1H), 2.40 – 2.33 (m, 2H), 2.33 – 2.28 (m, 1H); **^{13}C NMR (101 MHz, $CDCl_3$)** δ176.31, 174.01, 158.42, 149.43, 148.83, 130.75, 129.95, 127.19, 126.54, 126.41, 125.53, 124.87, 115.72, 104.93, 104.06, 103.84, 66.78, 56.00, 55.89, 55.34, 52.49, 41.96, 31.49, 29.73; **HRMS(ESI)** m/z: $[M+H]^+$ Calculated. for $C_{24}H_{26}NO_6^+$ 424.1755; found 424.1756.

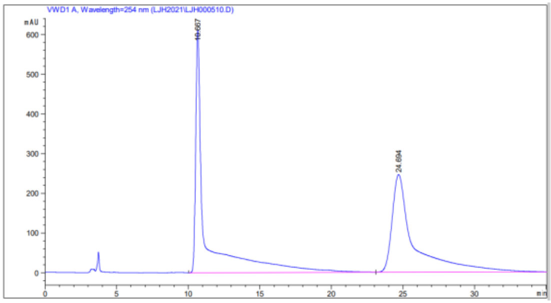

Peak #	RetTime [min]	Type	Width [min]	Area [mAU*s]	Height [mAU]	Area %
1	10.667	BB	0.6083	2.77106e4	610.50055	51.7161
2	24.694	BBA	1.4716	2.58715e4	245.47192	48.2839

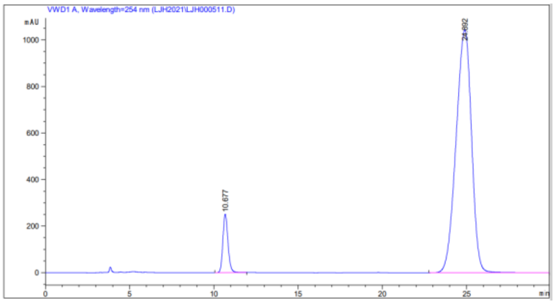

Peak #	RetTime [min]	Type	Width [min]	Area [mAU*s]	Height [mAU]	Area %
1	10.677	BB	0.3224	5282.14746	251.39505	7.3518
2	24.892	BBA	1.0030	6.65666e4	1042.39575	92.6482

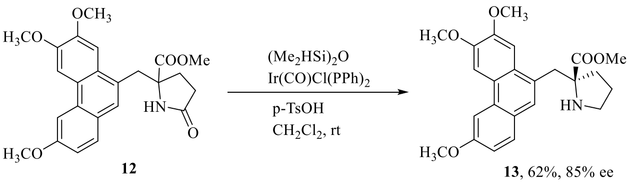

12 **13**, 62%, 85% ee

1,1,3,3-Tetramethyldisiloxane (0.56 mL, 3.6 mmol) was added to a solution of **12** (82.6 mg,

0.19 mmol) and $IrCl(CO)(PPh_3)_2$ (7.5 mg, 0.0095 mmol) in CH_2Cl_2 (15 mL) at room temperature. After stirring for 45 min, *p*-TsOH (39.6 mg, 0.23 mmol) was added to the resulting solution. The solution was continuously stirred for another 5 h and neutralized with saturated $NaHCO_3$ aqueous (10 mL). Then, the mixture was extracted with EtOAc (3 × 5 mL). The combined organic phase was washed with brine (5 mL), dried over Na_2SO_4, and concentrated by rotary evaporation. The residue was purified by flash chromatography column on silica gel (eluent: petroleum ether/ ethyl acetate/ triethylamine =100/200/3).

Methyl (S)-2-((3,6,7-trimethoxyphenanthren-9-yl)methyl)pyrrolidine-2-carboxylate (13):

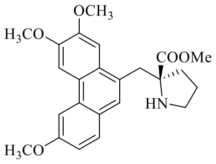

Colorless oil (49.9 mg, 62%); R_f = 0.32 (petroleum ether/ethyl acetate = 1:2); the enantiomeric excess was determined to be 85% by HPLC analysis on Daicel Chirapak OD-H column (hexane/isopropanol = 70/30, flow rate 1.0 mL/min, T = 30 °C), UV 254 nm, t_R(major) 21.968 min, t_R(minor) 11.253 min; $[\alpha]_D^{25}$ = +8.65 (c=0.58, $CHCl_3$); **^{1}H NMR (400 MHz, $CDCl_3$)** δ 7.74 (s, 1H), 7.67 (m, 1H), 7.58 (d, *J* = 8.8 Hz, 1H), 7.50 (s, 1H), 7.33 (s, 1H), 7.02 (dd, *J* = 8.8, 2.4 Hz, 1H), 3.95 (s, 3H), 3.93 (s, 3H), 3.85 (s, 3H), 3.44 (d, *J* = 14.0 Hz, 1H), 3.25 (s, 3H), 3.23 (d, *J* = 14.0 Hz, 1H), 2.87 (m, 1H), 2.82 – 2.70 (m, 1H), 2.23 (m, 1H), 2.13 (s, 1H), 1.88 – 1.77 (m, 1H), 1.73 – 1.63 (m, 1H), 1.59 – 1.48 (m, 1H); **^{13}C NMR (101 MHz, $CDCl_3$)** δ 177.33, 158.06, 148.97, 148.53, 132.10, 130.51, 129.85, 129.06, 127.33, 126.51, 125.88, 124.66, 115.43, 106.12, 103.83, 103.70, 70.85, 55.93, 55.56, 52.02, 45.81, 42.13, 35.77, 24.27; **HRMS(ESI)** m/z: $[M+H]^+$ Calculated for $C_{24}H_{28}NO_5^+$ 410.1962; found 410.1962.

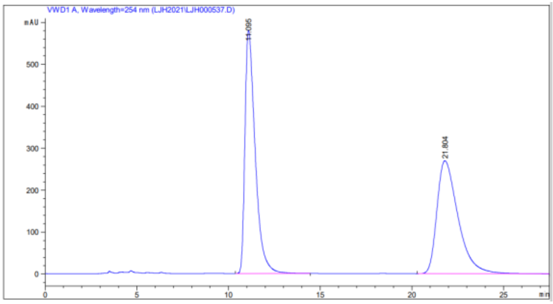

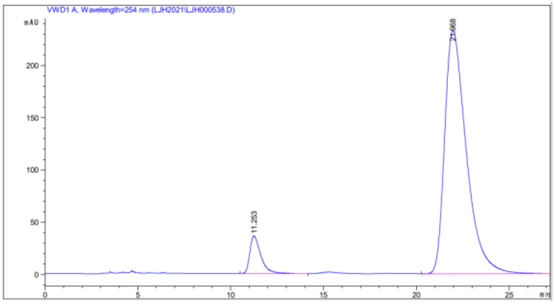

Peak #	RetTime [min]	Type	Width [min]	Area [mAU*s]	Height [mAU]	Area %
1	11.095	BB	0.5837	2.25421e4	579.33472	51.1524
2	21.804	BBA	1.2056	2.15263e4	269.18671	48.8476

Peak #	RetTime [min]	Type	Width [min]	Area [mAU*s]	Height [mAU]	Area %
1	11.253	BB	0.6161	1494.83496	36.07255	7.3416
2	21.968	BBA	1.2062	1.88665e4	232.84492	92.6584

6. Supplementary figures for the reaction mechanism investigation

6.1 Nonlinear effect investigation

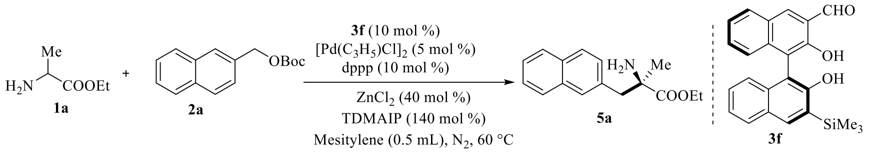

entry	ee of 3f (%)	ee of 5a (%)
1	19	21
2	39	39
3	58	50
4	78	71
5	100	90

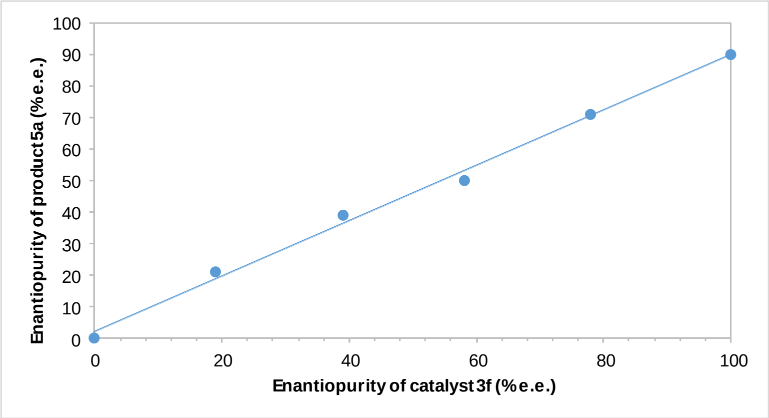

Supplementary Figure 1. The ee value relationship between 3f and product 5a.

6.2 Key intermediates detected by HRMS

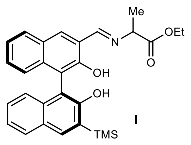

Mass Spectrum SmartFormula Report

Analysis Info

Analysis Name E:\Data\2022\20220317\2.d
Method DirectInfusion - MS - positive.m
Sample Name
Comment

Acquisition Date 3/17/2022 3:46:11 PM
Operator Demo User
Instrument impact II 1825265.10221

Acquisition Parameter

Source Type	ESI	Ion Polarity	Negative	Set Nebulizer	0.4 Bar
Focus	Active	Set Capillary	3000 V	Set Dry Heater	180 °C
Scan Begin	50 m/z	Set End Plate Offset	-500 V	Set Dry Gas	4.0 l/min
Scan End	1300 m/z	Set Charging Voltage	2000 V	Set Divert Valve	Source
		Set Corona	0 nA	Set APCI Heater	0 °C

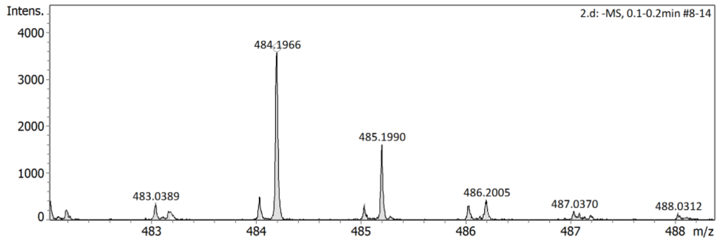

Meas. m/z	#	Sum Formula	m/z	Ion Formula	Adduct	err [ppm]	mSigma	Score	rdb	e⁻ Conf	N-Rule
484.1966	1	C29H31NO4Si	484.1950	C29H30NO4Si	M-H	-3.4	40.0	100.00	16.0	even	ok

Supplementary Figure 2: HRMS data of I.

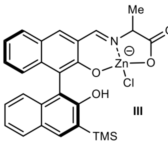

m/z = 554.0551 (M)

Mass Spectrum SmartFormula Report

Analysis Info

Analysis Name E:\Data\2022\20220317\2.d
Method DirectInfusion - MS - positive.m
Sample Name
Comment

Acquisition Date 3/17/2022 3:46:11 PM
Operator Demo User
Instrument impact II 1825265.10221

Acquisition Parameter

Source Type	ESI	Ion Polarity	Negative	Set Nebulizer	0.4 Bar
Focus	Active	Set Capillary	3000 V	Set Dry Heater	180 °C
Scan Begin	50 m/z	Set End Plate Offset	-500 V	Set Dry Gas	4.0 l/min
Scan End	1300 m/z	Set Charging Voltage	2000 V	Set Divert Valve	Source
		Set Corona	0 nA	Set APCI Heater	0 °C

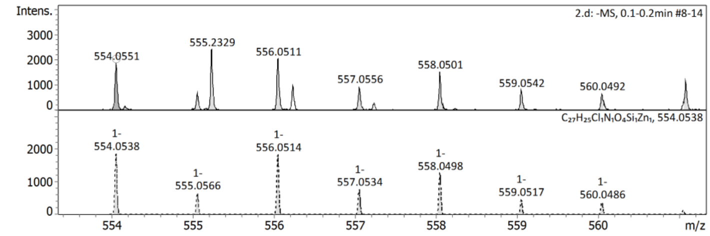

Meas. m/z	#	Sum Formula	m/z	Ion Formula	Adduct	err [ppm]	mSigma	Score	rdb	e⁻ Conf	N-Rule
554.0551	1	C27H26ClNO4SiZn	554.0538	C27H25ClNO4SiZn	M-H	-2.4	75.1	100.00	19.0	even	ok

Supplementary Figure 3: HRMS data of III.

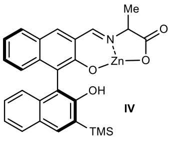

m/z = 518.0749 (M-H)

Mass Spectrum SmartFormula Report

Analysis Info

Analysis Name E:\Data\2022\20220317\2.d
Method DirectInfusion - MS - positive.m
Sample Name
Comment

Acquisition Date 3/17/2022 3:46:11 PM
Operator Demo User
Instrument impact II 1825265.10221

Acquisition Parameter

Source Type	ESI	Ion Polarity	Negative	Set Nebulizer	0.4 Bar
Focus	Active	Set Capillary	3000 V	Set Dry Heater	180 °C
Scan Begin	50 m/z	Set End Plate Offset	-500 V	Set Dry Gas	4.0 l/min
Scan End	1300 m/z	Set Charging Voltage	2000 V	Set Divert Valve	Source
		Set Corona	0 nA	Set APCI Heater	0 °C

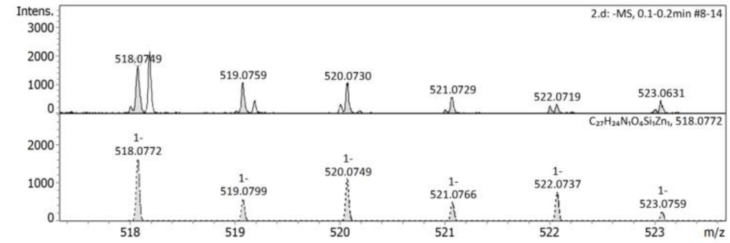

Meas. m/z	#	Sum Formula	m/z	Ion Formula	Adduct	err [ppm]	mSigma	Score	rdb	e⁻ Conf	N-Rule
518.0749	1	C27H25NO4SiZn	518.0772	C27H24NO4SiZn	M-H	4.3	163.3	100.00	17.0	even	ok

Supplementary Figure 4: HRMS data of IV.

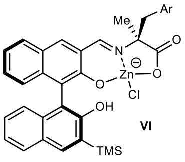

m/z = 694.1142 (M)

Mass Spectrum SmartFormula Report

Analysis Info

Analysis Name E:\Data\2022\20220317\2.d
Method DirectInfusion - MS - positive.m
Sample Name
Comment

Acquisition Date 3/17/2022 3:46:11 PM
Operator Demo User
Instrument impact II 1825265.10221

Acquisition Parameter

Source Type	ESI	Ion Polarity	Negative	Set Nebulizer	0.4 Bar
Focus	Active	Set Capillary	3000 V	Set Dry Heater	180 °C
Scan Begin	50 m/z	Set End Plate Offset	-500 V	Set Dry Gas	4.0 l/min
Scan End	1300 m/z	Set Charging Voltage	2000 V	Set Divert Valve	Source
		Set Corona	0 nA	Set APCI Heater	0 °C

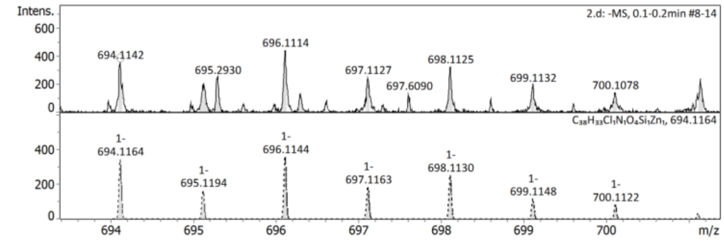

Meas. m/z	#	Sum Formula	m/z	Ion Formula	Adduct	err [ppm]	mSigma	Score	rdb	e⁻ Conf	N-Rule
694.1142	1	C38H34ClNO4SiZn	694.1164	C38H33ClNO4SiZn	M-H	3.2	79.6	100.00	26.0	even	ok

Supplementary Figure 5: HRMS data of VI.

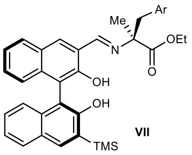

VII

m/z = 624.2575 (M-H)

Mass Spectrum SmartFormula Report

Analysis Info

Analysis Name	E:\Data\2022\20220317\2.d	Acquisition Date	3/17/2022 3:46:11 PM
Method	DirectInfusion - MS - positive.m	Operator	Demo User
Sample Name		Instrument	impact II 1825265.10221
Comment			

Acquisition Parameter

Source Type	ESI	Ion Polarity	Negative	Set Nebulizer	0.4 Bar
Focus	Active	Set Capillary	3000 V	Set Dry Heater	180 °C
Scan Begin	50 m/z	Set End Plate Offset	-500 V	Set Dry Gas	4.0 l/min
Scan End	1300 m/z	Set Charging Voltage	2000 V	Set Divert Valve	Source
		Set Corona	0 nA	Set APCI Heater	0 °C

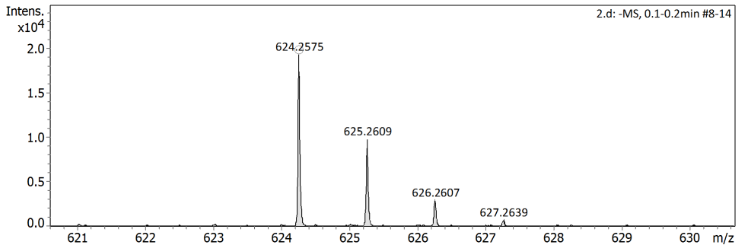

Meas. m/z	#	Sum Formula	m/z	Ion Formula	Adduct	err [ppm]	mSigma	Score	rdb	e⁻ Conf	N-Rule
624.2575	1	C40H39NO4Si	624.2576	C40H38NO4Si	M-H	0.1	8.8	100.00	23.0	even	ok

Supplementary Figure 6: HRMS data of VII.

7. Supplementary figures of ^{1}H and ^{13}C NMR spectrums

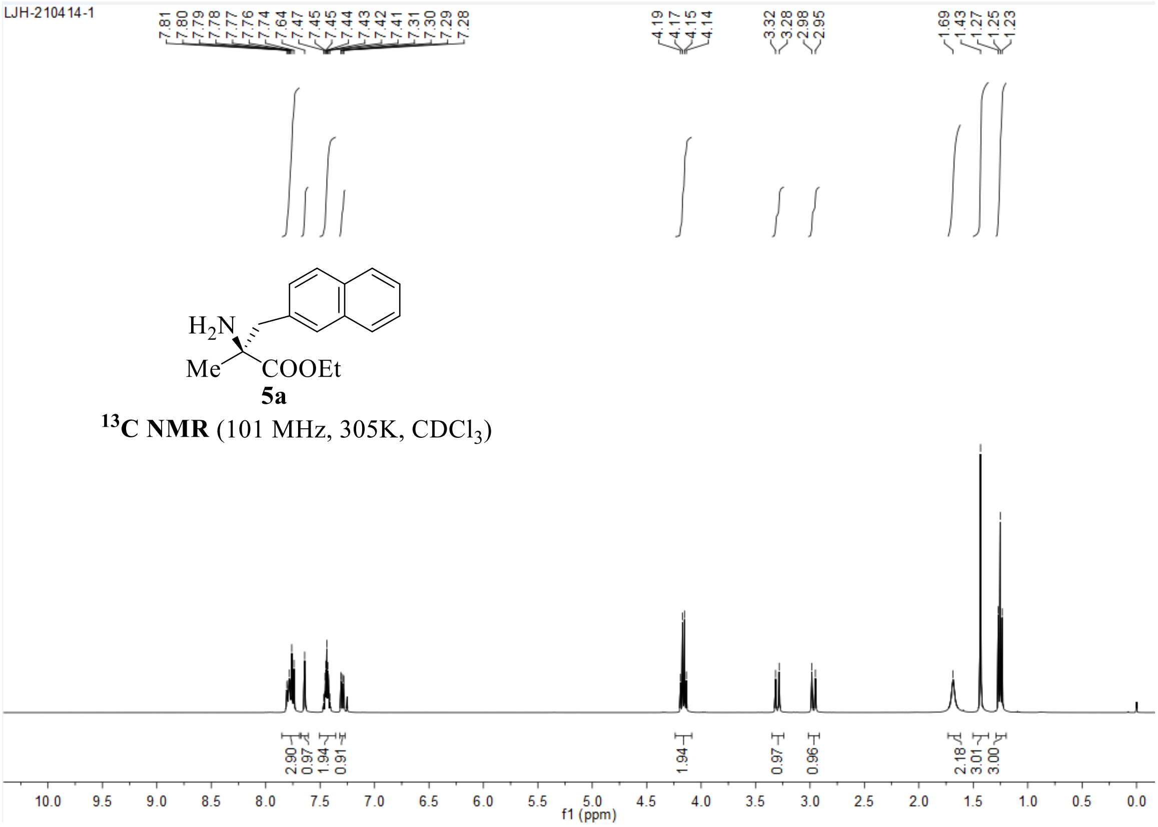

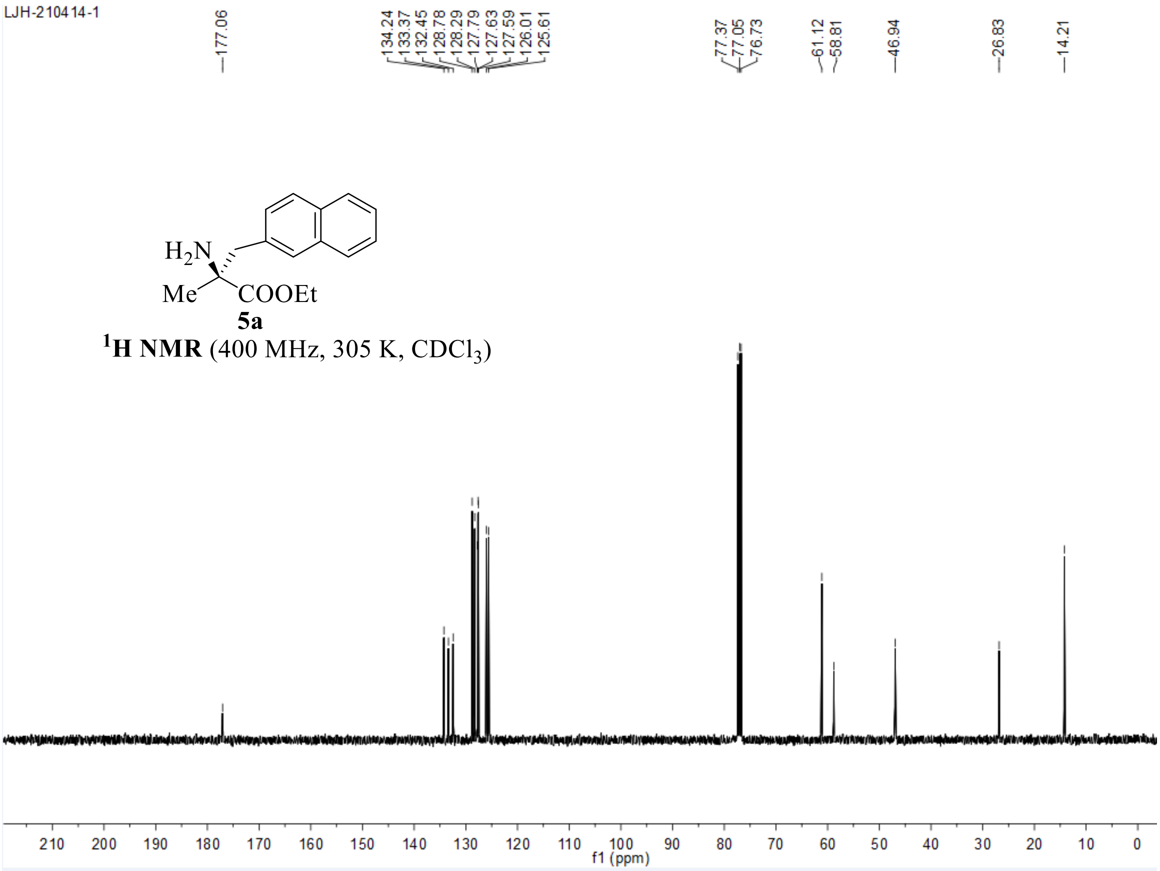

Supplementary Figure 7: NMR of compound 5a.

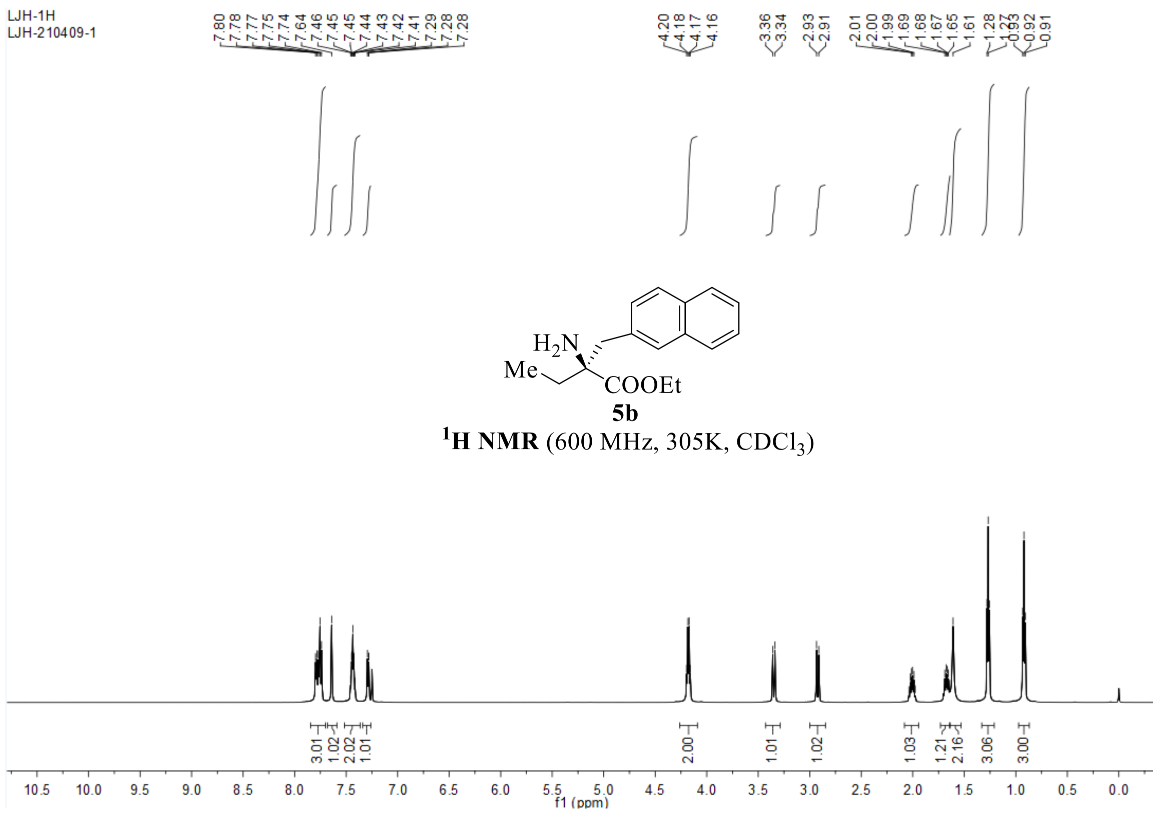

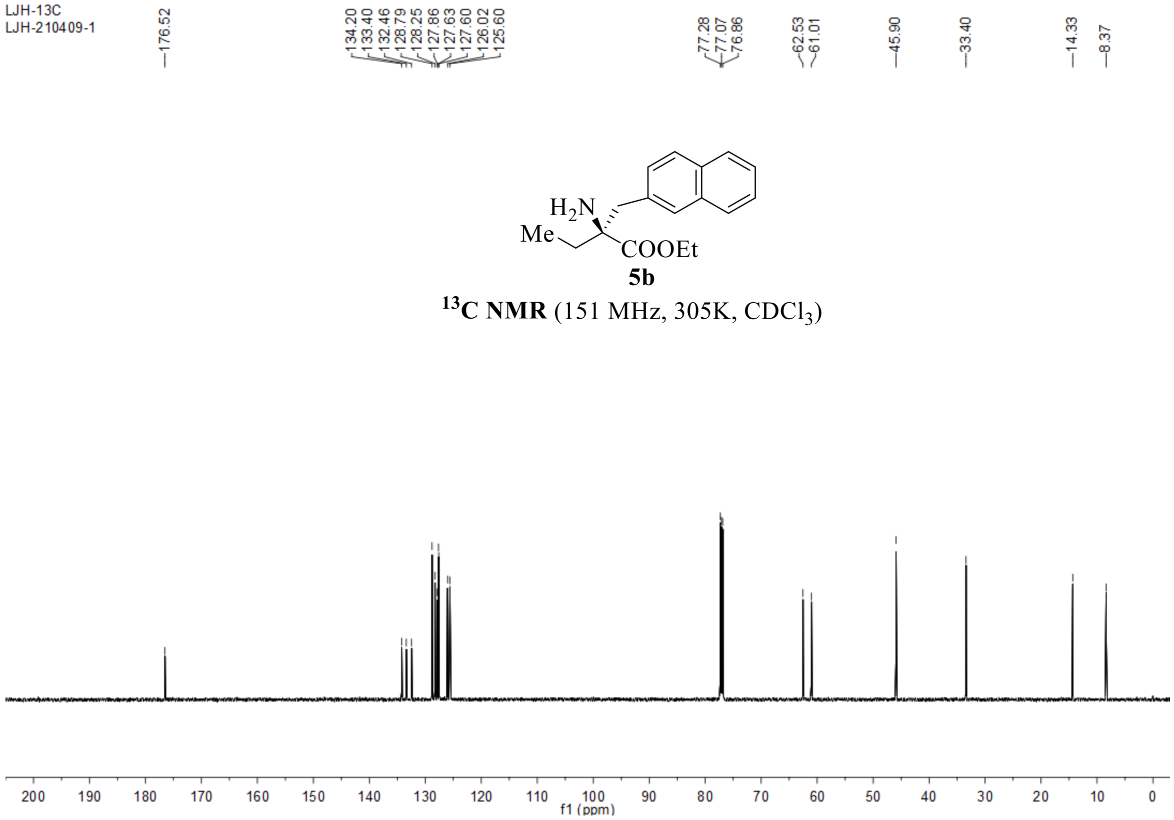

Supplementary Figure 8: NMR of compound 5b.

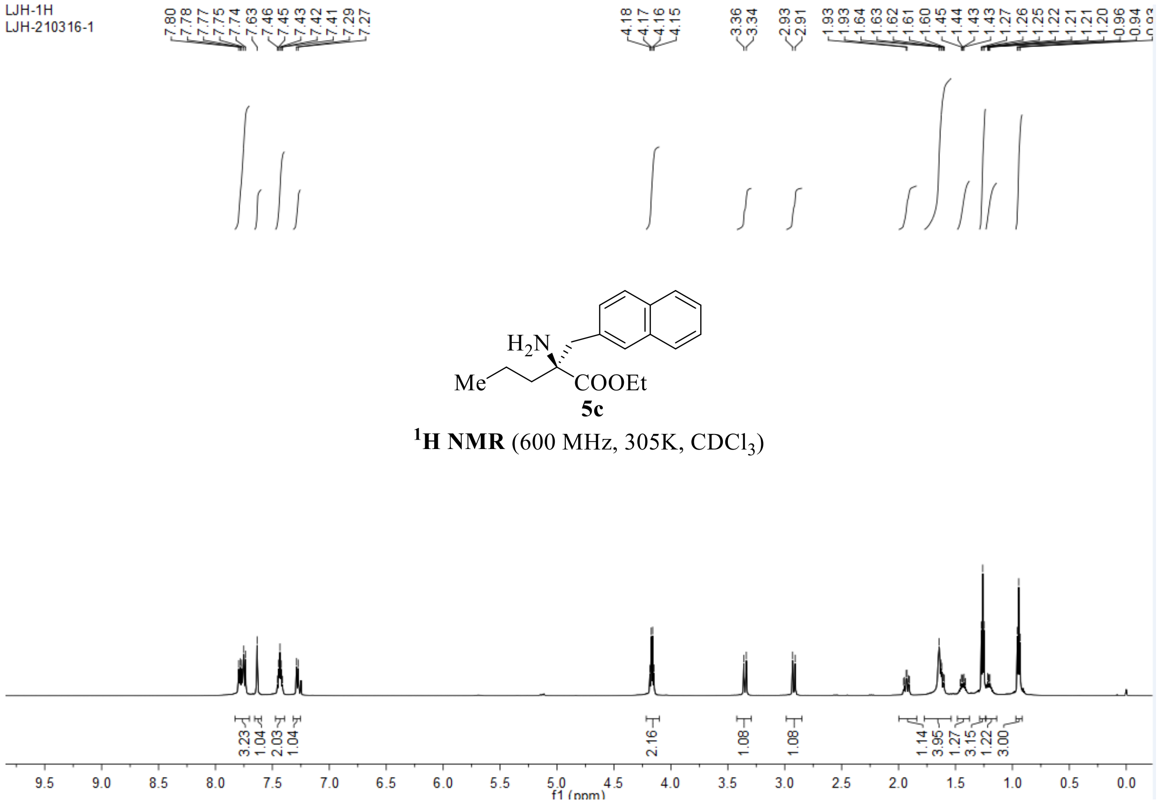

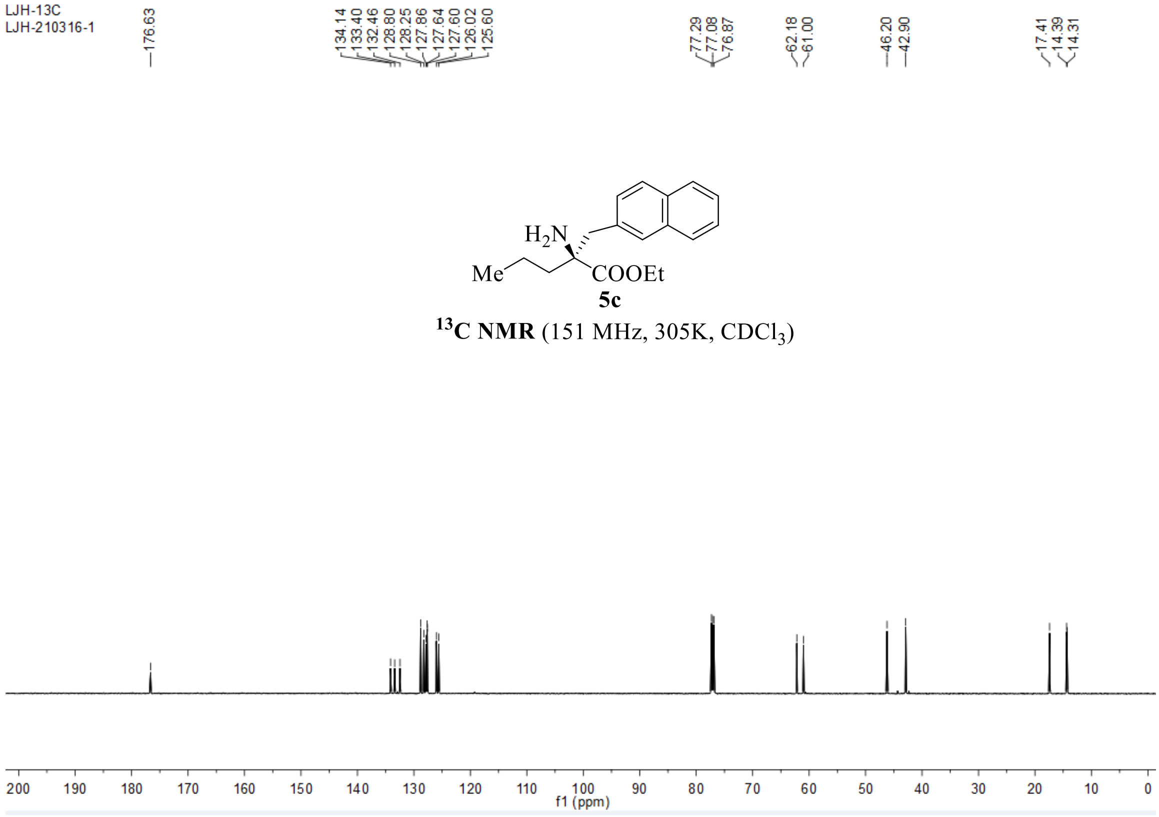

Supplementary Figure 9: NMR of compound 5c.

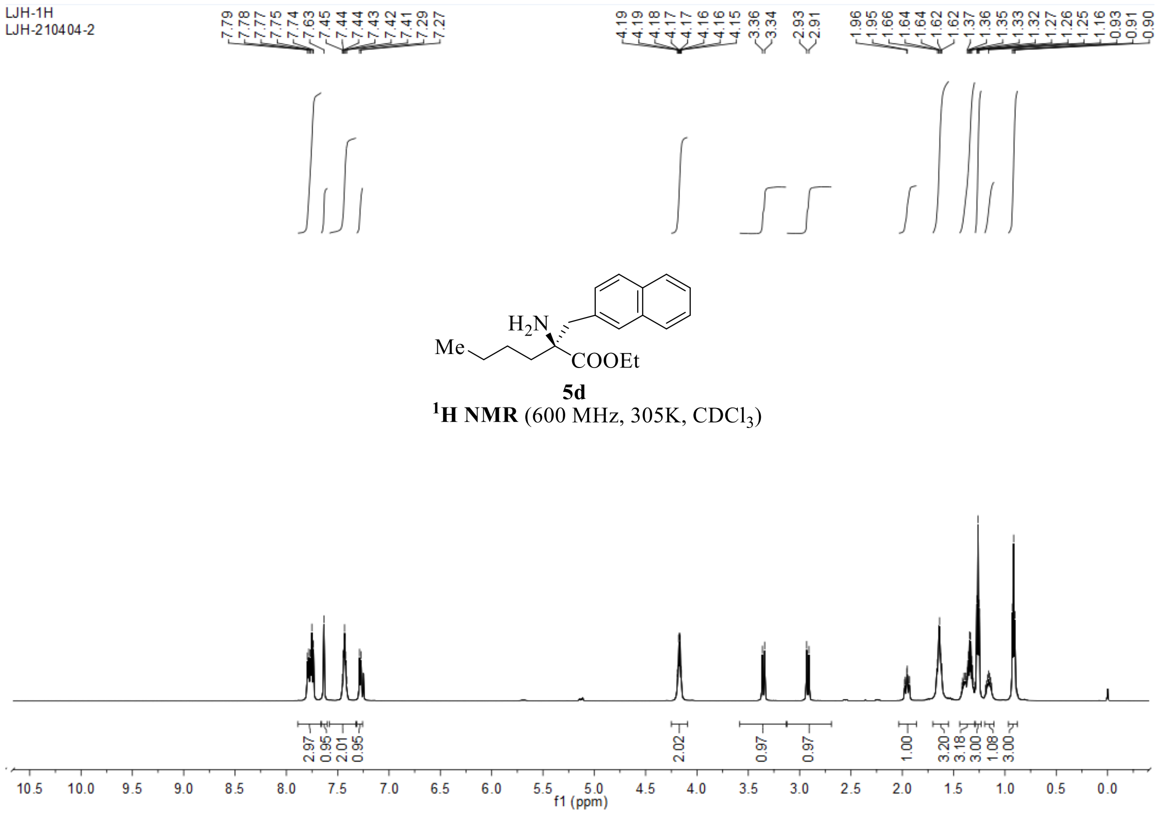

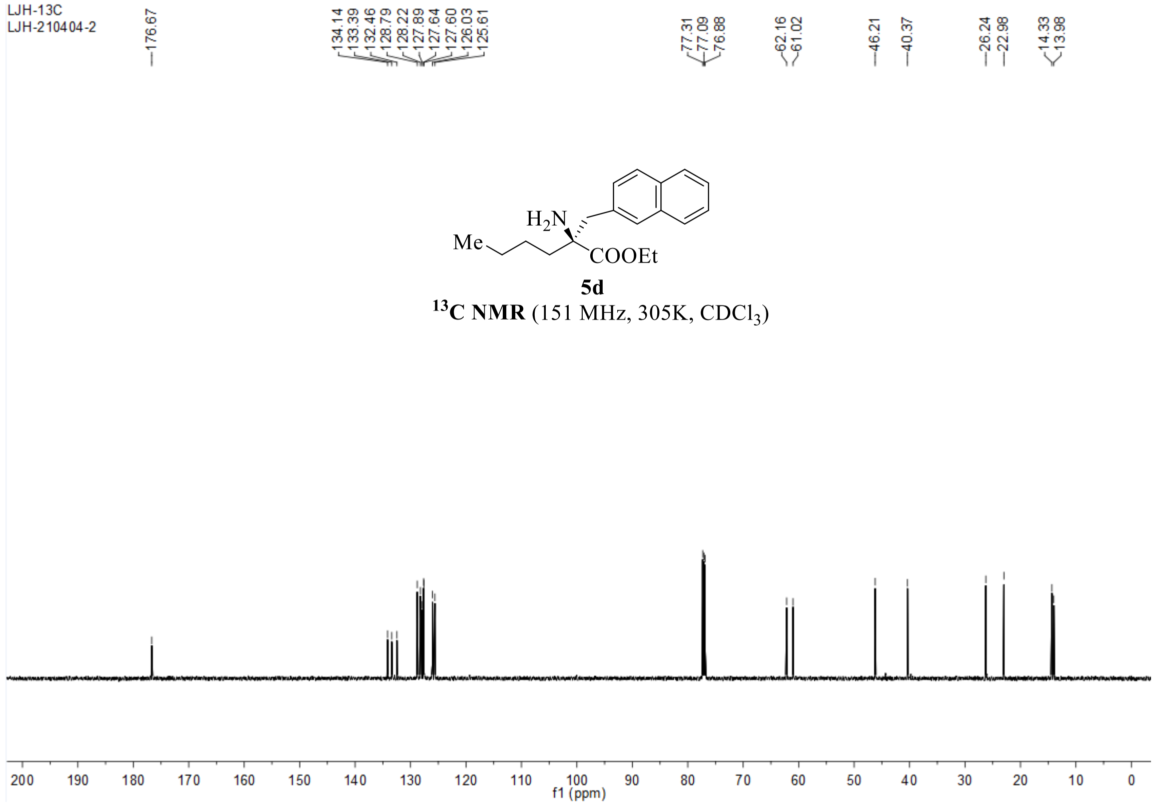

Supplementary Figure 10: NMR of compound 5d.

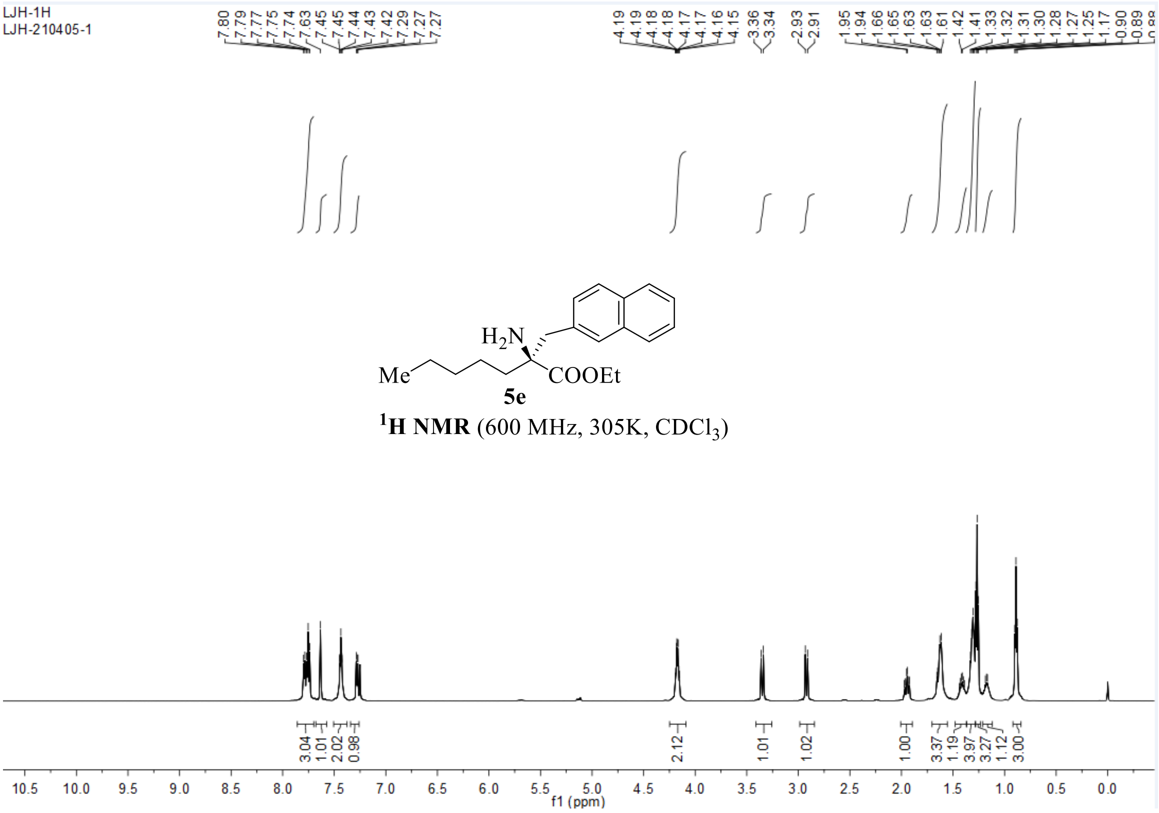

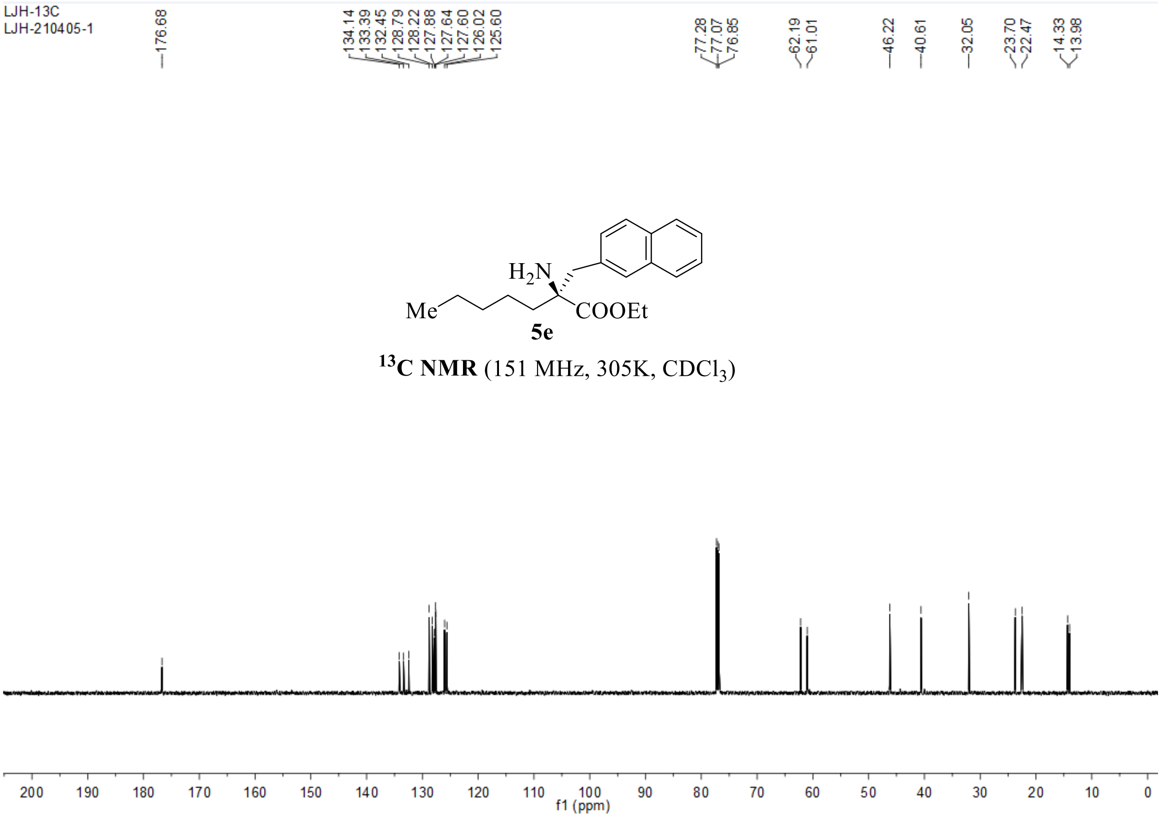

Supplementary Figure 11: NMR of compound 5e.

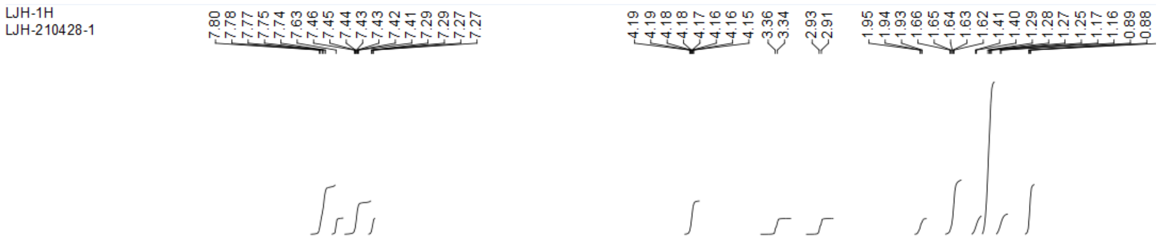

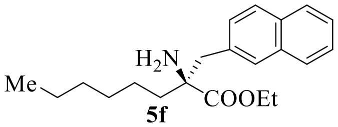

5f

[1]**H NMR** (600 MHz, 305K, $CDCl_3$)

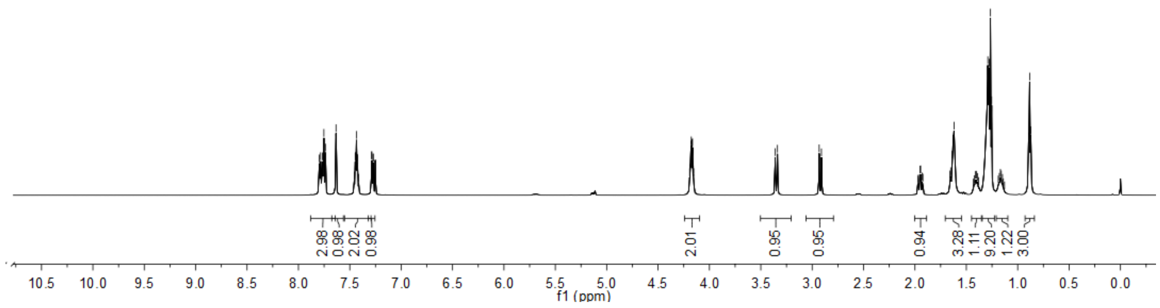

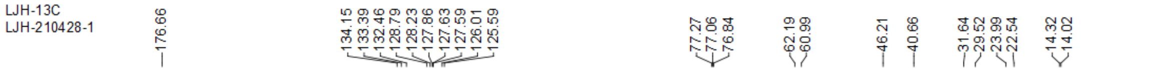

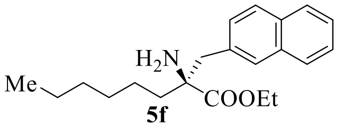

5f

[13]**C NMR** (151 MHz, 305K, $CDCl_3$)

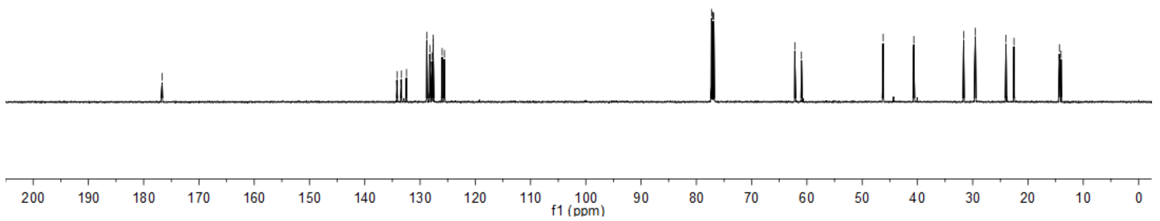

Supplementary Figure 12: NMR of compound 5f.

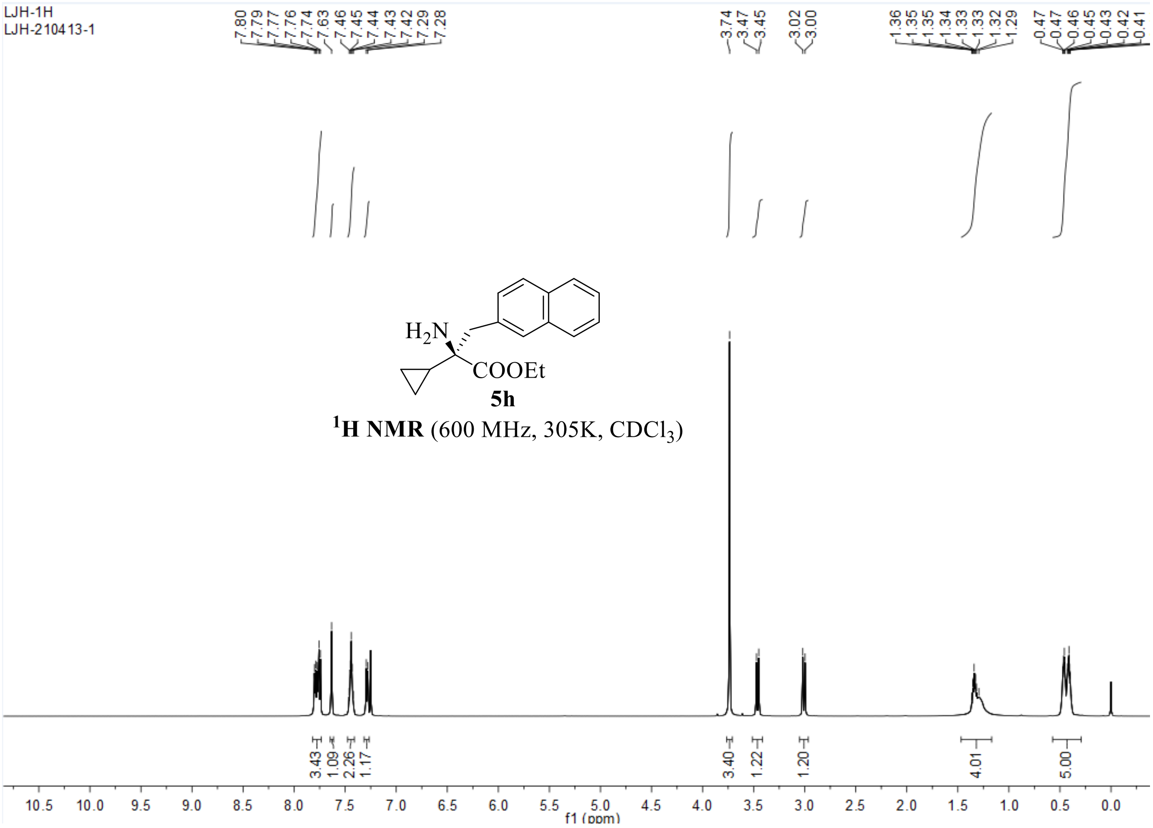

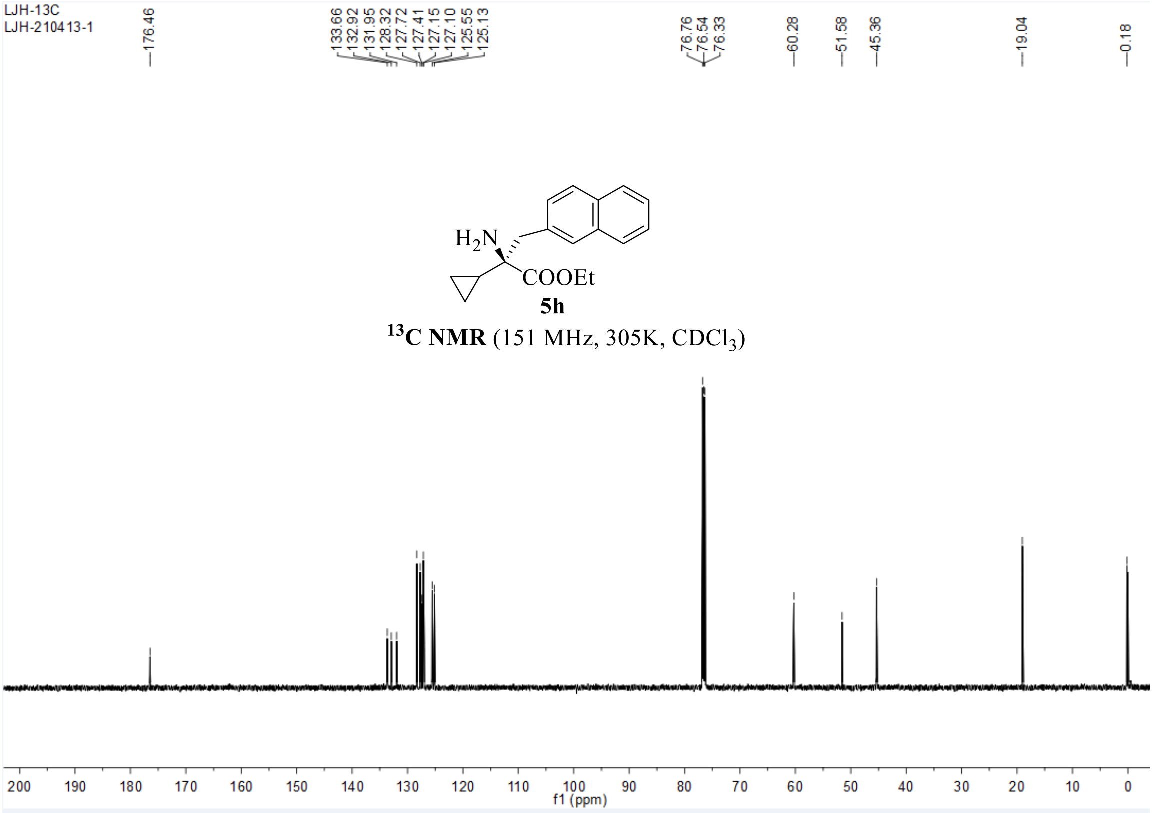

Supplementary Figure 13: NMR of compound 5h.

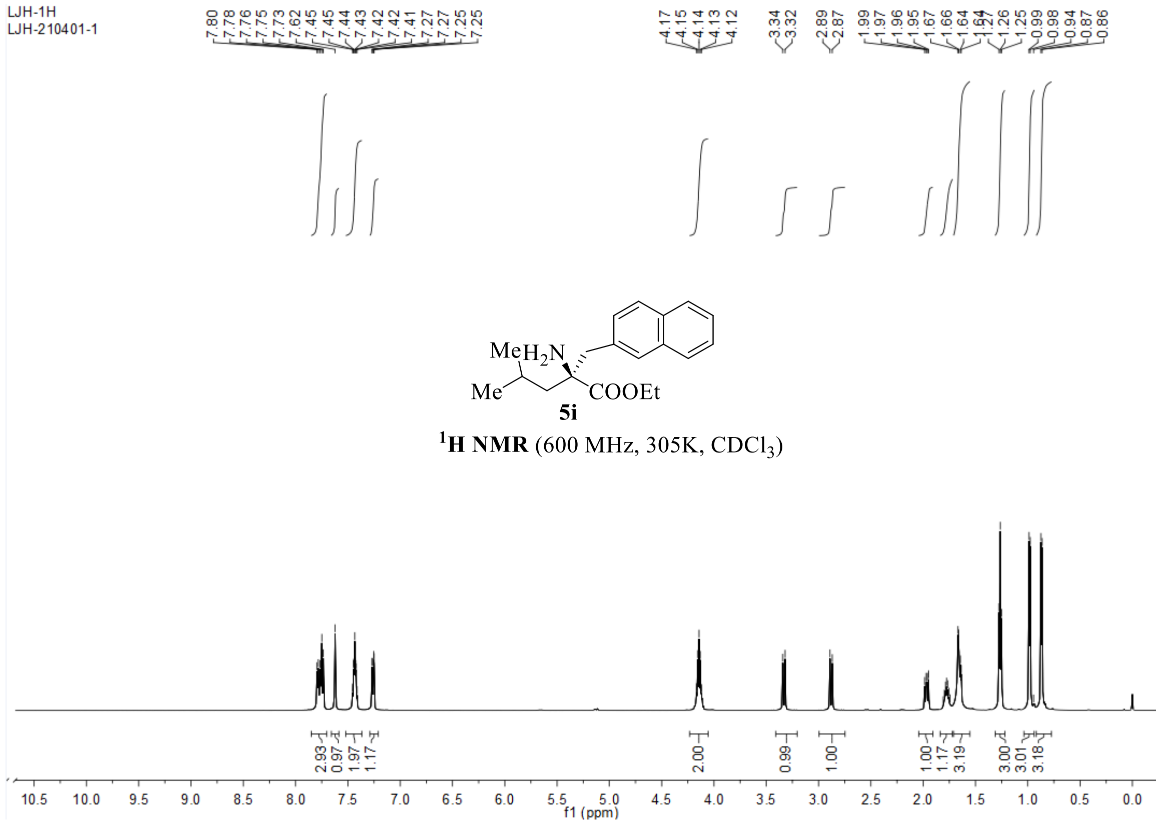

^{1}**H NMR** (600 MHz, 305K, $CDCl_3$)

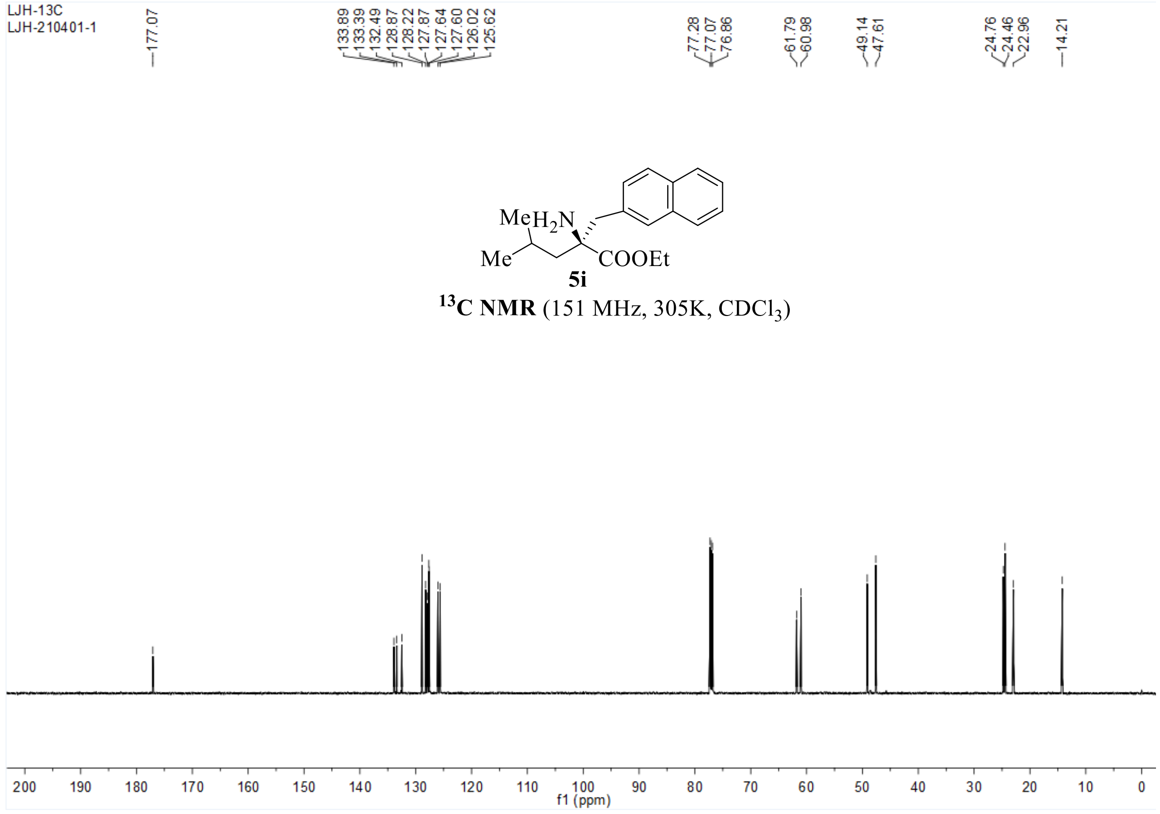

^{13}**C NMR** (151 MHz, 305K, $CDCl_3$)

Supplementary Figure 14: NMR of compound 5i.

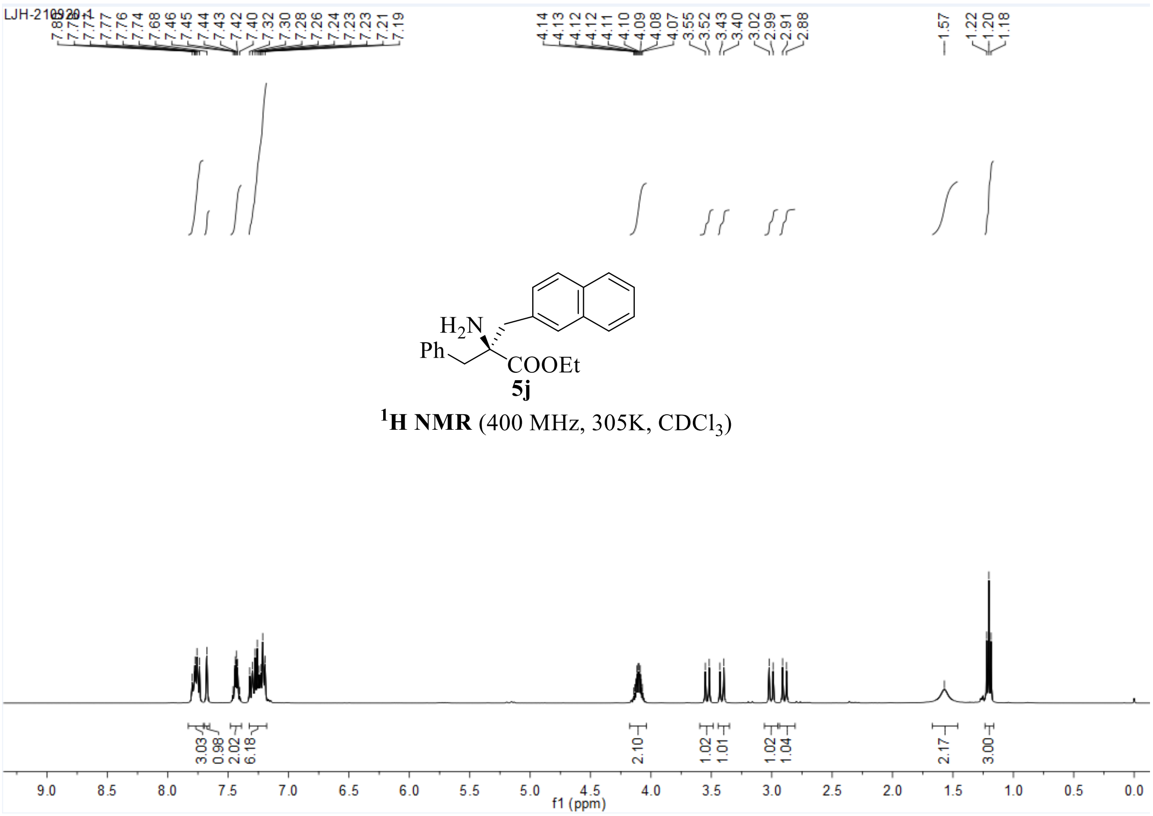

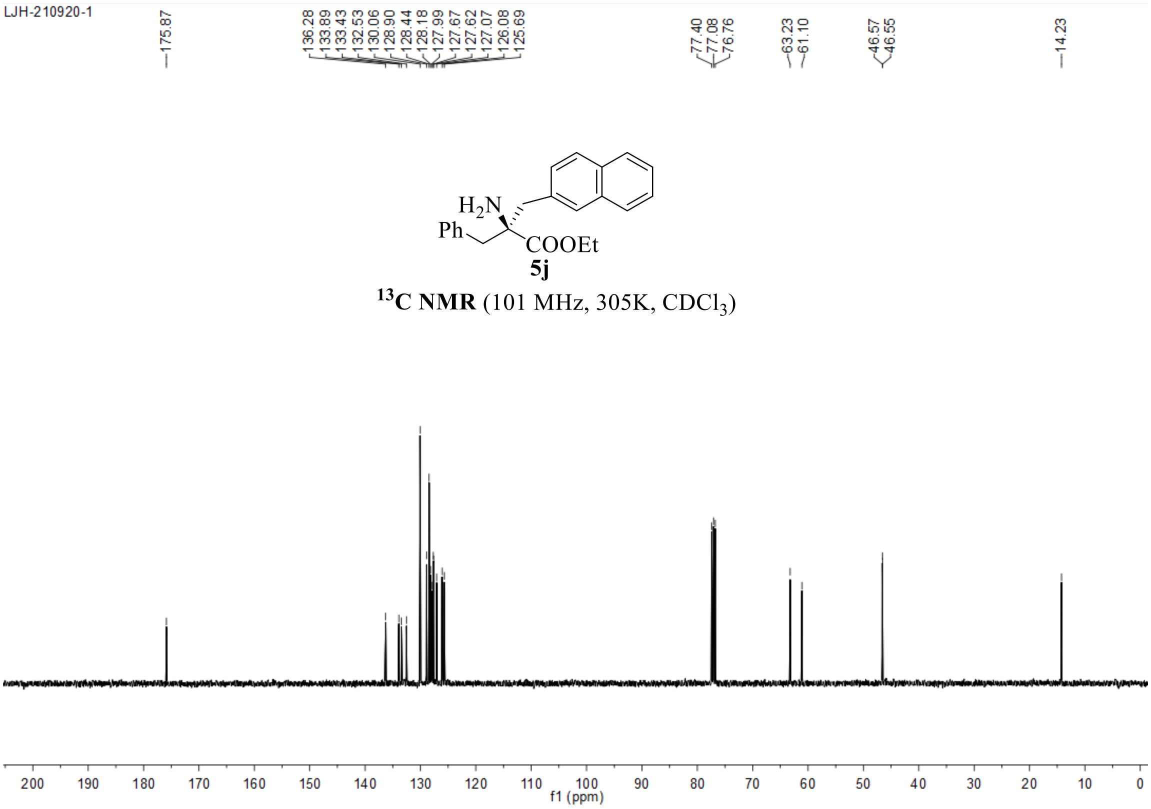

Supplementary Figure 15: NMR of compound 5j.

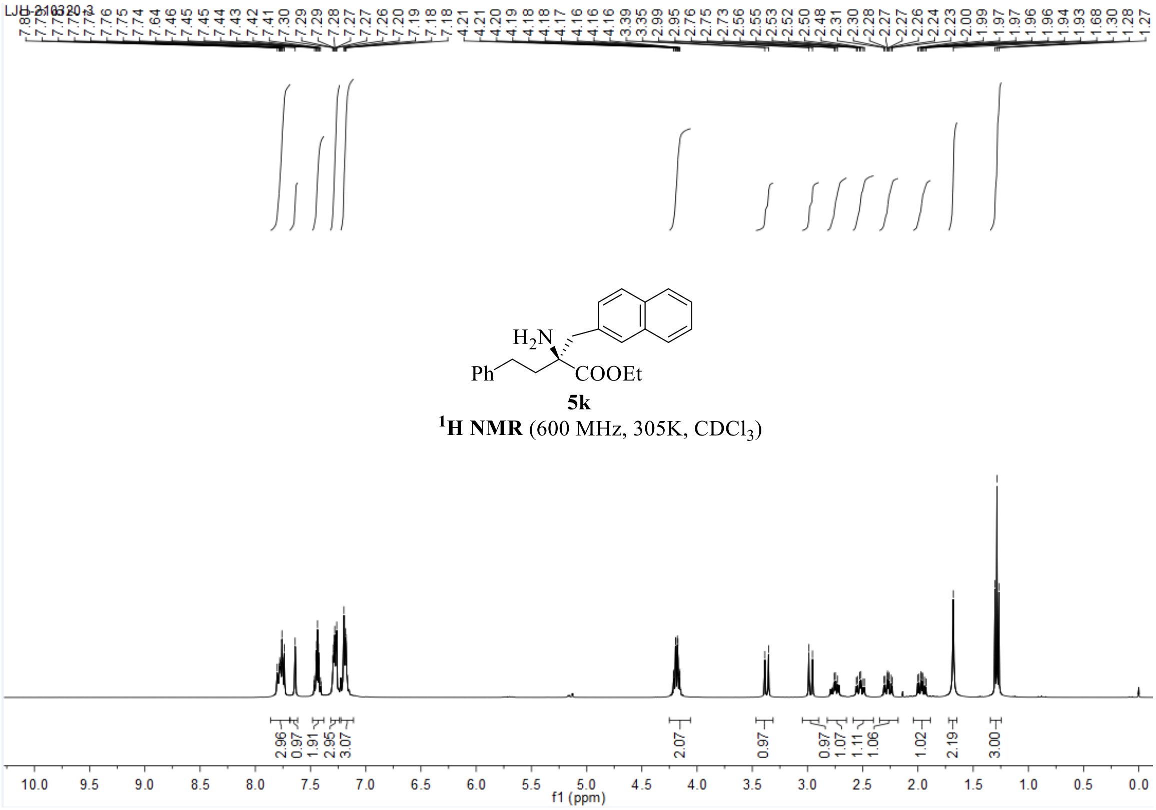

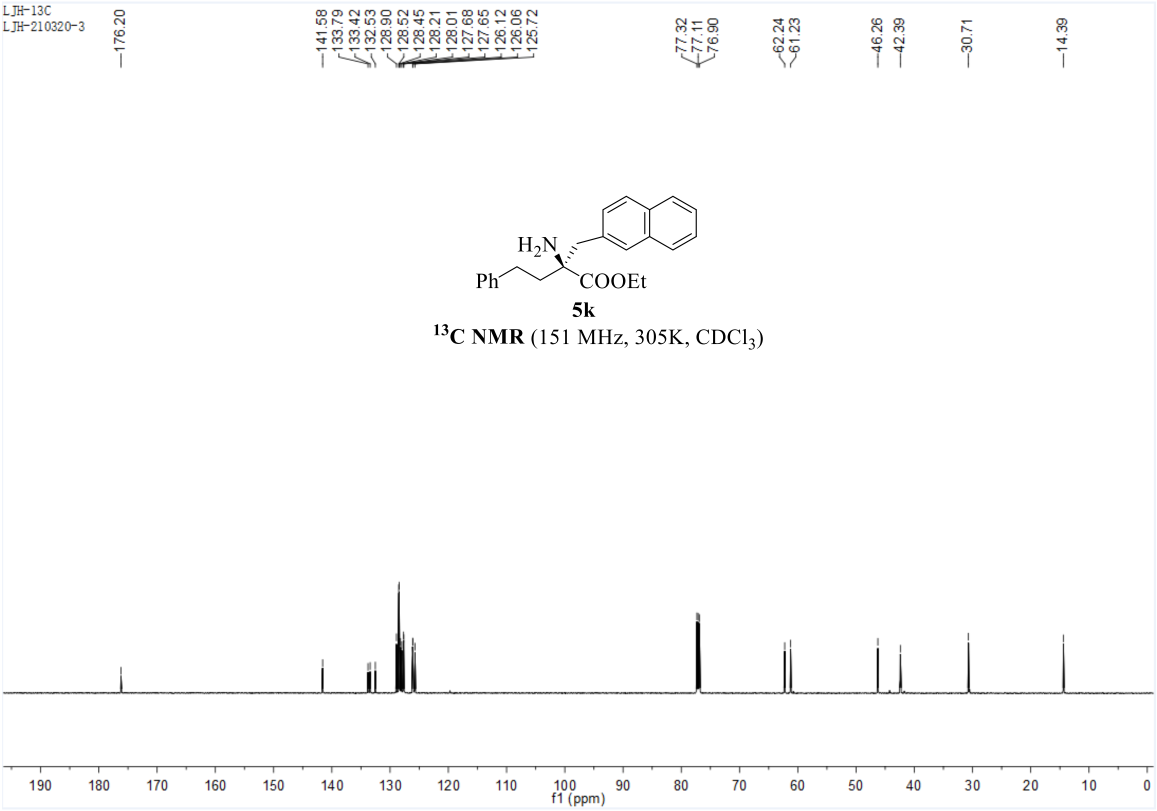

Supplementary Figure 16: NMR of compound 5k.

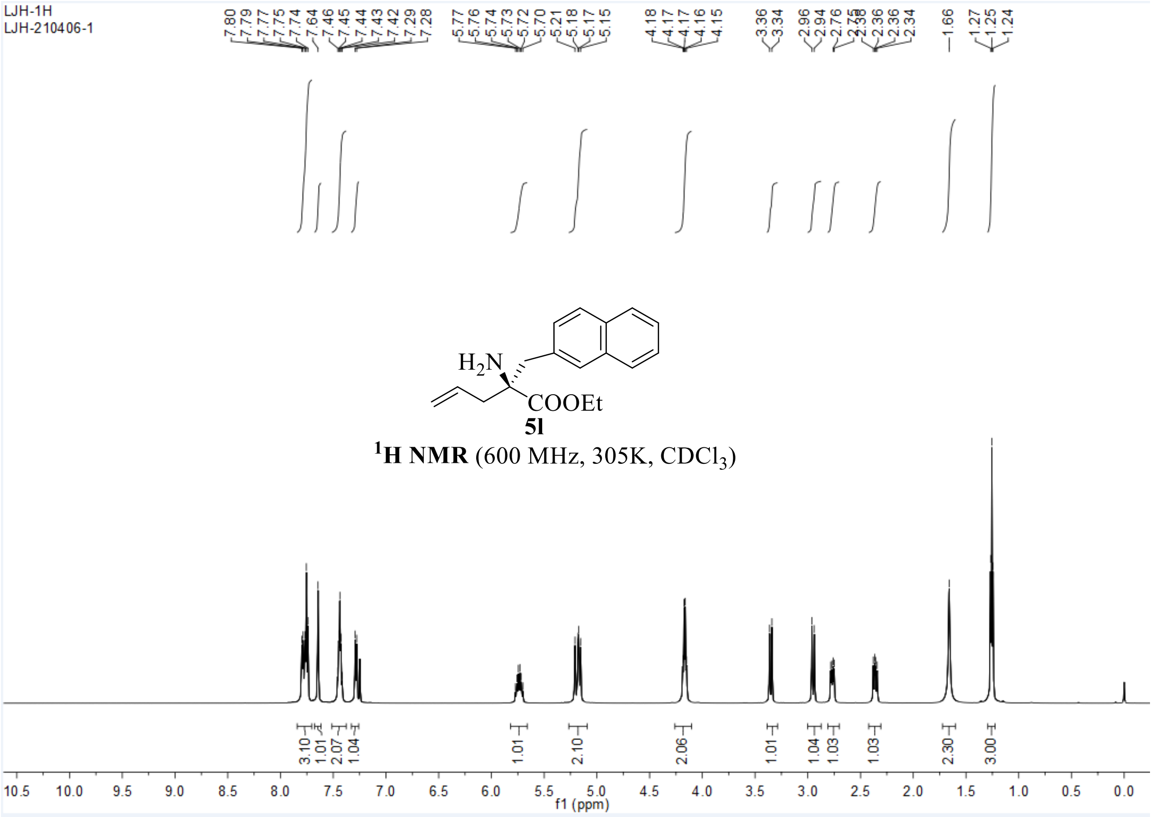

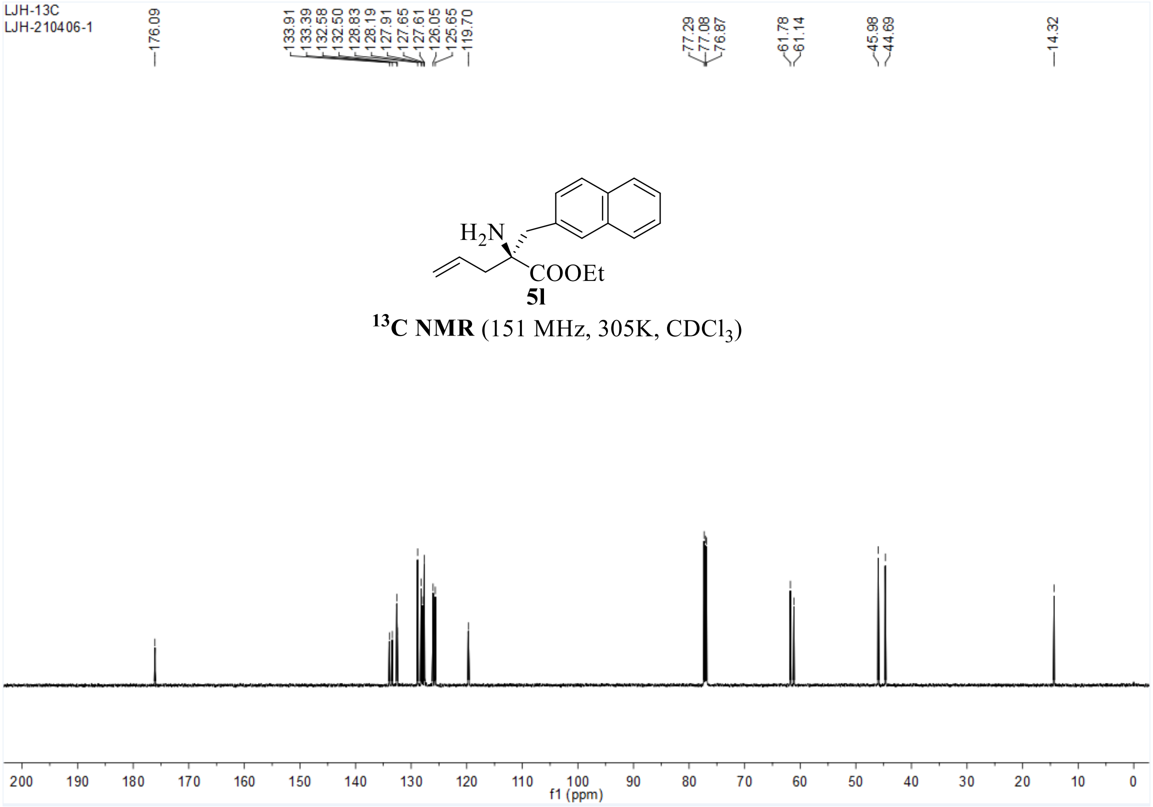

Supplementary Figure 17: NMR of compound 5l.

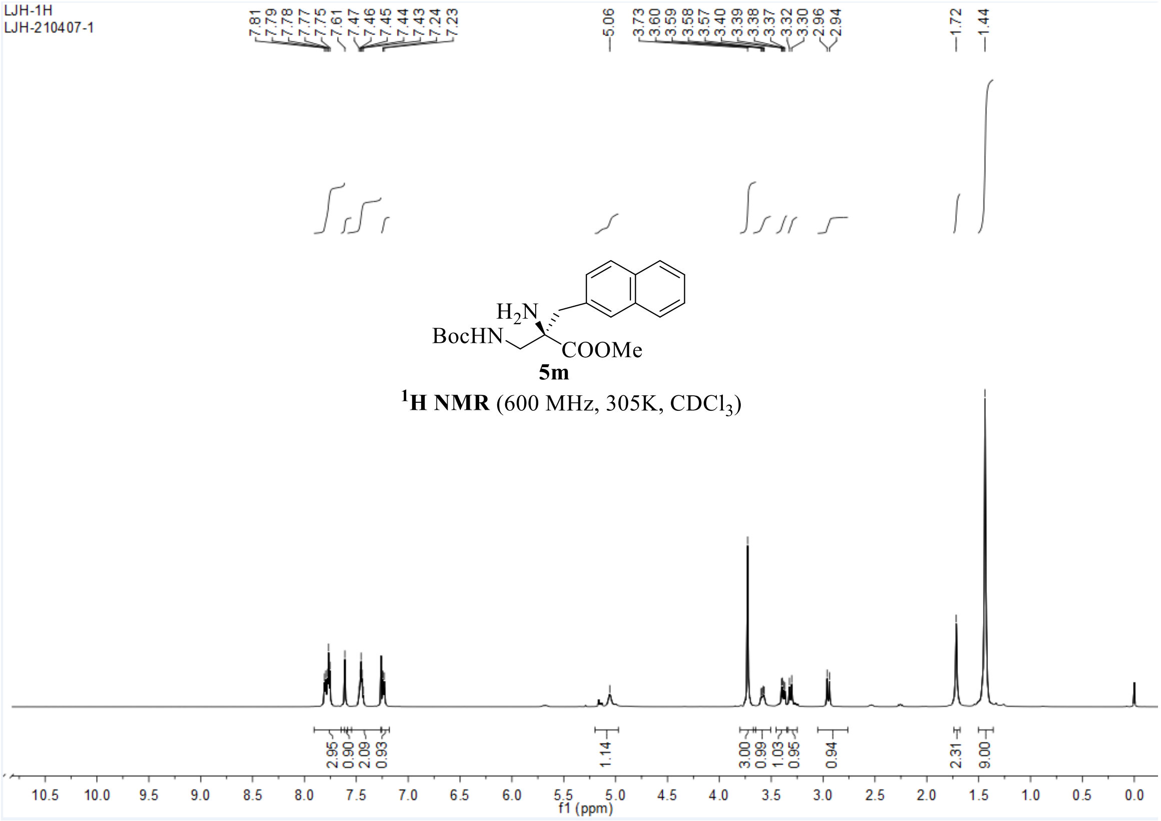

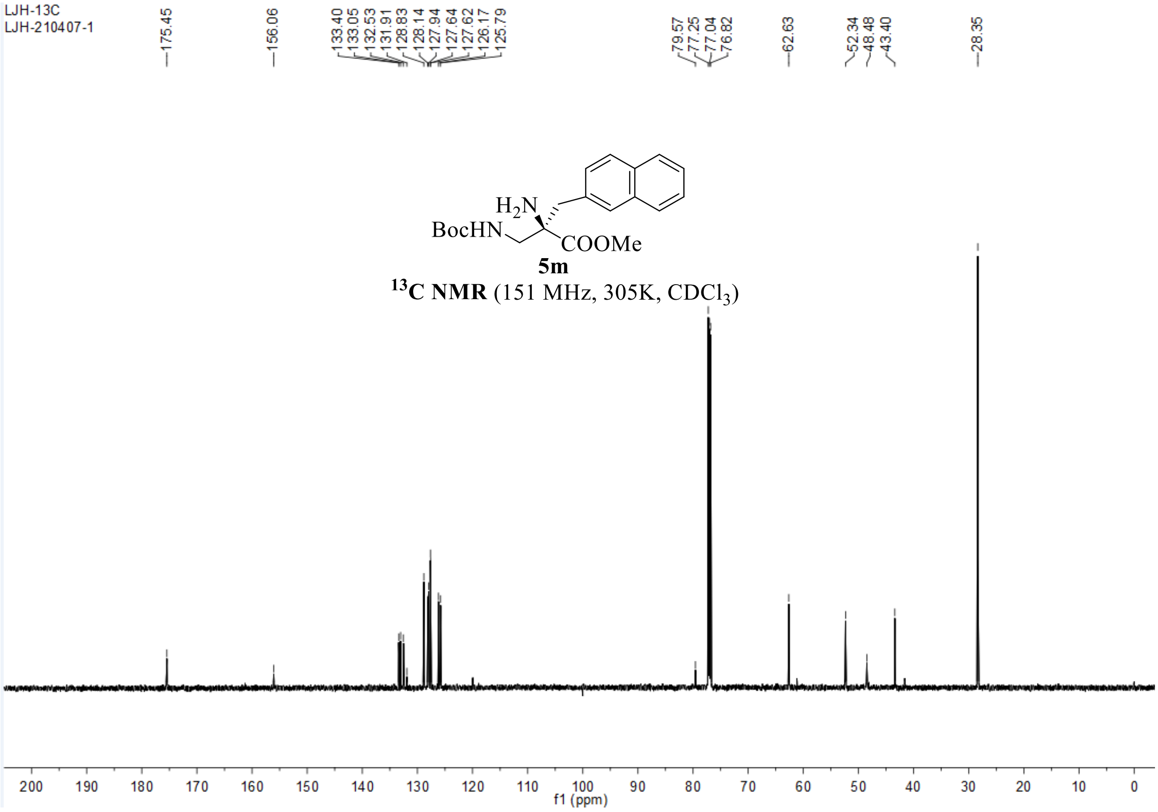

Supplementary Figure 18: NMR of compound 5m.

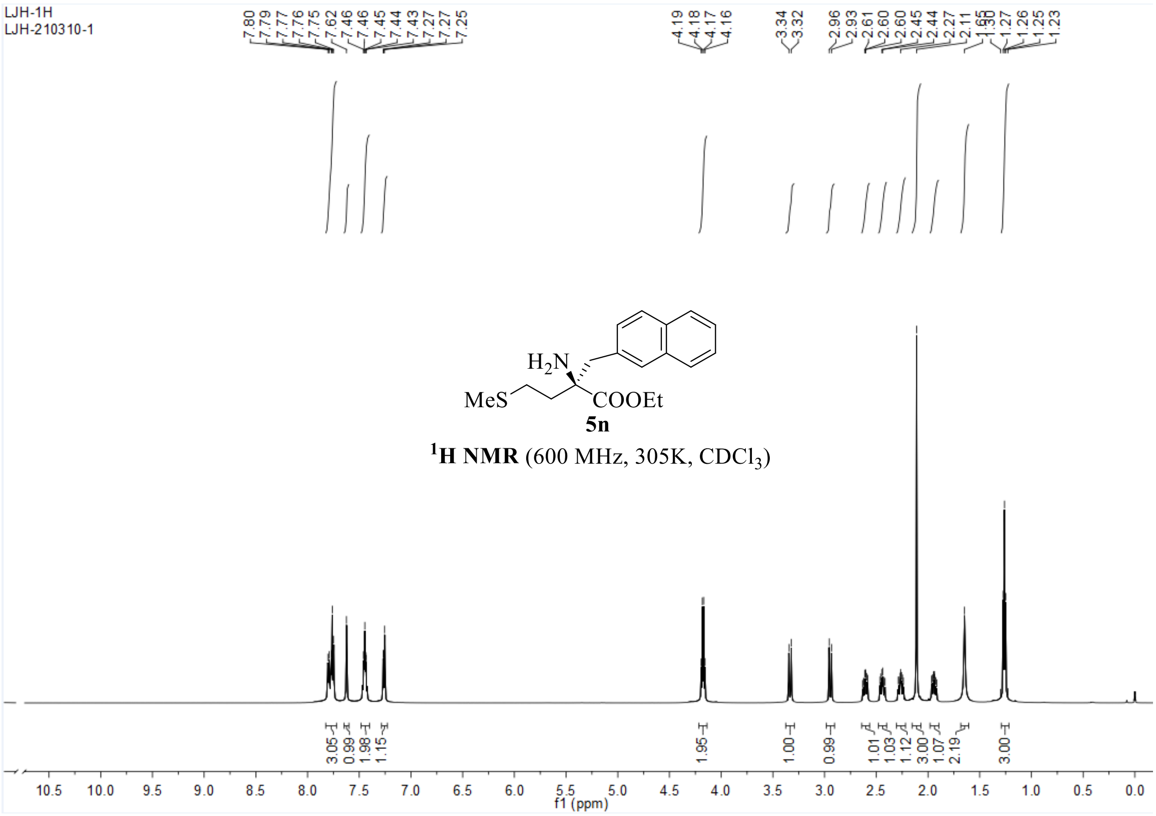

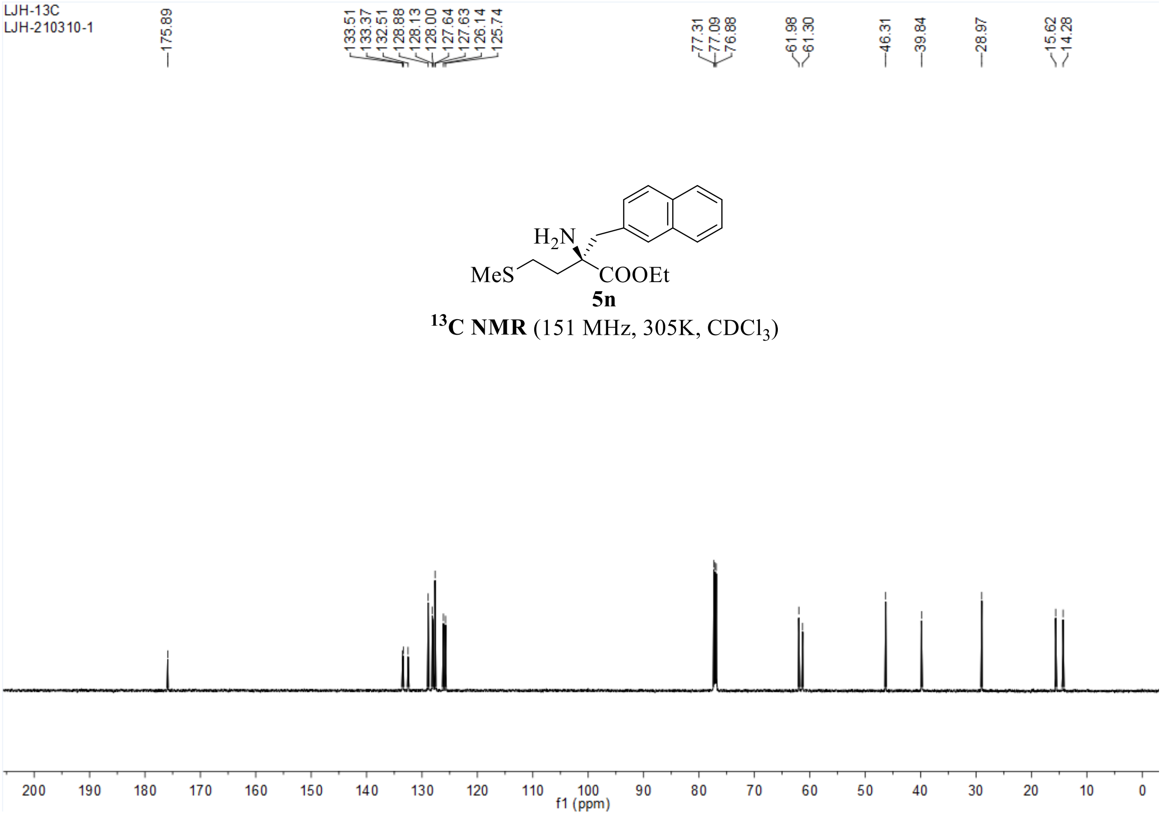

Supplementary Figure 19: NMR of compound 5n.

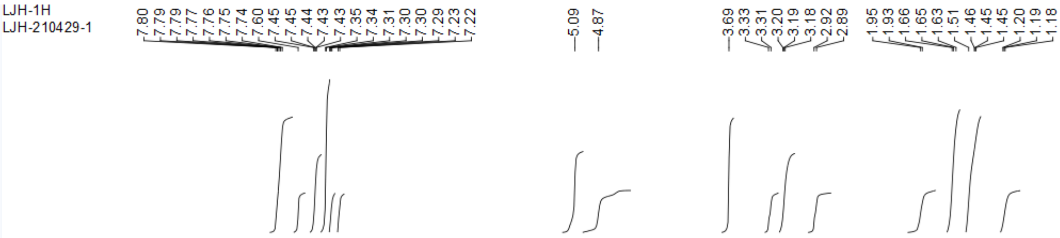

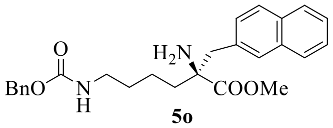

5o

^{1}H NMR (600 MHz, 305K, $CDCl_3$)

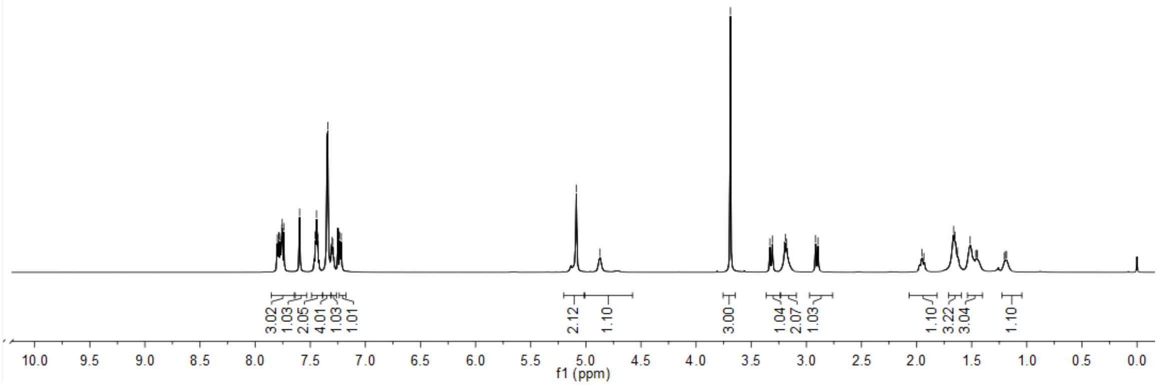

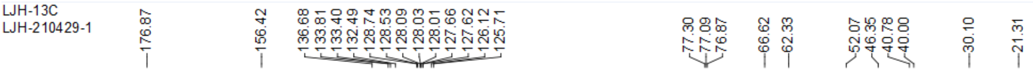

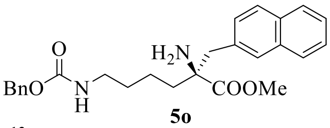

5o

^{13}C NMR (151 MHz, 305K, $CDCl_3$)

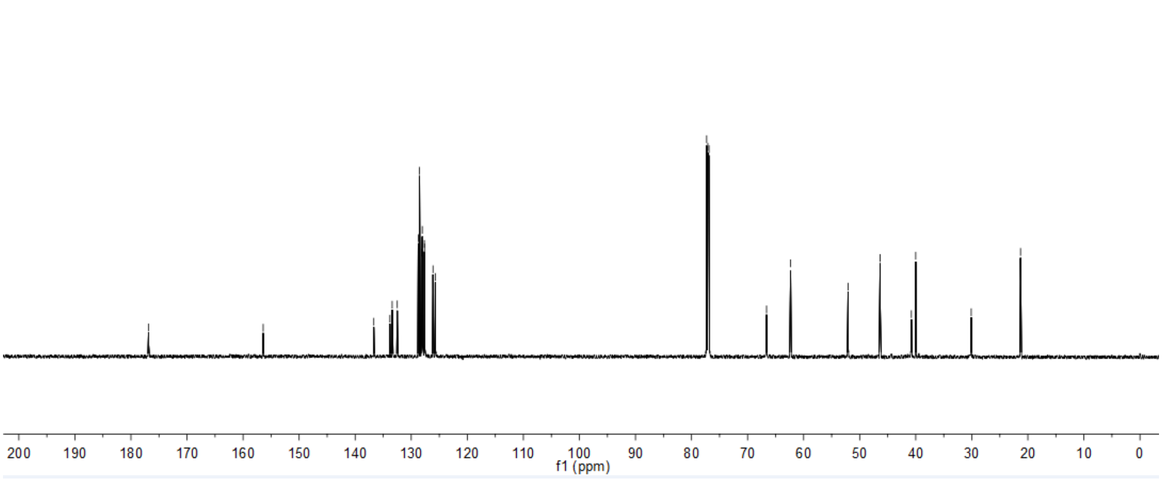

Supplementary Figure 20: NMR of compound 5o.

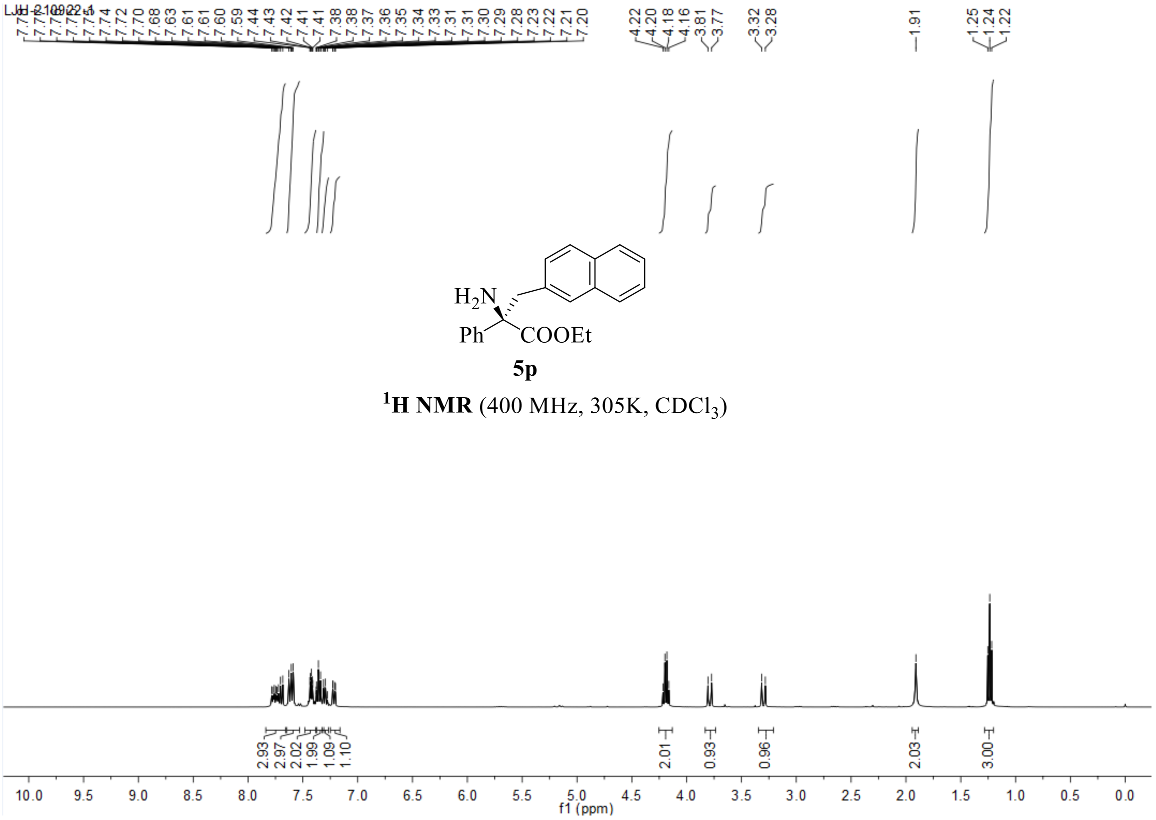

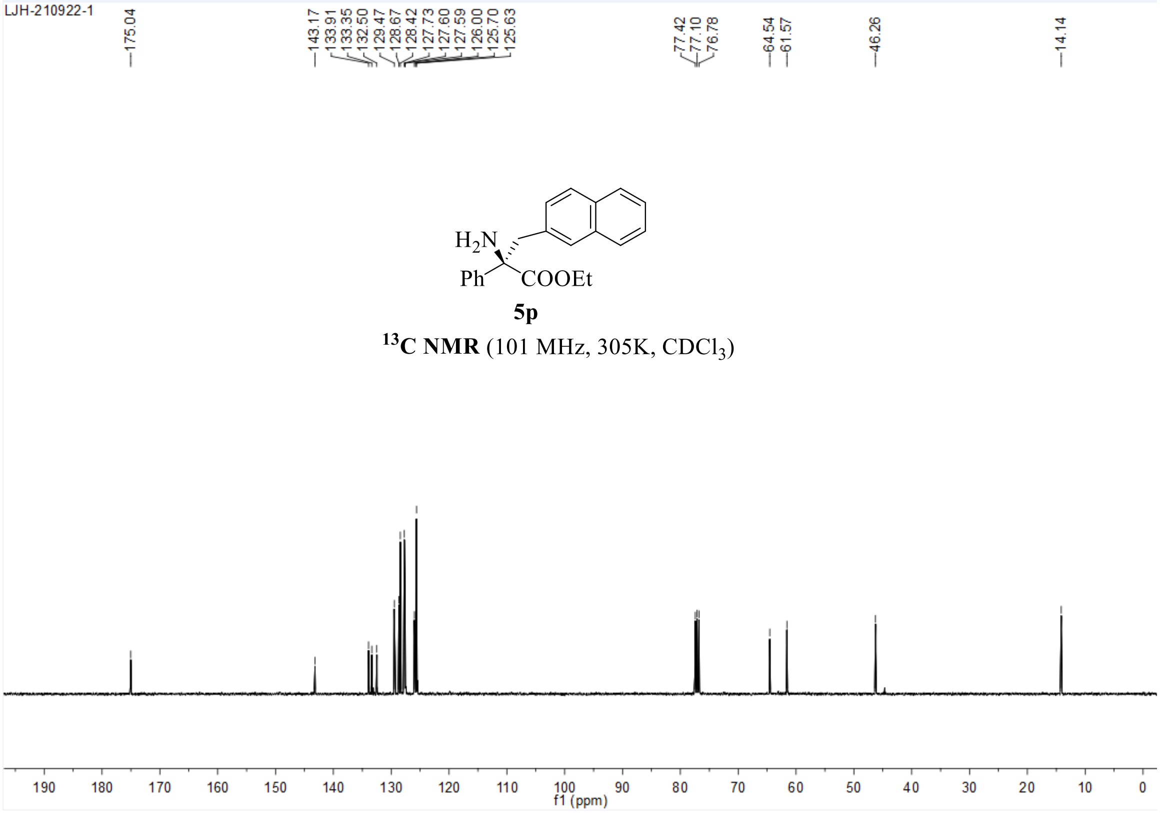

Supplementary Figure 21: NMR of compound 5p.

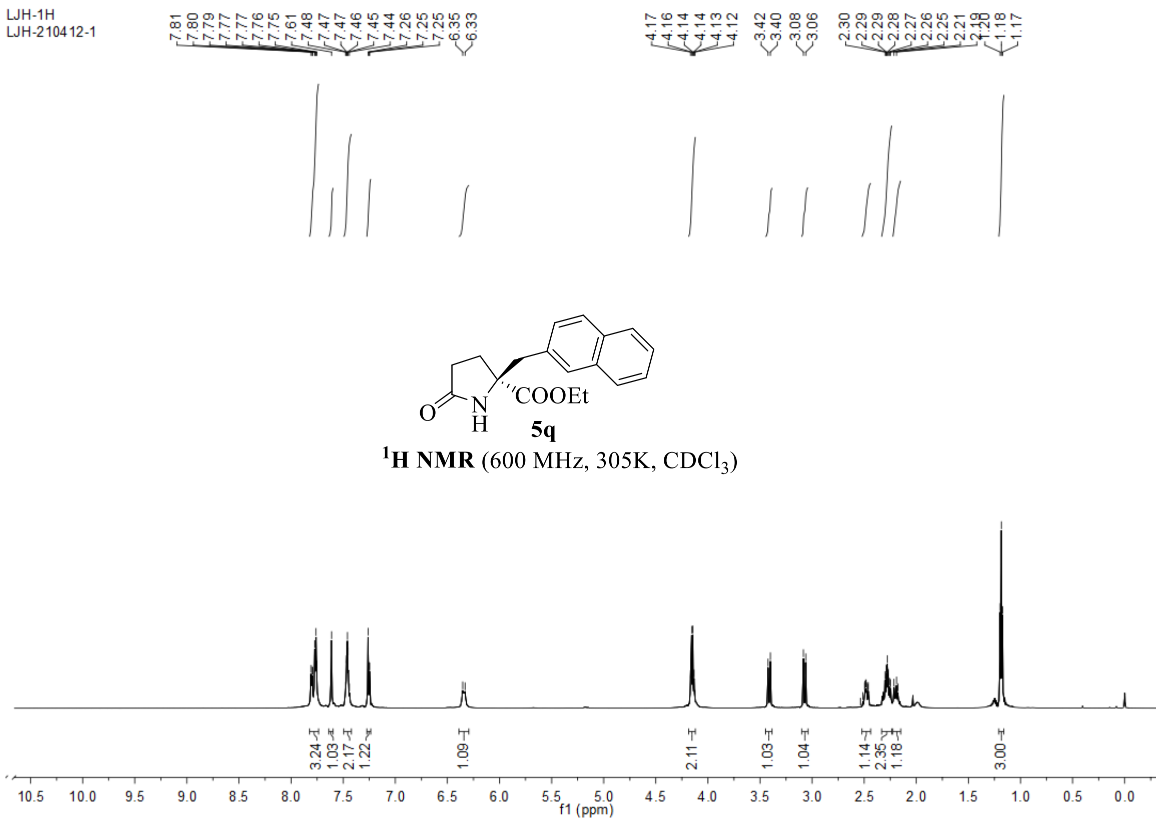

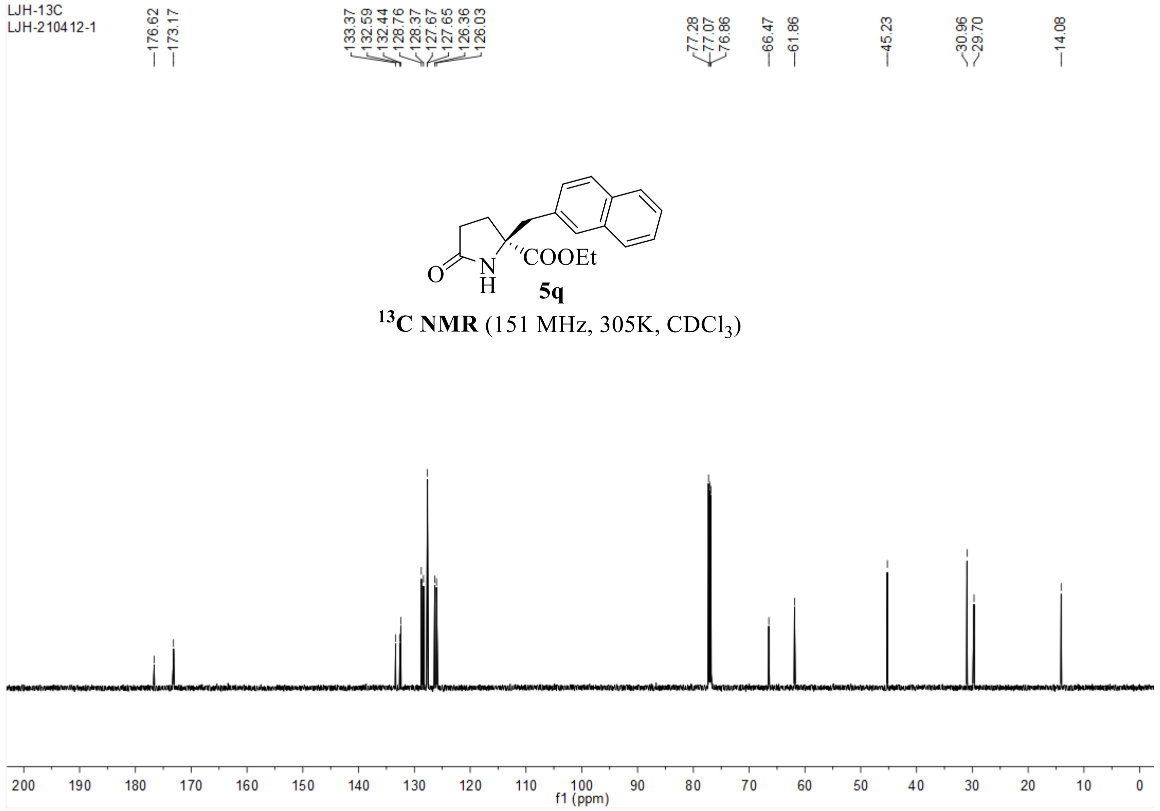

Supplementary Figure 22: NMR of compound 5q.

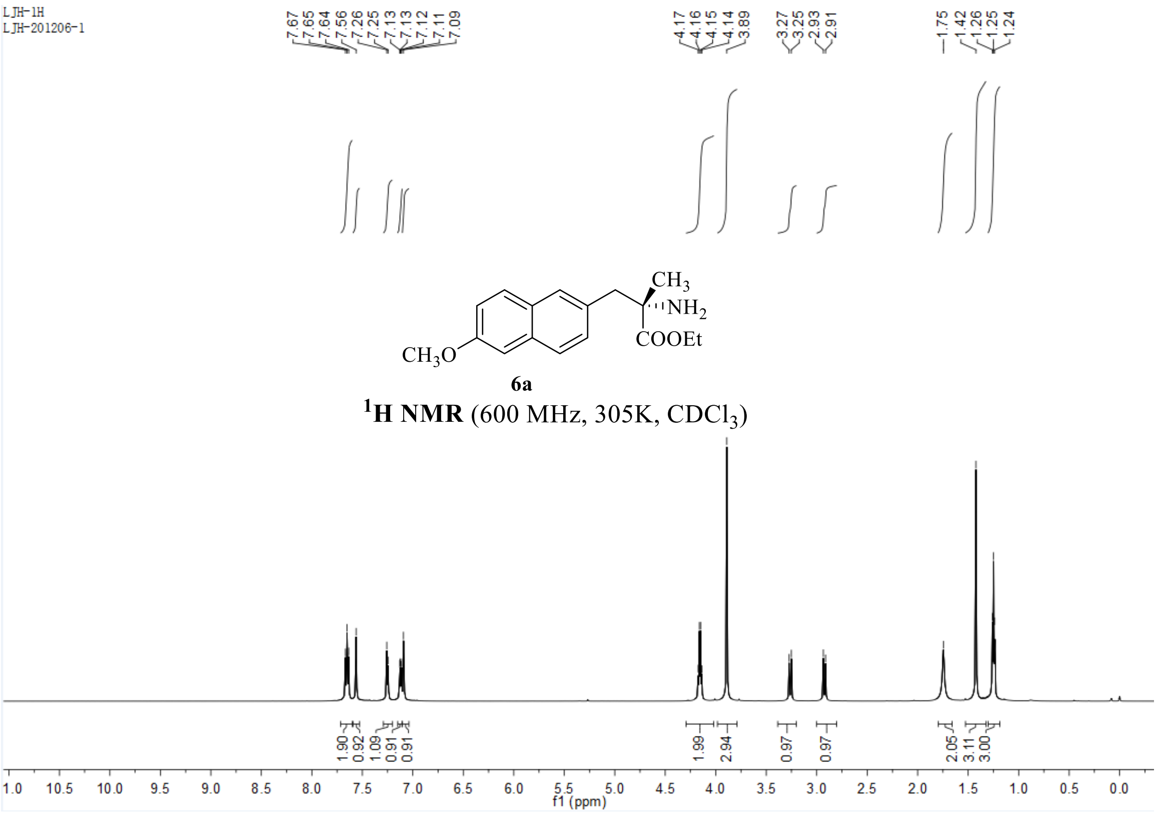

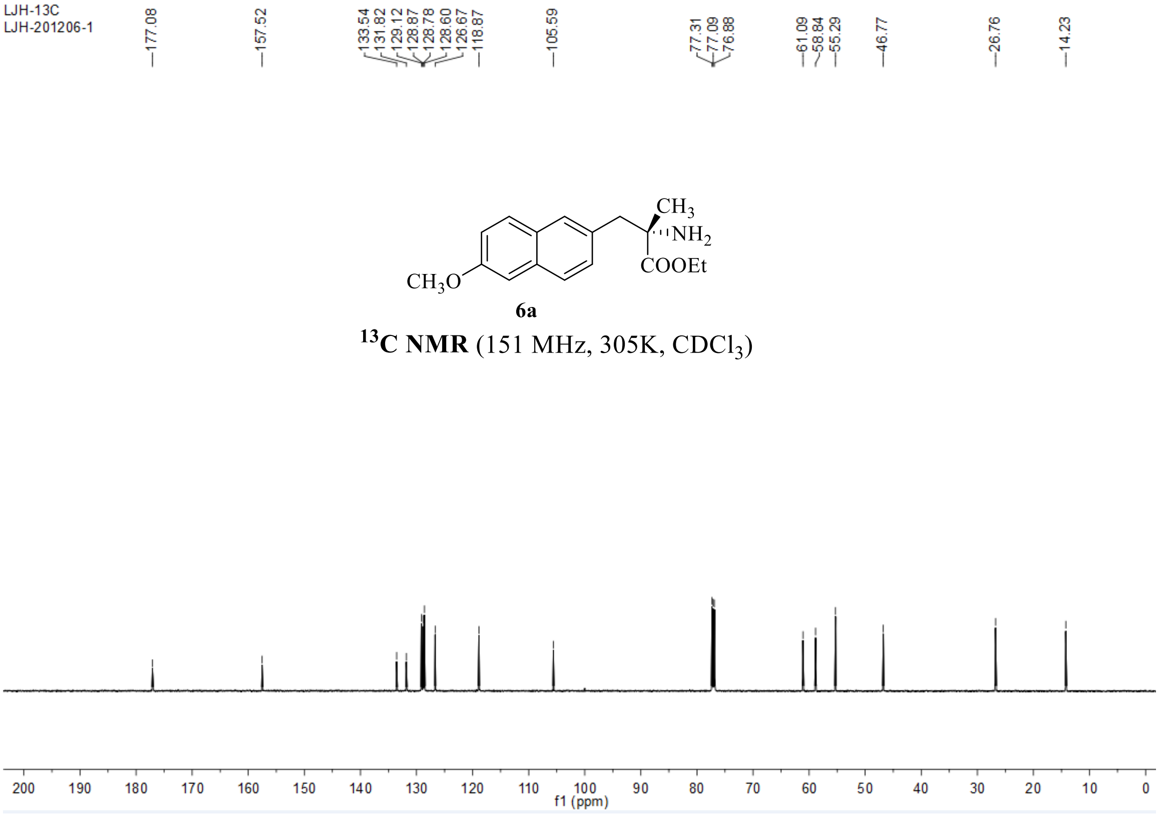

Supplementary Figure 23: NMR of compound 6a.

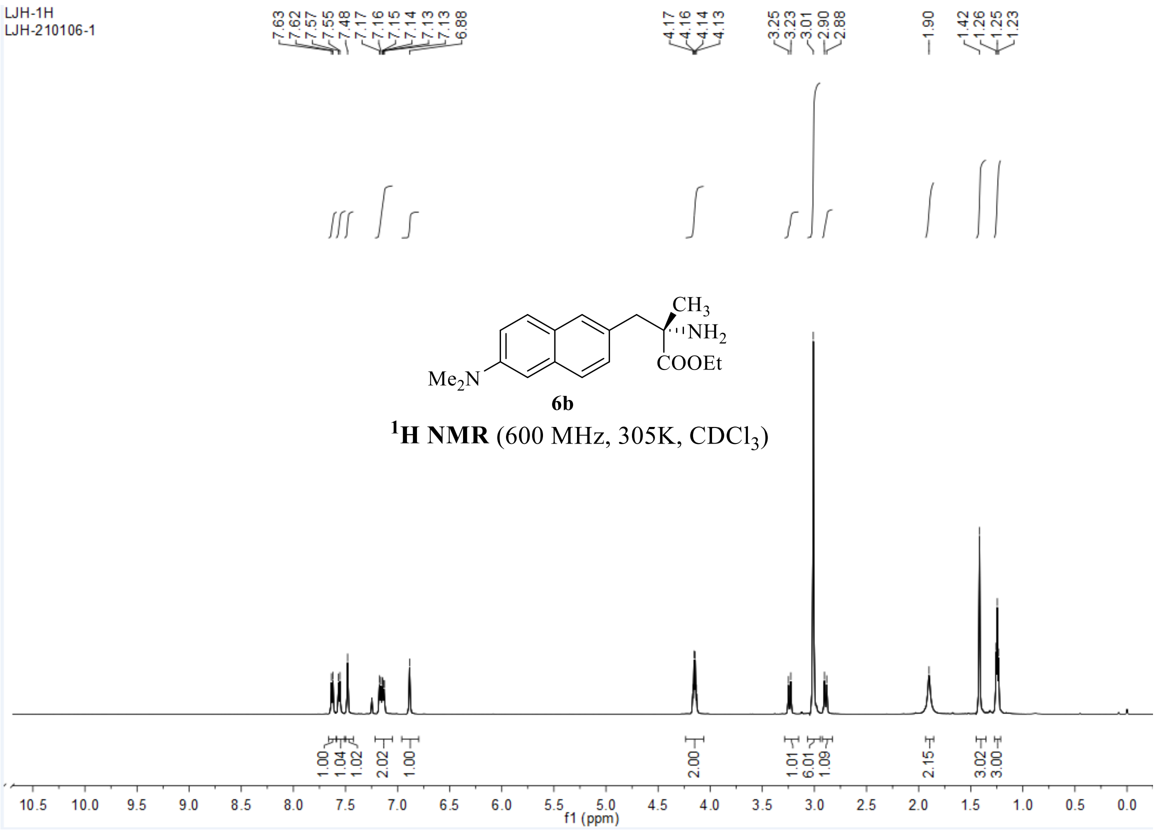

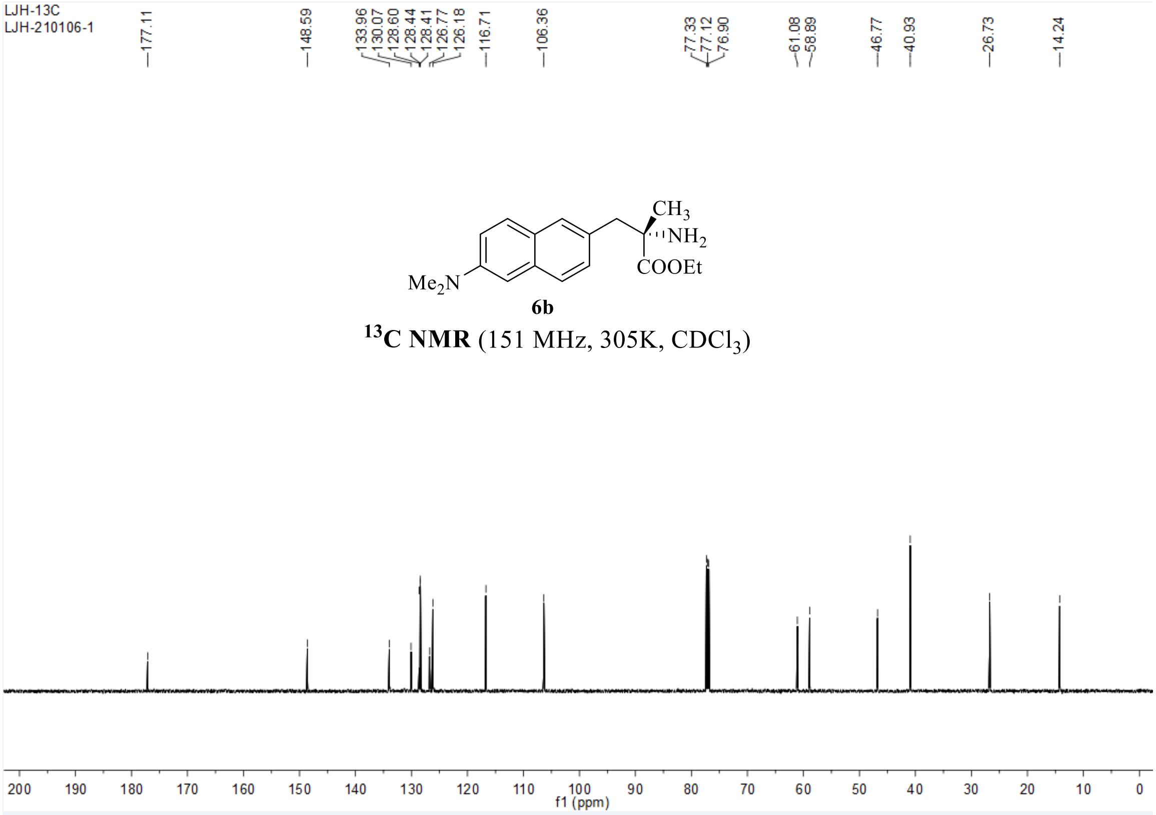

Supplementary Figure 24: NMR of compound 6b.

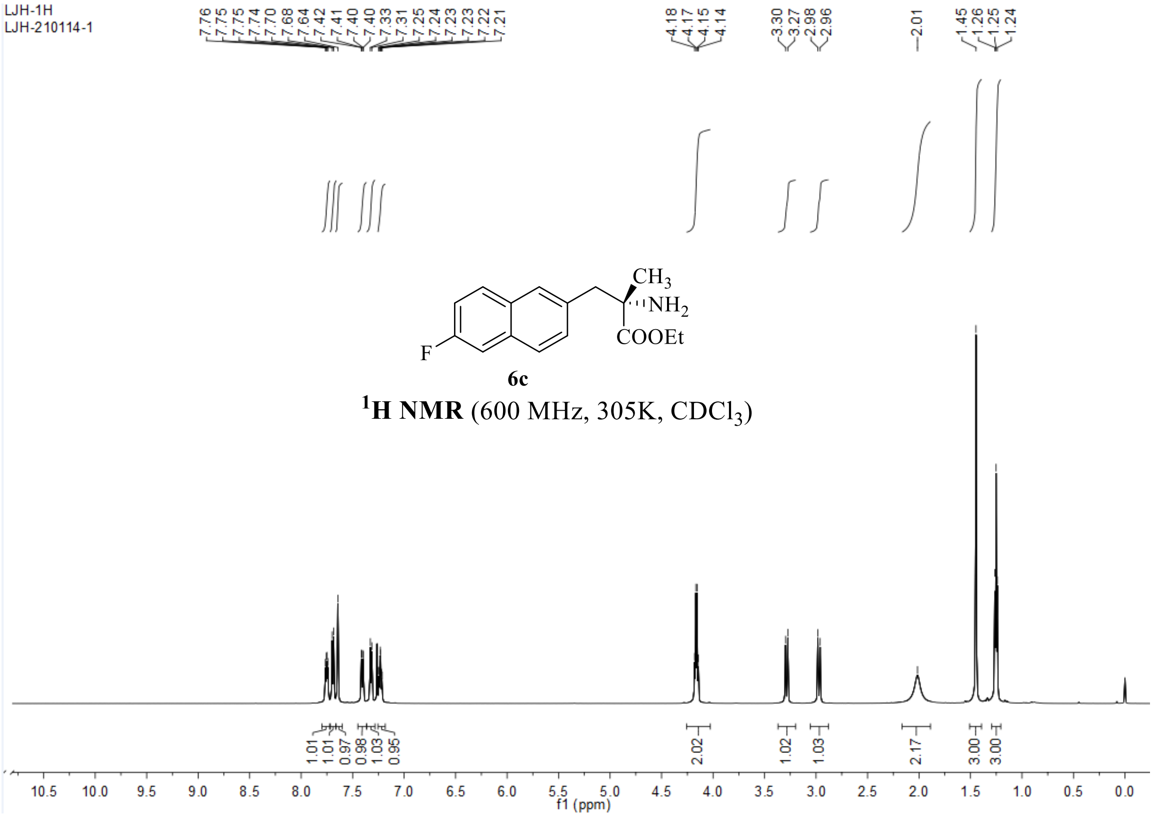

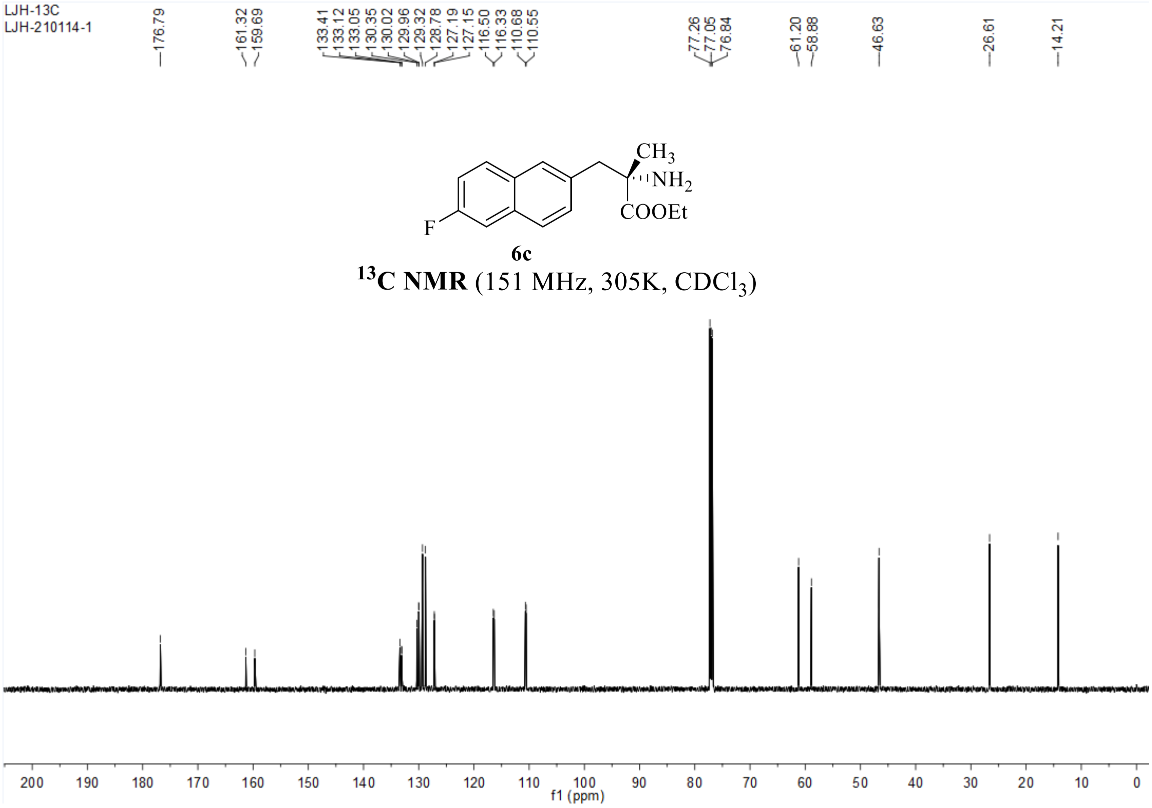

Supplementary Figure 25: NMR of compound 6c.

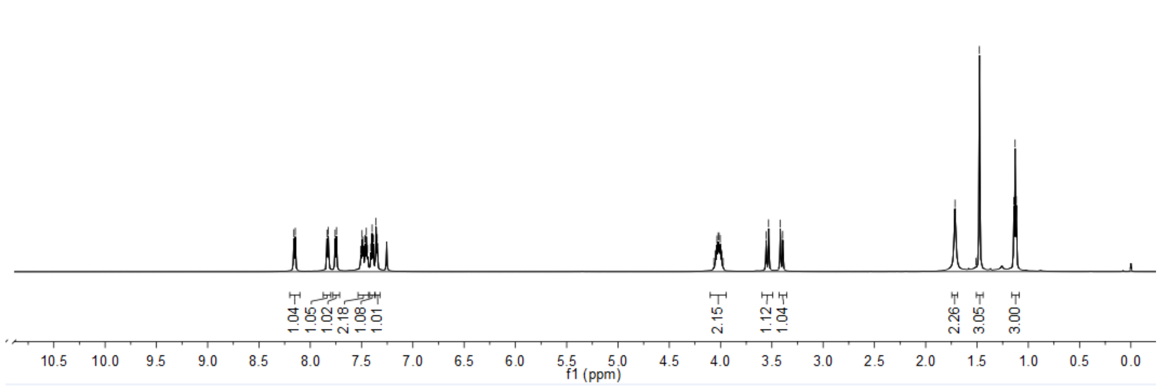

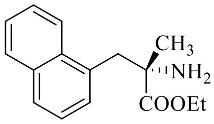

6d

^{1}H NMR (600 MHz, 305K, $CDCl_3$)

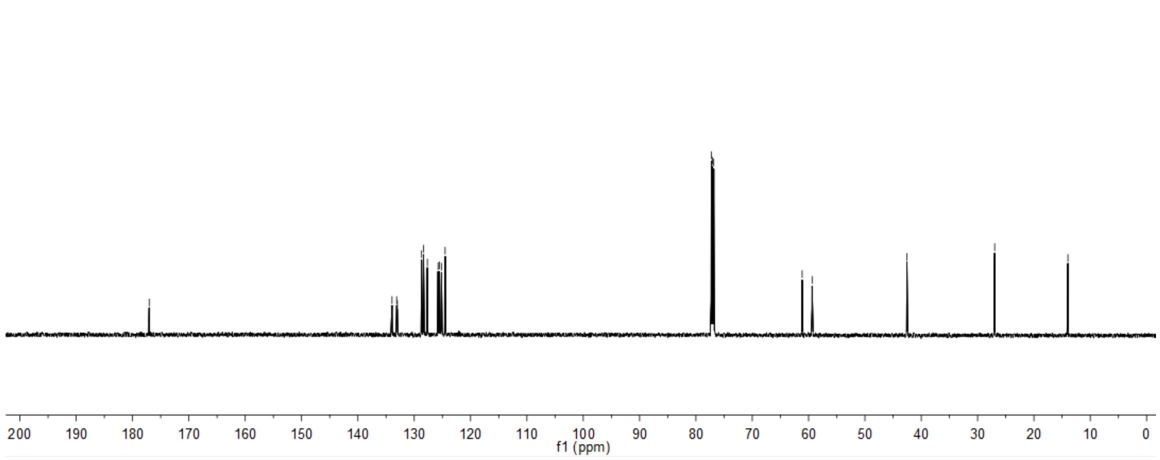

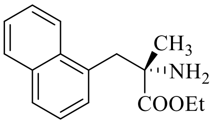

6d

^{13}C NMR (151 MHz, 305K, $CDCl_3$)

Supplementary Figure 26: NMR of compound 6d.

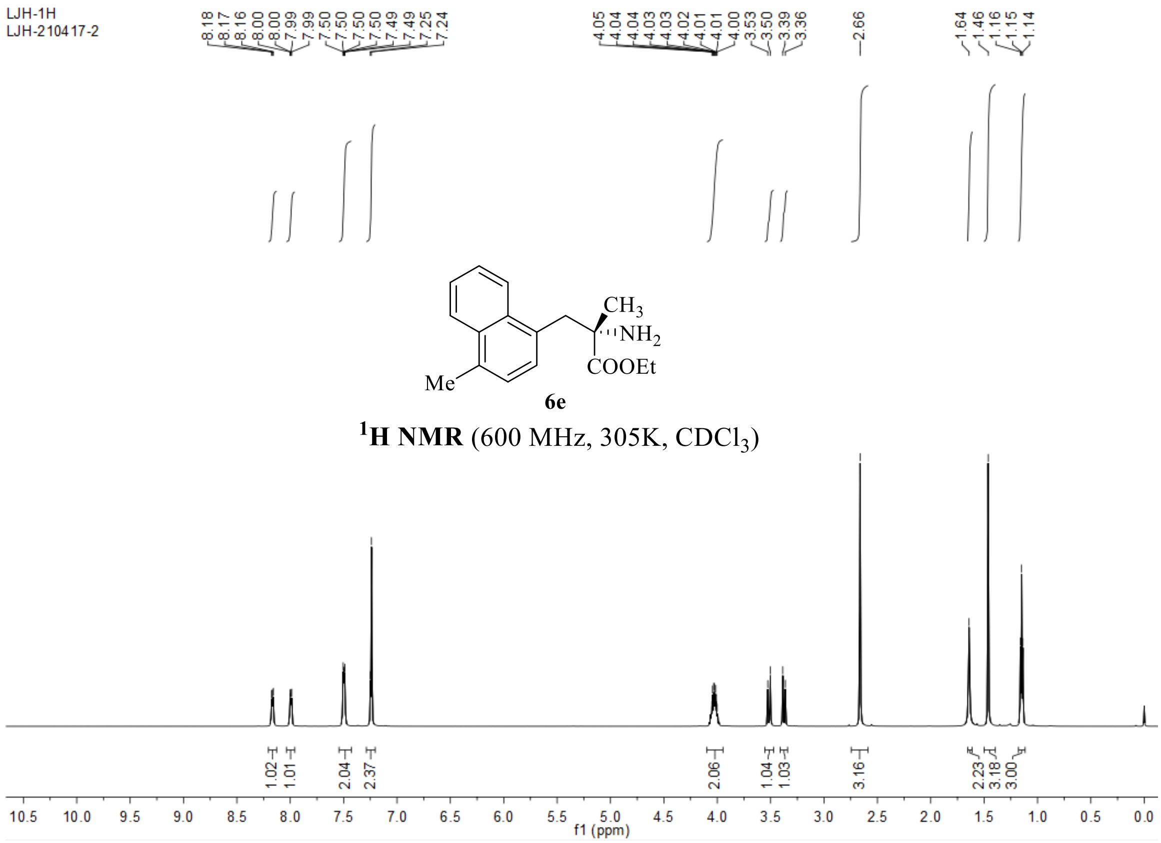

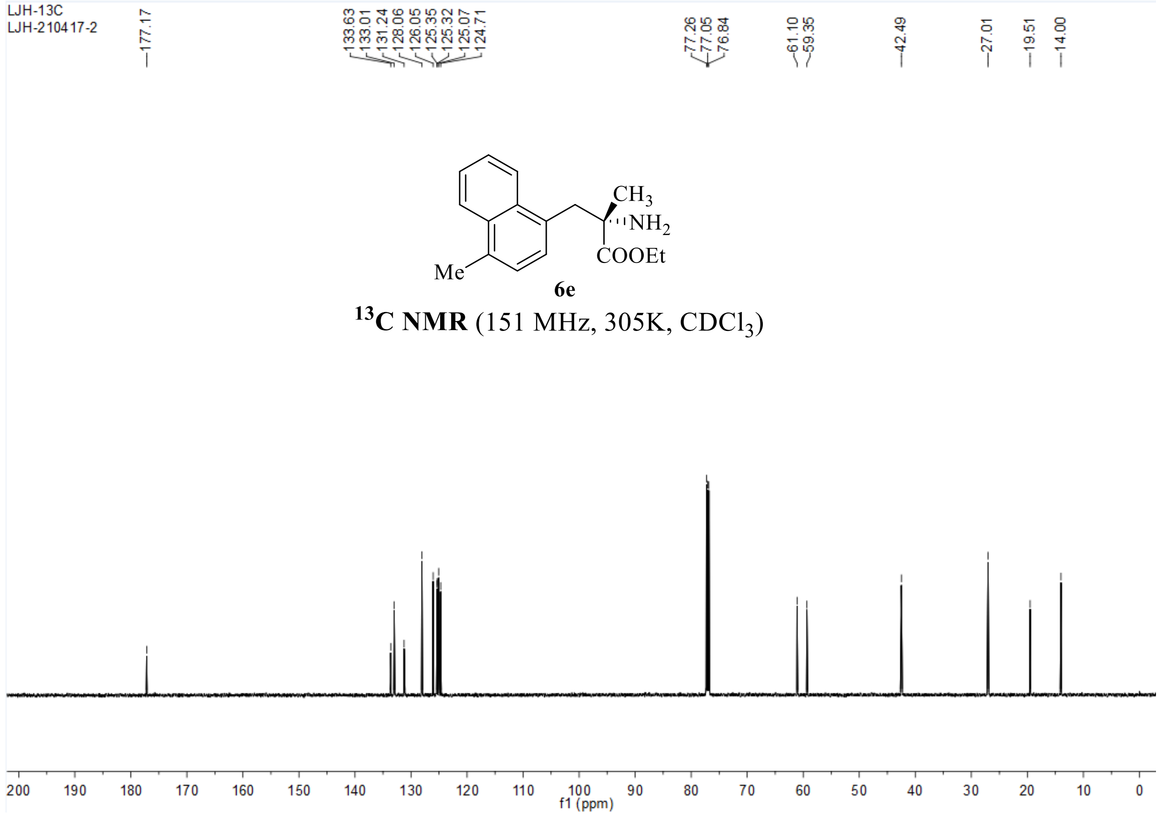

Supplementary Figure 27: NMR of compound 6e.

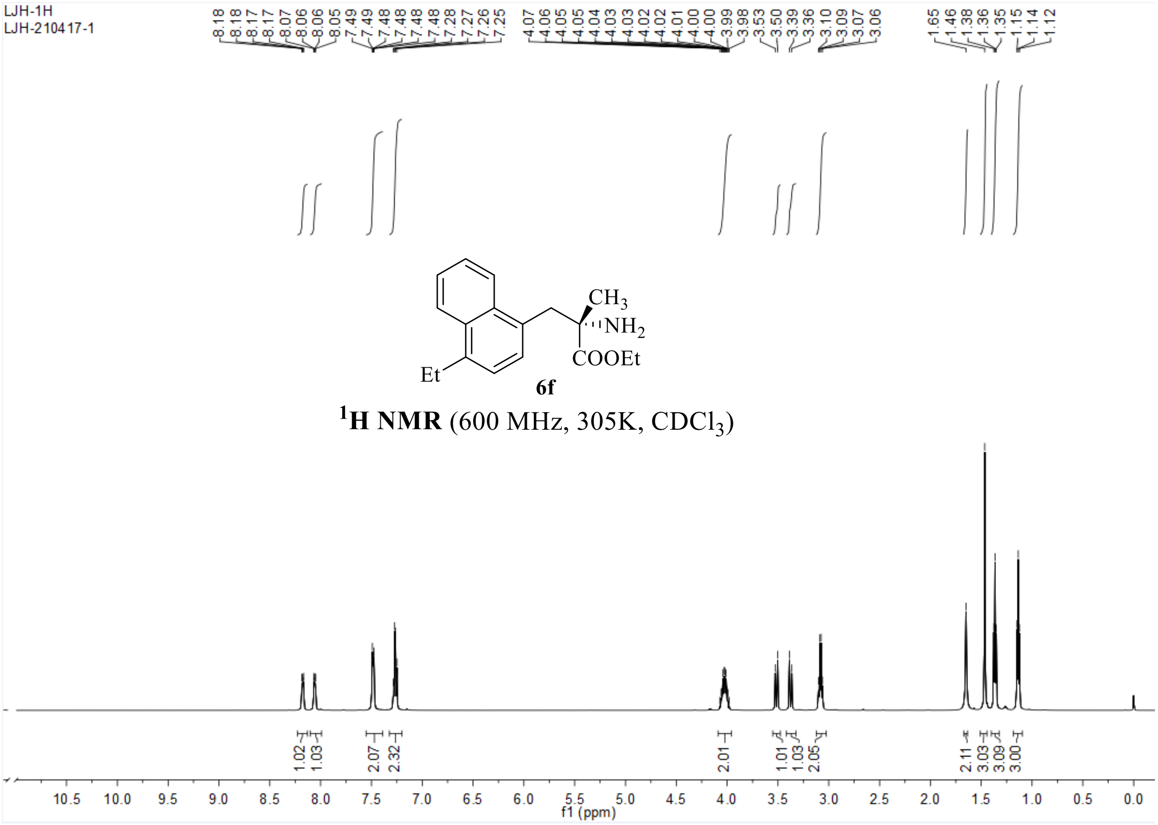

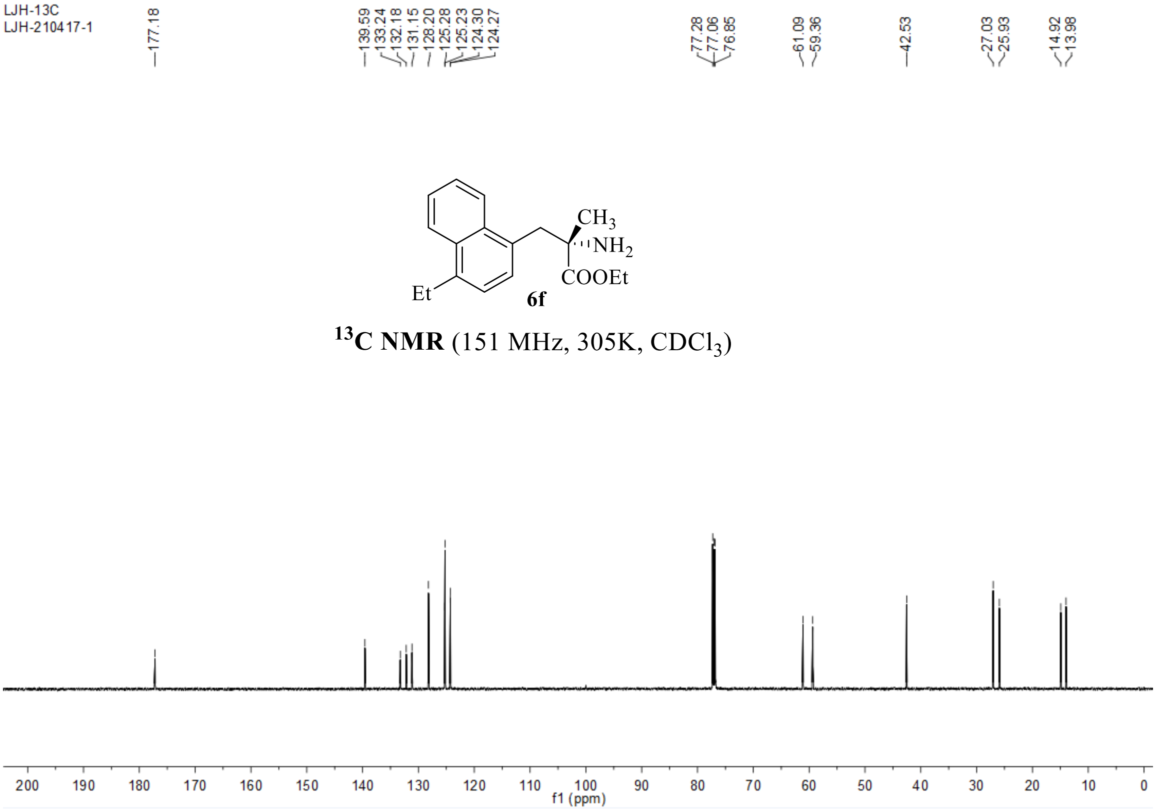

Supplementary Figure 28: NMR of compound 6f.

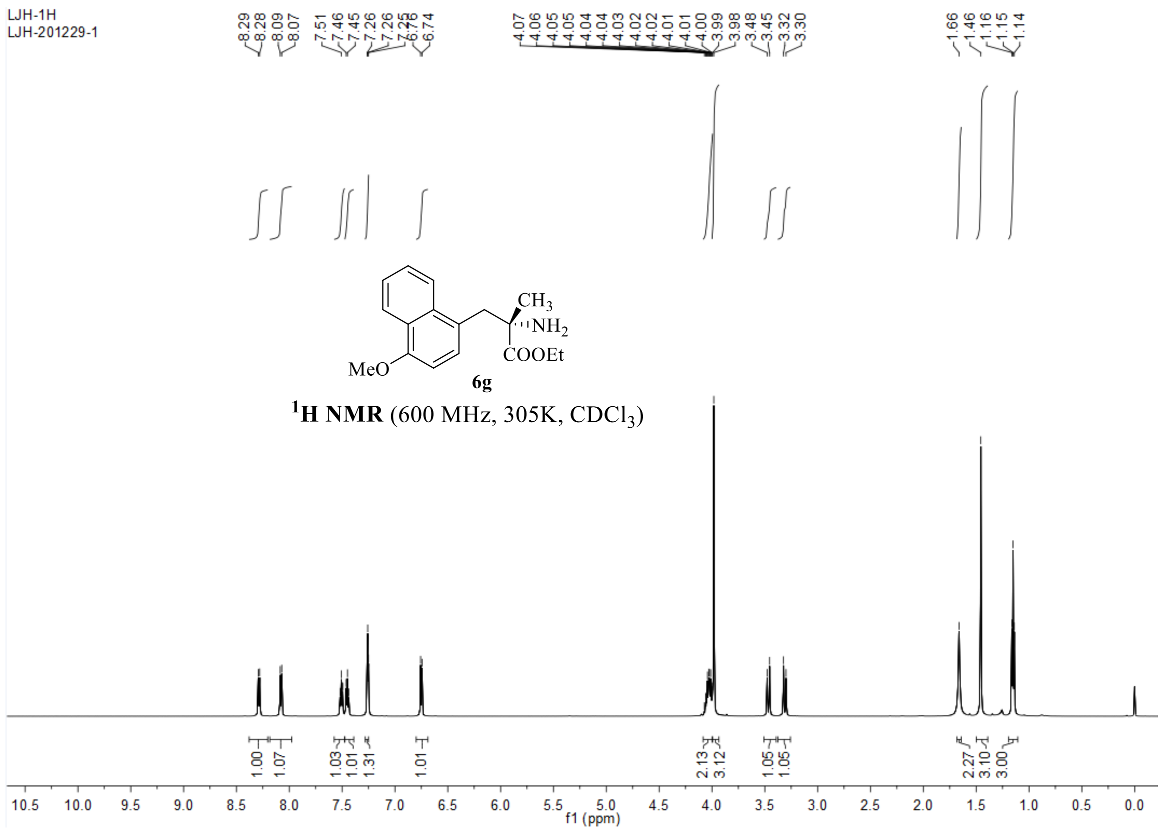

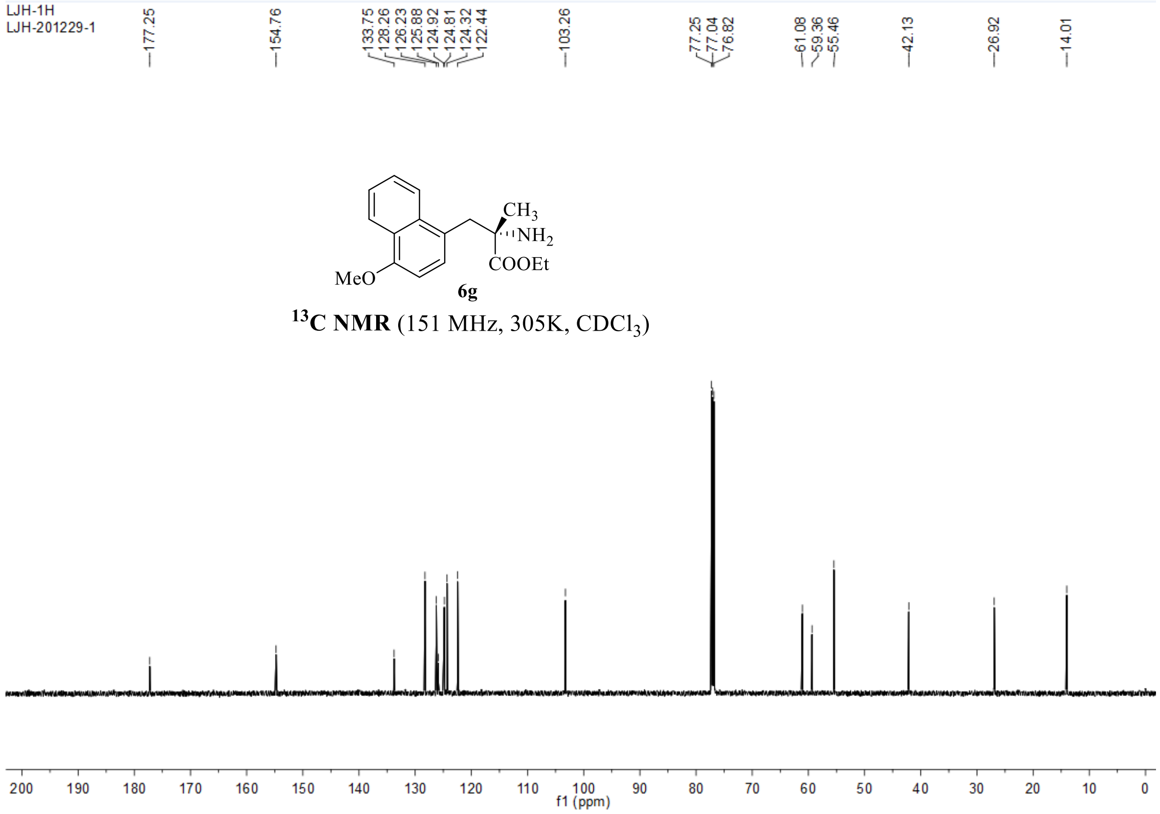

Supplementary Figure 29: NMR of compound 6g.

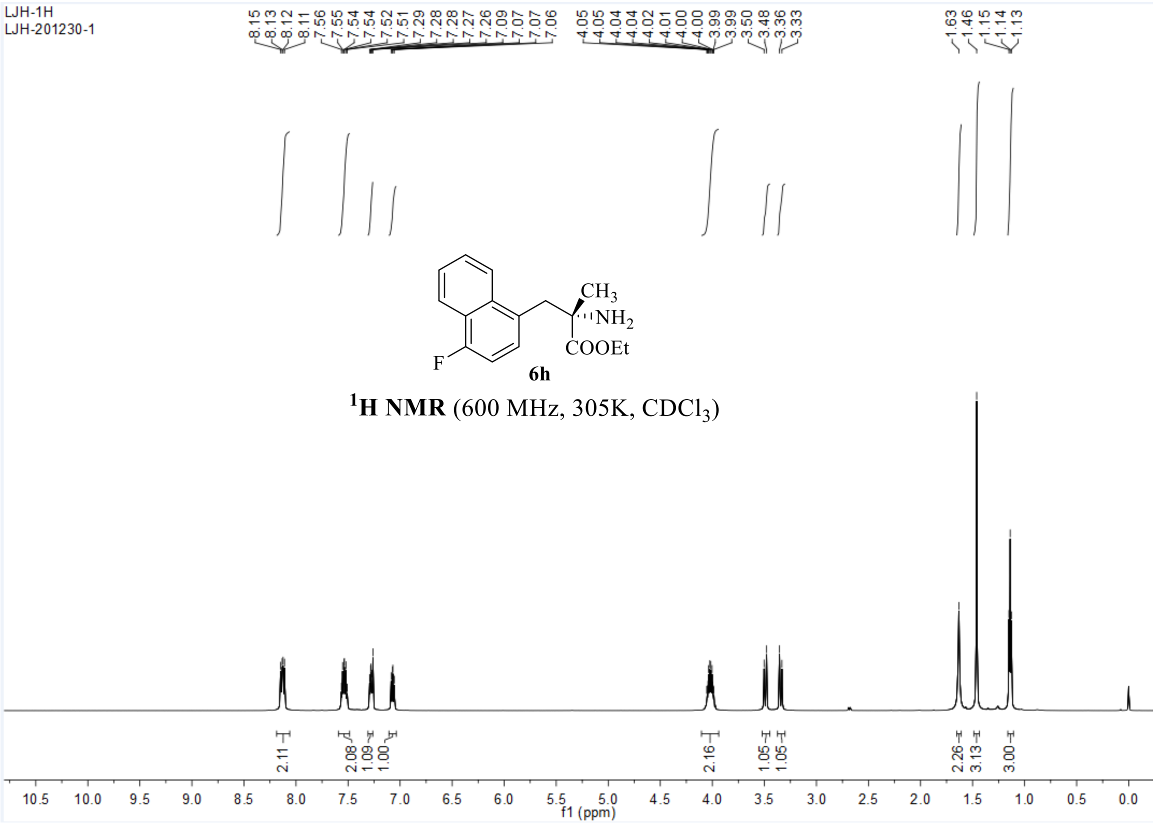

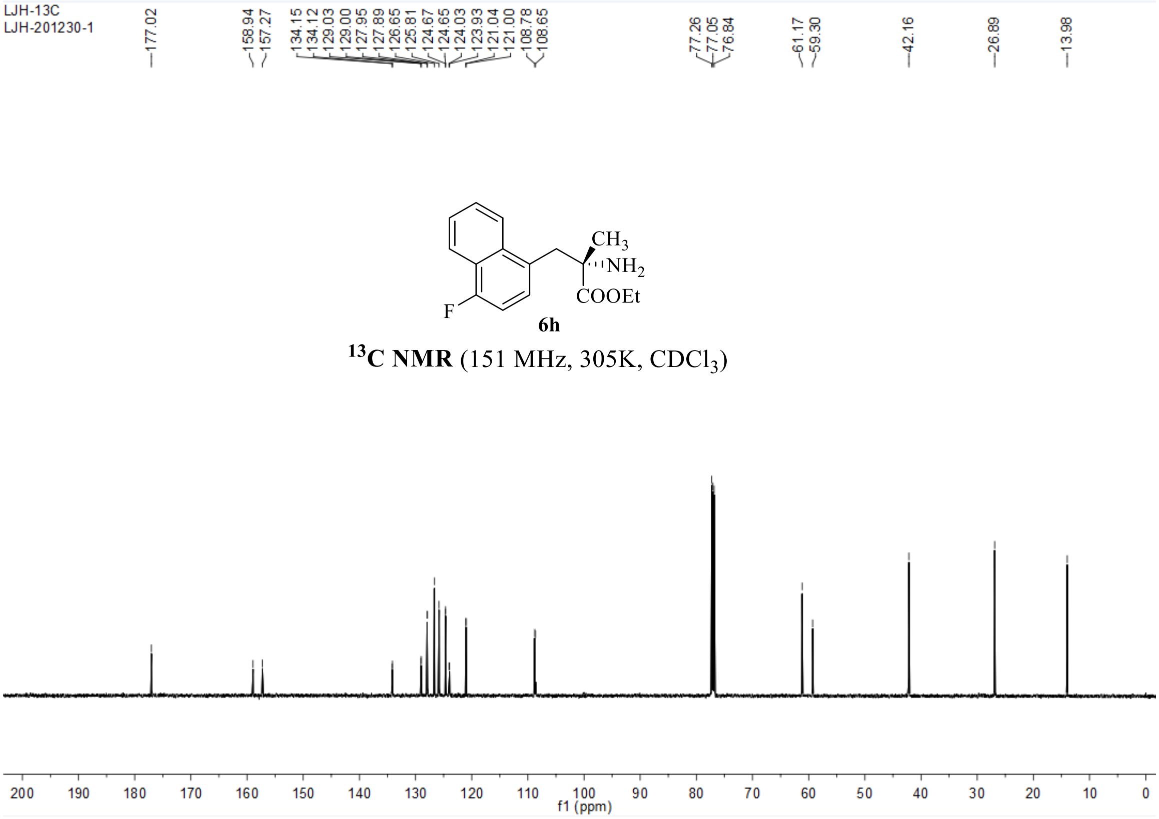

Supplementary Figure 30: NMR of compound 6h.

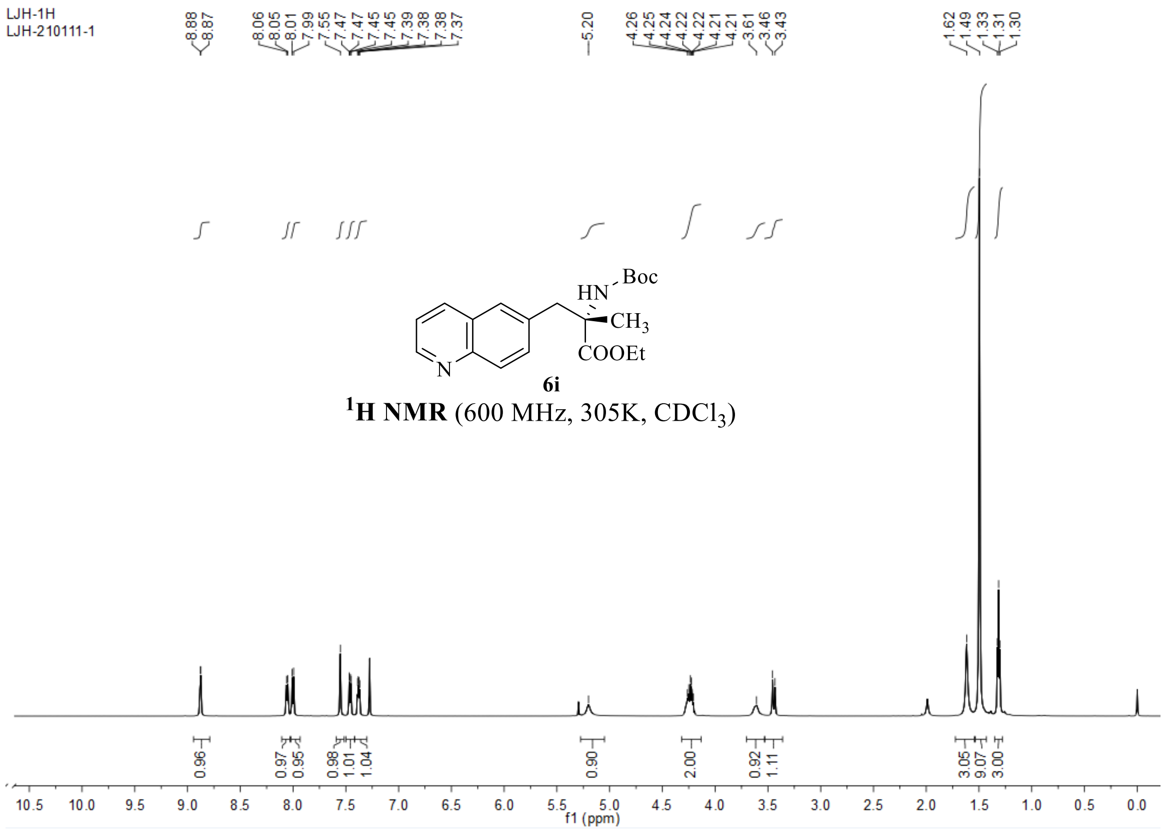

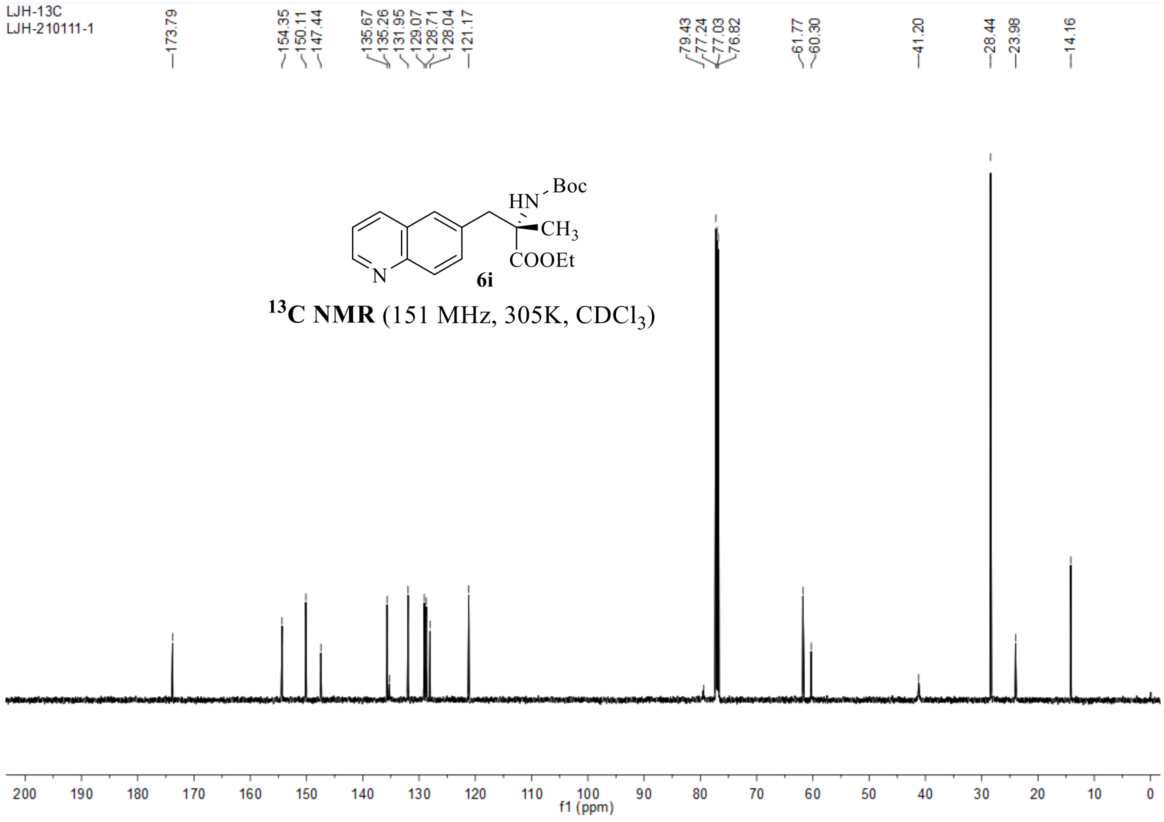

Supplementary Figure 31: NMR of compound 6i.

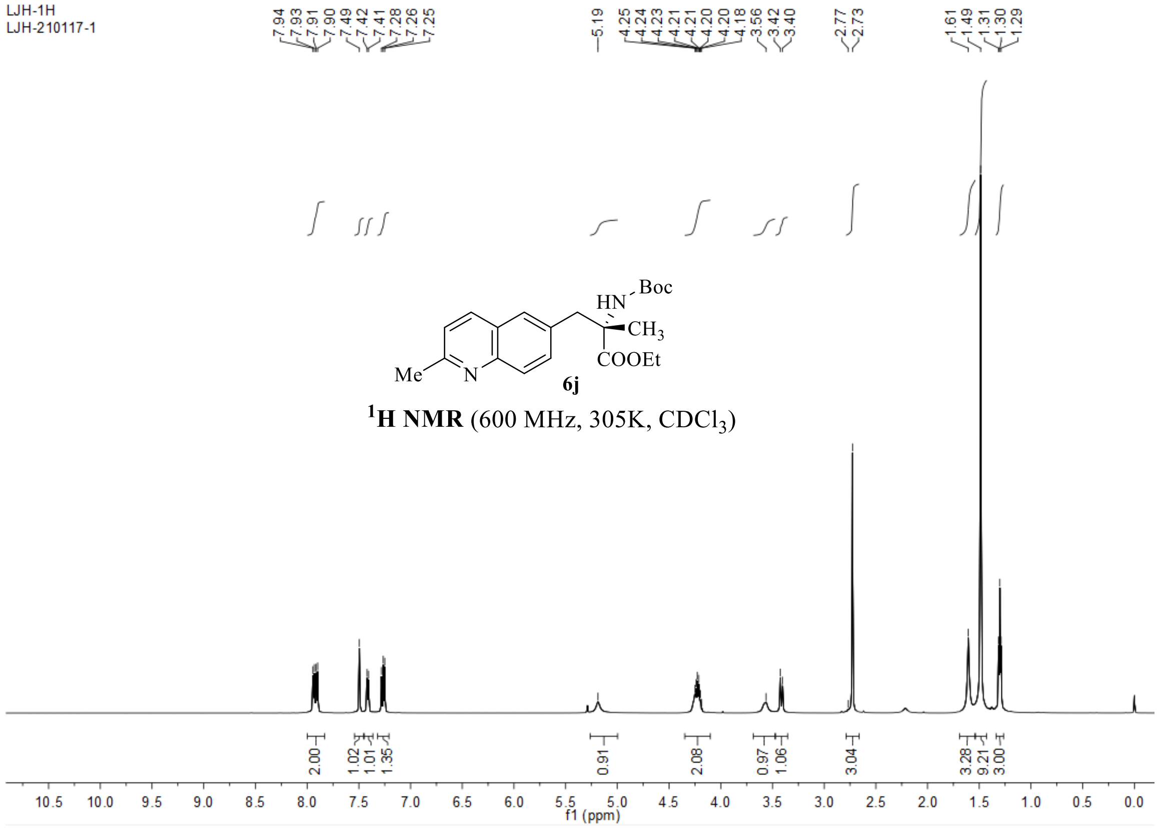

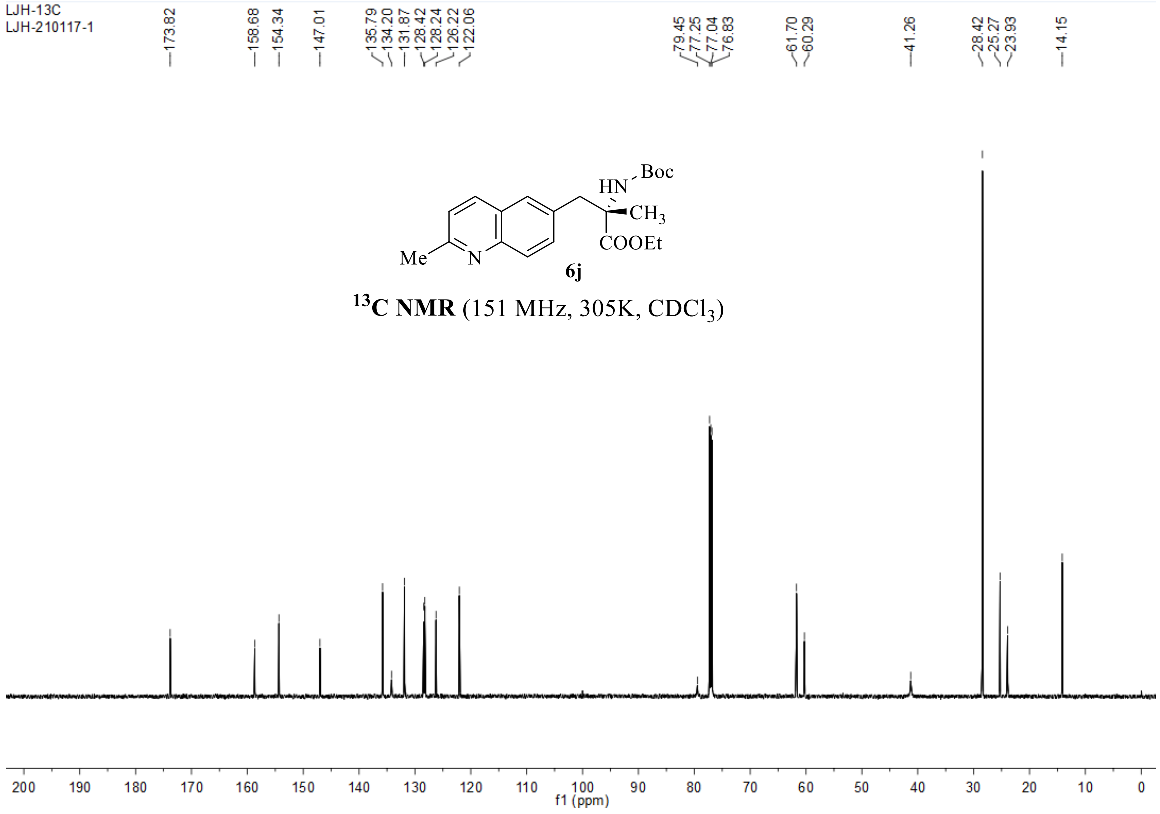

Supplementary Figure 32: NMR of compound 6j.

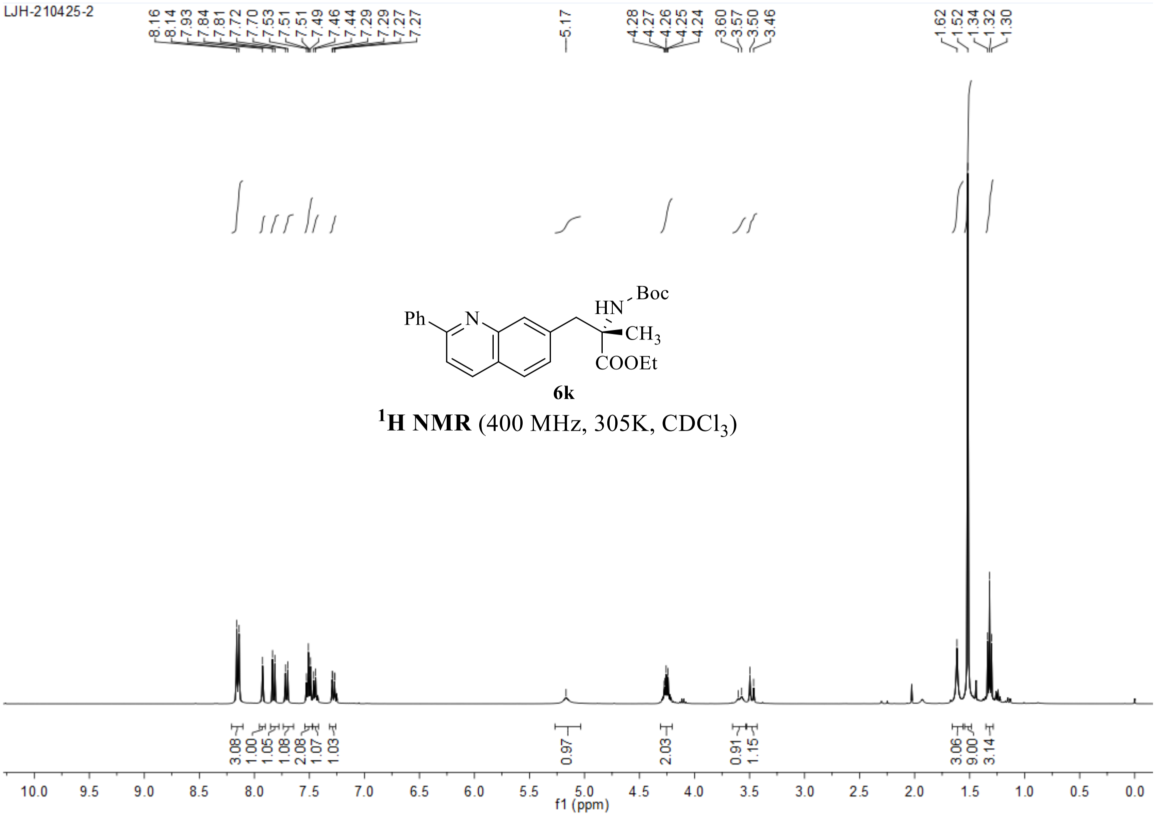

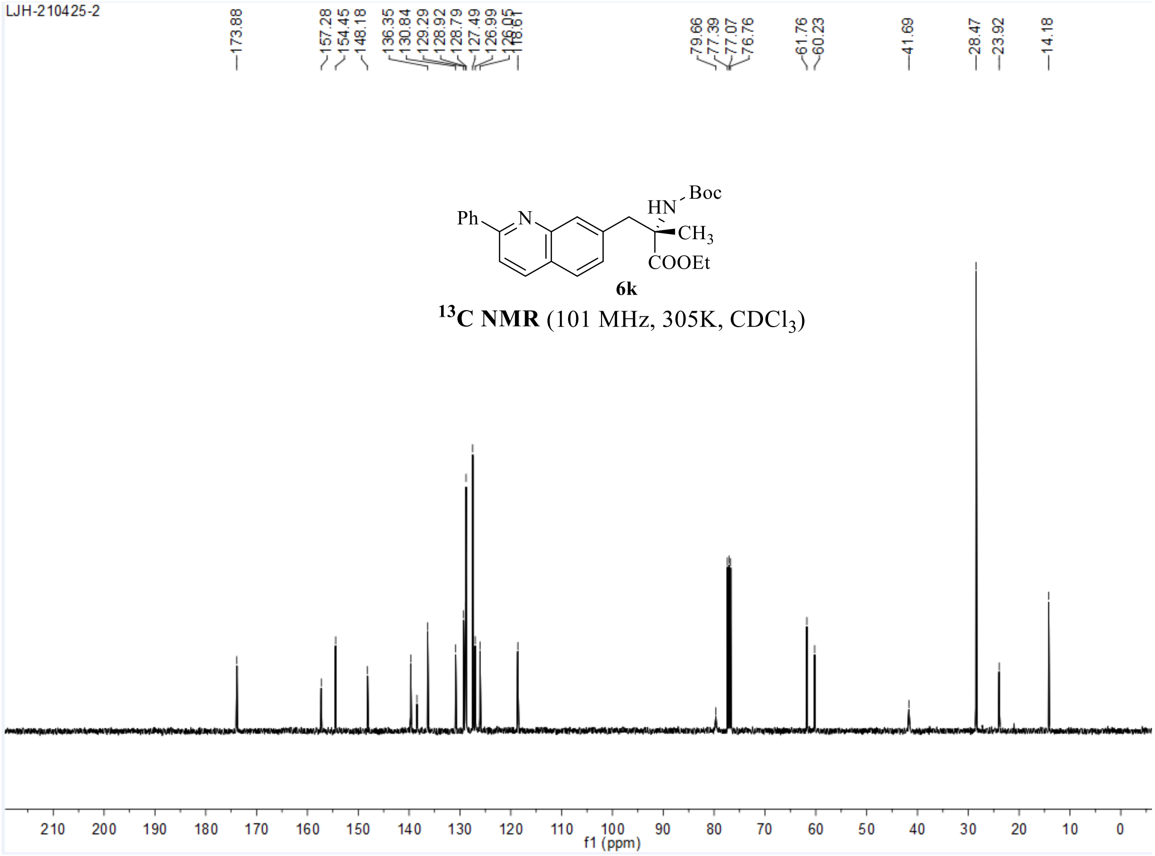

Supplementary Figure 33: NMR of compound 6k.

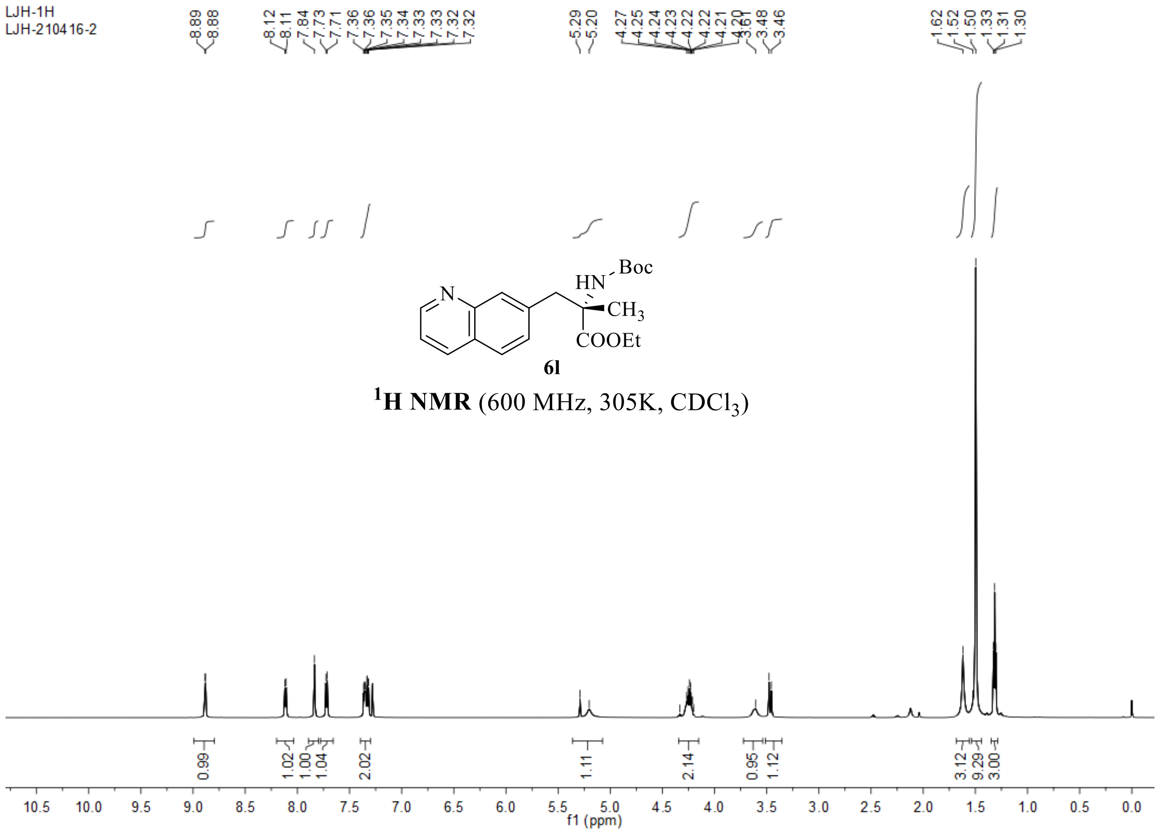

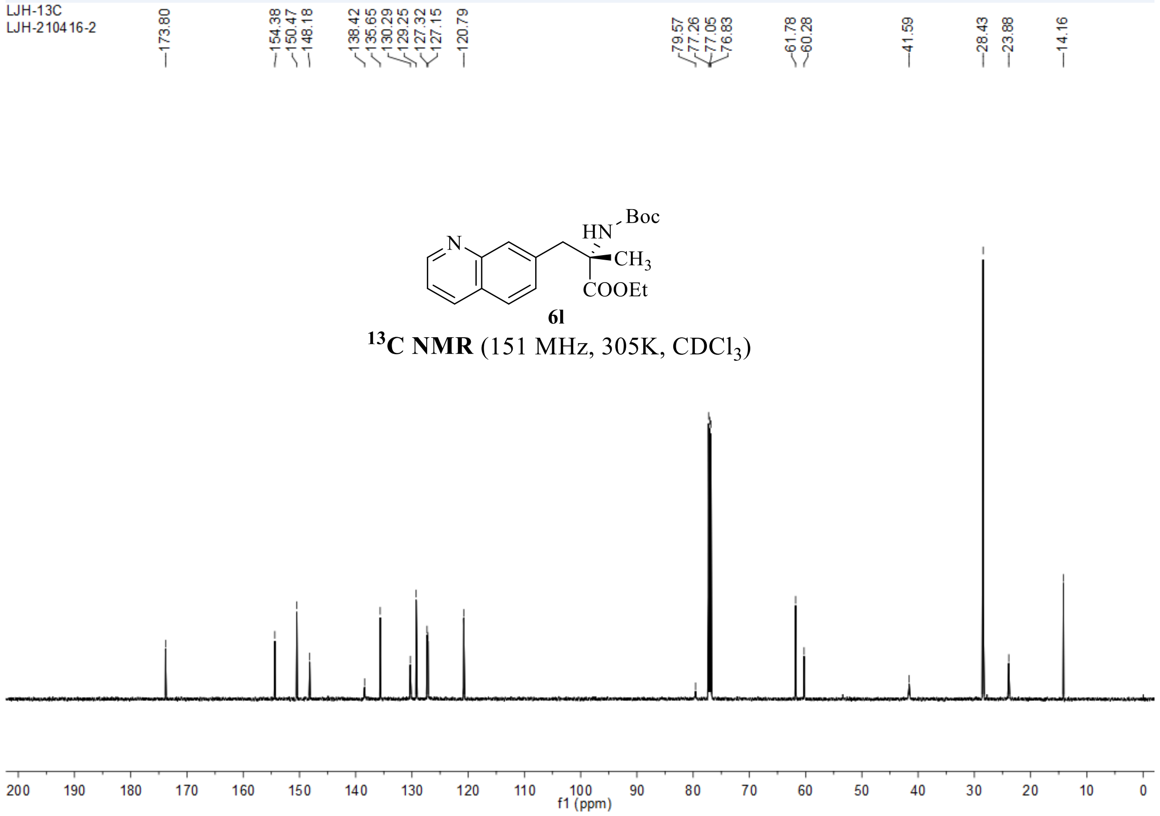

Supplementary Figure 34: NMR of compound 6l.

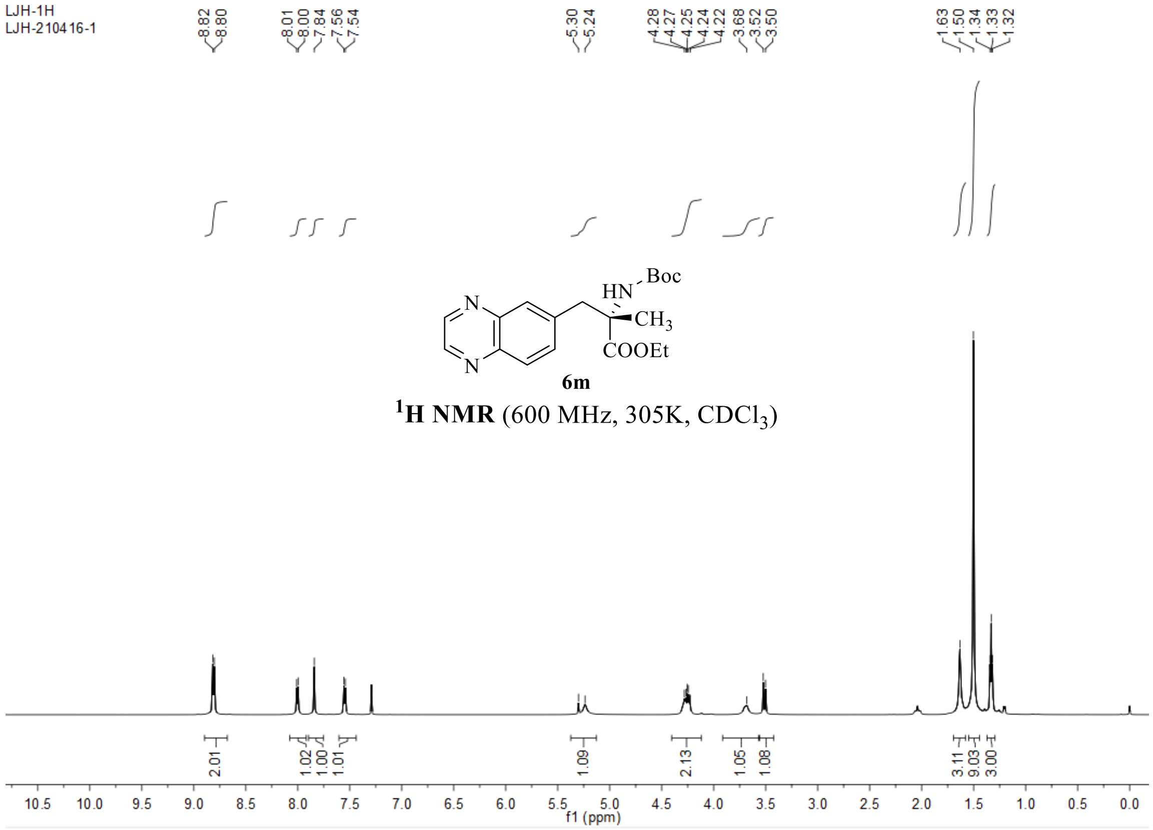

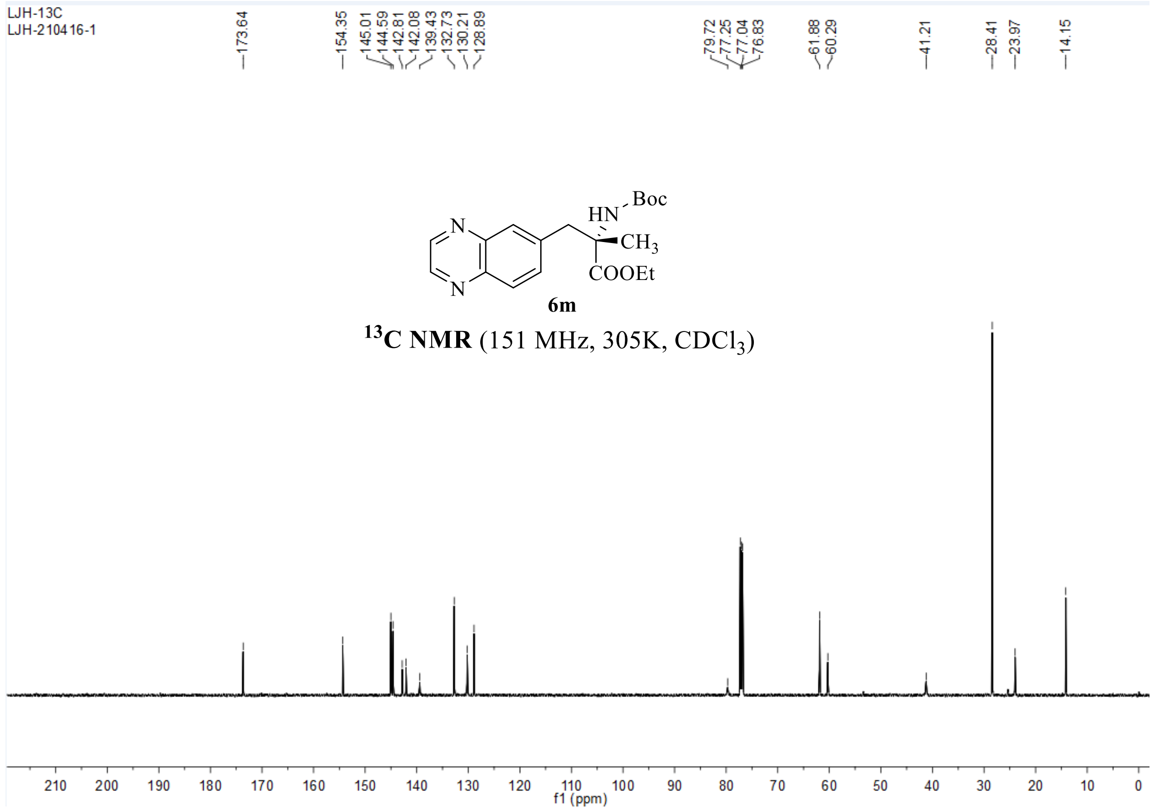

Supplementary Figure 35: NMR of compound 6m.

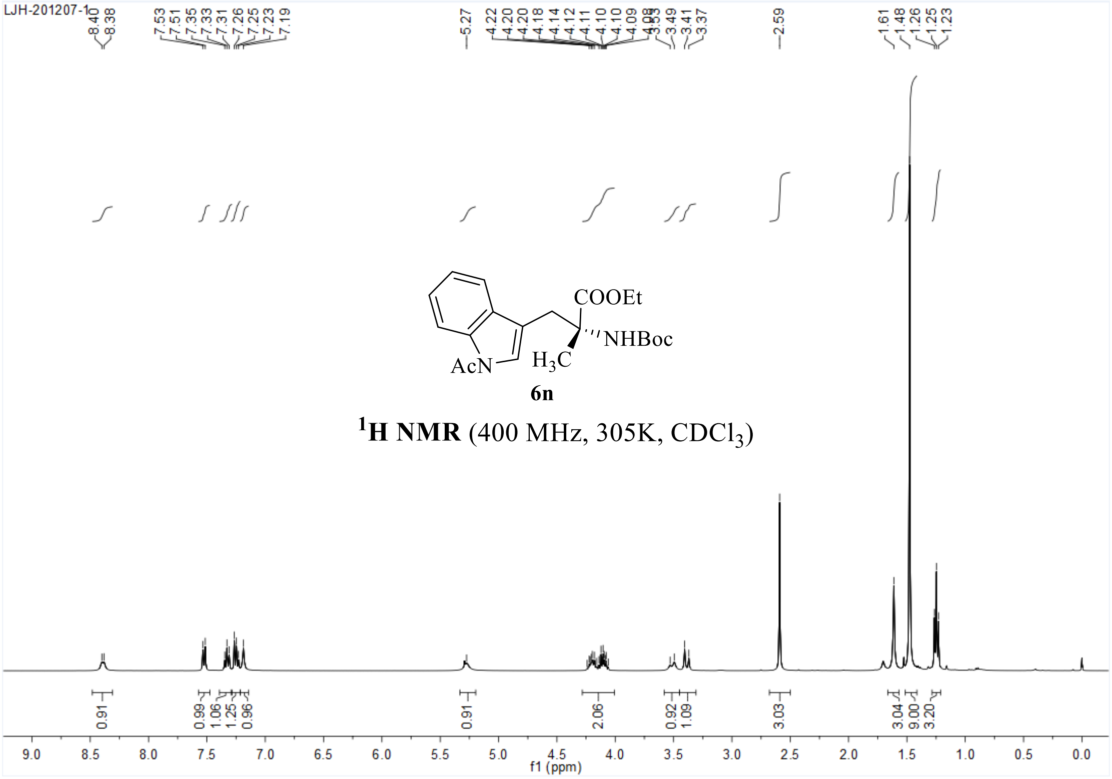

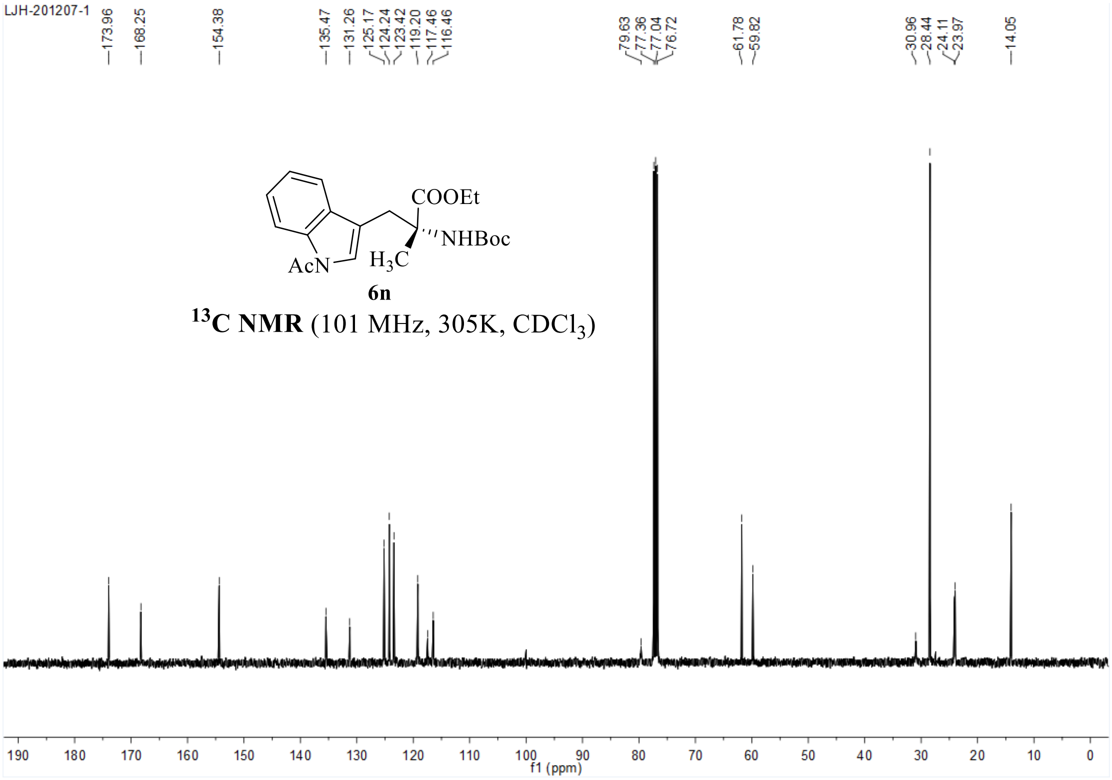

Supplementary Figure 36: NMR of compound 6n.

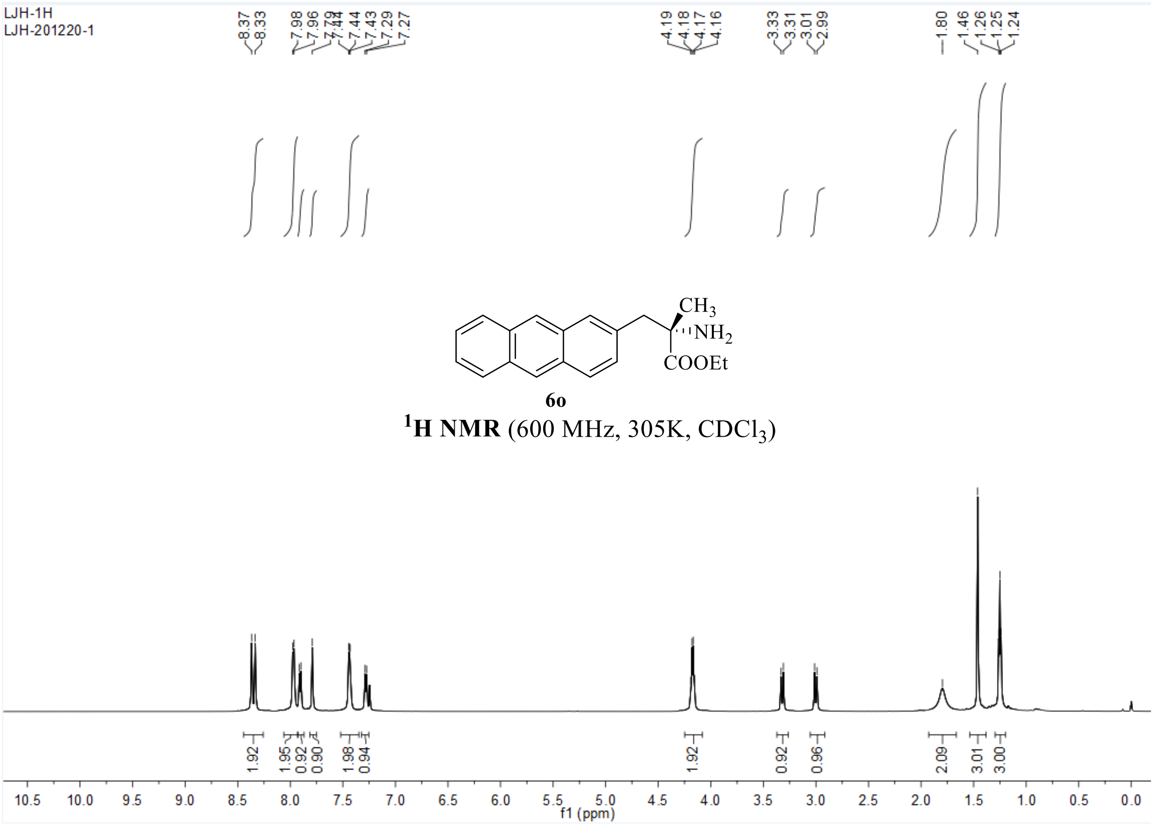

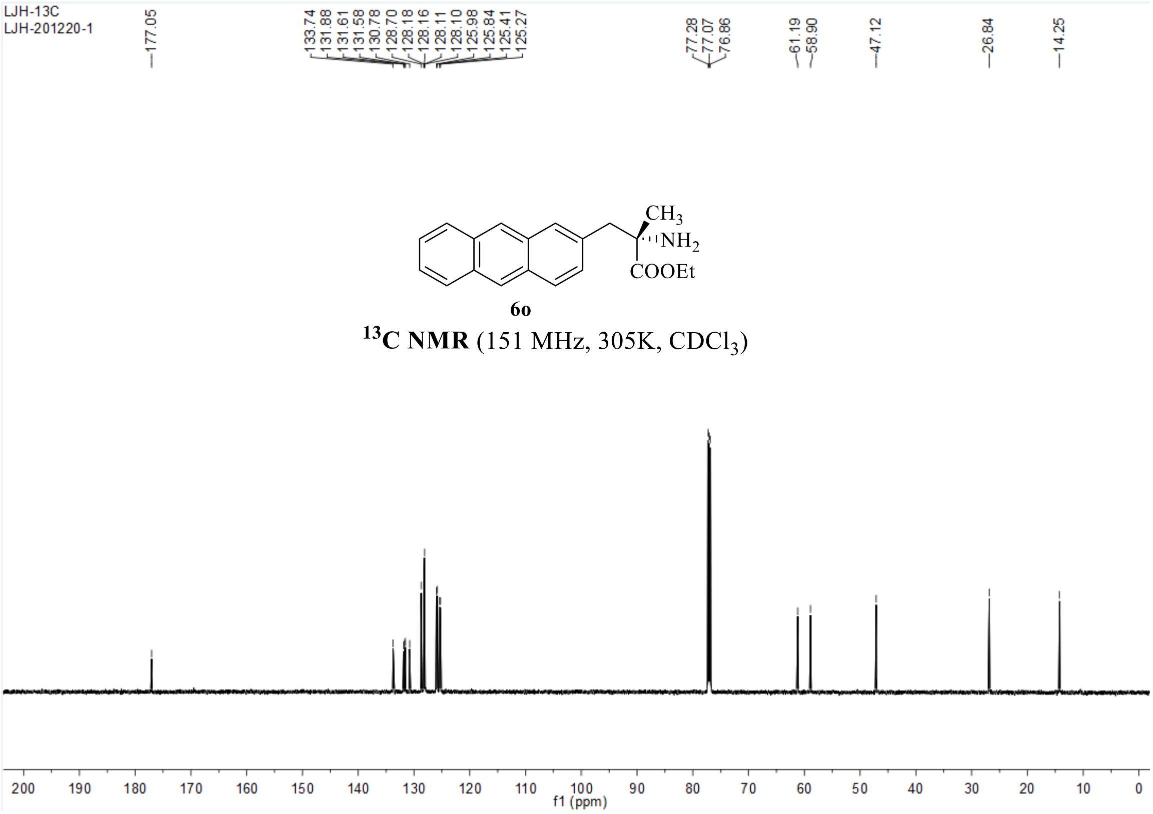

Supplementary Figure 37: NMR of compound 6o.

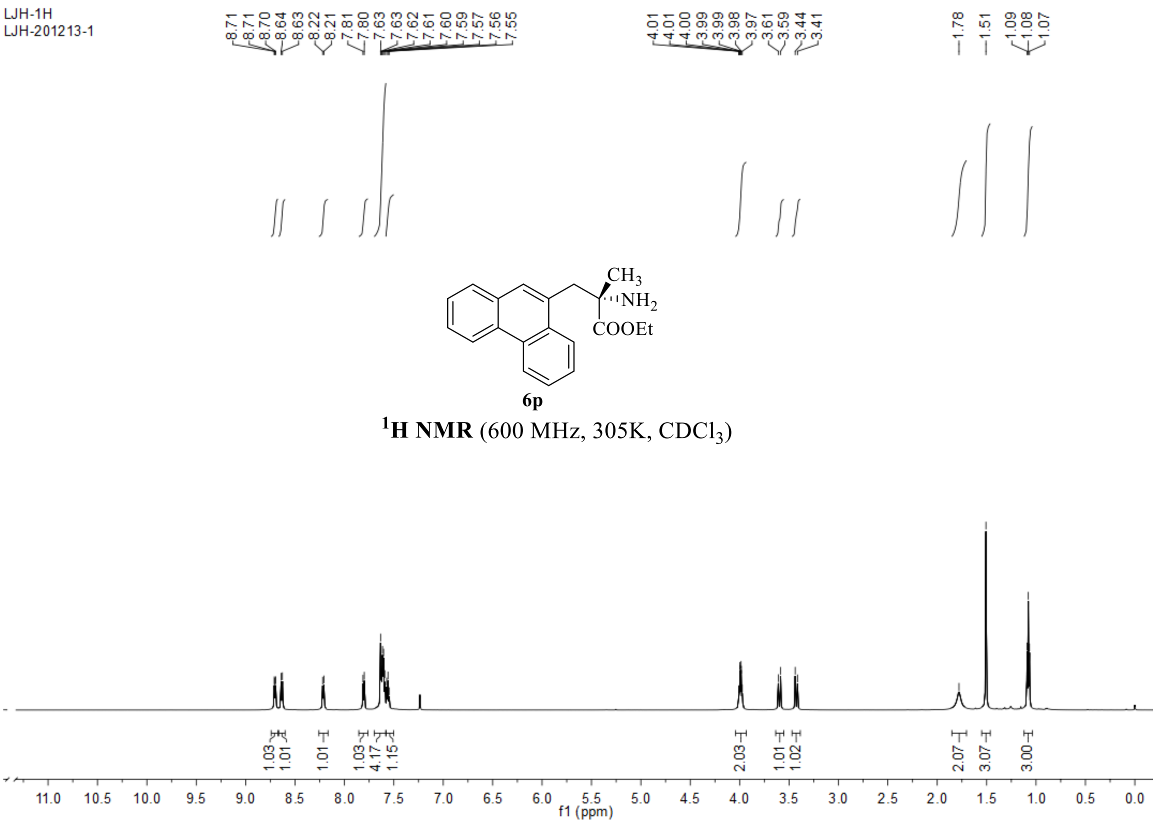

6p

^{1}**H NMR** (600 MHz, 305K, $CDCl_3$)

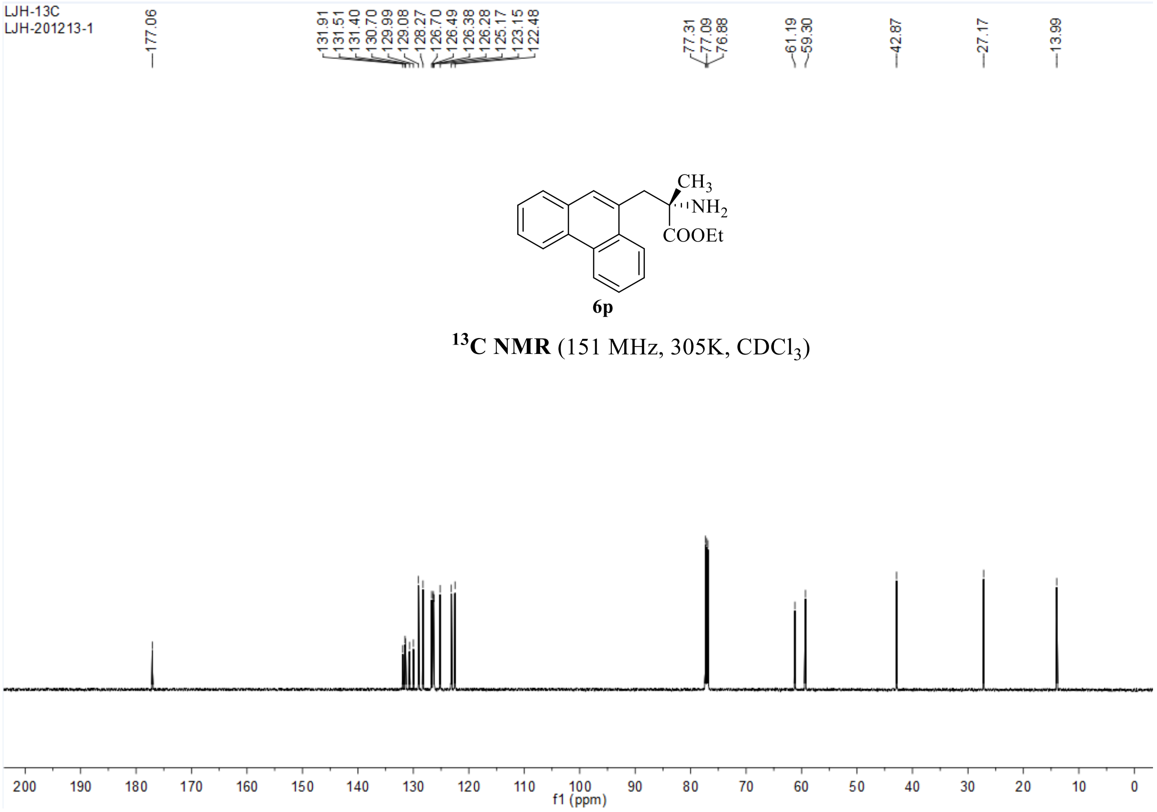

6p

^{13}**C NMR** (151 MHz, 305K, $CDCl_3$)

Supplementary Figure 38: NMR of compound 6p.

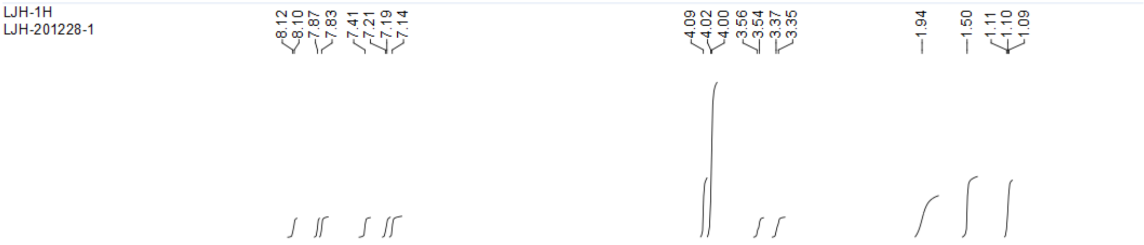

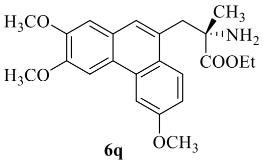

6q

^{1}H NMR (600 MHz, 305K, $CDCl_3$)

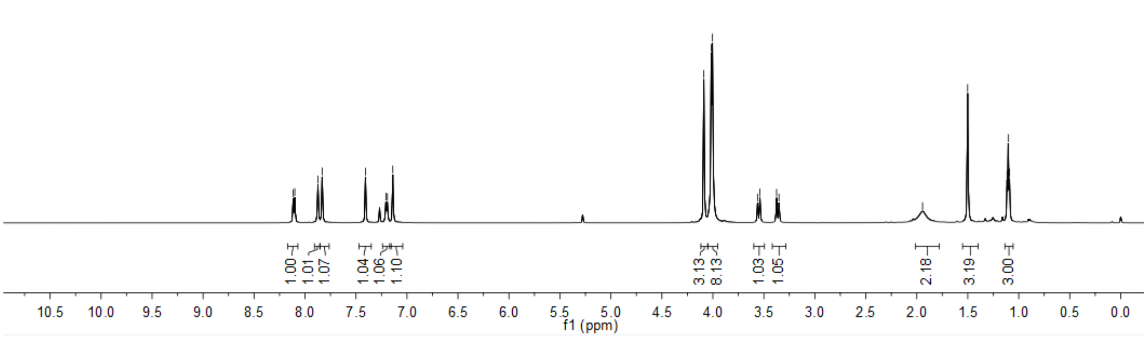

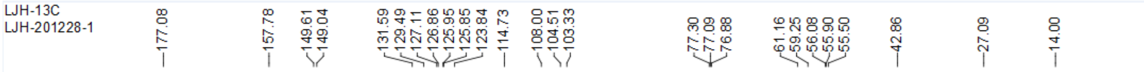

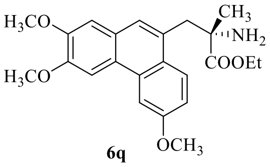

6q

^{13}C NMR (151 MHz, 305K, $CDCl_3$)

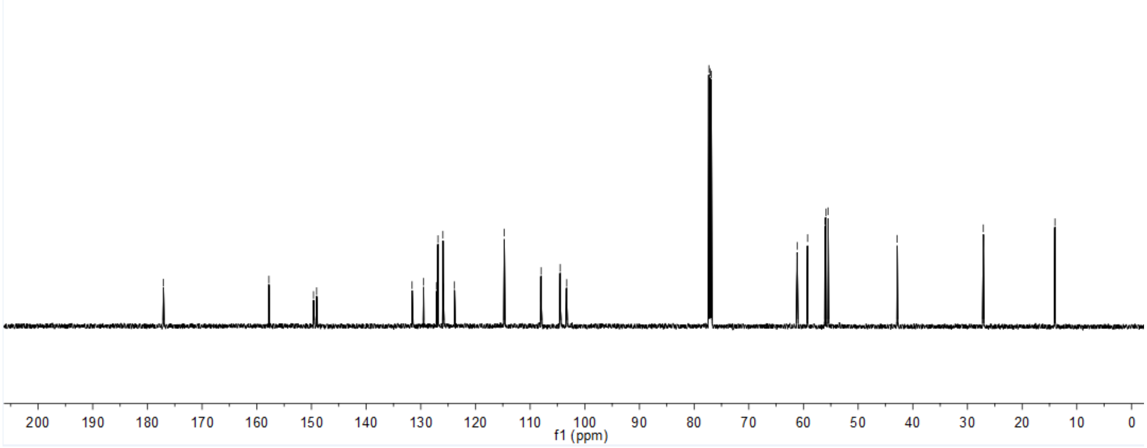

Supplementary Figure 39: NMR of compound 6q.

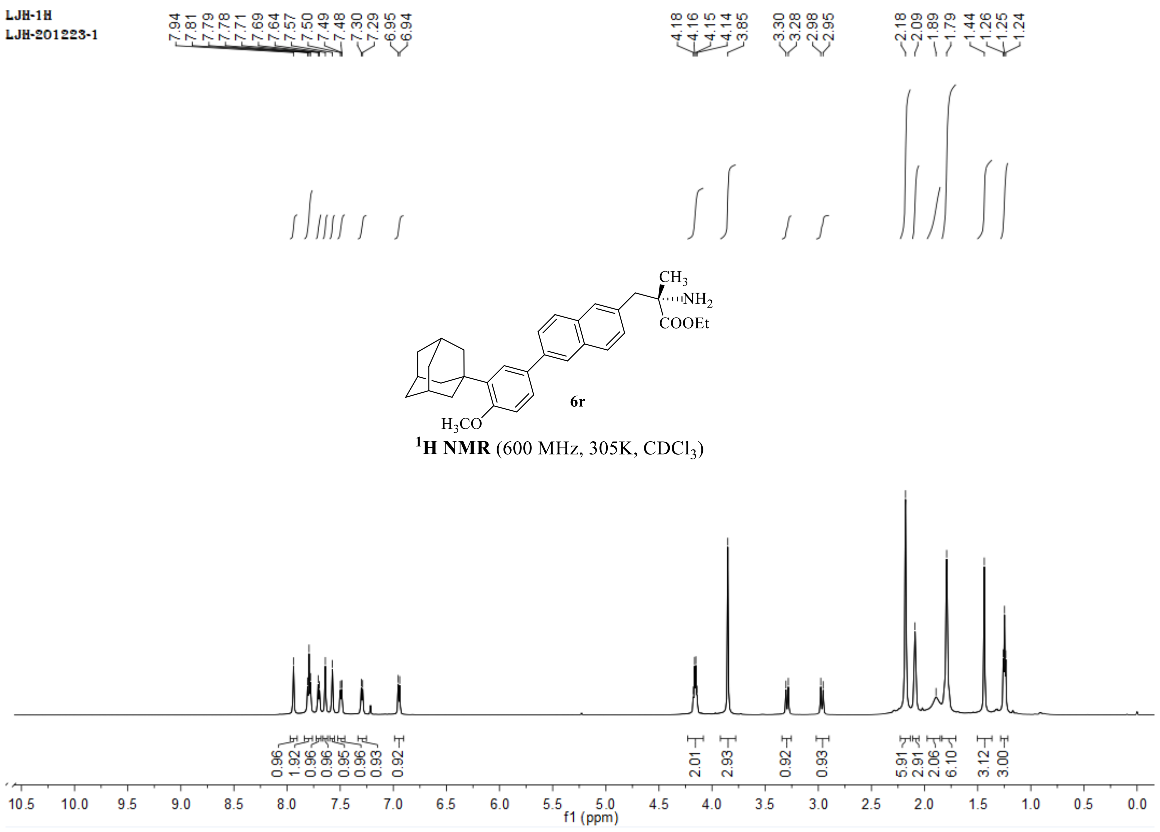

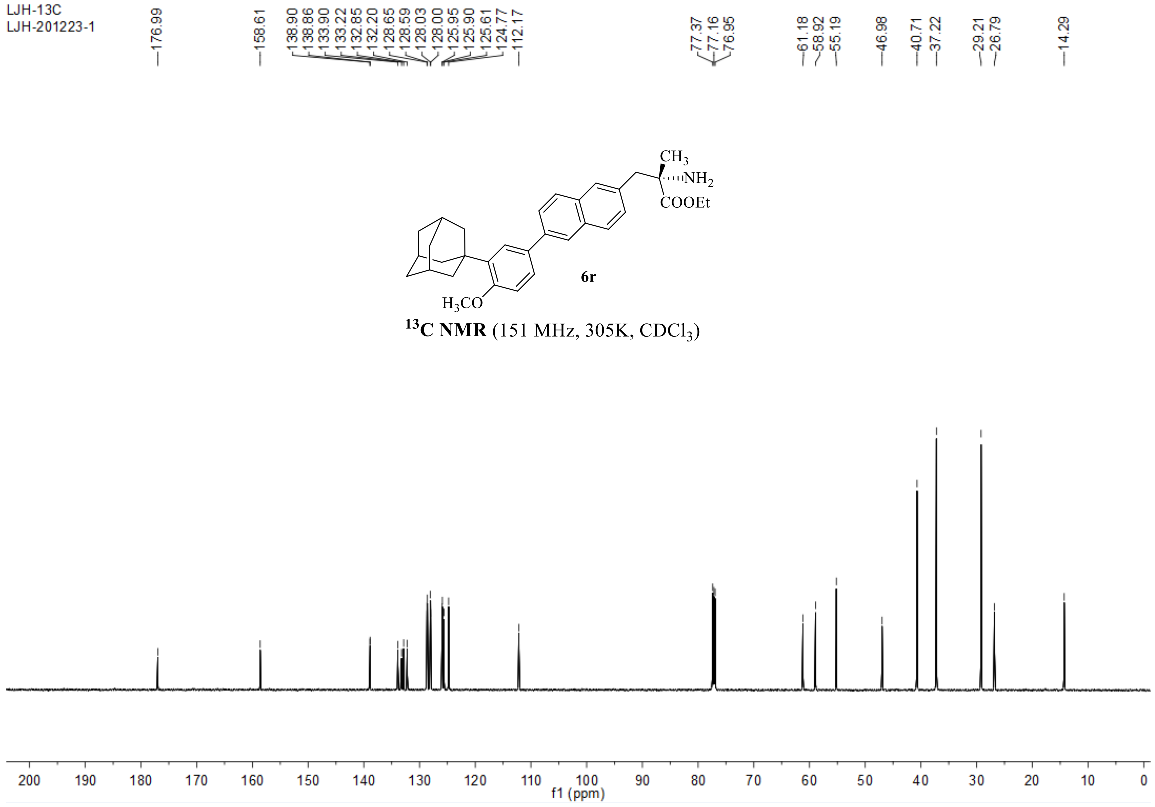

Supplementary Figure 40: NMR of compound 6r.

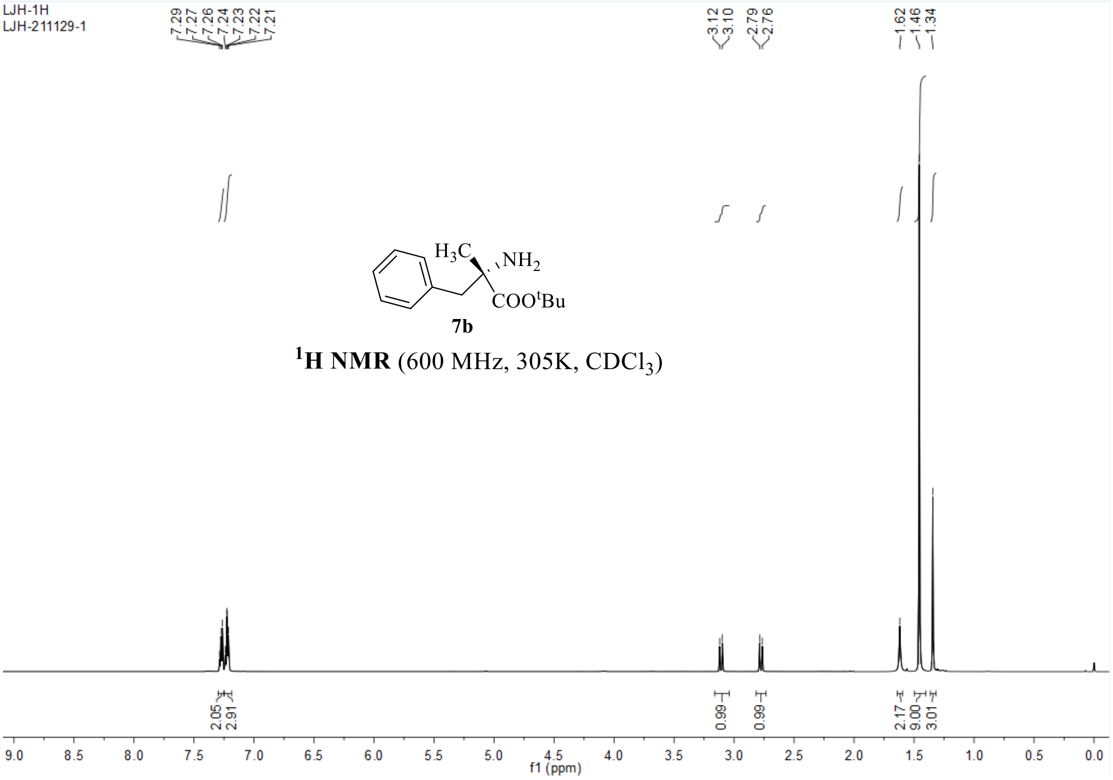

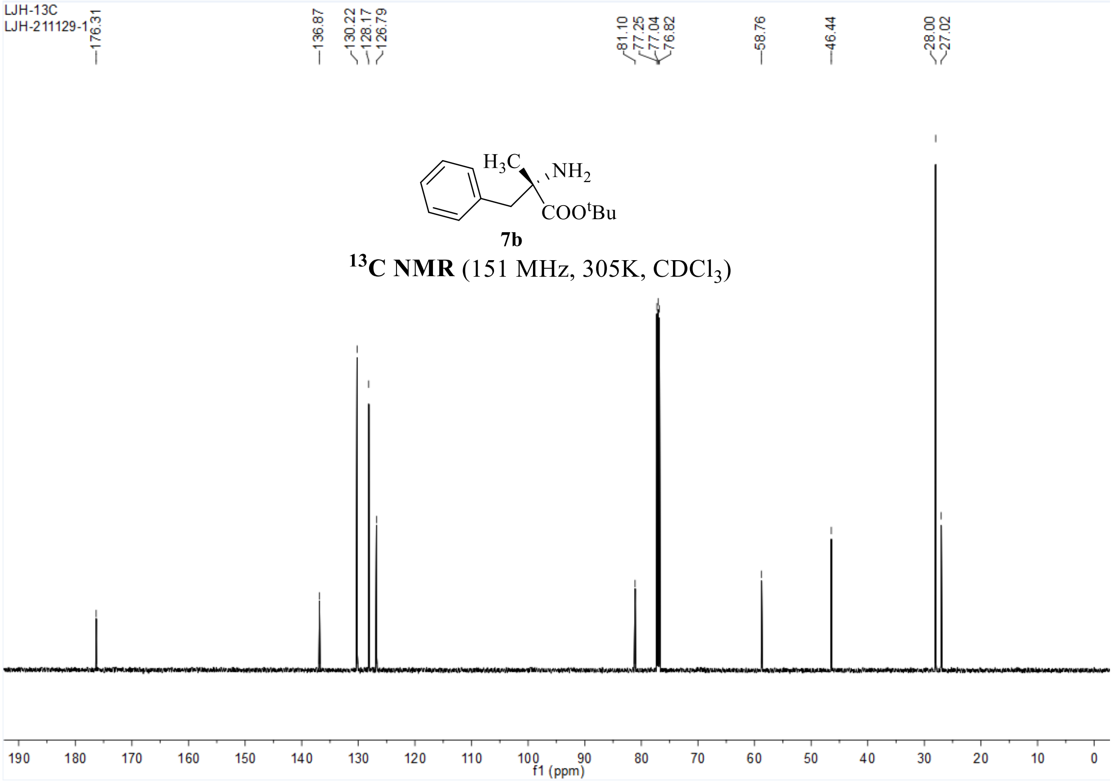

Supplementary Figure 41: NMR of compound 7b.

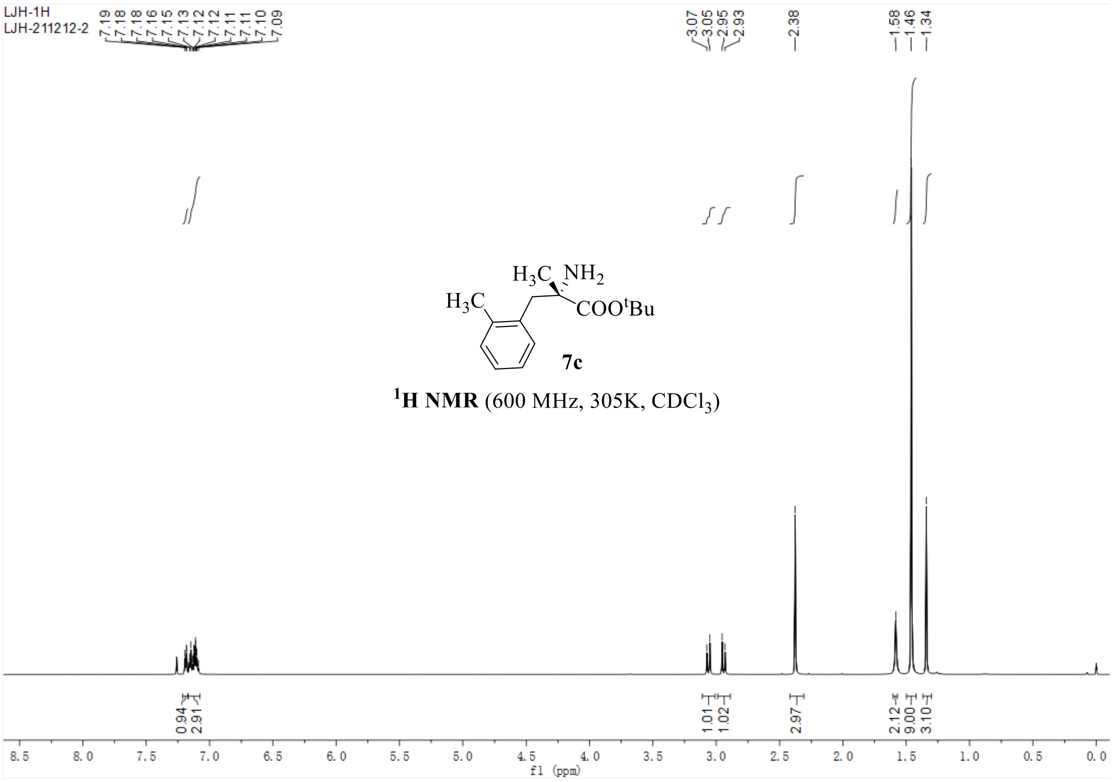

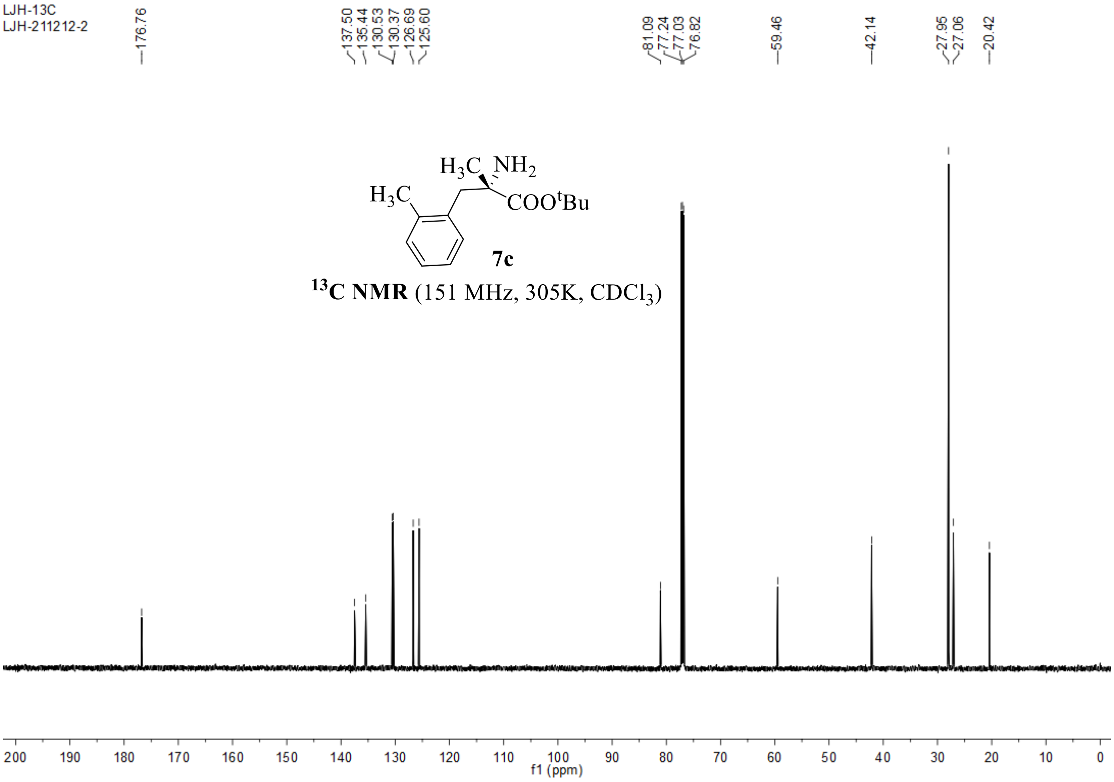

Supplementary Figure 42: NMR of compound 7c.

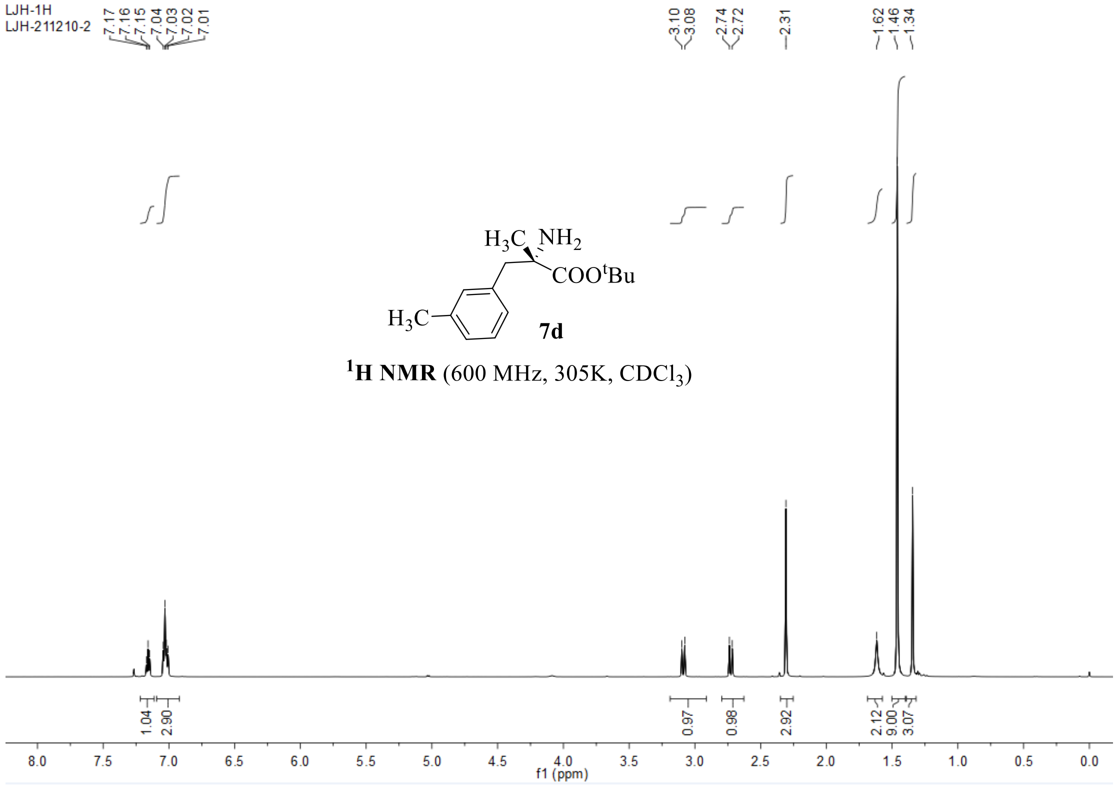

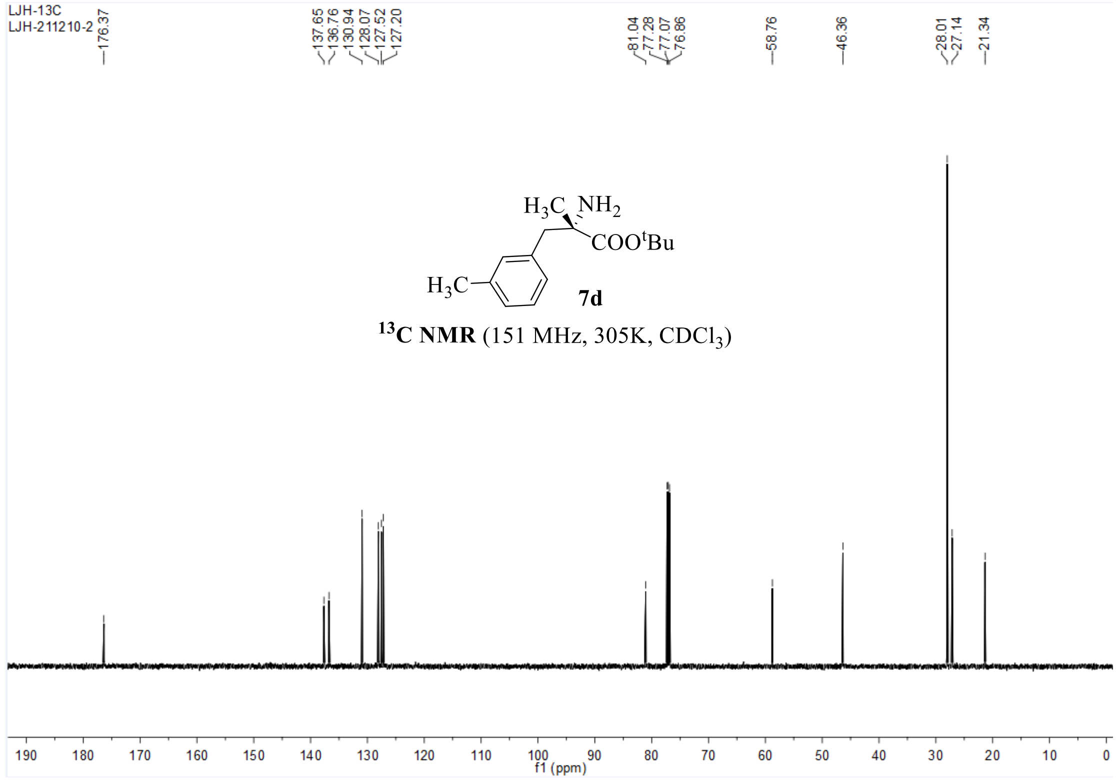

Supplementary Figure 43: NMR of compound 7d.

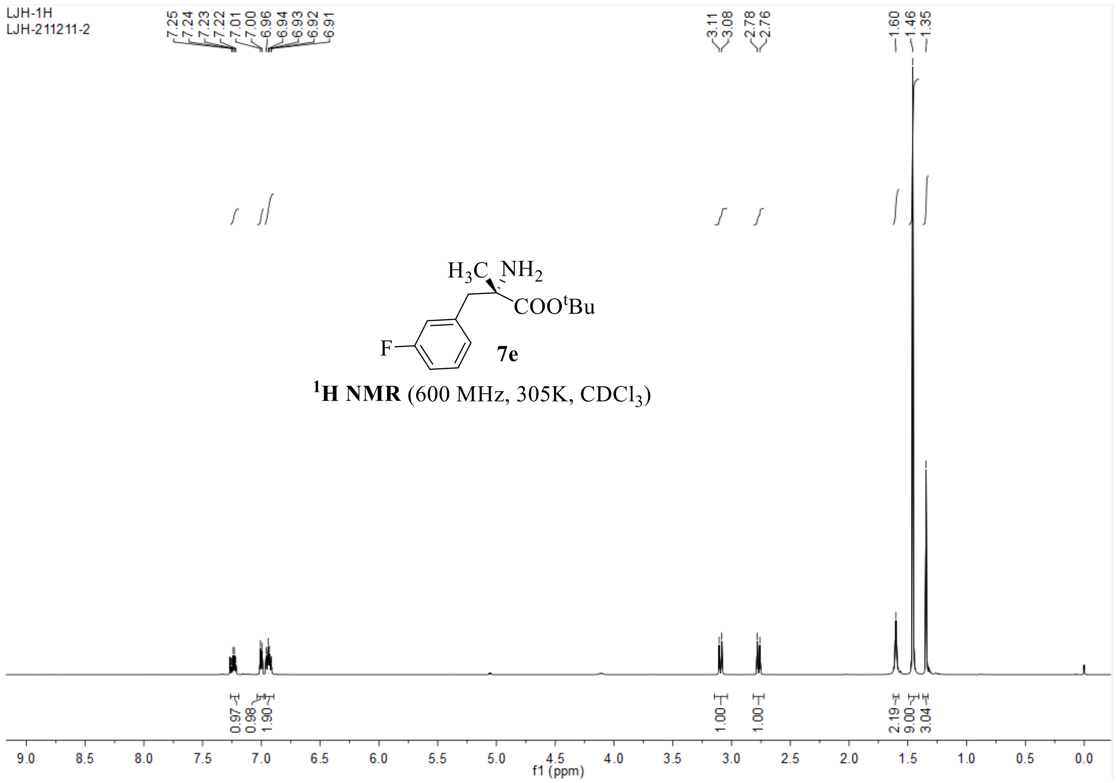

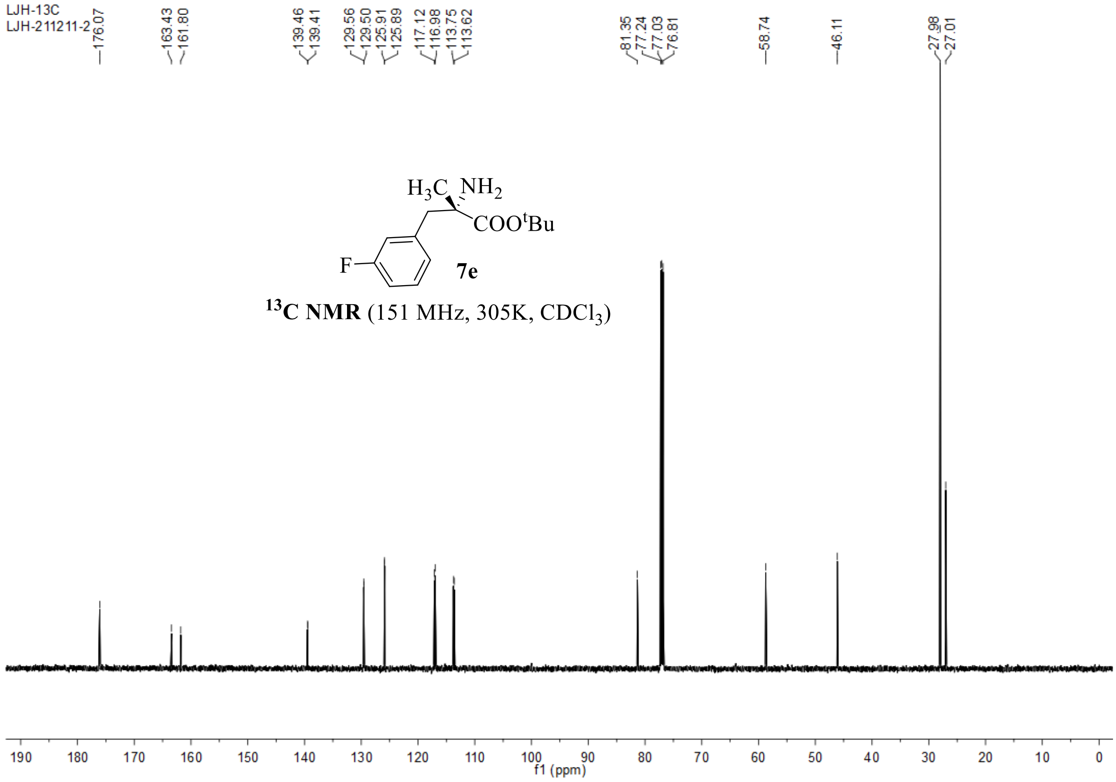

Supplementary Figure 44: NMR of compound 7e.

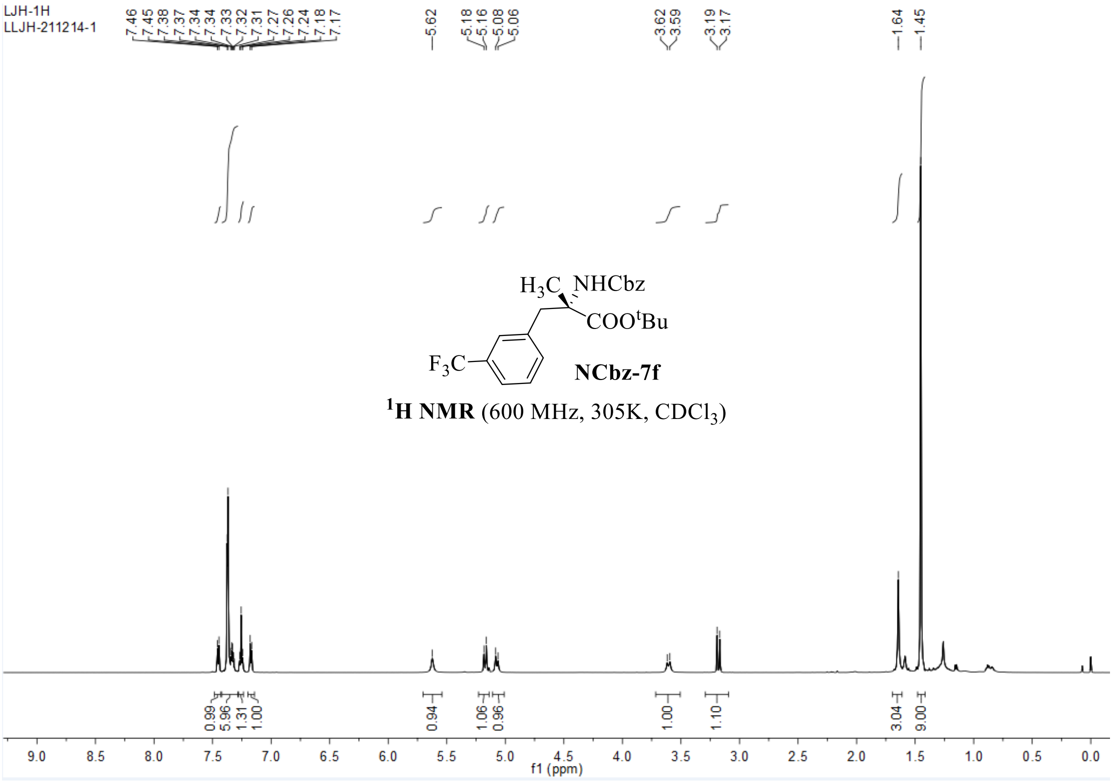

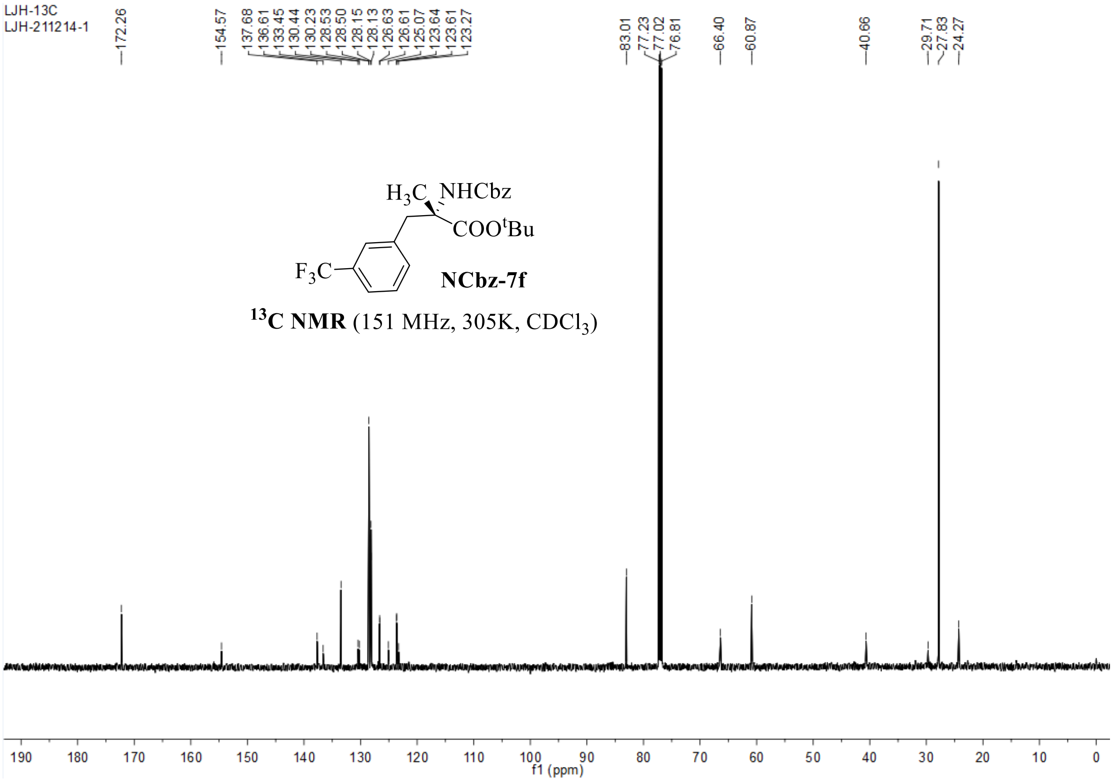

Supplementary Figure 45: NMR of compound NCbz-7f.

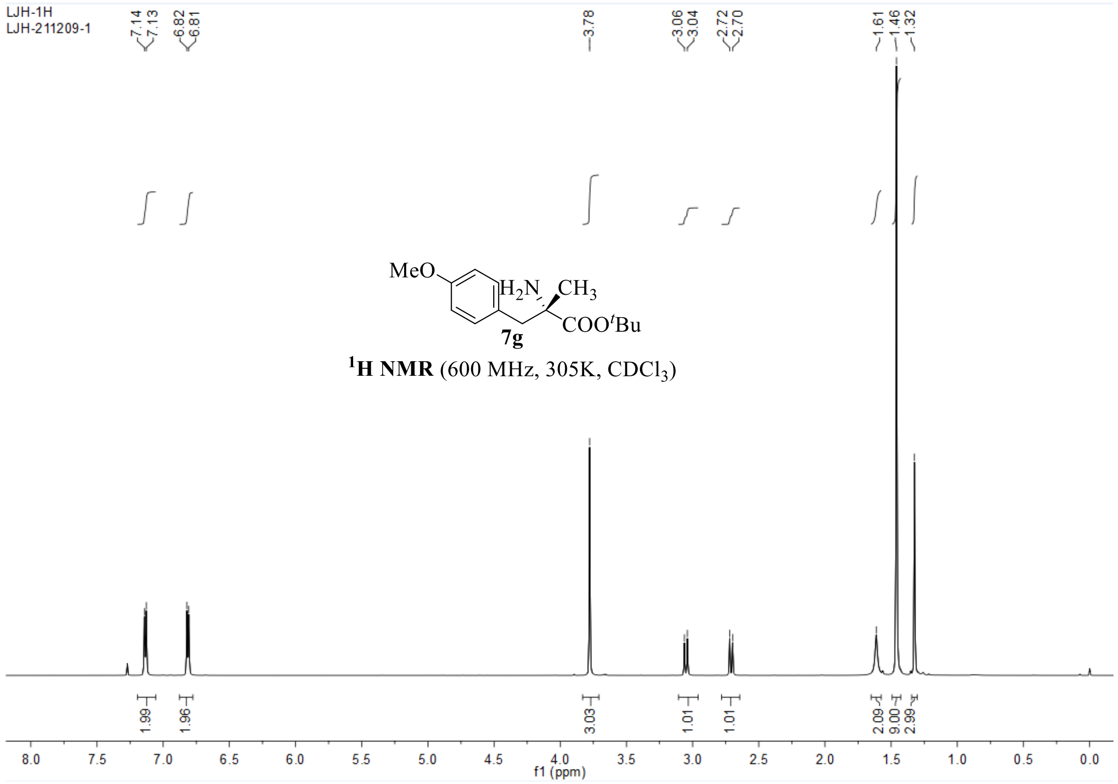

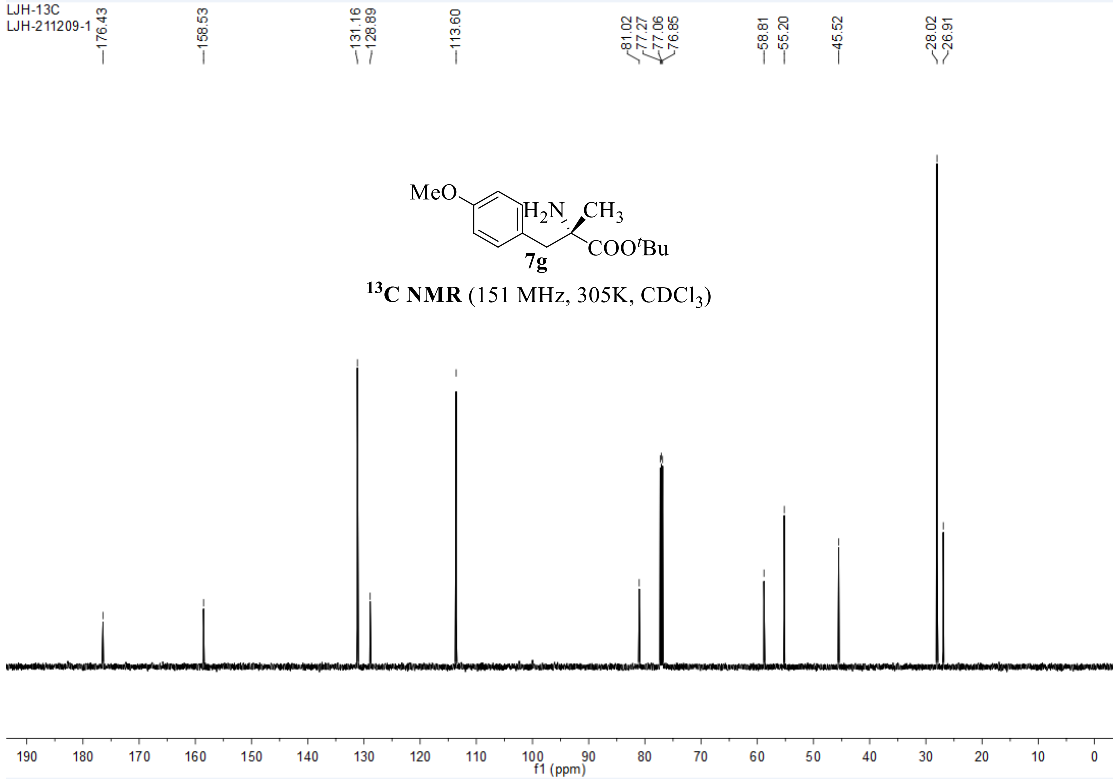

Supplementary Figure 46: NMR of compound 7g.

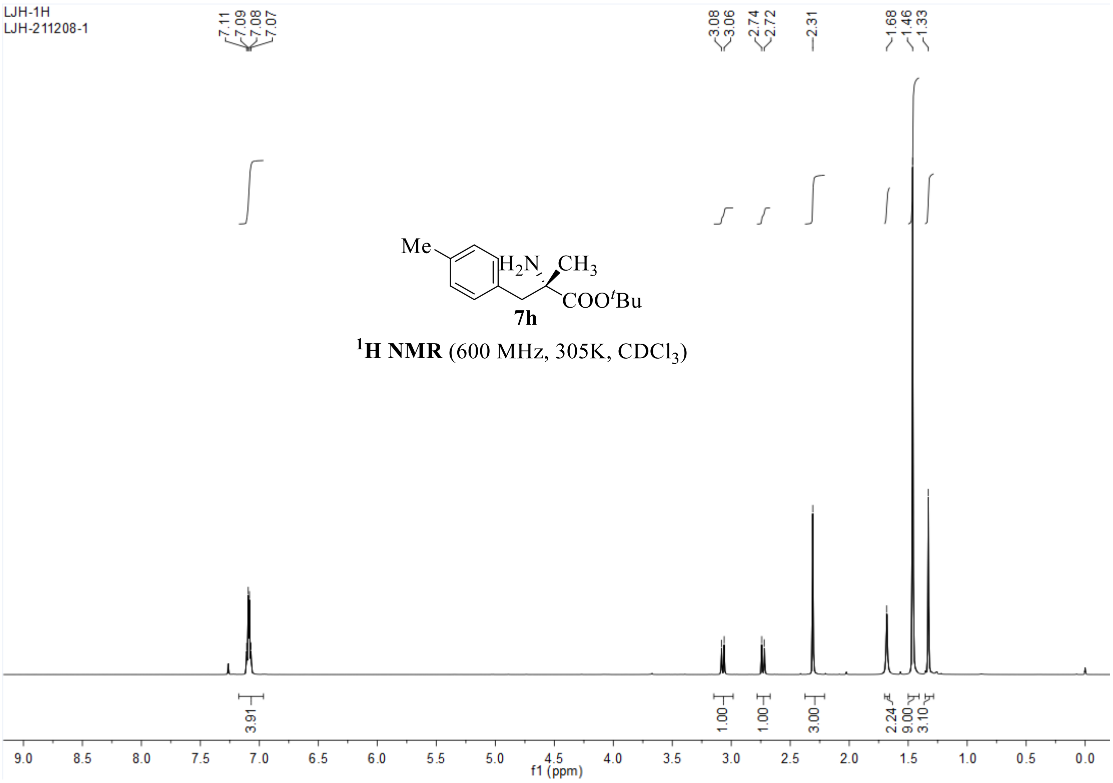

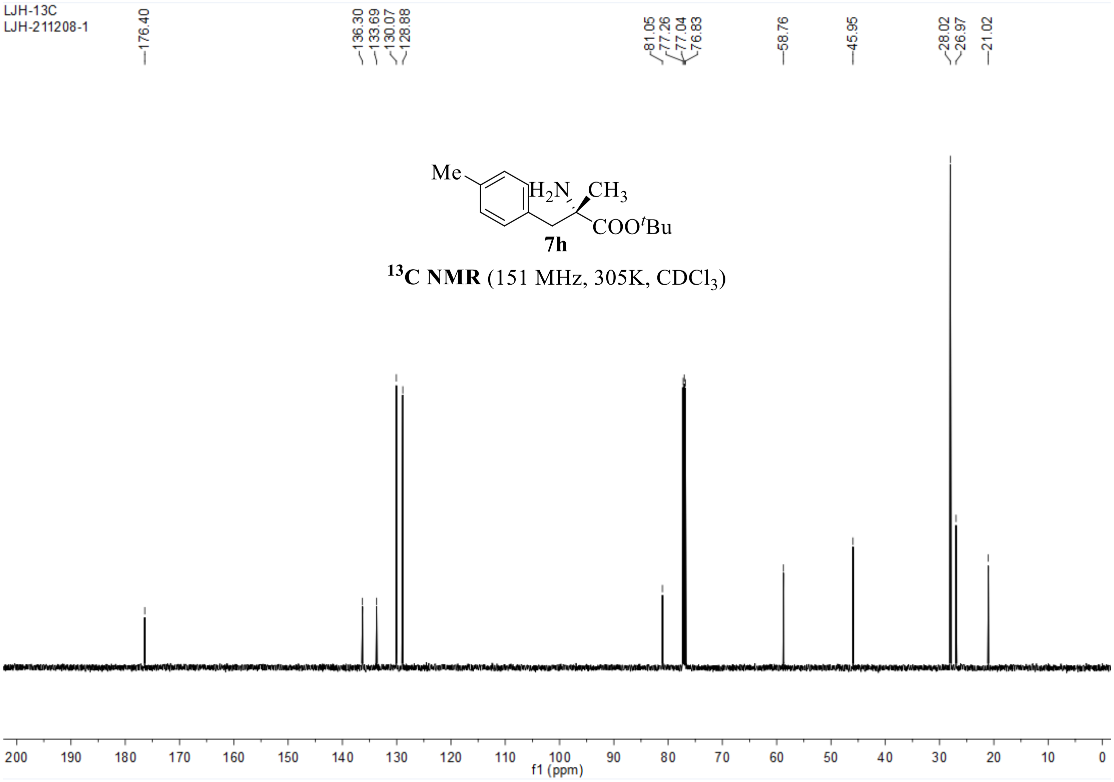

Supplementary Figure 47: NMR of compound 7h.

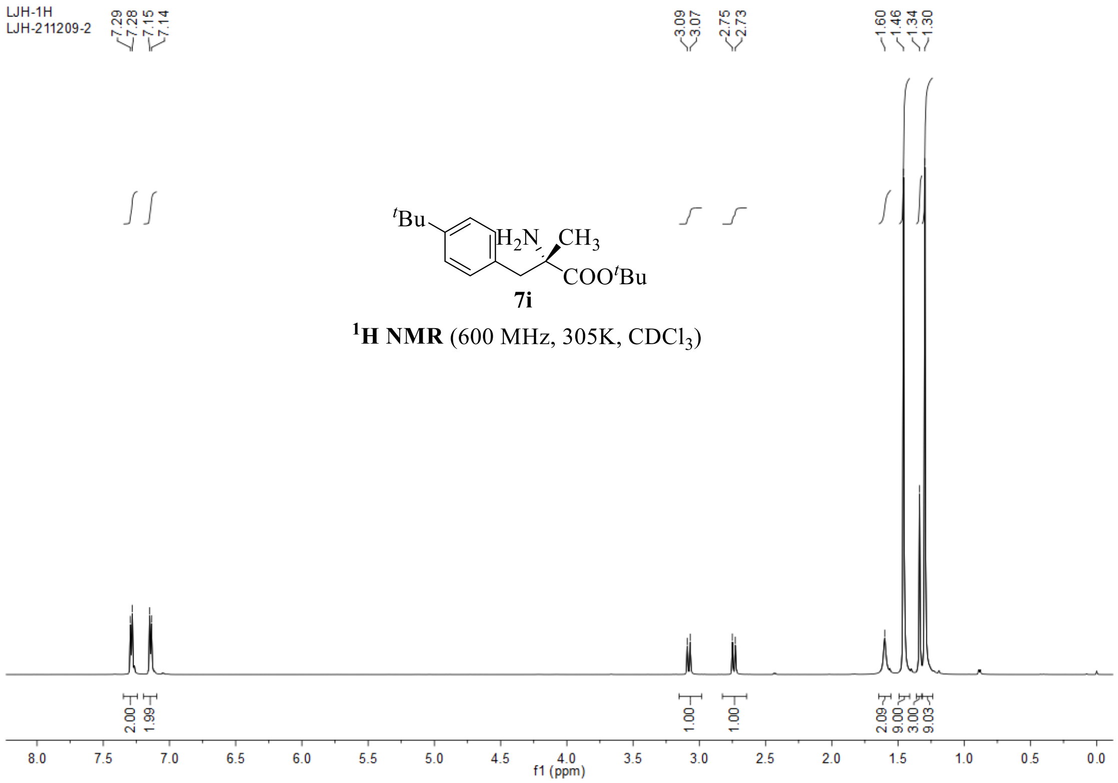

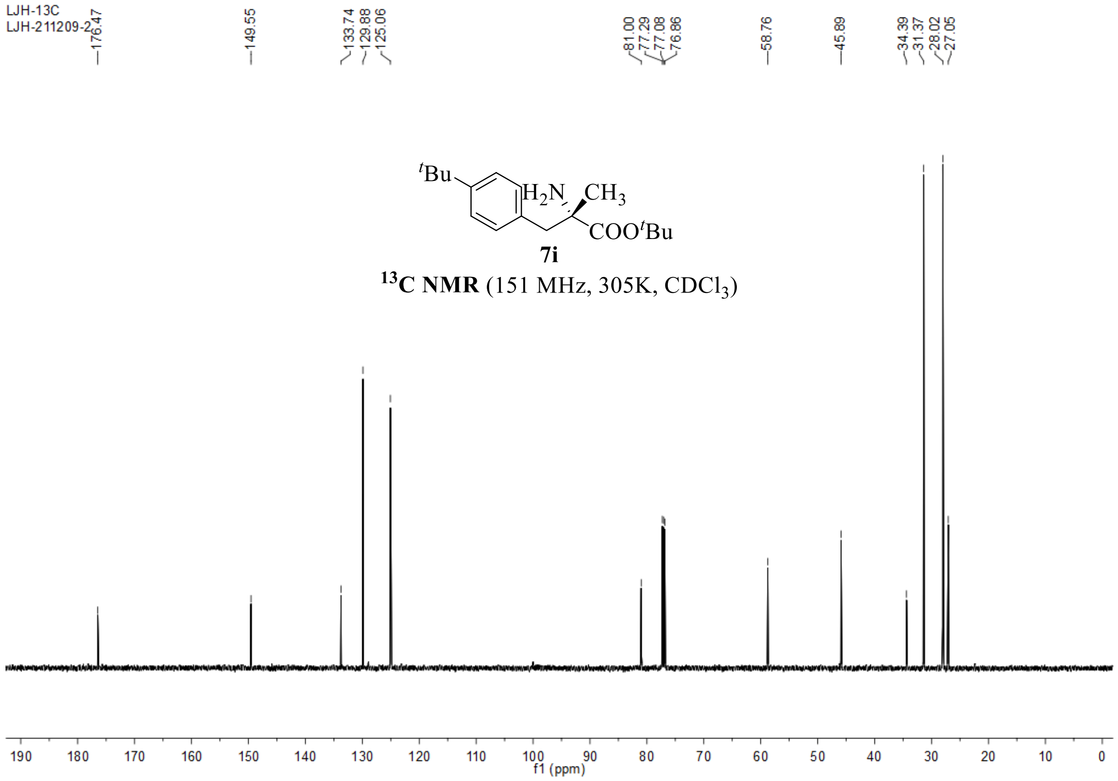

Supplementary Figure 48: NMR of compound 7i.

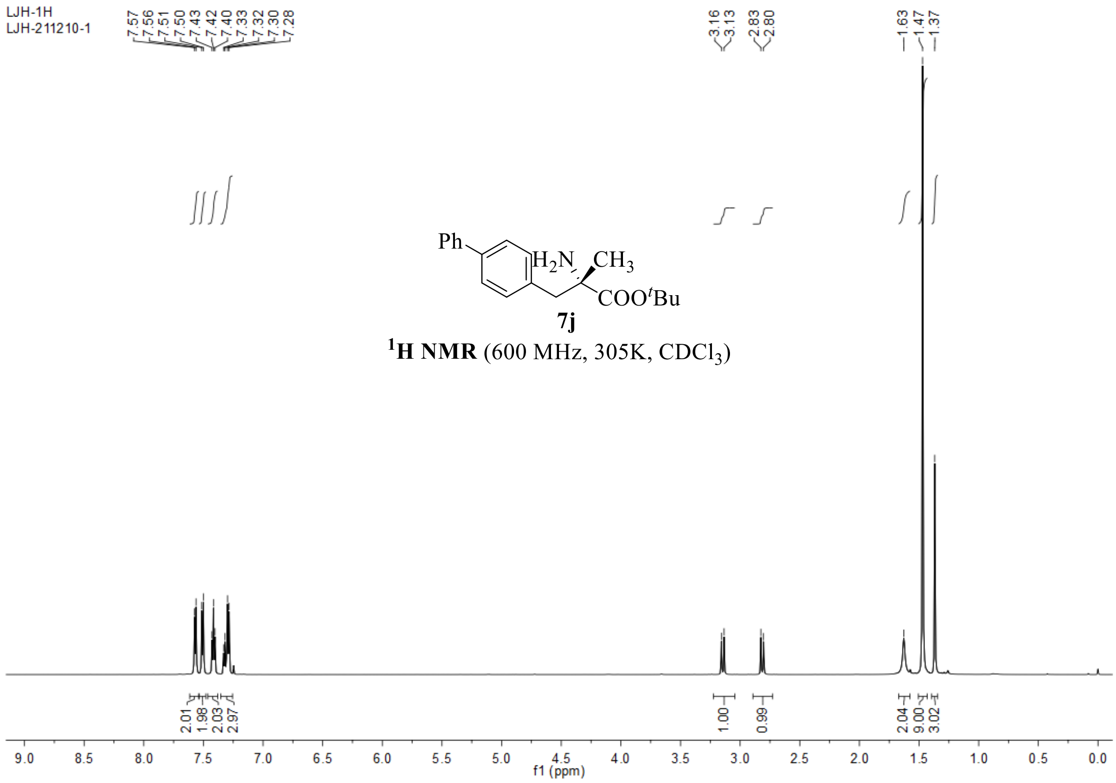

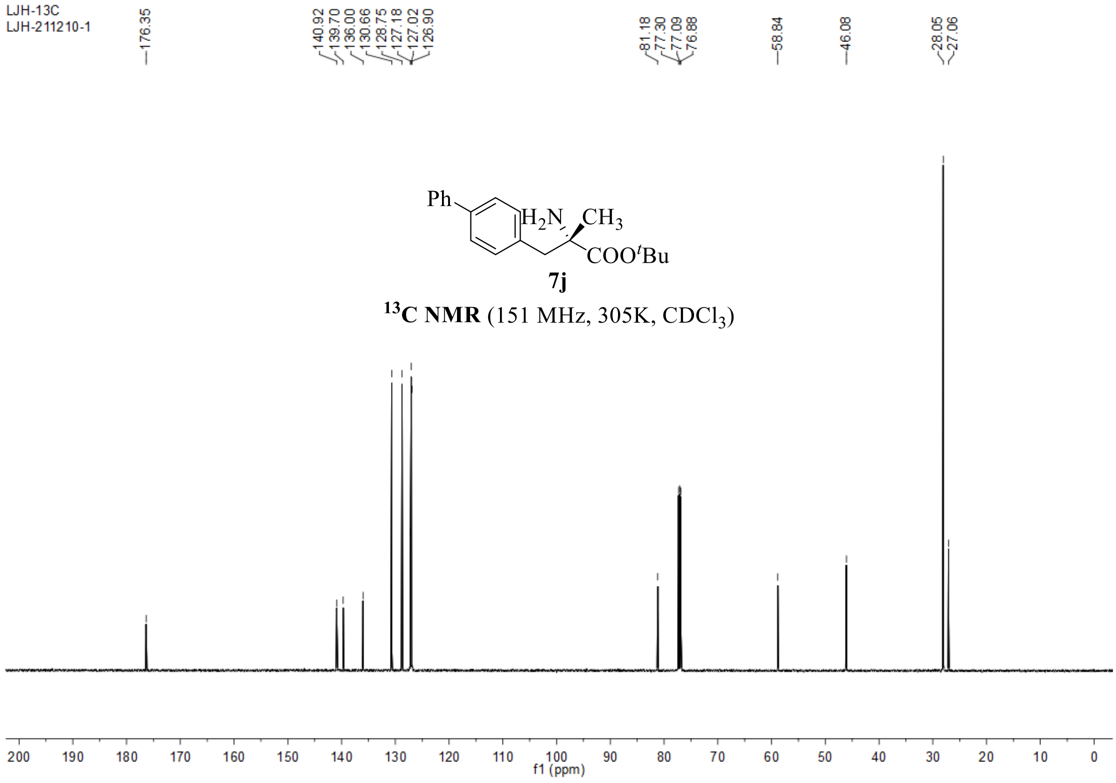

Supplementary Figure 49: NMR of compound 7j.

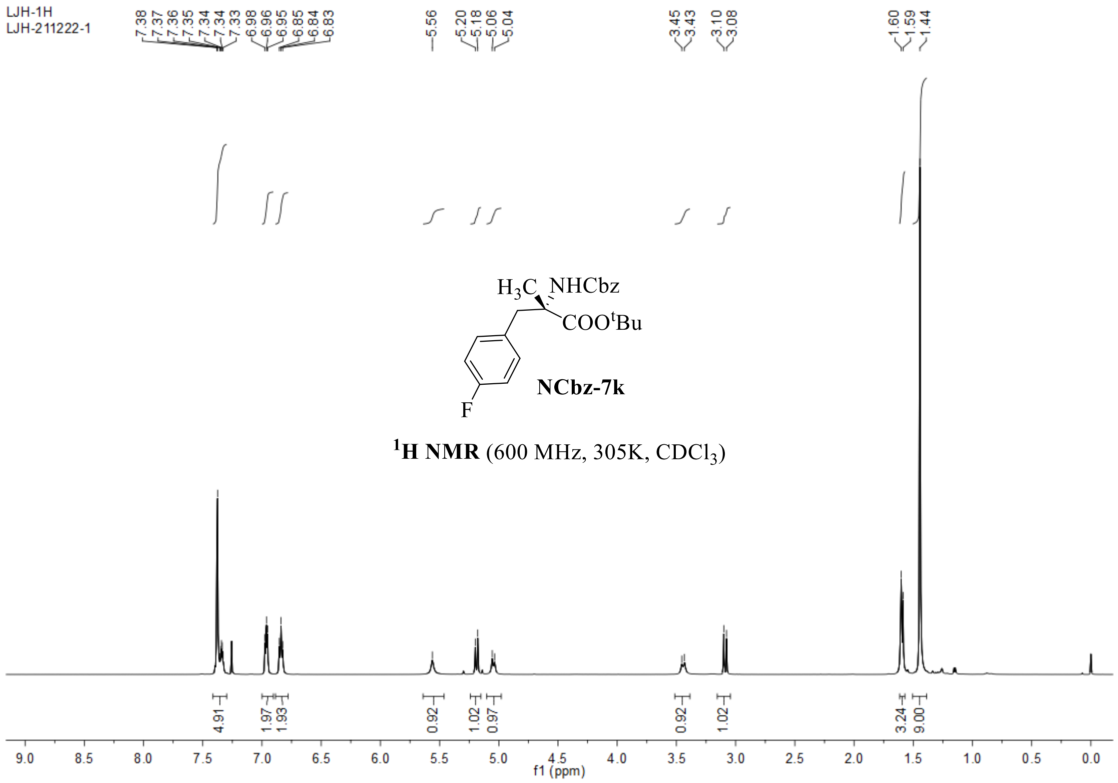

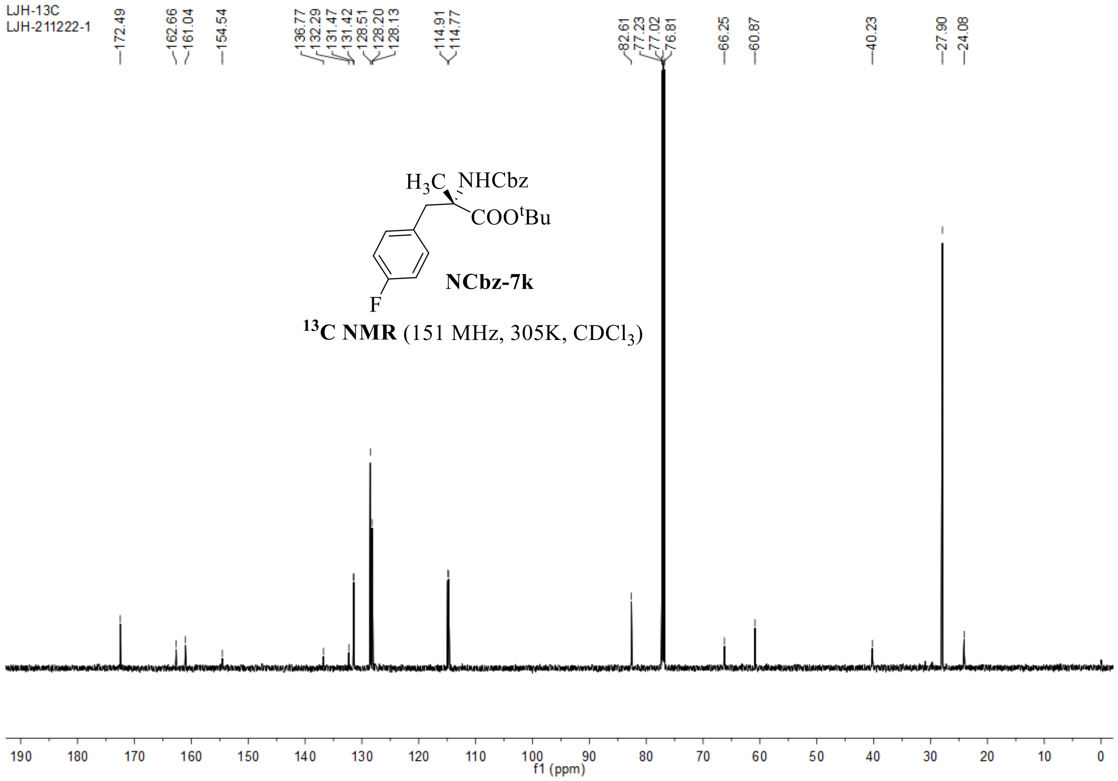

Supplementary Figure 50: NMR of compound NCbz-7k.

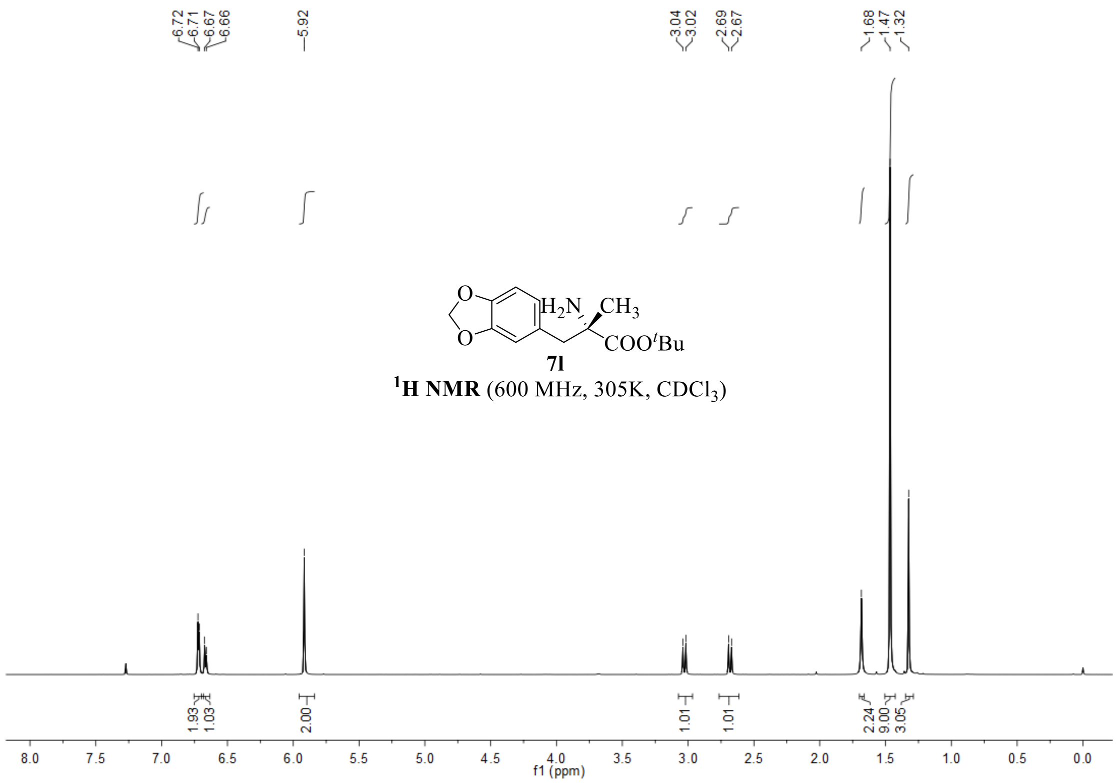

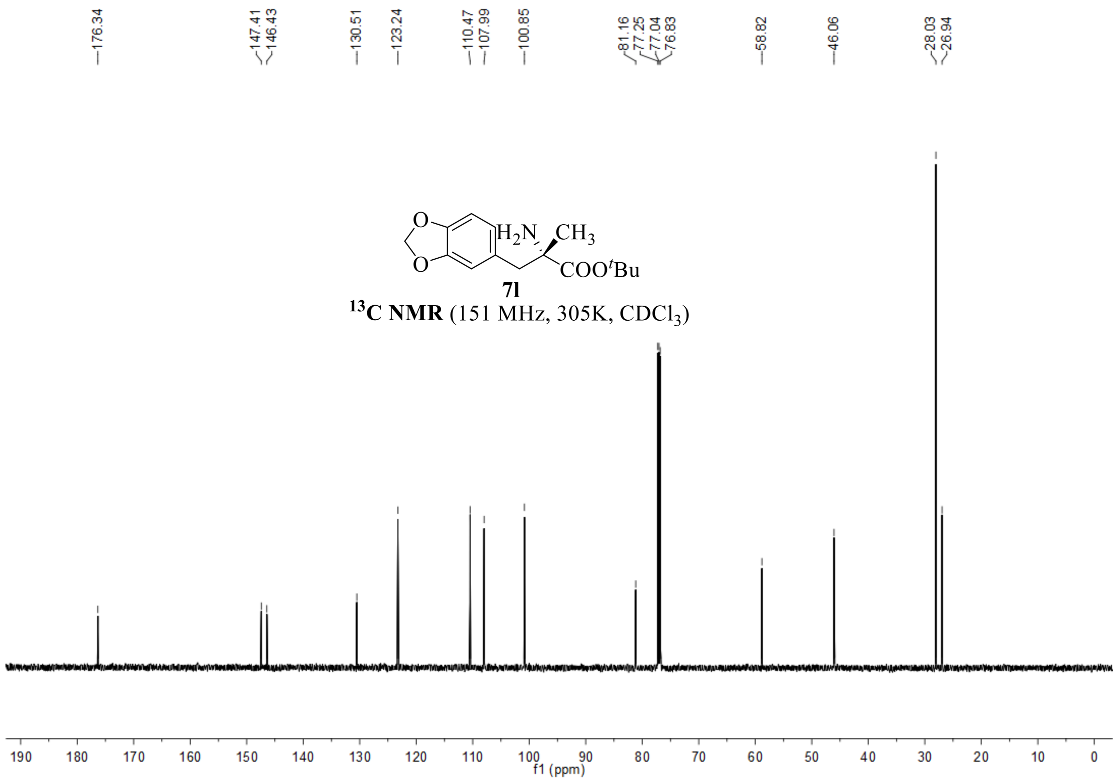

Supplementary Figure 51: NMR of compound 7l.

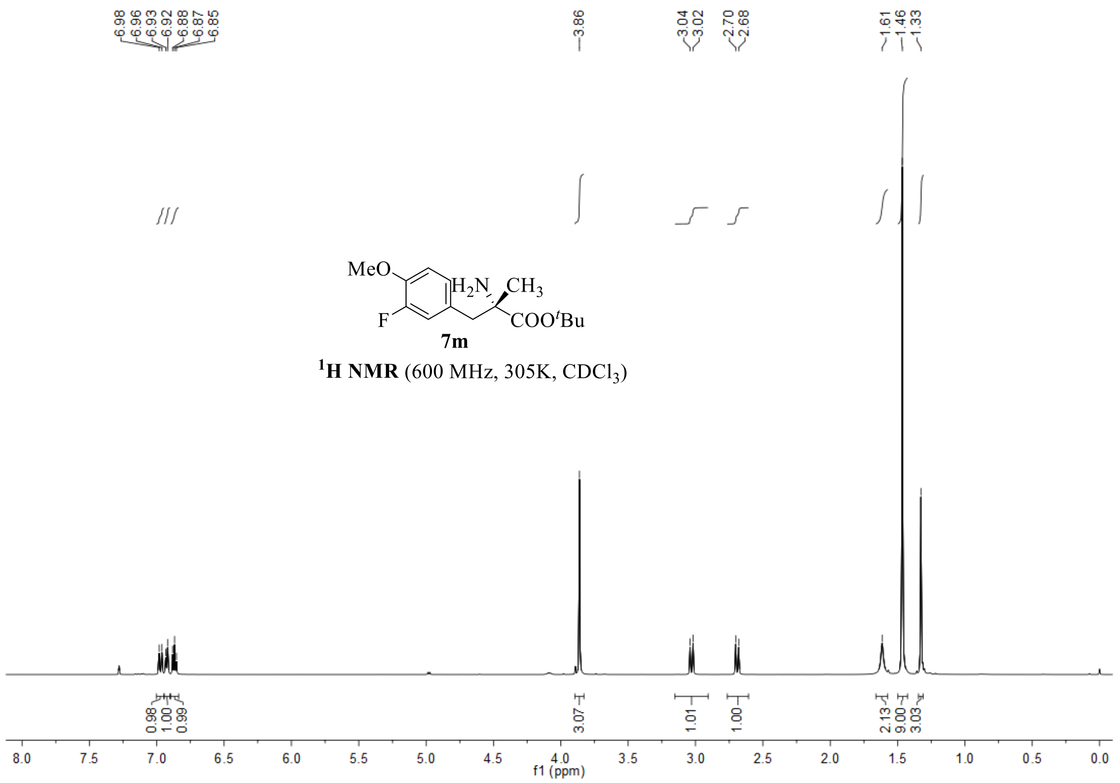

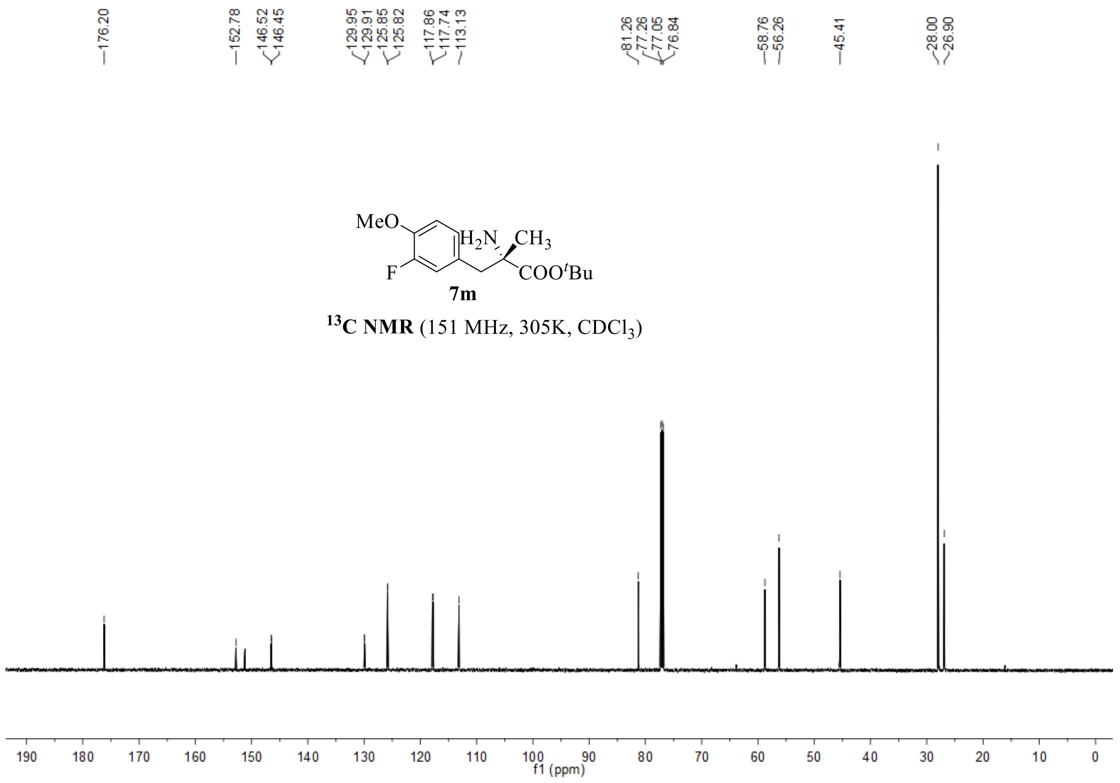

Supplementary Figure 52: NMR of compound 7m.

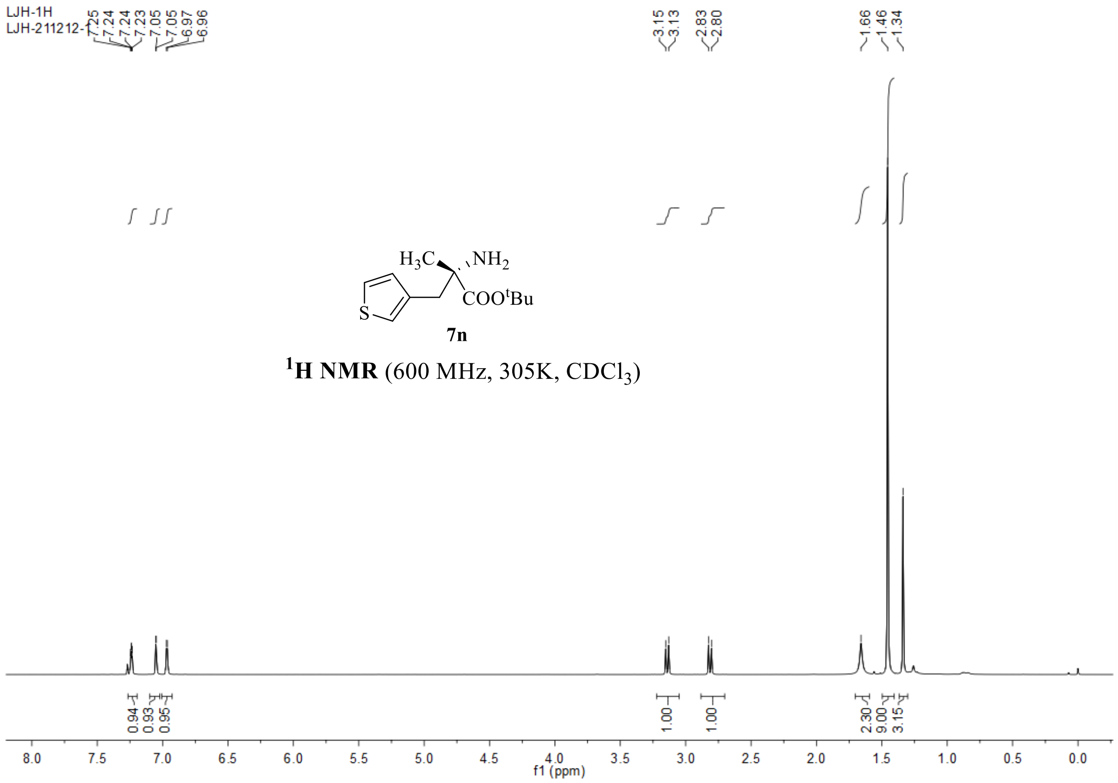

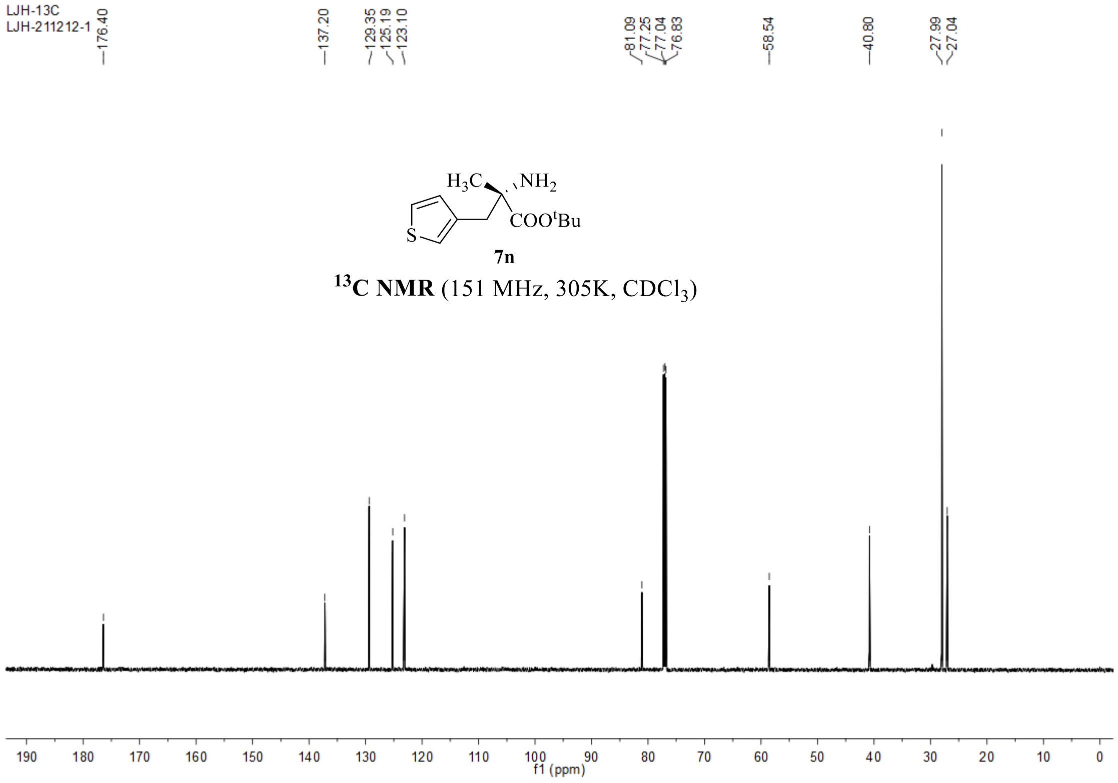

Supplementary Figure 53: NMR of compound 7n.

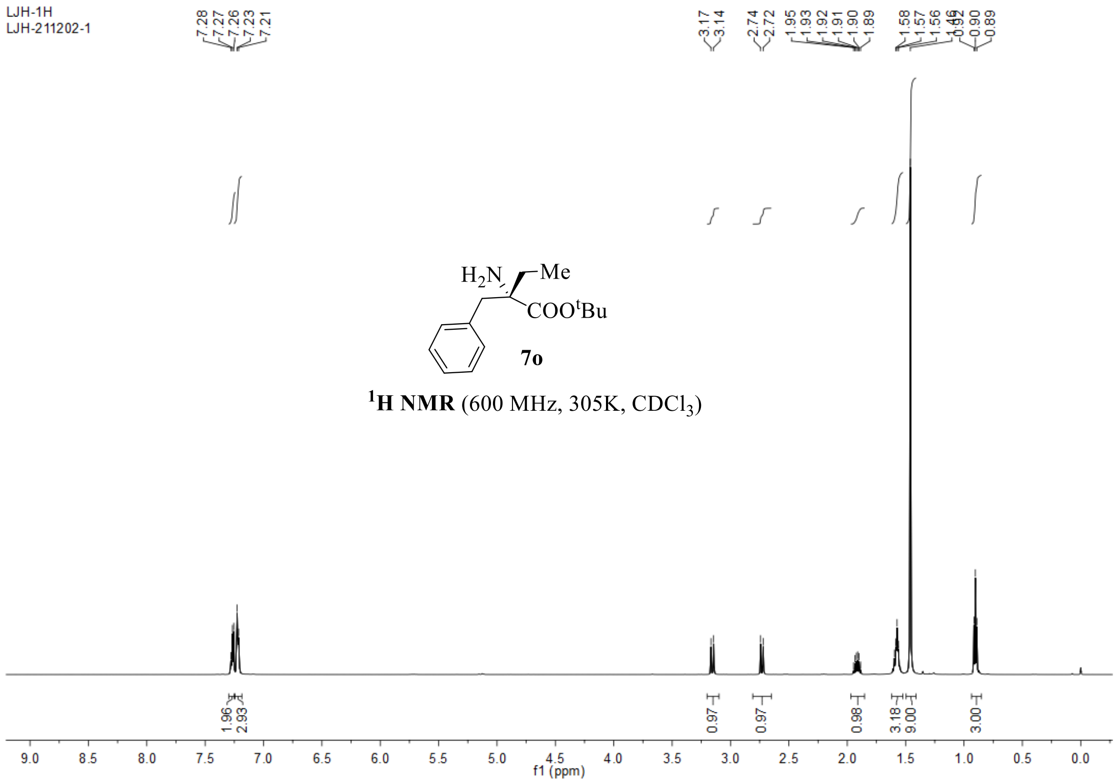

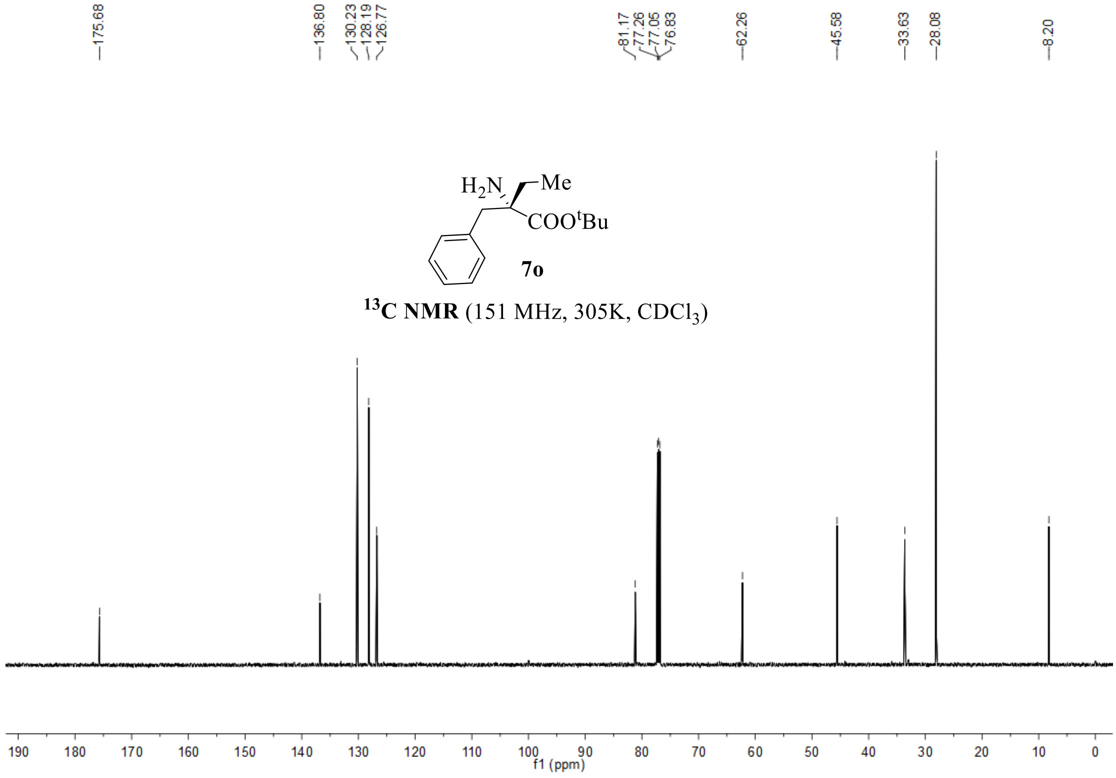

Supplementary Figure 54: NMR of compound 7o.

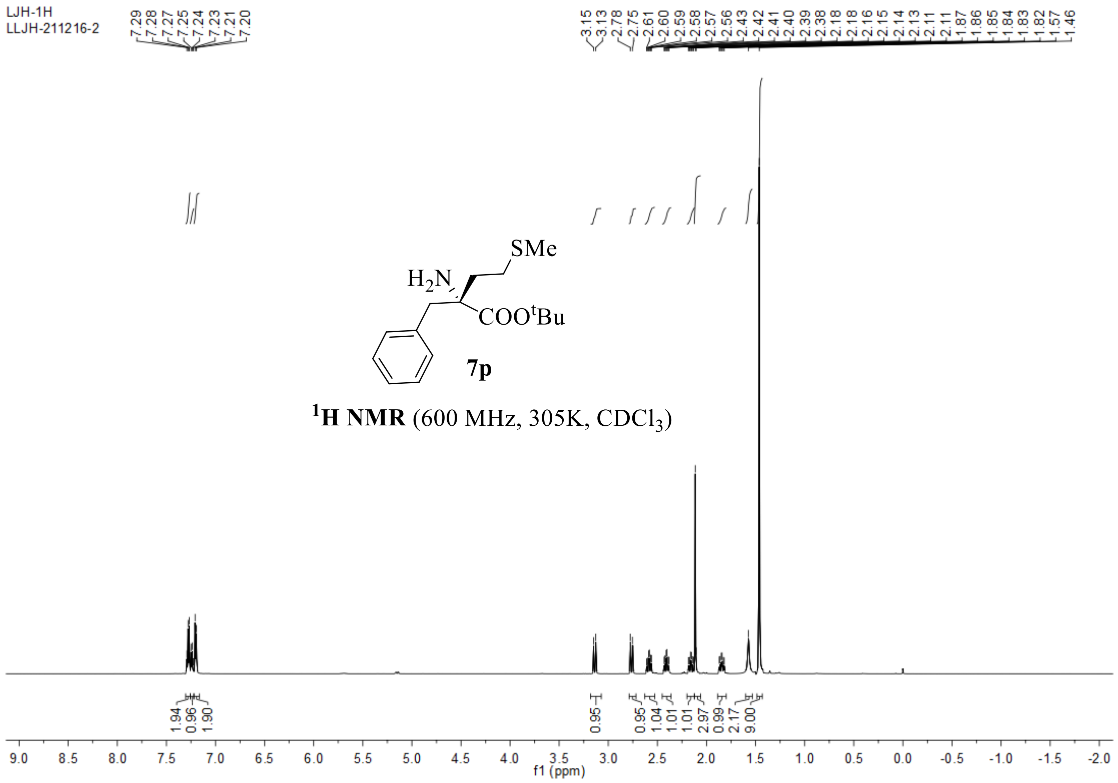

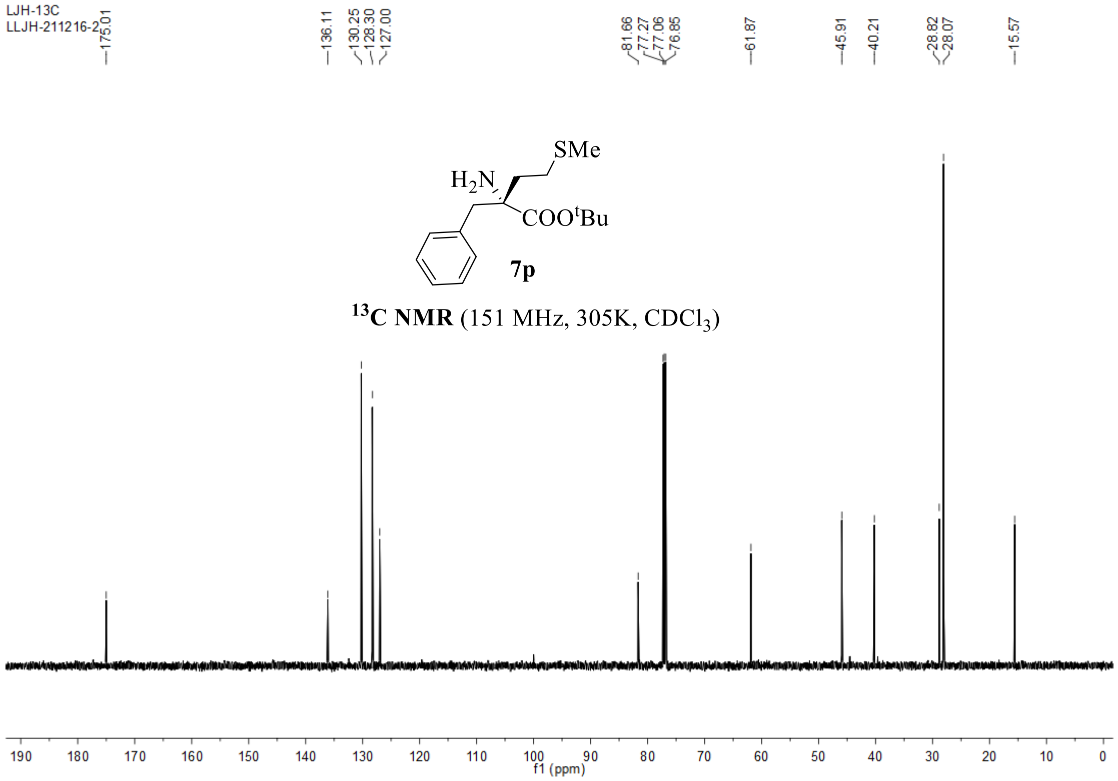

Supplementary Figure 55: NMR of compound 7p.

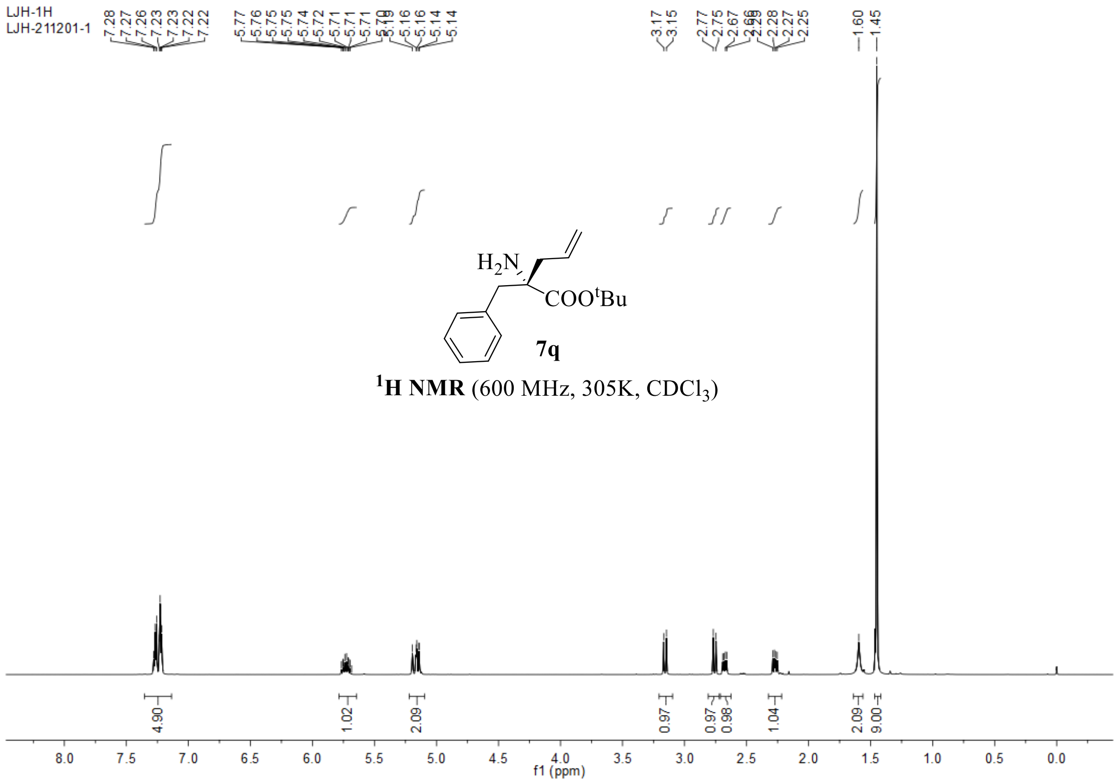

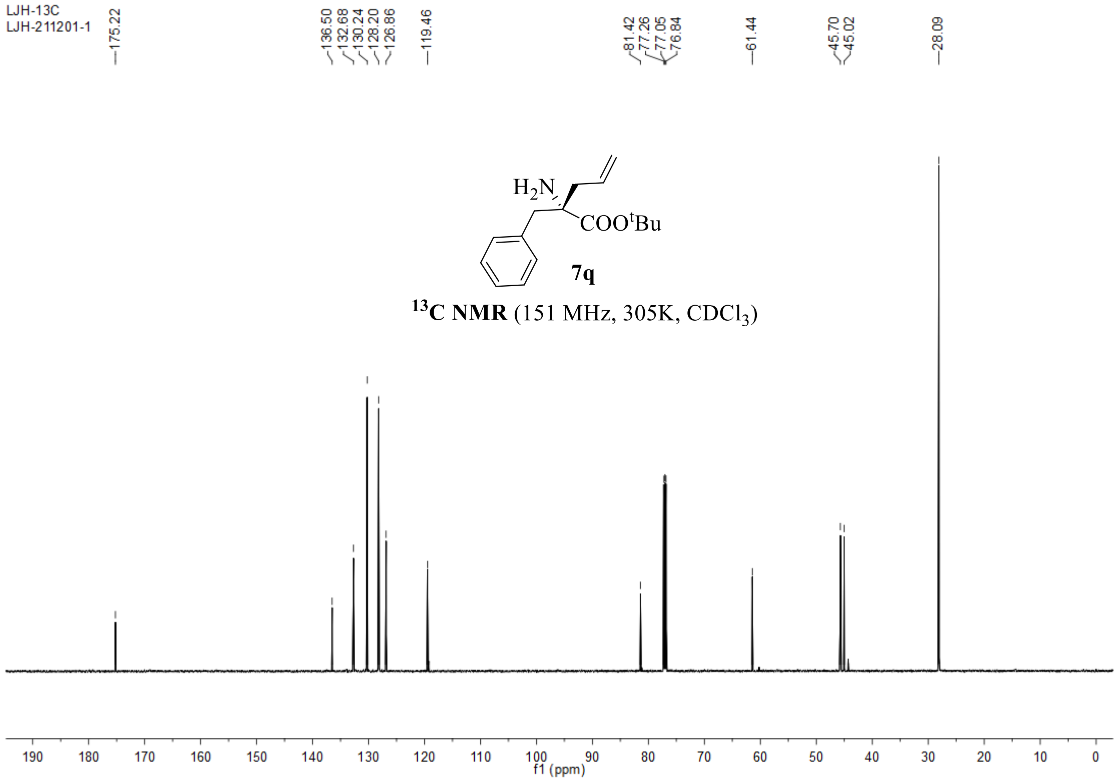

Supplementary Figure 56: NMR of compound 7q.

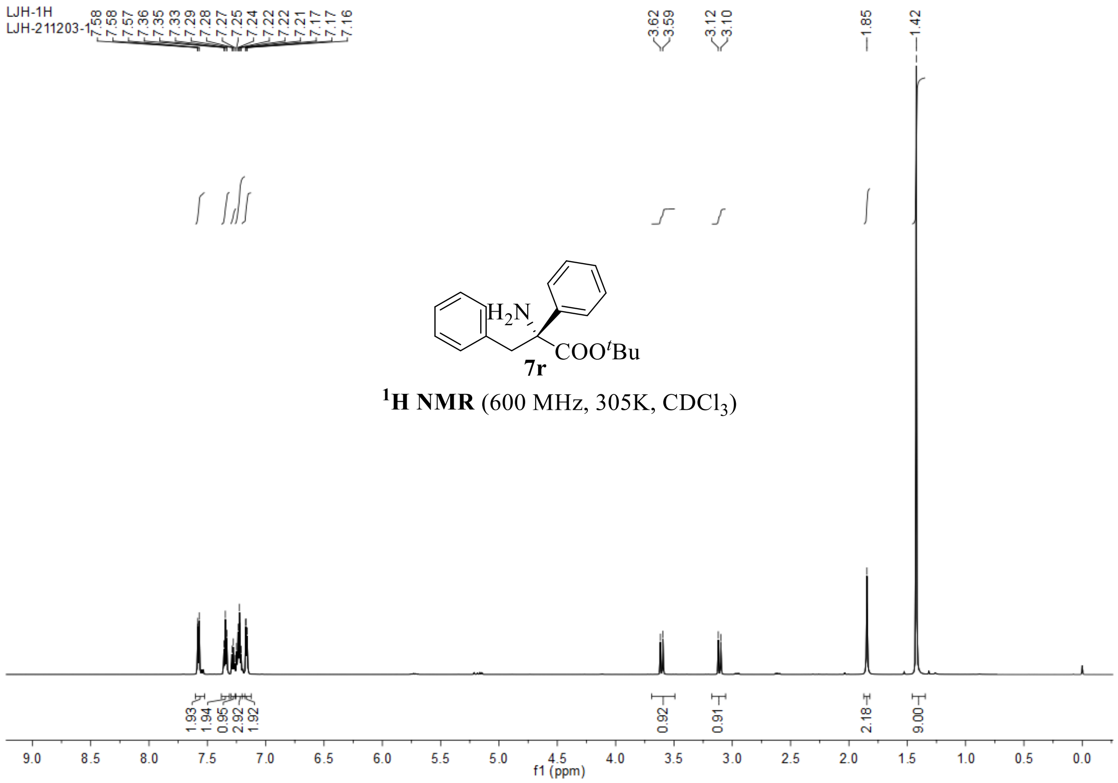

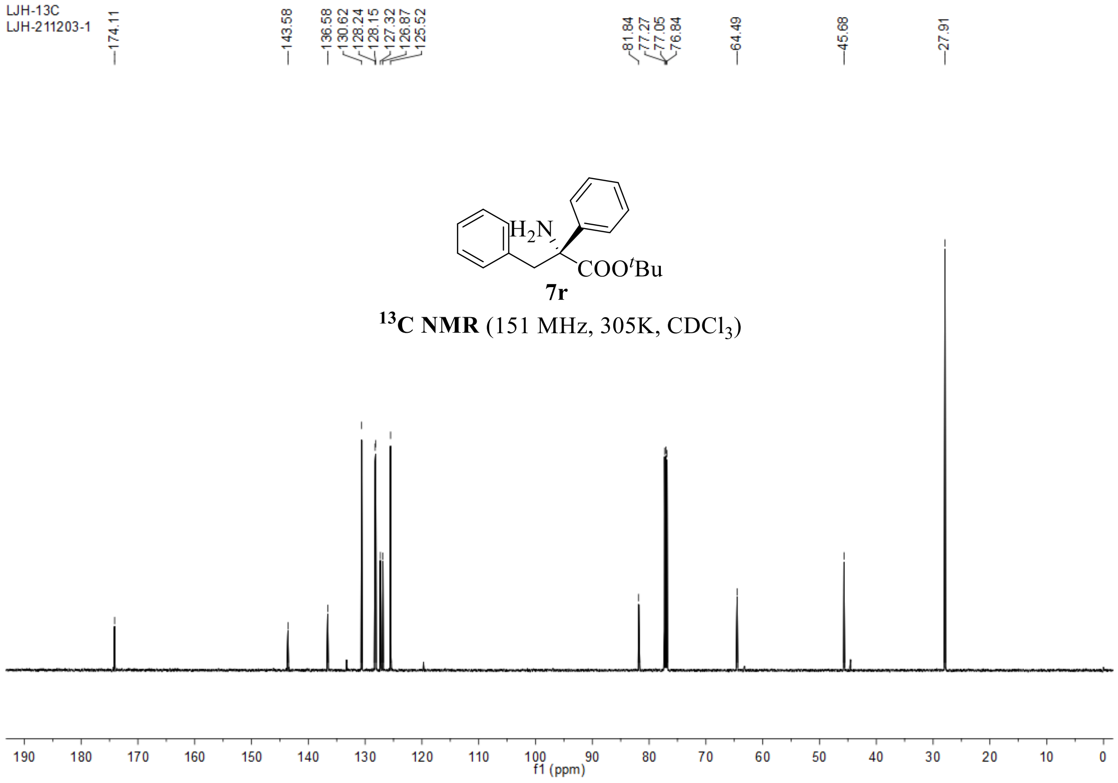

Supplementary Figure 57: NMR of compound 7r.

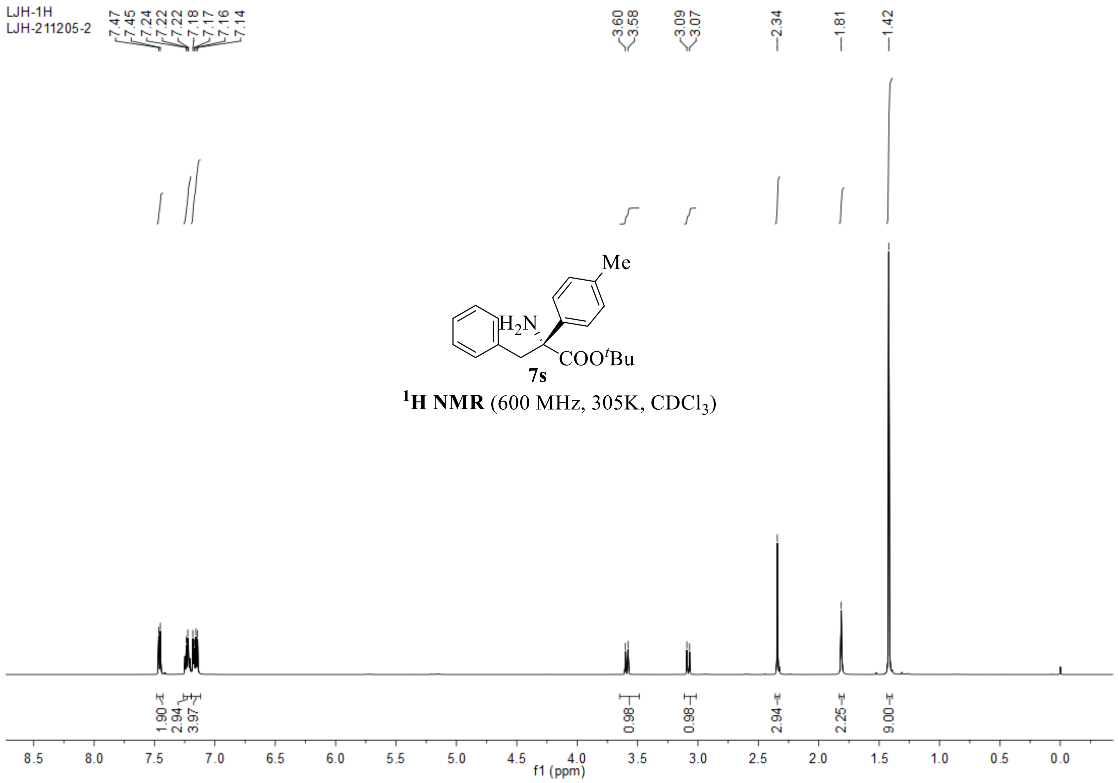

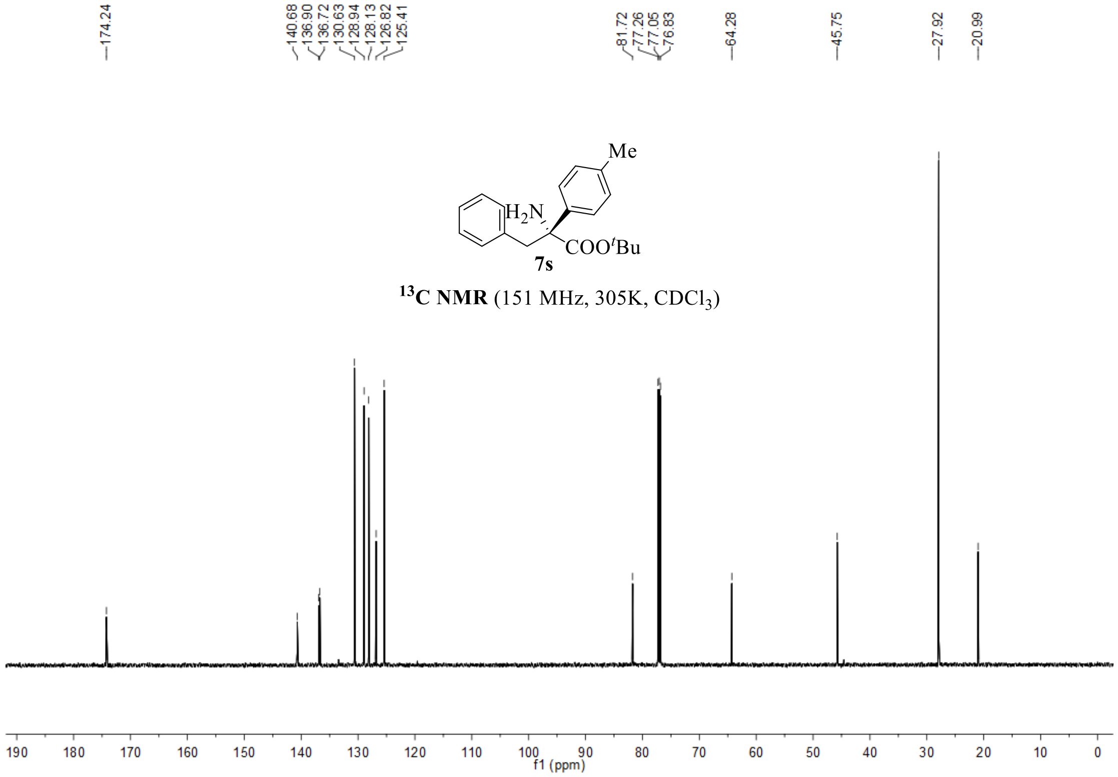

Supplementary Figure 58: NMR of compound 7s.

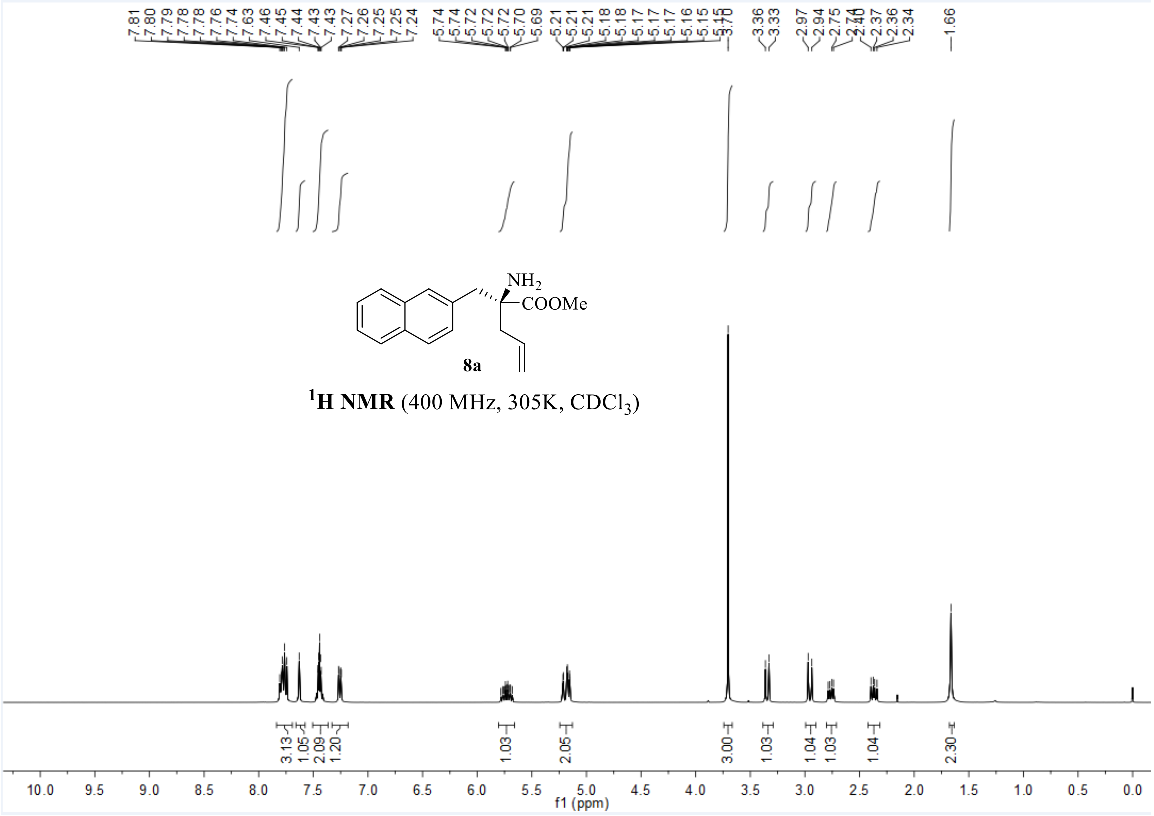

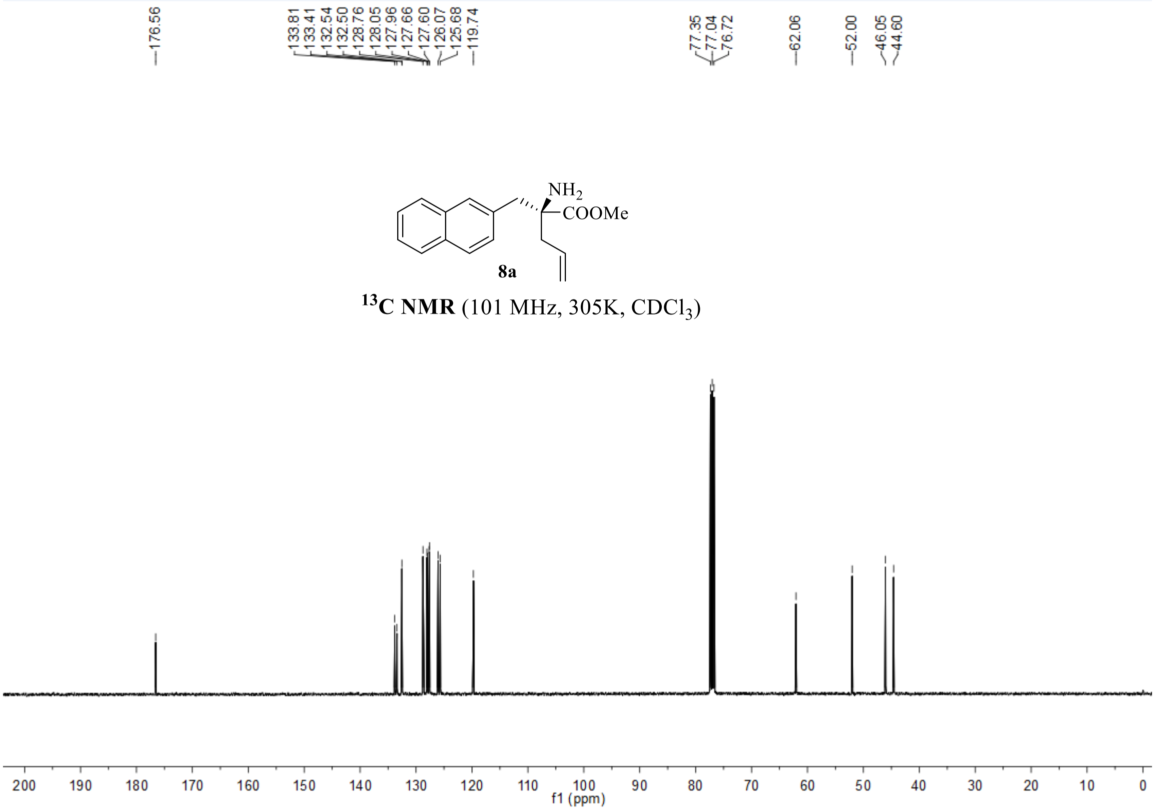

Supplementary Figure 59: NMR of compound 8a.

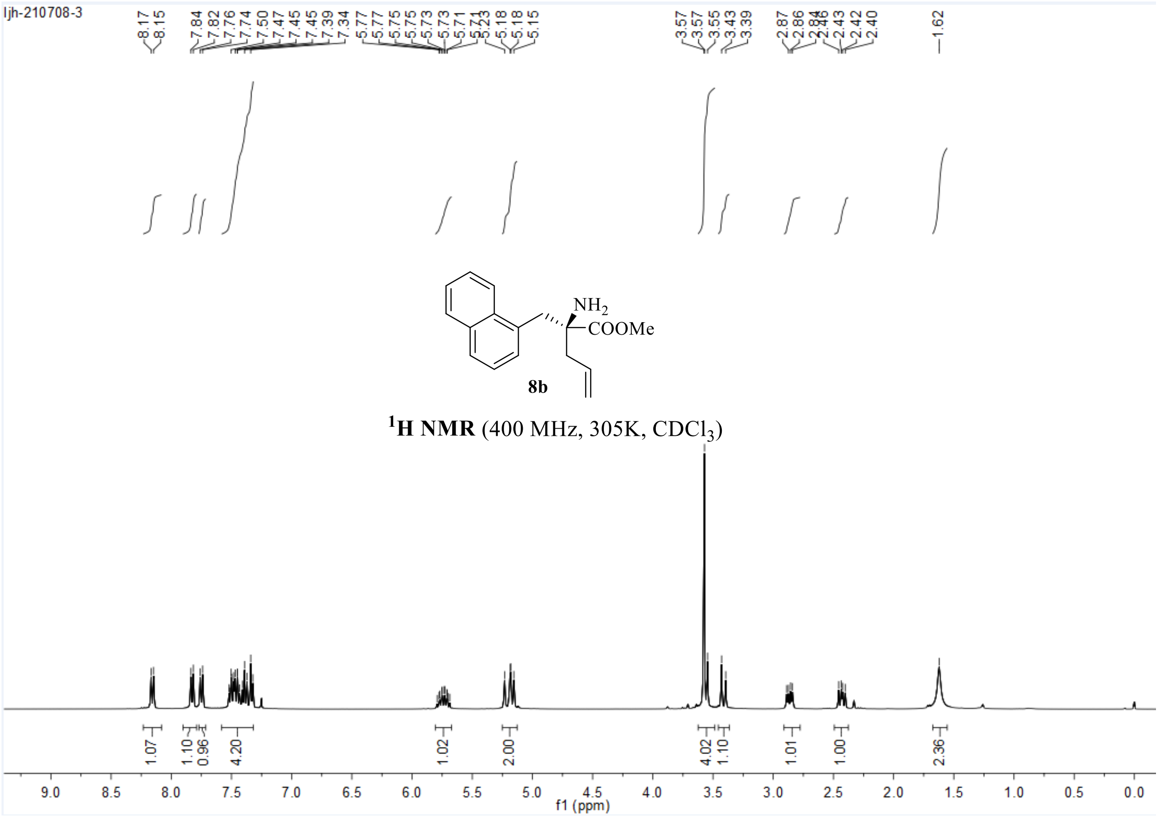

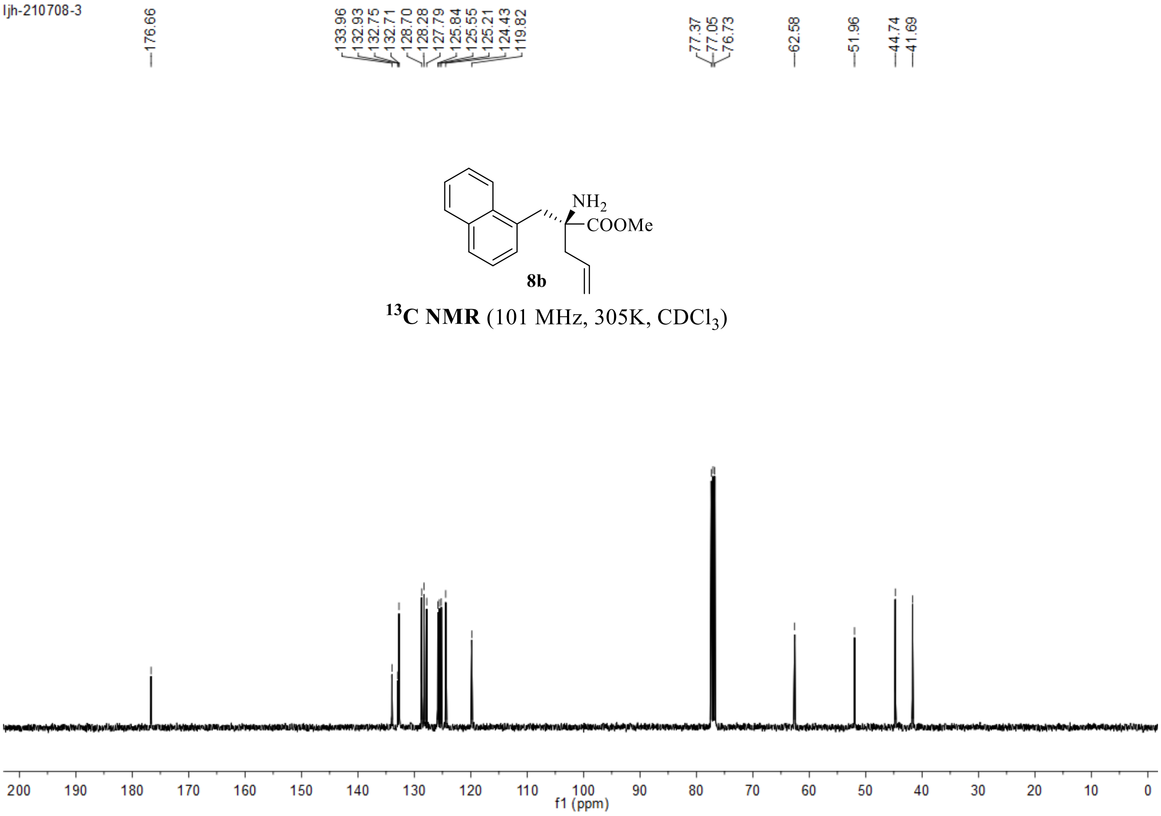

Supplementary Figure 60: NMR of compound 8b.

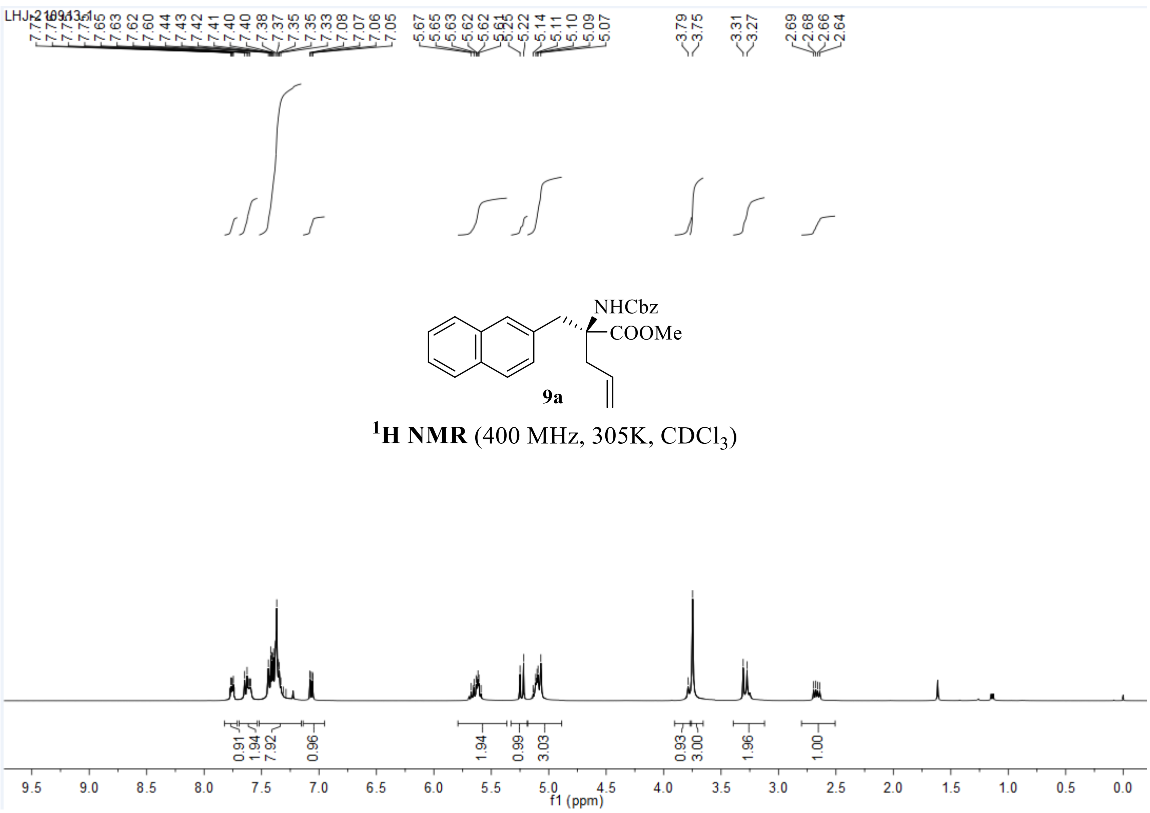

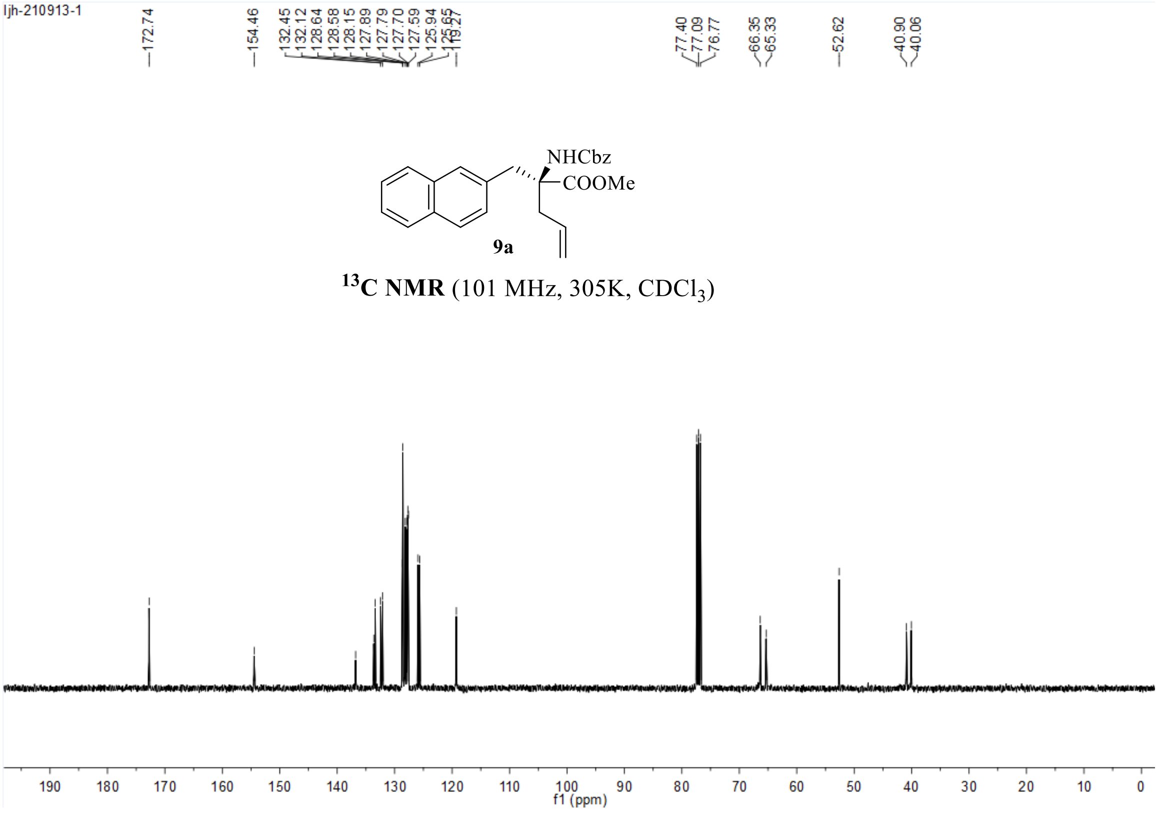

Supplementary Figure 61: NMR of compound 9a.

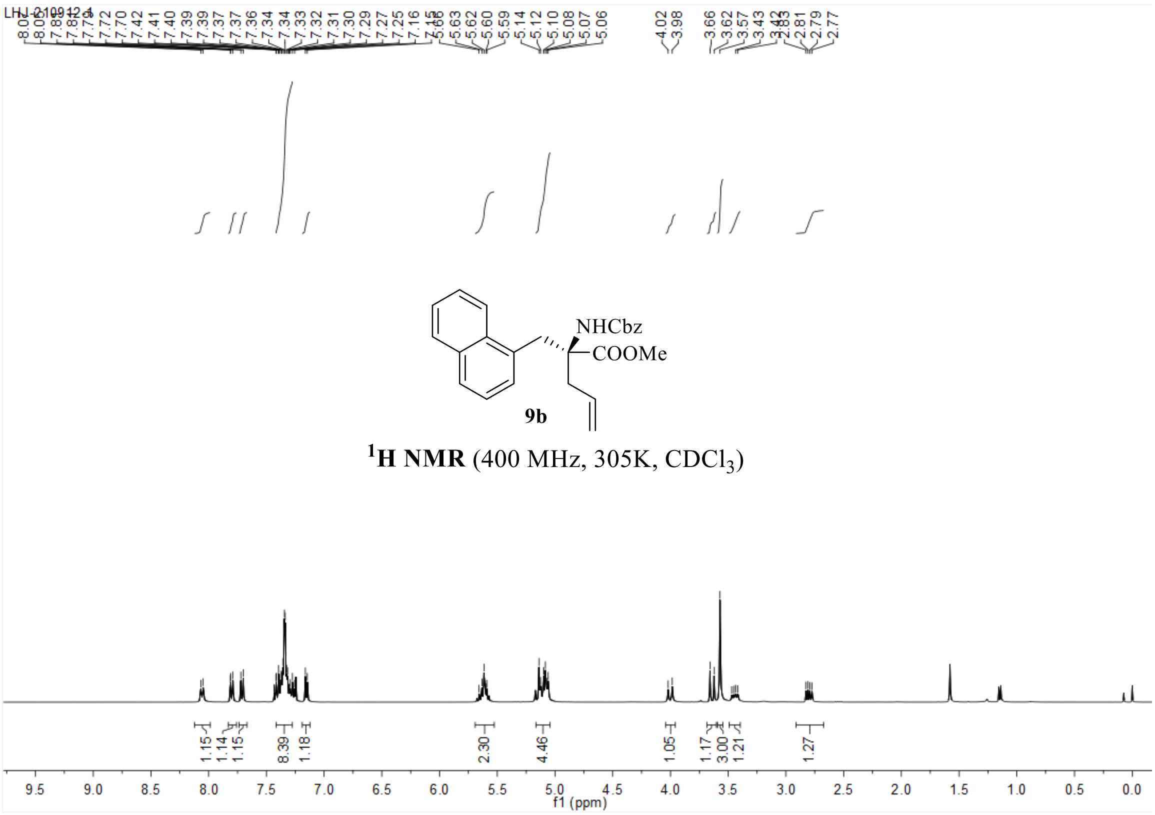

^{1}**H NMR** (400 MHz, 305K, $CDCl_3$)

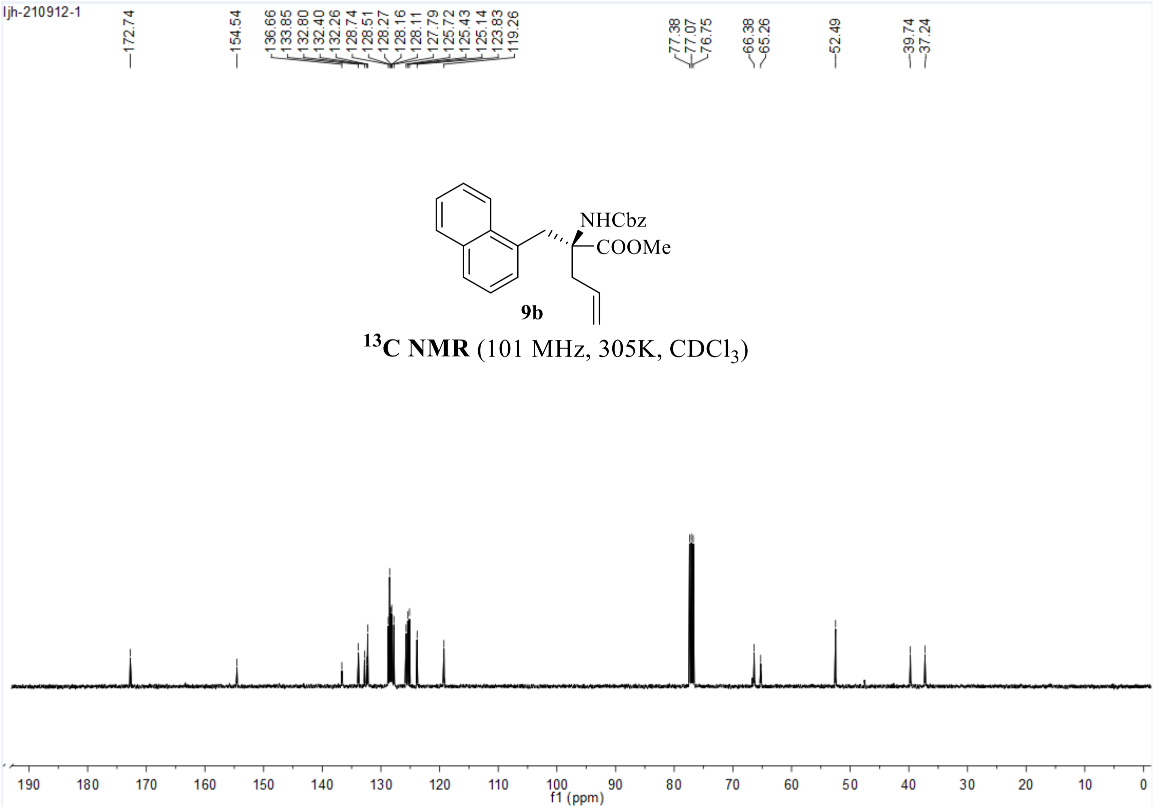

^{13}**C NMR** (101 MHz, 305K, $CDCl_3$)

Supplementary Figure 62: NMR of compound 9b.

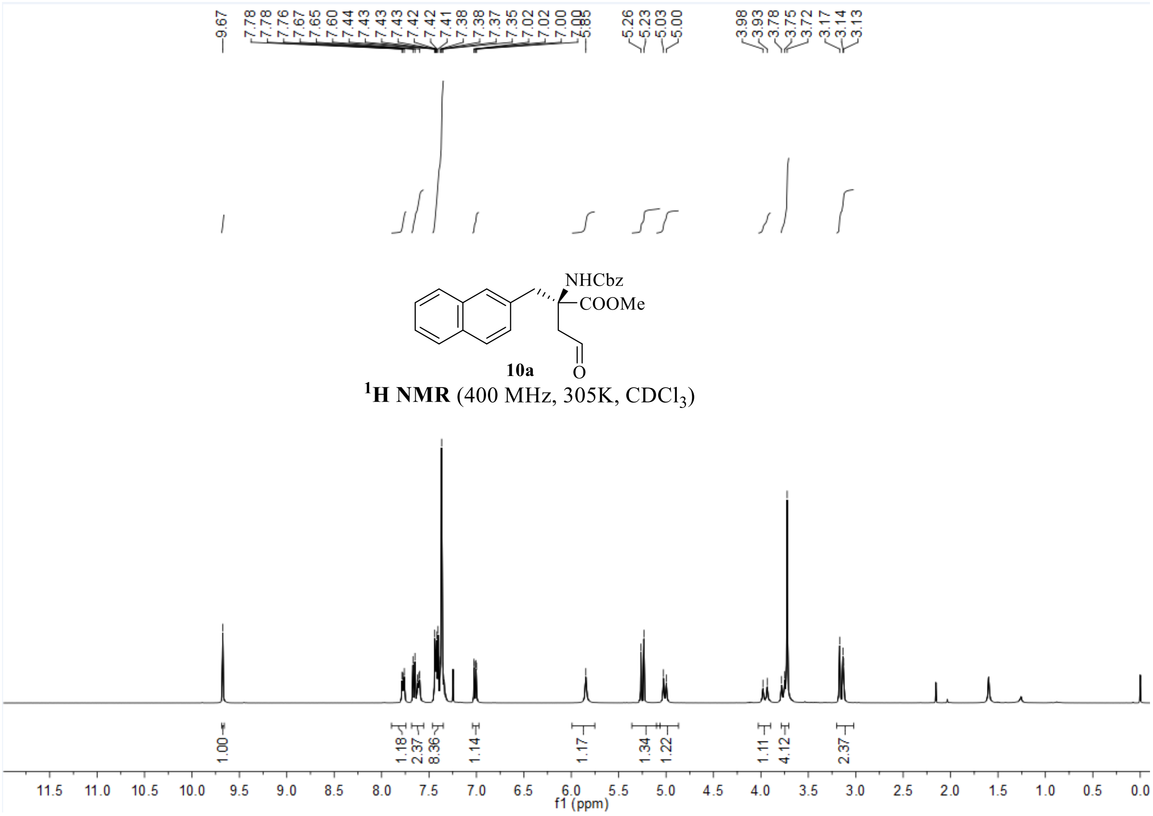

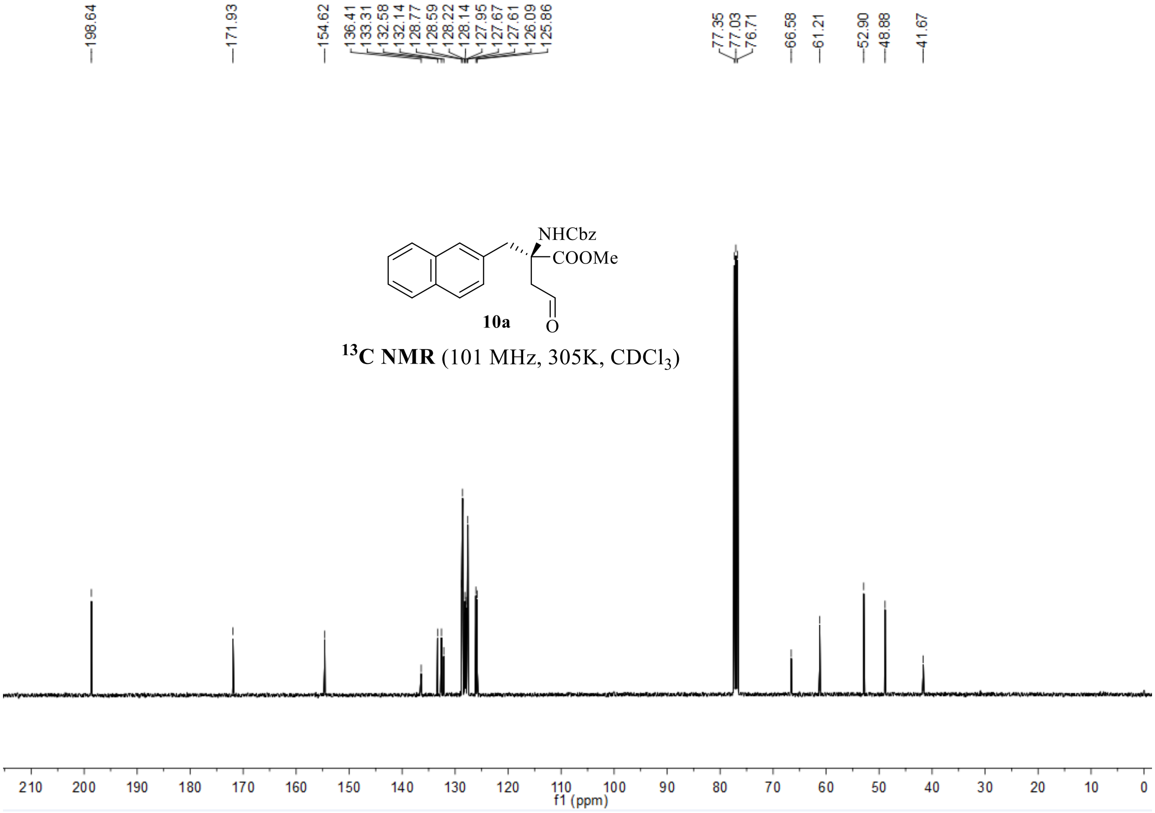

Supplementary Figure 63: NMR of compound 10a.

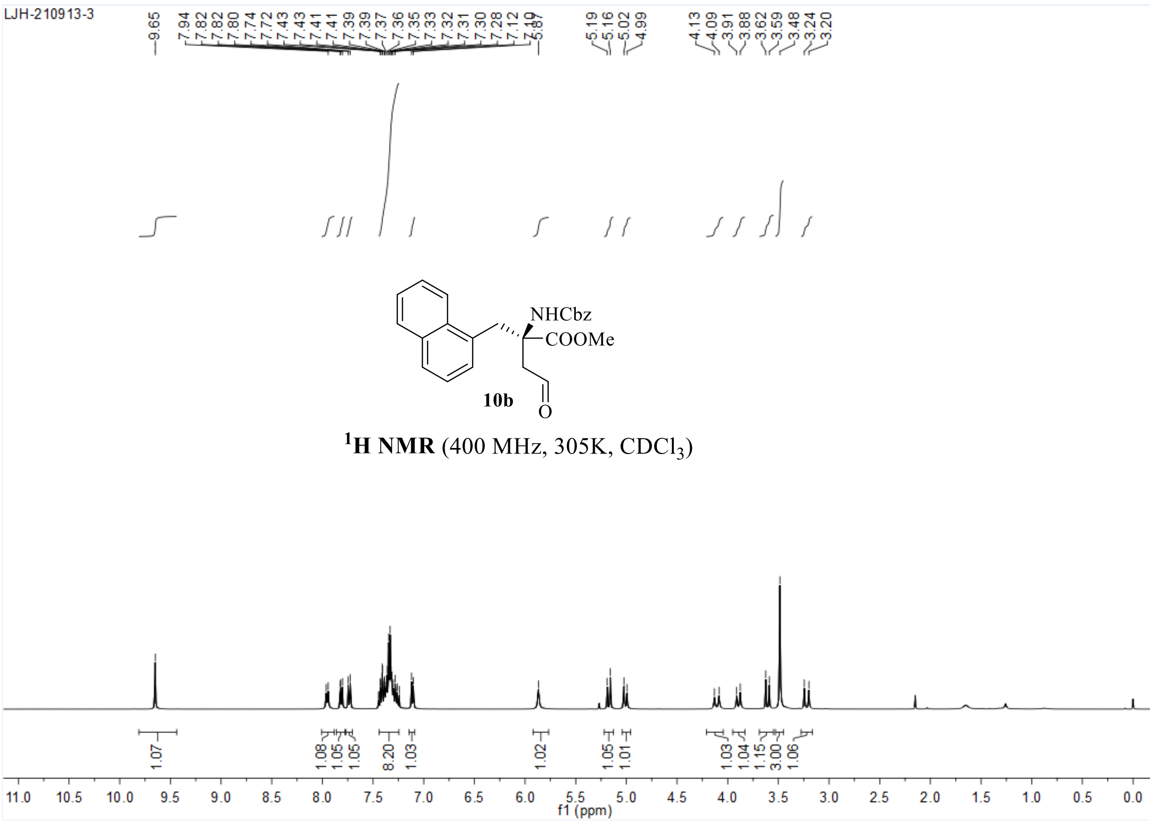

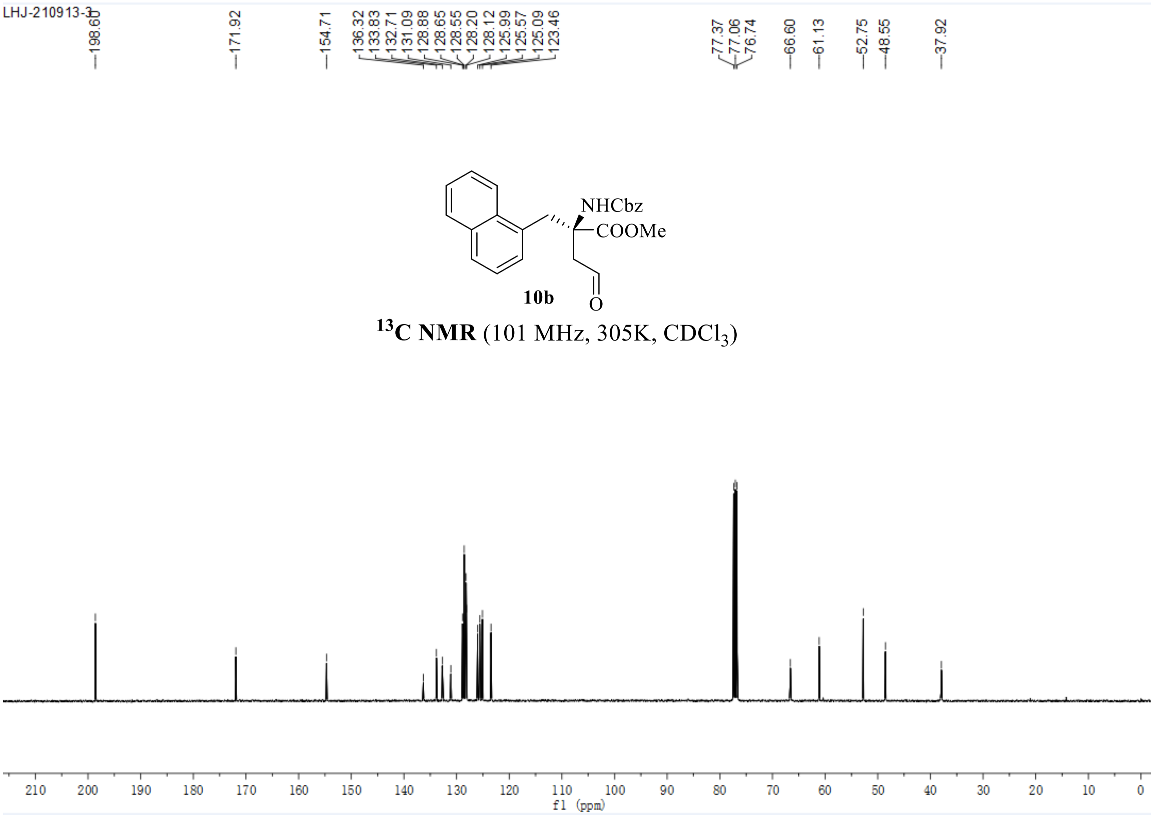

Supplementary Figure 64: NMR of compound 10b.

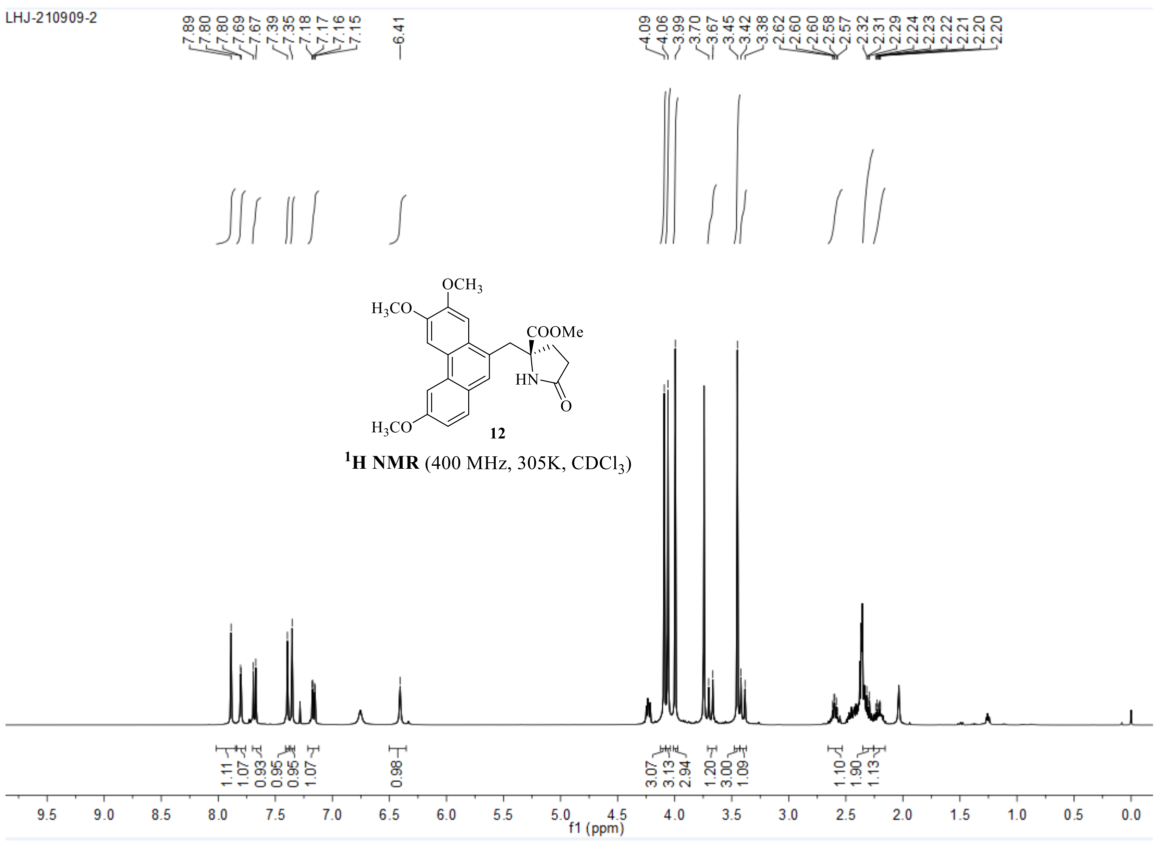

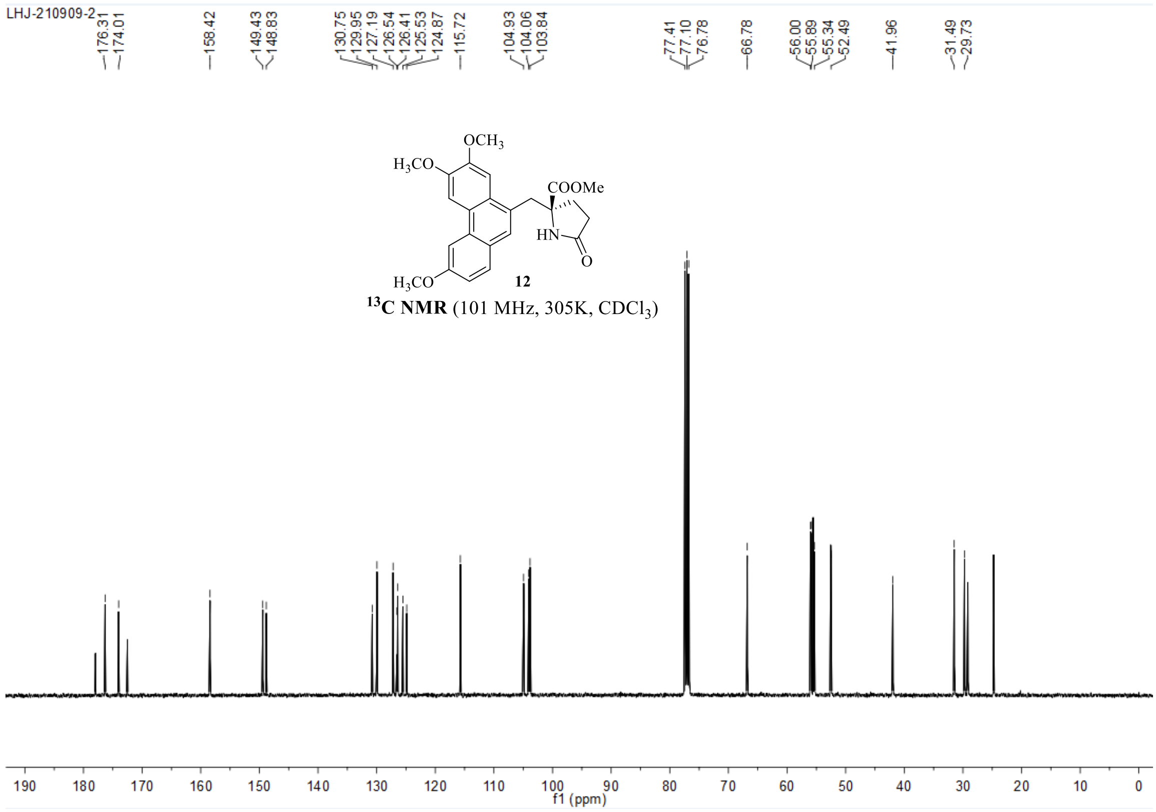

Supplementary Figure 65: NMR of compound 12.

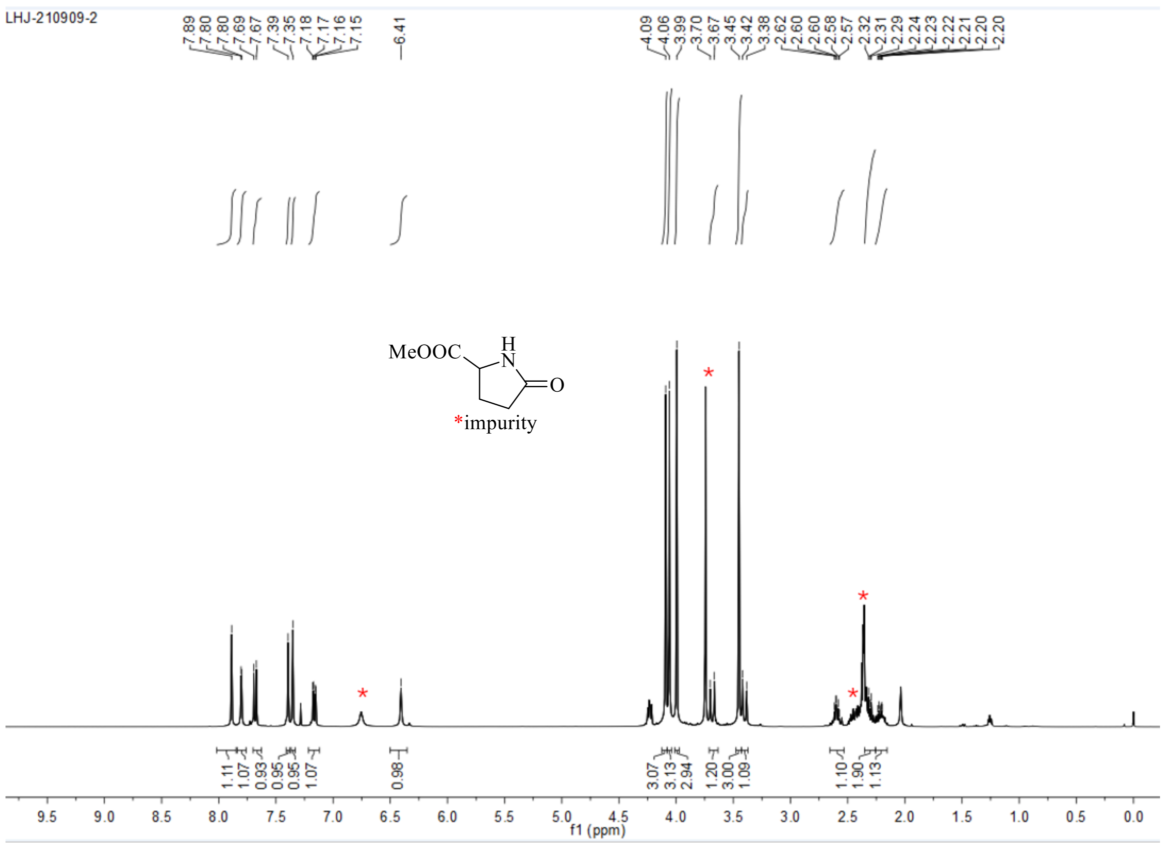

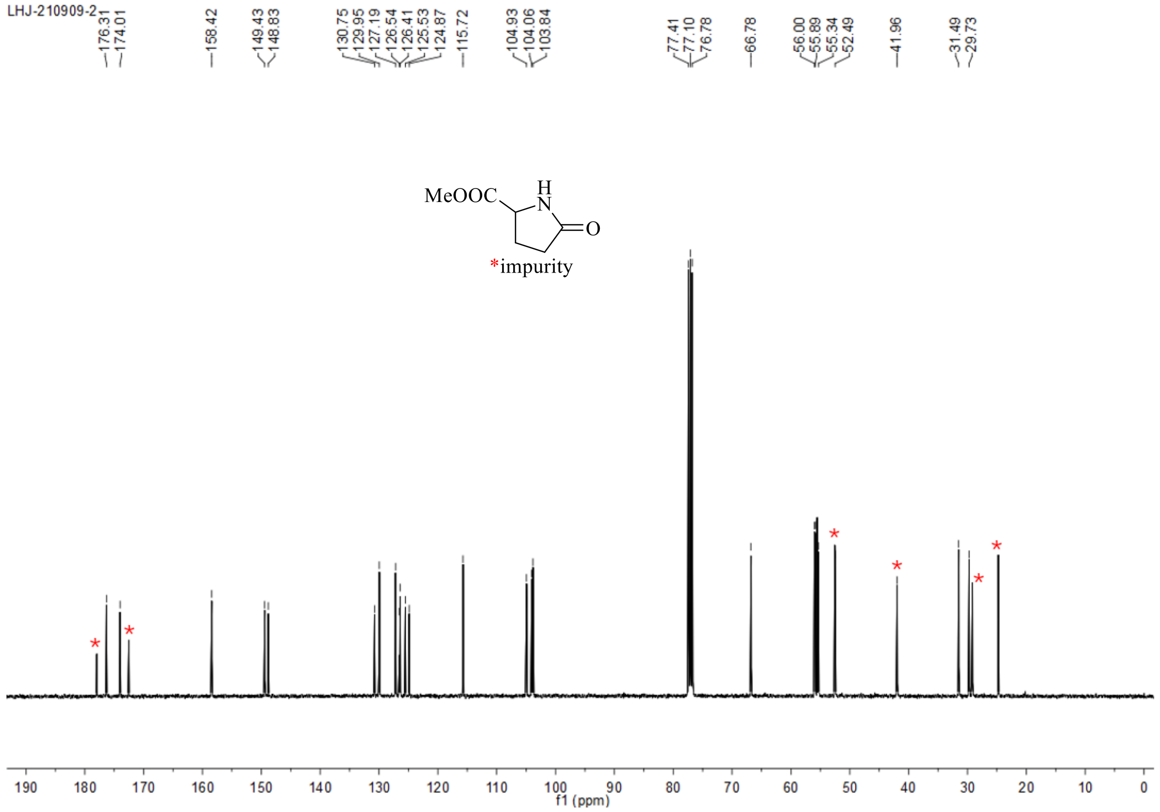

Supplementary Figure 66: NMR of the impurity γ–lactam (red marks).

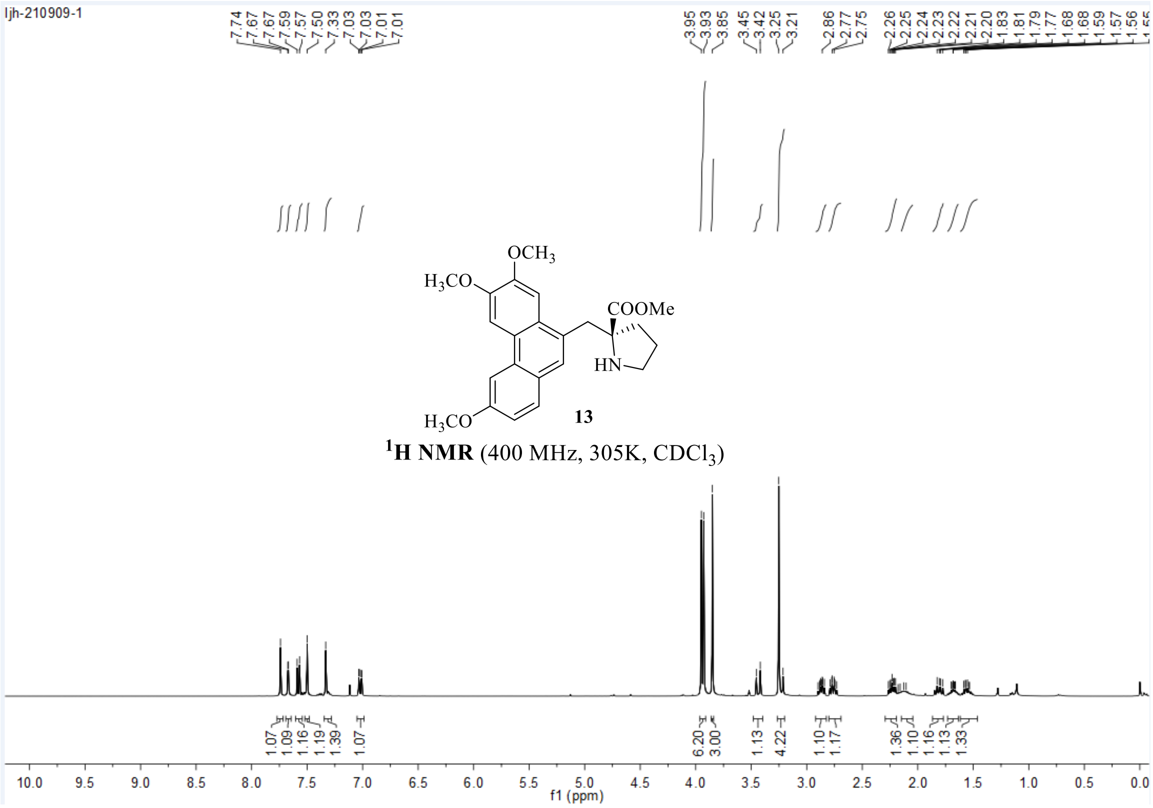

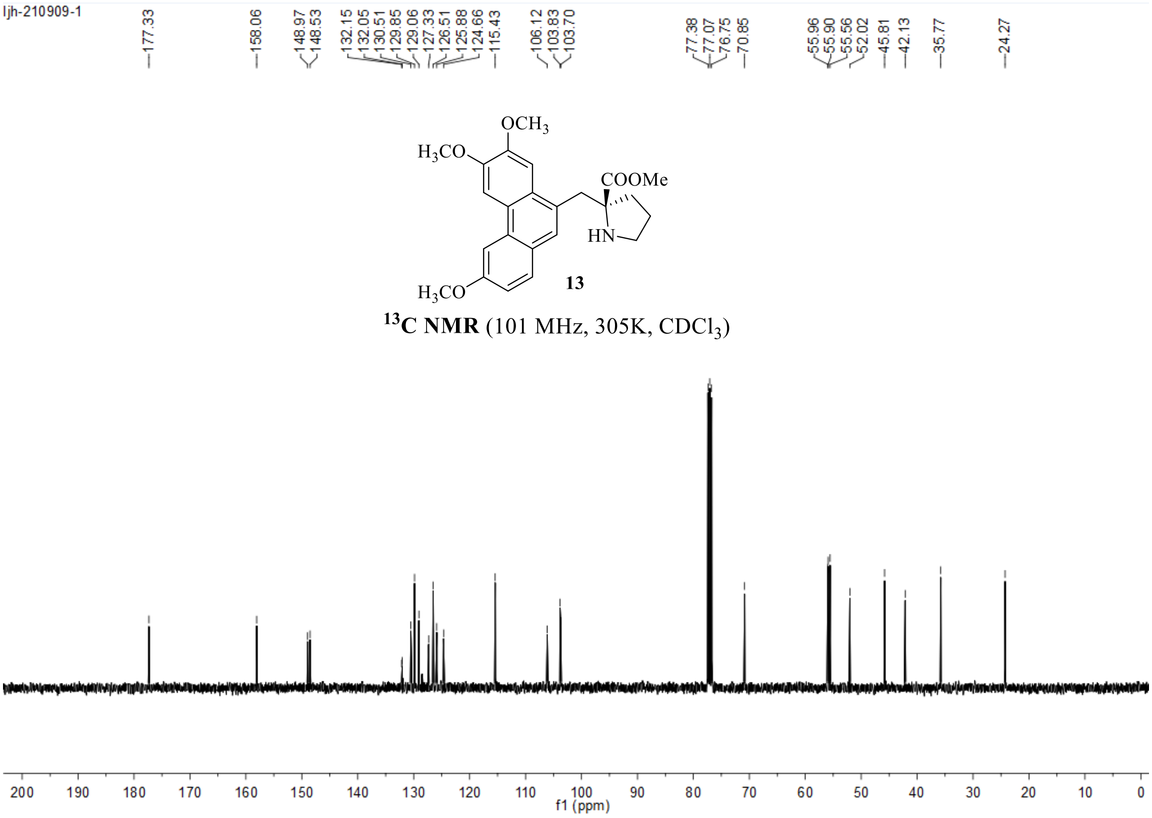

Supplementary Figure 67: NMR of compound 13.

8. Supplementary References

[1] Yochai, B.; Alfred, H. *J. Org. Chem.* **2000**, *65*, 6368-6380.

[2] Kasagani, V. P.; Kurma, S. H., Bhimapaka, C. R. *J. Org. Chem.* **2020**, *85*, 2976-2983.

[3] Chen, L.; Luo, M.-J.; Zhu, F.; Wen, W.; Guo, Q.-X., *J. Am. Chem. Soc.* **2018**, *140*, 9774-9780.

[4] Green, J. E.; Bender, D. M.; Jackson, S.; O'Donnell, M. J.; McCarthy, J. R. *Org. Lett.* **2009**, 11, 807-810.
